# Measuring the Surface Tension of Atmospheric Particles and Relevant Mixtures to Better Understand Key Atmospheric Processes

**DOI:** 10.1021/acs.chemrev.4c00173

**Published:** 2024-08-23

**Authors:** Manuella El Haber, Violaine Gérard, Judith Kleinheins, Corinne Ferronato, Barbara Nozière

**Affiliations:** † Institut de Recherches sur l’Environnement et la Catalyse de Lyon (IRCELYON), CNRS and Université Lyon 1, Villeurbanne 69626, France; ‡ Institute for Atmospheric and Climate Science, ETH Zürich, Universitätstrasse 16, 8092 Zürich, Switzerland; § Department of Chemistry, KTH Royal Institute of Technology, Stockholm 114 28, Sweden

## Abstract

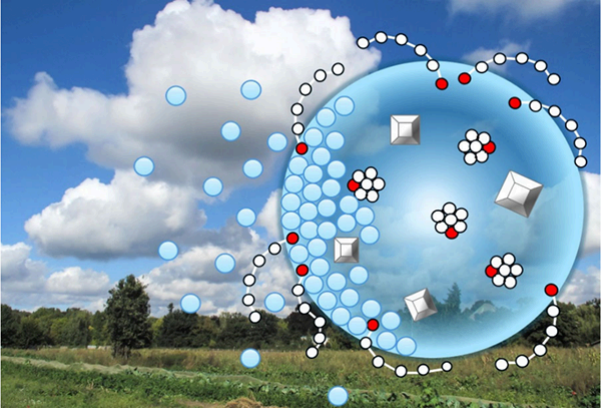

Aerosol and aqueous particles are ubiquitous in Earth’s atmosphere and play key roles in geochemical processes such as natural chemical cycles, cloud and fog formation, air pollution, visibility, climate forcing, etc. The surface tension of atmospheric particles can affect their size distribution, condensational growth, evaporation, and exchange of chemicals with the atmosphere, which, in turn, are important in the above-mentioned geochemical processes. However, because measuring this quantity is challenging, its role in atmospheric processes was dismissed for decades. Over the last 15 years, this field of research has seen some tremendous developments and is rapidly evolving. This review presents the state-of-the-art of this subject focusing on the experimental approaches. It also presents a unique inventory of experimental adsorption isotherms for over 130 mixtures of organic compounds in water of relevance for model development and validation. Potential future areas of research seeking to better determine the surface tension of atmospheric particles, better constrain laboratory investigations, or better understand the role of surface tension in various atmospheric processes, are discussed. We hope that this review appeals not only to atmospheric scientists but also to researchers from other fields, who could help identify new approaches and solutions to the current challenges.

## 1 Introduction

Surface tension is a key parameter that controls the shape and size of liquid particles present in another medium and the exchange of matter across their interface. It is thus expected to affect some important properties of the liquid particles present in Earth’s atmosphere and the processes in which they are involved, such as their formation and growth, size distribution, potentially their chemical evolution, and optical properties, all of which are important for the atmosphere and climate. However, measuring the surface tension of atmospheric particles is challenging. In fact, at the time of publication of this review, the surface tension of individual atmospheric particles has not yet been directly measured. Thus, for decades, the role of this parameter in atmospheric processes was neglected, and its value was systematically assumed to be equal to that of pure water. This has changed over the last 15 years with the emergence of various experimental approaches that are now giving some information about the surface tension of atmospheric aerosol samples and relevant laboratory mixtures or particles. This review presents an overview of this field of research, including a presentation of some basic concepts related to surface tension, emphasizing a molecular-level description (Section 1), a discussion of the atmospheric processes, in which surface tension is expected to play a role (Section 2), a presentation of the relevant measurement techniques (Section 3), and the current knowledge of the surface tension of atmospheric particles (Section 5). Section 4 presents a unique inventory of the experimental adsorption isotherms for about 130 water/organic mixtures, which can be used to develop relationships between molecular structure and surface tension in models or to validate other types of surface tension models for atmospheric particles. The last section of this review (Section 6) discusses the remaining challenges and identifies future areas of research such as future technical developments that would better constrain fundamental (laboratory) investigations, future investigations improving the understanding of atmospheric processes, and future areas of research addressing more specifically environmental issues. Previous reviews have discussed some aspects of the surface tension of atmospheric particles, but primarily focusing on specific processes, mostly cloud droplet formation,^1,2^ or on the compounds present at the surface of atmospheric aerosols and their properties.^3,4^ Other reviews have focused on the measurements techniques for surface tension.^5,6^ The sections overlapping with these previous articles have been kept as concise as possible in the present review to emphasize the complementary aspects and updates, and we refer to these articles for more complete information. Surface tension models themselves are beyond the scope of this review, and we refer to previous articles and reviews^7−14^ for more information on these theoretical approaches. We hope that, beyond atmospheric chemistry, this review raises the interest of chemists and chemical physicists from other fields to develop cross-disciplinary collaborations and possibly identify new solutions to the current challenges.

### 1.1 Definitions and Key Concepts

The following definitions are basic and can be found in textbooks. They are presented only briefly to clarify potential discrepancies between the concepts used in atmospheric chemistry and in other fields of chemistry and chemical physics. They are also presented to propose, whenever possible, a molecular description of the relevant processes and properties, most of which are inspired by the book of Rosen and Kunjappu.^15^

The historical definition of the surface tension, σ, by Gibbs^16^ is that of a thermodynamic and, thus, a macroscopic quantity: the energy per unit of surface area corresponding to the change *dA* to a surface area *A*, resulting from applying the element of work, *dW*:
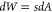
1where σ is usually expressed in mN m^–1^. Note that the surface tension is represented by the symbol “γ” in most fields of chemistry and chemical physics but by “σ” in the atmospheric chemical literature.

It has been recently demonstrated that the surface tension of solids is not related to their surface energy, thus that the Gibbs definition of surface tension does not apply to them.^17^ The present review thus discusses essentially the surface tension of liquid particles and mixtures. Surface tension effects can, however, been considered in solids such as the ice particles discussed in Section 2.4. Evidence has also been reported that solid particles such as soot coated with surface-active organic compounds, such as oleic acid or adipic acid, were more efficient in condensing water,^18,19^ which could potentially be attributed to surface tension effects.

Surface tension has a constant value (static surface tension) if the system of interest (bulk liquid or particle in contact with another phase) is equilibrated. However, with a rapidly changing system or interface, the surface tension will converge toward the new equilibrium value with some delay (dynamic surface tension), mostly due to diffusion effects.^20,21^ For atmospheric particles, such rapid changes in the interface occur, for instance, at the point of activation of forming water droplets or during the nucleation of new particles (see Section 2). The diffusion coefficients estimated for surfactants from atmospheric aerosols suggest that their diffusion time to the surface of a 1 μm-radius particle would be of the order of 1–100 s.^22^ Water droplet activation processes in the atmosphere are estimated to occur over the same time scale. Experimental measurements of the dynamic surface tension of aqueous solutions of amphiphilic surfactants (see Section 1.2.3 below) have shown that the largest differences between the static and dynamic surface tension occur at time scales shorter than 1 s.^20,23^ These dynamic surface tension effects are thus expected to have limited impacts on the atmospheric processes involving particles, as confirmed by recent measurements.^24^ The remainder of this review thus focuses on the static surface tension.

The International Union for Pure and Applied Chemistry (IUPAC)^25^ defines a surfactant as “a substance which reduces the surface tension of the medium in which it is present”. The above definition implies that surfactants are at low molar fraction in the medium (otherwise they are part of the medium itself) so that this condition is explicit in the definition used in surfactant science: “a substance which, at low concentration, reduces the surface tension of the medium”.^15^ Since the term “surfactant” is the contraction of “surface-active agent”,^15,25^ both terms have the same meaning, as well as similar terms such as “surface-active compounds”. In the atmospheric chemical literature there is often confusion between surfactants and organic coatings, surface layers, or surface films.^4^ The latter differ from surfactants, as they form separate phases at the surface of the liquids without (necessarily) lowering their surface tension. Organic coatings, surface layers, and surface films will not be further discussed in this review, unless they clearly involve surface tension effects, and we refer to previous reviews^3,4^ for more information on such systems.

Since surfactants act on the surface tension at low molar fraction, they can be described, from a molecular point of view, as molecules present in small concentration in a solvent. In atmospheric chemistry the solvent of highest relevance is water but, as will be underlined in Section 2, surface tension can also play a role in particles made of other substances, such as concentrated sulfuric acid (as in newly nucleated particles) or organic liquids (as in Secondary Organic Aerosols). Surfactant molecules reduce the surface tension by replacing a small fraction of the solvent molecules at the surface and weakening the interactions between them.^15^ To achieve this, the surfactant needs to be not entirely soluble in the solvent, i.e., is dissolved in the solvent only up to a specific concentration, beyond which it builds a separate phase on top of it. Their presence in the solvent thus results in a distortion of the solvent structure and in an increase of the free energy of the mixture.^15^ However, the surfactant also needs to be at least partly soluble in the solvent to avoid being expelled into the adjacent phase (e.g., into the gas for a liquid/gas system), since the mixture will tend to minimize its energy by reducing the contact between the surfactant and the solvent and expelling the surfactant to the surface. Most organic compounds fulfill this partial solubility criterion in aqueous mixtures, thus acting as a surfactant with various degrees of efficiency (see Section 4). However, sugars, which are highly soluble in water, do not significantly reduce the surface tension of aqueous solutions (in fact, they tend to increase it). Similarly, organic compounds do not act as surfactants in organic solvents or particles in which they are fully soluble. At the opposite, the most efficient surfactants in aqueous solutions are amphiphilic compounds, which possess both a water-soluble (hydrophilic) moiety and nonwater-soluble (hydrophobic) molecular chains. The different types of surfactants potentially present in atmospheric aerosols have been discussed in previous reviews^3,4^ and the role of the molecular structure on the surfactant efficiency will be further discussed in Section 4.

#### 1.1.1 Variation of the Surface Tension with Surfactant Concentration: Adsorption Isotherms

Building on the thermodynamic description of the surface tension (eq 1), the relationship between the surface concentration of surfactant, Γ, and the corresponding bulk concentration, *C*, is described with an adsorption isotherm, where the term “isotherm” indicates that it is established for a given temperature. However, for liquid/liquid and liquid/gas systems, the surfactant concentration at the interface can not be easily measured, and the Gibbs adsorption equation is rather expressed as a relationship between the surface tension of the mixture, σ, the surface concentration, Γ_*i*_, and chemical potential, μ*_i_* (thus, the bulk concentration) of the different components “*i*” present in the mixture:
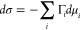
2

For each component in the system
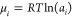
3where *a_i_* = activity of compound *i*, *R* the gas constant, and *T* temperature. Thus, for system made of a solvent (for instance, water) with *a*_1_ ∼ 1 and a surfactant with *a*_2_ ∼ *C*, the bulk concentration, and Γ_2_ = Γ_m_,
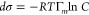
4^15,20^

Eq 4 thus gives the variation of σ with the concentration of surfactant, *C*. Integrating eq 4 is not straightforward, and various assumptions can be used. An empirical equation that is often used to approximate the integration of eq 4 is the Szyszkowski equation:
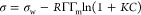
5where *K* is a constant depending on the surfactant, and σ_w_ is the surface tension of the pure solvent (here, assumed to be water). Numerous examples of adsorption isotherms are presented in Section 4 of this review, displaying a large range of shapes. Nonamphiphilic compounds accumulate at the surface of a mixture proportionally to their concentration in the bulk. This results in isotherms displaying two main regions: a first region at low bulk concentration, *C*, where σ ∼ σ_w_, followed by a second region where σ decreases with *C*, until the concentration reaches the maximum solubility of the compound in water. Such isotherms are often described with a Szyszkowski-type equation (eq 5). By contrast, amphiphilic surfactants accumulate essentially at the surface (or interface) of a mixture so that their bulk concentration, *C*, is very small until the surface reaches saturation. When surface saturation is reached the surface tension is at its minimum value, σ_o_. Beyond this concentration, the surfactant molecules do not dissolve in the bulk but produce micelles, which are highly organized phases and distinct from the bulk solution phase. The surface tension of the mixture does not further decrease with *C* but remains constant at σ_o_. The value of *C* for which surface saturation is reached is thus called Critical Micelle Concentration (CMC). As a result, the adsorption isotherms for amphiphilic compounds display three distinct regions: a region at low *C* where σ = σ_w_, an intermediate region where σ decreases sharply with *C* to reach σ_o_, and a third region at large *C* where σ is constant and equal to σ_o_ (this last part is not predicted by the Szyszkowski equation, eq 5).

### 1.2 Surfactant Properties and External Parameters Affecting the Surface Tension

This section lists the various parameters affecting the surface tension of particles and mixtures, which includes both properties of the surfactants themselves (semisoluble surfactants, amphiphilic surfactants, etc.) and properties resulting from the medium in which they are present (mixing effects, geometric effects, etc.).

#### 1.2.1 In Aqueous Media: Hydrogen Bond and Solvation

In aqueous solutions, the most important type of mixtures for atmospheric particles, the main molecular interactions between the solvent molecules (water) are hydrogen bonds. These bonds are responsible for the exceptionally large surface tension of water, σ (293 K) = 72.8 mN m^–1^. Although the strongest hydrogen bonds are those between water molecules, hydrogen bonding can also occur in other solvents containing hydrogen and electronegative atoms, such as oxygen, nitrogen, or halogen atoms: sugars, alcohols, organic acids, for instance. However, many organic compounds form only weak or no hydrogen bonds and, as solvents, have a surface tension markedly lower than that of water (typically, σ ≤ 30 mN m^–1^).^26^

As a consequence, semisoluble organic molecules present in aqueous mixtures, which do not form significant hydrogen bonds with water, weaken the hydrogen bonds between the water molecules and reduce the surface tension of the mixtures (see examples for many different organic molecules in Section 4). This effect is, however, modest and usually requires large bulk concentrations (>1 M) to substantially lower the surface tension (δσ > 10 mN m^–1^). It also decreases rapidly with dilution. Highly water-soluble organic molecules, such as sugars, which form substantial hydrogen bonds with water, do not act as surfactants as they do not fulfill the condition of partial solubility of surfactants. In addition, the formation of solvation shells (or solvation cages) around these compounds creates new structures for the water molecules, thus reinforcing the cohesion of the solvent and increasing the surface tension (see Section 4).

#### 1.2.2 In Ion-Containing Media: Electrostatic Interactions

Ions, especially inorganic ones, are ubiquitous in the natural environment, including atmospheric aerosols. They strongly affect the interactions between the solvent molecules in which they are present and, thereby, the surface tension because they generate strong electrostatic forces. Depending on the solvent and on their charge and size, ions can either strengthen or weaken the cohesion between the solvent molecules and, thus, the surface tension. Inorganic salts such as NaCl and (NH_4_)_2_SO_4_ and, therefore, ions such as Na^+^, Cl^–^, SO_4_^2–^, and NH_4_^+^ are ubiquitous and abundant in atmospheric particles. However, because they are fully soluble in aqueous mixtures, they do not act as a surfactant. However, the strong electrostatic fields that they produce reinforce the cohesions between the water molecules, resulting in the well-known effect of inorganic salts in increasing the surface tension of aqueous mixtures compared with pure water. The intensity of these effects depends on ions with their charge/radius ratio. Thus, among anions, SO_4_^2–^ has a larger effect than Cl^–^ and, among cations, NH_4_^+^, has stronger effects than Na^+^.^27,28^ However, as electrostatic forces decrease rapidly with the distance between the charges, all of these effects decrease rapidly with dilution. Note, however, that the electrostatic interactions generated by dissolved inorganic ions also affect other components of solutions beside the solvent molecules and that their overall effects on the surface tension can be opposite to their effect in water alone (see Section 1.2.5 below).

#### 1.2.3 Amphiphilic Interactions

**Figure 1:**
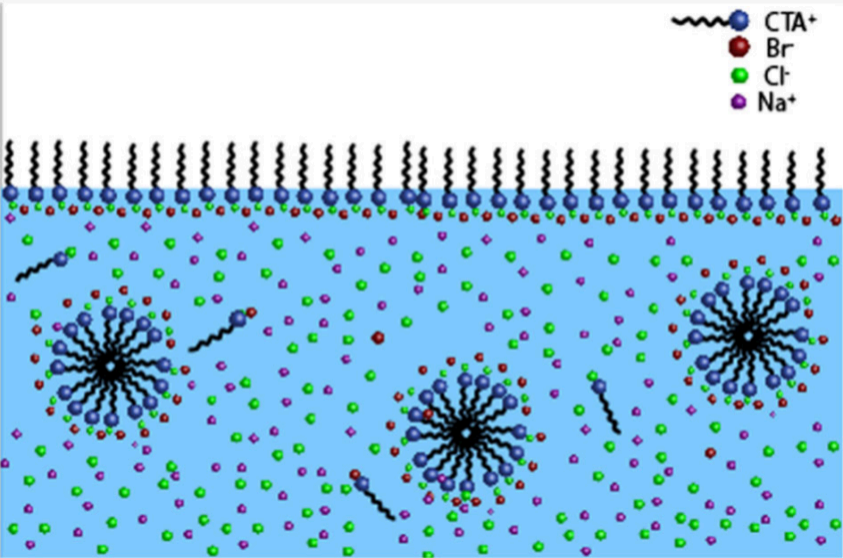
Illustration of the salting out and solvation of an amphiphilic surfactant (represented by the molecules with a long “tail”) by inorganic ions (red and green dots) and distribution of the different components between the surface and the bulk of an aqueous solution. From ref (60). Copyright 2020 American Chemical Society. Licensed under the CC-BY-NC-ND.

As explained above, the most efficient surfactants for aqueous mixtures are amphiphilic molecules (also called amphipathic), carrying both a hydrophilic group (water-soluble) and one or more hydrophobic (=nonwater-soluble) groups (usually organic chains). In a water/air system, their hydrophobic groups ensure that their presence is essentially limited to the surface, the hydrophobic chains being in the air above the surface, where they can adopt a range of conformations (Figure 1). At the same time, their water-soluble moieties ensure their “anchoring” in the aqueous phase and prevent the molecules from being expelled from the aqueous phase and forming a separate phase above the surface. The physical process by which amphiphilic molecules lower the surface tension of aqueous solutions has been the subject of numerous experimental and theoretical studies (the latter mostly by molecular dynamics simulations).^29,30^ This effect is generally accepted to result from micromechanical “push-pull” effects of the hydrophobic chains perpendicular to the surface, as evidenced, for instance, by correlations between the surface tension and the chain length and rigidity of different amphiphilic surfactants.^29^ Depending on their water-hydrophilic group, amphiphilic compounds are classified as anionic, cationic, zwitterionic, or nonionic.^15^ These properties affect, for instance, their affinity toward negatively or positively charged surfaces.

#### 1.2.4 Macromolecules

Some organic molecules found in atmospheric aerosols contain more than 20 C atoms and have a molecular weight of several hundred Da or more. From the point of view of atmospheric chemistry, they can be considered as macromolecules, even though in chemistry and biochemistry this term is usually employed for much larger molecules. They include biopolymers (polysaccharides such as cellulose, chitosan, chitin, starch, etc.; polypeptides such as collagen and gelatin, etc.),^31,32^ polymers from biomass burning (some Polycyclic Aromatic Hydrocarbons, PAHs, and graphitic material, etc.),^33−37^ gels and hydrogels (extracellular polymeric substances),^38−40^ and polyphenolic compounds (lignin, humic, and fulvic substances).^41−45^ Their large molecular structure limits their solubility in water, thus allowing them to act as a surfactant. Some of those reported in atmospheric particles, and their commercial reference, have been shown to reduce the surface tension of aqueous solutions (see also Section 4, Table 5). They include fulvic acids (Suwannee River Fulvic Acid, SRFA^41,44,46−48^ and Nordic Aquatic Fulvic Acids, NAFAs^41,49,50^), commercial humic acid,^48,51,52^ aerosol-extracted Humic-Like substances (HULISs),^41,42,44^ and microbial or environmentally extracted Extracellular Polymeric Substances (EPSs).^53−55^ The mode of action of these macromolecular compounds on the surface tension of aqueous mixtures is unclear. Aqueous solutions of macromolecules have been shown to undergo liquid–liquid phase separation and organize internally to lead to ultralow surface tension (down to 1 mN m^–1^).^56,57^ Such effects can thus not be excluded with humic substances, HULISs, and EPSs. However, humic and fulvic substances have also been shown to have some amphiphilic properties,^58^ and EPSs to contain non-negligible fractions of amphiphilic compounds.^59^ Thus, their surface tension properties could also partly result from these amphiphilic properties.

#### 1.2.5 Mixing Effects: Salting Out

In contrast with the increase in surface tension resulting from the presence of inorganic ions alone in water, adding inorganic ions to aqueous mixtures containing organic surfactants further reduces the surface tension. This effect has been observed both with nonamphiphilic, semisoluble organic compounds^41,49,61−68^ and with amphiphilic surfactants.^60,68−74^ It is known to result from the “salting out” of the organic molecules toward the surface, and is quantified by a Setschenow (or salting out) coefficient, *K*_s_ [M^–1^].^15,75−77^ In this process, the strong electrostatic interactions result in a strong reorganization of the water molecules as solvation shells (or “cages”) around the ions (Figure 1), thus lowering the solvation of the organic compounds and increasing the energy of the mixture.^15^ To minimize this energy, the organic molecules are “pushed” to the surface, resulting in larger surface concentration and thus lower surface tension than in the absence of salt. It also implies that surface saturation is reached with lower surfactant concentration and thus that the CMC is shifted to lower concentrations. As for the electrostatic interactions described above in Section 1.2.2, different anions and cations have different efficiencies in these processes, depending on their charge/radius ratio. Thus, SO_4_^2–^ and NH_4_^+^ have stronger salting out effects than Cl^–^ and Na^+^,^15^ resulting in a more efficient surface tension reduction.

The salting out of organic surfactants by inorganic salts and additional reduction of the surface tension has been evidenced not only with bulk mixtures but also with submicrometer particles, both artificial ones^49,67,78^ and particles generated from surfactants extracted from atmospheric aerosols.^64^ In all cases, combining surface tension measurements with CN/CCN or CCN growth factor measurements (where “CN” stands for “Condensation Nuclei” and “CCN” for “Cloud Condensation Nuclei”) revealed that adding inorganic salts to the organic particles further decreased their critical supersaturation, which was unambiguously attributed to a decrease in the surface tension rather than to hygroscopic effects. In some cases, the critical supersaturation obtained with the mixed particles was even below that obtained with the salt alone,^49,64^ thus evidencing synergistic effects (see also definition in next paragraph) even in activated particles. This was shown, in particular, for particles made of organic fractions extracted from atmospheric (biomass burning) aerosols, displaying surface-active properties, with σ = 35–68 mN m^–1^. Adding (NH_4_)_2_SO_4_ to these particles reduced the critical supersaturation to below the value for pure (NH_4_)_2_SO_4_ particles.^64^ Salting out effects are thus important to take into account in atmospheric aerosols.

#### 1.2.6 Mixing Effects: Nonideality, Synergism, and Antagonism

Atmospheric particles contain different types of organic compounds, which can affect the surface tension in different ways than simply adding their individual contributions. The simplest description of such mixtures is a two-component organic mixture including an organic acid that is abundant in atmospheric aerosols, such as oxalic, succinic acid, and an amphiphilic surfactant in a much smaller molar fraction. If these two components do not interact molecularly, i.e., have no direct or induced electrostatic attraction or repulsion between them (or at least not more than between each compound and the solvent) the mixture is defined as ideal.^79^ In that case, its overall properties, in particular the surface tension and CMC, is simply the combination of the contributions of each component, weighted by their relative molar fractions. However, if the organic components interact, the surface tension or CMC of the mixture can be lower or higher than expected for an ideal mixture, and the mixture is said to be nonideal. In some extreme cases, the overall surface tension can be even lower than both those for the pure components, and the mixture is said to be synergistic.^15^ Inversely, if the surface tension of the mixture is larger than those of the pure components, the mixture is antagonistic.^15^

Until now, mixing and ideality effects have mostly been studied for mixtures of different amphiphilic compounds. The molecular interactions between surfactants at the surface of a liquid and in the bulk during micelle formation are very different. At the surface, all the surfactant molecules have the same orientation, hydrophilic end in water and hydrophobic chains in the air above the surface. Thus, attraction or repulsion between surfactants can occur either between the hydrophilic ends at the surface or between the hydrophobic chains just above the surface. By contrast, the molecular interactions (attractions or repulsion) taking place in the bulk during micelle formation involve the entire surfactant molecules, which can take any orientation or conformation, thus leading to a wide variety of micelle structures. These different interactions at the surface and in the bulk explain why some mixtures of surfactants can be nonideal in surface tension but ideal in CMC, and vice versa.^15,80^ Examples of mixtures reported to be nonideal, and even synergistic or antagonistic, on the surface tension but ideal in CMC are mixtures of dodecyltrimethylammonium bromide (DTAB), and didodecyldimethylammonium bromide (DDAB) in water.^80,81^ Recently, it has been shown that mixtures of amphiphilic compounds (Sodium Dodecyl Sulfate or SDS, CetylTrimethyl Ammonium Chloride or CTAC, Triton X100/X114, Brij35) with oxalic and glutaric acid are nonideal in surface tension, even exhibiting some synergistic effects, but ideal in CMC.^68^ The nonideal effects on the surface tension were attributed to weakly repulsive effects (ionic or dipole–dipole) between the two types of molecules at the surface, thus reducing the surface tension. Synergistic effects on the CMC, such as observed in mixtures of different amphiphilic surfactants, are attributed to the formation of mixed micelles, i.e., including the two types of molecules.^15,82^ Antagonistic effects on the CMC are attributed to competition or steric hindrance between the two surfactants during the formation of the micelles.^15,82^

Nonideality, synergism, and antagonism are also likely to take place within the mixtures of amphiphilic surfactants present in atmospheric particles. However, to simplify the description of the surface tension of atmospheric aerosols, it might be easier to consider these amphiphilic mixtures as a single component, with net surface properties and isotherms resulting from all the interactions in the mixture.

#### 1.2.7 Surface Curvature: Tolman Length

**Figure 2:**
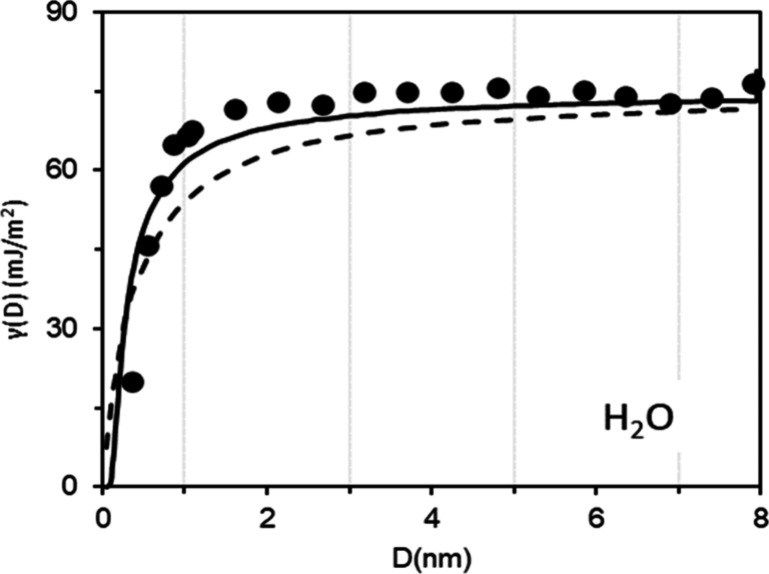
Variation of the surface tension of water (here, “γ”) as a function of the particle radius predicted by different models (dashed line = fit with Tolman’s equation; continuous line: modified Tolman model accounting for the size-dependent surface energy; dots: simulation of the system using thermodynamic perturbation theory). Adapted with permission from ref (87). Copyright 2005 American Chemical Society.

Because the surface tension results from the interactions between molecules at the surface of a liquid, it can be affected by the geometry of this surface. In small particles the curvature of the surface results in larger distances between the solvent molecules than in flat surfaces, thus weakening the interactions between the molecules^83^ and lowering the surface tension compared to flat surfaces. The impact of such geometry on the surface tension has been extensively studied since the 1950s^84−86^ and resulted in the definition of a characteristic radius, Tolman length, δ, for which the surface tension of a substance diverges significantly from that of a planar surface. For most substances the Tolman length is less than 1 nm:^84,87,88^ δ = 0.21^89^ and 0.53 nm^87^ for pure water (Figure 2), δ = 0 0.1 nm for deliquescent NaCl particles,^86^ and δ = 0.5–0.7 nm for organic compounds such as pentane and heptane.^88^ Most of these estimates are, however, obtained from theoretical models, as measuring this quantity experimentally is difficult and experimental values are scarce. In conclusion, particles made of pure substances or of homogeneous mixtures (i.e., having the same composition throughout the particle) have the same surface tension as flat surfaces of the same composition, down to radii as small as a few nanometers.

It is interesting to note that, in small particles, the “Tolman effect” on the surface tension and the well-known Kelvin effect^83^ on the vapor pressure (i.e., a larger vapor pressure of a compound above a curved surface than above a flat surface) are two sides of the same molecular phenomenon: the weakening of the bonds between the molecules on the curved surface. In the Kelvin effect, the weakening of the bonds allows more molecules to leave the surface for the gas, thereby increasing the vapor pressure. This effect becomes significant for larger radii (several 10 of nm) than the “Tolman effect” (≤1 nm) because the molecules occupy more space in the gas than in the condensed phase (or at the surface), thereby resulting in a stronger effect on the vapor pressure than on the surface tension. While the Kelvin effect is the main constraint in homogeneous nucleation processes, and largely taken into account to describe the nucleation of new aerosol particles and water droplets in the atmosphere, the role of surface tension in these processes has been much less taken into account (see Section 2).

#### 1.2.8 Surface/Volume Ratio: Bulk-to-Surface Partitioning

Because surfactants accumulate primarily at the surface of liquids rather than in the bulk, the existence of concentration gradients for these compounds inside small particles, referred to as bulk-to-surface partitioning, was proposed.^90^ The main implication is that, for a given ratio of total surfactant molecule number to sample volume (indicated as bulk concentration in adsorption isotherms), the surface tension of small particles would be larger than that of large-volume samples. As a discussion of surface tension models is beyond the scope of the present review, we refer to previous articles^2−4,8,91,92^ for more details on these theoretical discussions. Practically, bulk/surface partitioning implies that the surface tension values obtained from large-volume samples (>μL, corresponding to a particle radius of ∼1 mm), using classical techniques such as Wilhelmy plates, Du Noüy ring, or pendant drop (see Section 3) should underestimate the surface tension of microscopic particles of the same composition. As further discussed in Section 6, the occurrence and magnitude of these partitioning effects is still being debated, as very few experimental setups are able to investigate them and give somewhat contradictory results. For now, we underline in Section 3 that the measurements obtained from large-volume samples might need to be corrected for partitioning effects to be applied to micrometer or submicrometer particles.

#### 1.2.9 Temperature

**Figure 3:**
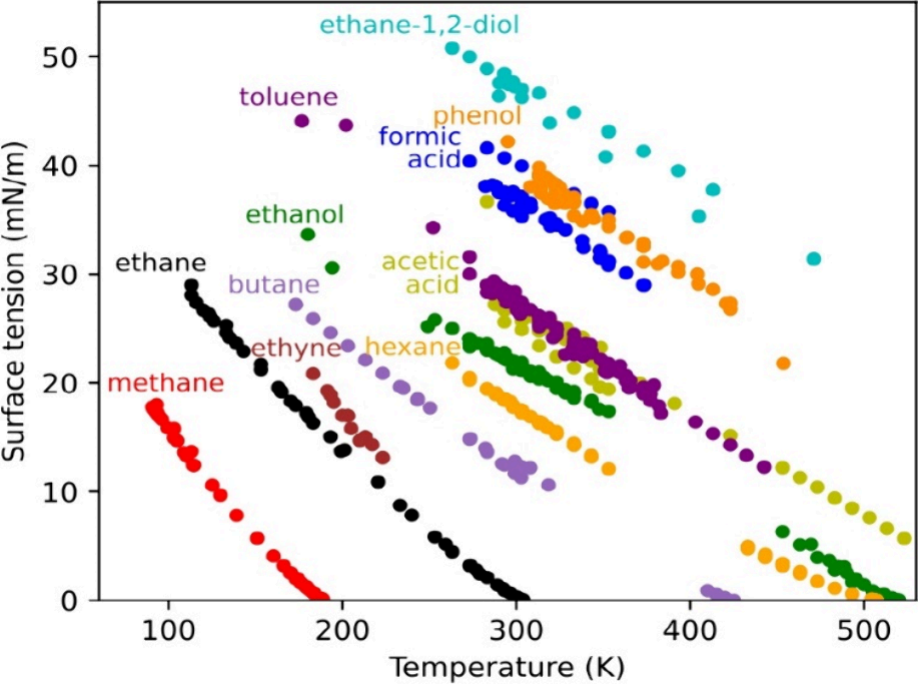
Variation of the surface tension with temperature for various organic compounds. Plotted from the surface tension data from ref (94). Reproduced with permission of SNCSC. Copyright 1997 Springer-Verlag.

As the temperature increases the motion of molecules, it weakens the interactions at the surface of liquids. Thus, high temperature reduces the surface tension, while low temperature increases it. The effects are, however, relatively small over the range of atmospheric temperature. The surface tension of water decreases by about 10% between 273 and 323 K.^93^ And while the surface tension of pure organic compounds varies by as much as 40% over the same range^94^ (Figure 3) it varies much less when they are present in aqueous solutions (<5% over 290–330 K).^95^ No significant effects of temperature was observed either on the CMC of SDS mixtures over 298–313 K.^96^

## 2 The Role of Surface Tension in Atmospheric Processes

**Figure 4:**
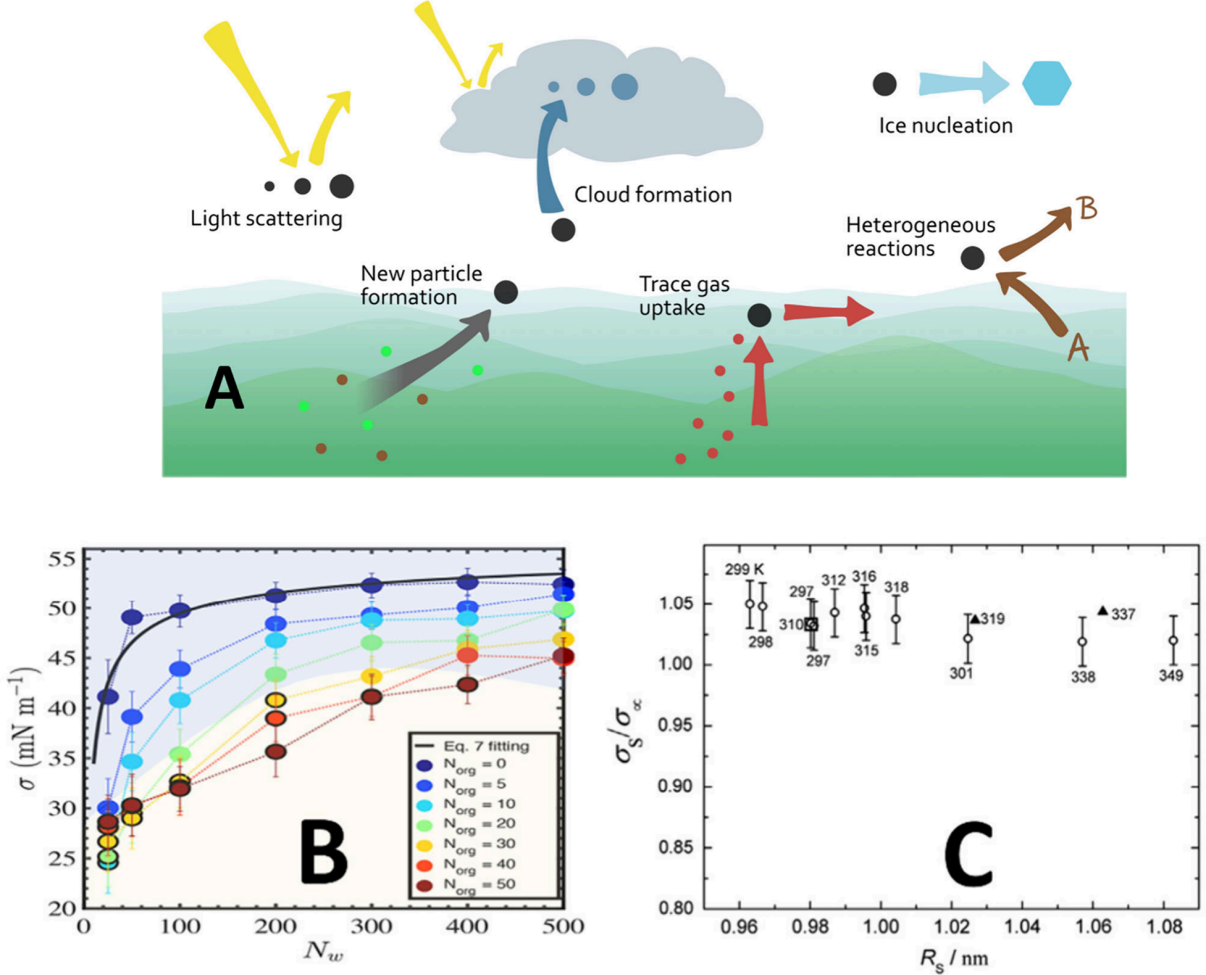
A) Overview of the atmospheric processes in which surface tension can potentially play a role; B) Variation of the surface tension (σ) of nanodroplets containing different amounts of organic molecules (represented by *N*_org_ = number of organic molecules) during the condensation of water (*N*_w_ = water molecule number). From ref (122). Copyright 2023 American Chemical Society. Licensed under CC-BY-NC-ND 4.0; C) Evolution of the surface tension of sulfur particles during their nucleation, normalized by that of the corresponding flat surface, σ_∞_, and determined experimentally from their nucleation rate at different temperatures. Reproduced with permission from ref (125). Copyright 2016 Elsevier Ltd.

Surface tension affects a number of phenomena and properties in gas/liquid systems, from their shape and size (spherical shape of droplets, formation or dampening of surface waves, etc.) and other capillary phenomena^97^ to the more complex Marangoni effects,^98^ resulting in common observations such as tears of wine and coffee stains.^99^ It also affects the transfer of mass and heat across the gas/liquid interface.^100−104^ Surface tension is thus expected to control many important properties and processes in atmospheric particles and droplets (Figure 4, top). However, only a few of them have been studied so far, which are discussed below. We emphasize again that the present review discusses exclusively the processes directly related to surface tension and not those involving organic coatings and surface films, for which we refer to previous reviews.^3,4^ The studies discussed below are thus either those in which surface tension was measured and a reduction evidenced or those that involve amphiphilic surfactants. It is also important to keep in mind that, besides actual atmospheric processes, surface tension is likely to be important in most of the techniques used for generating artificial particles in the laboratory. These techniques and their limits are directly relevant to the understanding of atmospheric processes as generating artificial particles in a controlled way, i.e., with a controlled composition and size distribution, is essential for constraining fundamental investigations.

### 2.1 Cloud Droplet Formation

The atmospheric process in which the role of surface tension has been the most investigated is by far the formation of cloud droplets. By potentially affecting the size distribution of cloud droplets, surface tension could affect the cloud optical and radiative properties (Figure 4A) and also the cloud lifetime in the atmosphere. Because the Kelvin effect precludes the homogeneous nucleation of liquid water in Earth’s atmosphere (see Section 2.3 below), liquid cloud droplets are formed exclusively by the condensation of water on pre-existing particles, called Cloud Condensation Nuclei (CCN).^105^ The founding work of Köhler^105,106^ resulted in an equation describing the equilibrium between the water vapor concentration in the gas (or saturation ratio, *S*) and the particle radius, *r*, resulting from the water uptake:
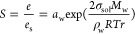
6where *e* is the water vapor pressure, *e*_s_ the saturation water vapor, and *M*_w_ and ρ_w_ are the molecular weight of water (18 g mol^–1^) and density of water (1 g cm^–3^), respectively. In eq 6 three parameters depend on the properties of the initial particle: its initial size (as an offset in the variable *r*), the water activity, *a*_w_, and the surface tension of the particle and forming droplet, σ_sol_. The curves corresponding to eq 6 display a maximum, defining a critical radius, *r*_crit_, and critical in saturation, *S*_crit_, below which (*S* < *S*_crit_) evaporation dominates and the droplets evaporate and above which (*S* > *S*_crit_) condensation dominates and the droplets grow. Lowering the surface tension reduces *S*_crit_ thus enhances either evaporation or condensation, depending on the conditions of relative humidity (i.e., the value of *S*). Since these early works, these processes have been the subject of thousands of articles, some of the most representative being refs (1, 8, 9, 107, and 108) and the reviews (1−4), to which we refer for more information. For instance, ref (108) alone is cited by more than 2200 articles, giving a scale for the number of studies focusing on CCN activation over the last 20 years. A small fraction of these studies has addressed specifically the role of surface tension.^8,9^ For a long time, the approaches to investigate the CCN properties of atmospheric aerosols were almost exclusively the measurement of CCN growth factors with Hygroscopic Tandem Differential Mobility Analyzers, HTDMAs or CCN numbers Cloud Condensation Nuclei Counters, CCNCs.^1,109^ It is, however, difficult to isolate the effects specifically due to surface tension from other effects (hygroscopcity, etc.) with these instruments. In laboratory, these effects have been distinguished, for instance, by exposing (NH_4_)_2_SO_4_ particles to organic vapors (methylglyoxal and acetaldehyde) and observing a reduction of the critical supersaturation based on CN/CCN measurements.^110^ However, only a few HTDMA-based observations of surface tension effects have been reported for atmospheric particles: measurements of CCN growth factors in the tropical Atlantic Ocean^111^ and Central Germany^112^ indicated that these particles might have a surface tension significantly lower than pure water (50–60 mN m^–1^).^111,112^ Recent HTDMA measurements of CCN growth factors in Southern China reported similar observations for newly formed particles (σ ∼ 60 mN m^–1^).^113^ To go around the lack of sensitivity of HTDMA and CCNC measurements to surface tension and the absence of direct surface tension measurements for atmospheric particles, different approaches have been developed over the last 15 years to estimate this parameter and its importance in cloud droplet formation. One approach consists of using in models the surface tension properties of surfactants extracted from atmospheric aerosols to estimate the surface tension of atmospheric particles and their contribution to cloud droplet formation. However, such multiple-step approaches result in large uncertainties. More global (or top-down) investigations of the role of surface tension in cloud droplet formation would be advantageous. To our knowledge, at the time of publication of this review, no direct evidence for the role of surface tension on cloud formation or properties has been reported yet. However, some potential future directions of investigation are proposed below in Section 2.5.

### 2.2 Nucleation of New Particles

Besides aqueous droplets, surface tension is expected to affect the nucleation of all types of materials. This parameter would affect both the condensation processes and the evaporation of the forming nanoparticles (the Kelvin effect). In the atmosphere, this applies to the homogeneous nucleation of sulfuric acid, amines and other organic compounds,^114−119^ processes reported to be potentially reversible in some observations.^120^ However, while surface tension in an explicit parameter in nucleation theory,^115,117^ its value is generally assumed to be constant and its importance for the formation of atmospheric particles has, to our knowledge, not been investigated experimentally. An experimental study of the nucleation of sub-3 nm sulfuric acid particles has shown that the classical condensation model fails to predict the observed growth, as well as its dependence on temperature and RH,^121^ indicating the involvement of other parameters, which could include the surface tension. Molecular dynamics simulations of the nucleation of organic/water particles,^122^ have also reported strong deviations of the surface tension from the continuous behavior assumed in usual theories, with surface tension of ∼20 to 40 mN m^–1^ predicted for sub-4 nm particles (Figure 4, bottom), thus underlining the importance of investigating this parameter experimentally.

To our knowledge, experimental investigations of the role of surface tension in nucleation processes were performed only in fields other than atmospheric science. They further confirm the importance of the surface tension in these processes. For instance, a study measuring the surface tension as a function of the surface curvature for nucleating colloidal particles of polyn-isopropylacrylamide (PNIPAM) in solutions of 3-methylpyridine (3MP) and heavy water showed that a reduction of the surface tension by 20% resulted in an increase of the nucleation rate by 3 orders of magnitude.^123^ In other studies, the surface tension of nucleating metal zinc and silver particles^124^ and of sulfur nanoparticles^125^ (Figure 4, bottom) from the gas phase and its variation with the particle radius (down to less than 1 nm) were determined from their nucleation rates. Given the strong impact of surface tension on nucleation rates, it could be interesting to study this parameter in atmospherically relevant nucleation processes. Some potential directions of investigation are proposed in Section 2.5.

### 2.3 Uptake of Gases and Heterogeneous Reactions

In addition to condensation/evaporation processes, surface tension is expected to affect the exchange of other compounds across the gas/liquid interface. For instance, adding relatively small surfactants (up to 10 C atoms) to solutions was shown to enhance the uptake of ammonia, NH_3_,^100,104^ and carbon dioxide, CO_2_.^103^ Inversely, adding long-chain surfactants (C > 10) to aqueous solutions was shown to oppose the uptake of NH_3_^100^ and O_2_,^101^ with a clear correlation between the surfactant chain length and mass-transfer reduction in the H_2_O/NH_3_ system.^100^ Thus, the surface tension of atmospheric particles is expected to affect the uptake and release of gases, which can, in turn, affect the removal of some gases from the atmosphere, the chemical composition of the particles, and possibly some reactions at the particle surface (heterogeneous reactions).

However, the effect of surface tension on the uptake of gases by atmospheric particles has been little studied. Most focus has been on the effects of organic coatings and surface films on the uptake of gases,^3,4^ which oppose the exchanges with the gas, but are not related to surface tension. A few exceptions could have been the studies involving amphiphilic surfactants such as Sodium Dodecyl Sulfate (SDS). However, they focused on the oxidation processes at the air/water interface, in particular on the reaction of SDS with an OH radical^126,127^ and its impact on the reaction rate of an organic compound (Tricarballylic acid, TCA) dissolved in the bulk.^127^ However, surface tension was not measured in these studies, and its potential effects on the gas uptake and surface reactivity were not investigated. Thus, to the best of our knowledge, the effects of surface tension on the uptake of gases by atmospheric particles and their heterogeneous chemistry still remain to be studied.

### 2.4 Ice Nucleation

Although, as indicated in Section 1, the Gibbs definition of surface tension might not apply to solids, a number of studies have addressed the potential role of surfactants on the nucleation of ice crystals in the atmosphere (see, for instance, the reviews^3,4^). This topic is thus briefly discussed here. In Earth’s atmosphere, the formation of ice crystals can occur either by homogeneous nucleation from liquid water at a temperature near or below −38 °C (235 K)^105^ or by heterogeneous nucleation on pre-existing solid particles, referred to as Ice Nucleating Particles (INPs) at much higher temperature, almost up to 0 °C. These heterogeneous processes can occur in different modes, referred to as immersion freezing, condensation freezing, and contact freezing. In immersion freezing the INP acts as CCN and the resulting water droplet freezes, in condensation freezing water vapor condenses on the INP to form first a liquid droplet, then freezes, and in contact freezing the INP collides with a water droplet, then freezes.^128^ While immersion freezing involves initially the same processes as described in Section 2.1 for liquid droplets formation, homogeneous nucleation and condensation freezing can be described by classical nucleation theory,^129^ in which surface tension is an explicit parameter. However, the key surface tension in these processes, and possibly also contact freezing, is that between the ice-nucleating surface (liquid water or INP) and the ice phase.^105^ Substances such as mineral dust, inorganic salts, soot,^130^ or biological particles,^131^ have been reported to act as INPs, i.e., to increase the threshold temperature at which ice crystals are formed. In particular, experimental studies such as refs (132−139) have even evidenced the INP efficiency of monolayers of amphiphilic organic compounds. However, these substances do not affect the interfacial tension between the ice-nucleating surface and the ice phase. Their INP efficiency is due to their highly ordered molecular structures, providing a 2D-lattice pattern favoring the nucleation of hexagonal ice. To our knowledge, no experimental data are available on the interfacial tension between the ice-nucleating surface and the ice phase, as this parameter is very challenging to measure. Such experimental data would be interesting to have. However, unlike for liquid surfaces, this interfacial tension is not expected to vary significantly during the ice nucleation processes in the atmosphere or to be affected by the presence of surfactants and, thus, to be a limiting factor in the nucleation processes.

### 2.5 Perspectives on the Investigations of Surface Tension in Various Atmospheric Processes

As discussed in this section, the role of surface tension in atmospheric processes has mostly been investigated for cloud droplet formation. However, even in this case, no clear evidence of a role of surface tension on cloud formation has been established, and there is a need to develop new directions of investigation to overcome the limits of the current techniques. An interesting approach could be, for instance, to develop methods to achieve the selective sampling of CCN and interstitial aerosols or of cloudwater and interstitial aerosols. Such a selection could perhaps be performed based on growth factors at the output of a HTDMA, collecting separately the particles with GF = 1 (interstitial particles) and those with GF > 1 (CCN). Provided that enough material can be accumulated, the surface tension could be measured for both populations and compared and indicate whether the CCN or cloudwater contain more surfactants than the interstitial aerosols. Even more global investigation of a role of surface tension on cloud formation or properties could consist, for instance, in evidencing correlations or causality relationships^140^ between long-term series of cloud properties (frequency, lifetime, droplet size distribution, etc.) at a given site and corresponding series for the surface tension of the aerosols or CCN upwind from the site.

As discussed in Section 2.2, although evidenced in other fields, the role of surface tension in the nucleation of materials other than water remains entirely to be studied for atmospheric particles. This could be investigated in the laboratory. For instance, for organic particles by comparing the nucleation rates of different organic materials with different surface tensions. For more complex mixtures, such as sulfuric acid/organic, the surface tension could be estimated from the vaporization enthalpies.^141,142^

The role of surface tension in the uptake and release of gases, while also evidenced in other fields of research, remains also largely to be investigated from the point of view of atmospheric particles. This could also be done in the laboratory, where the role of common organic aerosol components (for instance, organic acids) or amphiphilic surfactants in the uptake and release of gases could be studied, the same way as many other uptake processes have been studied in the atmospheric literature.

As in the case of cloud droplet formation, a main reason for not exploring the role of surface tension in these other processes was, for a long time, the lack of data on the surface tension of atmospheric particles and widespread belief that it was identical with that of pure water. However, the progress made over the last 15 years shows that this is not the case, and the new approaches for measuring the surface tension now allow us to explore its role in these other processes.

## 3 Surface Tension Measurement Techniques for Atmospherically Relevant Mixtures and Particles

Some of the most spectacular developments in the investigation of the surface tension of atmospheric particles and relevant mixtures over the last 10 years were those of measurement techniques. While surface tension had mostly been studied with techniques requiring large volume samples in other fields of chemistry and chemical physics, within a decade the atmospheric community has developed approaches to determine the surface tension of μL-atmospheric samples and individual pL (pico-L) particles. This section presents the techniques currently available to determine the surface tension of atmospherically relevant mixtures and particles. This includes the classical techniques requiring large volume samples (≥μL), which are still largely used for the investigation of model mixtures in the laboratory, those requiring mL- to μL-volume samples, that have been applied to atmospheric fogwater, cloudwater and aerosol sample extracts (see Section 5), and the techniques applicable to micrometer or submicrometer individual particles. The main reason for developing techniques applicable to individual micrometer-sized particles is to eventually measure the surface tension of individual atmospheric particles. In addition, as discussed in Section 1.2.8, such techniques are the only ones allowing to investigate bulk-to-surface partitioning and other effects specific to microscopic particles. It is thus important to keep in mind that the results of the “bulk techniques” presented below (and the isotherms presented in Section 4) might require some bulk-to-surface partitioning corrections to be applicable to microscopic particles.

Note that as underlined in Section 1, the techniques presented in this section measure the static surface tension, except perhaps for the optical traps and optical tweezers techniques described in Section 3.2.1. Many of the techniques described below have already been presented elsewhere,^5^ in particular those for individual particles,^6,143−146^ and we refer to these previous articles for more detail. The descriptions below are kept short to focus on the main features, advantages, or limitations regarding atmospheric samples. Each of the techniques described below is assigned an abbreviation, referring to the literature data presented in Section 4 and in Tables 1−5.

**Table 1:** List of the Binary Mixtures of Organic Acids in Water Included in This Inventory

n.	Common name (IUPAC Name)	Brut formula	CAS n.	MW (g mol^–1^)	*σ_pur_ (mN m^–1^)	**ρ (g cm^–3^)^248^	***Atmos. ?	Method
AC1	formic acid (methanoic acid)	CH_2_O_2_	64-18-6	46.03	37.2,^15^ 38.17,^249^ 37.03,^250^ 35.81–40.71^94^	1.220	Y^192,194,196^	DVT,^249^ WP^250^
AC2	acetic acid (ethanoic acid)	C_2_H_4_O_2_	64-19-7	60.05	29.4,^10^ 27.08,^249^ 27.12,^250^ 25.61–27.8^94^	1.045	Y^196^	DVT,^249^ WP^250^
AC3	propionic acid (propanoic acid)	C_3_H_6_O_2_	79-09-4	74.08	26.17,^250^ 26.2,^20,248^ 26.15,^249^ 25.13–27^94^	0.988		DVT,^249^ WP^250^
AC4	butyric acid (butanoic acid)	C_4_H_8_O_2_	107-92-6	88.11	26.05,^248^ 26.19,^249^ 26.21,^251^ 25.31–26.83^94^	0.953		DVT,^249,251^ CR^151^
AC5	oxalic acid (ethanedioic acid)	C_2_H_2_O_4_	144-62-7	90.03		1.900	Y^192−198^	PD,^47,68,252,253^ WP^48,149,150,254^
AC6	methanesulfonic acid	CH_4_O_3_S	75-75-2	96.11	53^255^	1.481	Y^196^	WP,^255,256^ NR^148^
AC7	valeric acid (pentanoic acid)	C_5_H_10_O_2_	109-52-4	102.13	26.7,^15^ 26.63^251^	0.934		DVT,^251^ NR^257^
AC8	malonic acid (propanedioic acid)	C_3_H_4_O_4_	141-82-2	104.06		1.63^a^	Y^192,196,197^	PD,^47,252,253^ WP,^149,150^ AFM^183^
AC9	β-hydroxybutyric acid (3-hydroxybutanoic acid)	C_4_H_8_O_3_	300-85-6	104.11		1.13^b^		WP^52^
AC10	maleic acid ((2Z)-but-2-enedioic acid)	C_4_H_4_O_4_	110-16-7	116.07		1.590	Y^192,197,198^	PD,^47,253^ WP^150,254^
AC11	caproic acid (hexanoic acid)	C_6_H_12_O_2_	142-62-1	116.16	27.51,^251^ 27.2–28.1^94^	0.921		DVT,^251^ NR,^257^ AFM,^186^ NP^186^ CR^62^
AC12	succinic acid (butanedioic acid)	C_4_H_6_O_4_	110-15-6	118.10		1.572	Y^192−199^	PD,^46,47,252,253,258,259^ WP,^48,149,150,254^ CR^65^
AC13	benzoic acid (benzenecarboxylic acid)	C_7_H_6_O_2_	65-85-0	122.12		1.266		DW^260^
AC14	cyclohexylmethanoic aci (cyclohexanecarboxylic acid)	C_7_H_12_O_2_	98-89-5	128.17		1.033		DVT^261^
AC15	enanthic acid (heptanoic acid)	C_7_H_14_O_2_	111-14-8	130.19	27.8,^15^ 30.07,^257^ 28.14–28.7^94^	0.912		NR,^257^ WP^262^
AC16	glutaric acid (pentanedioic acid)	C_5_H_8_O_4_	110-94-1	132.11		1.429	Y^193,194,196−198^	PD,^47,68,252,253,259^ WP,^48,149,174,263^ AFM,^183^ OT^174,264^
AC17	malic acid (2-hydroxybutanedioic acid)	C_4_H_6_O_5_	6915-15-7	134.09		1.601	Y^192,194,197−199^	PD,^47,253^ WP^150^
AC18	p-toluic acid (4-methylbenzoic acid)	C_8_H_8_O_2_	99-94-5	136.15		1.06^c^		DW^260^
AC19	3-hydroxybenzoic acid	C_7_H_6_O_3_	99-06-9	138.12	71.3^52^	1.485		WP^52^
AC20	cyclohexylethanoic acid	C_8_H_14_O_2_	5292-21-7	142.20		1.042		DVT^261^
AC21	caprylic acid (octanoic acid)	C_8_H_16_O_2_	124-07-2	144.21	28.2–29.2^94^	0.907		NR^257^
AC22	adipic acid (hexanedioic acid)	C_6_H_10_O_4_	124-04-9	146.14		1.360	Y^193,197,198^	PD,^47,252,253^ WP^48,265^
AC23	4-ethylbenzoic acid	C_9_H_10_O_2_	619-64-7	150.17		1.1^p^		DW^260^
AC24	cyclohexylpropanoic acid	C_9_H_16_O_2_	701-97-3	156.22		0.912		DVT^261^
AC25	pelargonic acid (nonanoic acid)	C_9_H_18_O_2_	112-05-0	158.24	26.2–29.7^94^	0.905		NR,^257^ WP,^266^ PB^267^
AC26	4-propylbenzoic acid	C_10_H_12_O_2_	2438-05-03	164.20		1.1^p^		DW^260^
AC27	phtalic acid (benzene-1,2-dicarboxylic acid)	C_8_H_6_O_4_	88-99-3	166.13		1.59^d^	Y^192,197−199^	WP^48^
AC28	cyclohexylbutanoic acid	C_10_H_18_O_2_	4441-63-8	170.25		1.0^p^		DVT^261^
AC29	capric acid (decanoic acid)	C_10_H_20_O_2_	334-48-5	172.27		0.9^p^		NR,^257^ PB^268^
AC30	4-butylbenzoic acid	C_11_H_14_O_2_	20651-71-2	178.23		1.1^p^		DW^260^
AC31	pinonic acid (3-acetyl-2,2-dimethylcyclobutylacetic acid)	C_10_H_16_O_3_	473-72-3	184.23		1.1^p^	Y^193^	DVT,^61^ PD,^47,253^ WP,^52,150^ NR^63^
AC32	undecylic acid (undecanoic acid)	C_11_H_22_O_2_	112-37-8	186.29		0.891		NR^257^
AC33	azealic acid (nonanedioic acid)	C_9_H_16_O_4_	123-99-9	188.22		1.225	Y^193,197−199^	WP^48,52^
AC34	citric acid (2-hydroxypropane-1,2,3-tricarboxylic acid)	C_6_H_8_O_7_	77-92-9	192.12		1.665		PD,^47,253,269^ WP^254,263^
AC35	4-pentylbenzoic acid	C_12_H_16_O_2_	26311-45-5	192.25		1.0^p^		DW^260^
AC36	lauric acid (dodecanoic acid)	C_12_H_24_O	143-07-7	200.32		0.9^p^		WP^270^
AC37	trimesic acid (benzene-1,3,5-tricarboxylic acid)	C_9_H_6_O_6_	554-95-0	210.14		1.7^p^		WP^48^
AC38	oleic acid ((9Z)-octadec-9-enoic acid)	C_18_H_34_O_2_	112-80-1	282.47	32.79,^271^ 31.8,^272^ 30.99–32.8^94^	0.894		NR,^273,274^ PD,^275^ WP^276^
AC39	ricinoleic acid ((9Z,12R)-12-hydroxyoctadec-9-enoic acid)	C_18_H_34_O_3_	141-22-0	298.46		0.945		NR^277^
AC40	arachidonic acid ((5Z,8Z,11Z,14Z)-icosa-5,8,11,14-tetraenoic acid)	C_20_H_32_O_2_	506-32-1	304.5		0.908		NR^278^
AC41	7,10-dihydroxy-8(E)-octadecenoic acid	C_18_H_34_O_4_	131021-99-3	314.5		1.0^p^		NR^277^

* Data measured at 20–25 °C.

** Data measured at 15–25 °C.

*** Reported in atmospheric aerosols; WP = Whilhelmy plate; PD = pending droplet (shape of a droplet); DVT = drop volume tensiometry, NR = Nouy ring; CR = capillary rise; DW = drop weight; PB = pending Bubble; AFM = atomic force microscopy, NP: Du Noüy-Padday method, OT = optical tweezer.

a ThermoFischer Scientific, Safety Data sheet according to Regulation UK SI 2019/758 and UK SI 2020/1577, Malonic acid, A11526, 2024 Revision 6.

b Sigma-Aldrich, Safety Data Sheet according to Regulation (EC) No. 1907/2006, 3-Hydroxybutyric acid, 166898, 2023 Version 6.4.

c Sigma-Aldrich, Safety Data Sheet according to Regulation (EC) No. 1907/2006, 4-Methylbenzoic acid for synthesis, 2023 Version 6.12.

d Sigma-Aldrich, Safety Data Sheet, 4-Phthalic acid for synthesis, 2023 Version 9.0.

p Predicted data from ChemSpider (RSC) generated using the ACD/Laboratories Percepta Platform - PhysChem Module version 14.00.

**Table 2:** List of the Binary Mixtures of Aldehydes, Ketones, and Alcohols in Water Included in This Inventory

n.	common name (IUPAC Name)	Brut formula	CAS n.	MW (g mol^–1^)	*σ_pur_ (mN m^–1^)	**ρ (g cm^–3^)^248^	***Atmos. ?	Method
AK1	formaldehyde (methanal)	CH_2_O	50-00-0	30.03		1.09^*sa*^		PD^279^
AK2	methanol	CH_3_OH	67-56-1	32.04	22.51,^280^ 24,^281^ 22.14,^282^ 25,^283^ 22.7,^284^ 21.8–22.95^94^	0.791		WP,^280,281^ DVT,^282^ BP,^283^ NR^284^
AK3	acetaldehyde (ethanal)	C_2_H_4_O	75-07-0	44.05	20.6–21.2^94^	0.783		PD^279^
AK4	ethanol	C_2_H_5_OH	64-17-5	46.07	22.0,^285^ 21.82,^280^ 22,^281^ 21.72,^282^ 22.07–22.85,^286^ 22.6,^284^ 21.3–23.32^94^	0.789		CR,^285^ WP,^280,281^ DVT,^282^ DN,^286^ NR^284^
AK5	acetone (propan-2-one)	C_3_H_6_O	67-64-1	58.08	23.1,^287^ 23.02,^288^ 24.5,^283^ 21.62–24.02^94^	0.785		CR,^289^ BP,^283,287^ PD,^288^ NR^290^
AK6	propan-1-ol	C_3_H_7_OH	71-23-8	60.1	23.28,^280^ 26,^291^ 23.1,^292^ 23.32,^282^ 23.5,^284^ 23.1–23.9^94^	0.800		WP,^280^ BP,^291^ CR,^151,292^ DVT,^282^ NR,^284^ CR^62^
AK7	propan-2-ol	C_3_H_7_OH	67-63-0	60.1	21.22,^280^ 23.5,^283^ 20.34–21.74^94^	0.781		WP,^280^ BP^283^
AK8	ethylene glycol (ethane-1,2-diol)	C_2_H_6_O_2_	107-21-1	62.06	46.24,^293^ 47,^291^ 47.6–48.49^94^	1.114		CR,^293^ BP^291^
AK9	propylene glycol (propane-1,2-diol)	C_3_H_8_O_2_	57-55-6	76.09	35.46,^293^ 36.6,^294^ 35.6,^292^ 35.8–36.6^94^	1.036		CR,^292,293^ BP^294^
AK10	propane-1,3-diol	C_3_H_8_O_2_	504-63-2	76.09	45.58,^292^ 46.95,^293^ 45.62–49.2^94^	1.054		CR^292^
AK11	pentan-1-ol	C_5_H_11_OH	71-41-0	88.15	24.8–25.6^94^	0.814		DVT^295^
AK12	1,3-butanediol	C_4_H_10_O_2_	107-88-0	90.12	37.04^293^	1.005		CR^293^
AK13	1,4-butanediol	C_4_H_10_O_2_	110-63-4	90.12	43.79,^293^ 44.6–47.4^94^	1.017		CR^293^
AK14	glycerol (propane-1,2,3-triol)	C_3_H_8_O_3_	56-81-5	92.09	63.4,^296^ 62.5,^285^ 63.0,^292^ 59.5,^189^ 62.9,^189^ 59.4–63.7^94^	1.261		CR,^285,292^ WP,^189,296^ AFM^189^
AK15	phenol (benzenol)	C_6_H_6_O	108-95-2	94.11	39.59–42.2^94^	1.07^a^		DW^260^
AK16	hexan-1-ol	C_6_H_13_OH	111-27-3	102.18	24.08–26.55^94^	0.814		DVT,^295^ WP^297^
AK17	hexan-2-ol	C_6_H_13_OH	626-93-7	102.18	24.25–24.7^94^	0.818^b^		WP^297^
AK18	2,3-dimethylbutan-2-ol	C_6_H_13_OH	594-60-5	102.18	23.74–23.74^94^	0.824		WP^297^
AK19	2-methylpentan-2-ol	C_6_H_13_OH	590-36-3	102.18	22.58–22.9^94^	0.835		WP^297^
AK20	1,5-pentanediol	C_5_H_12_O_2_	111-29-5	104.15	44.16,^298^ 43.3^94^	0.991		WP^298^
AK21	p-cresol (4-methylbenzenol)	C_7_H_8_O	106-44-5	108.13		1.034^c^		DW^260^
AK22	heptan-1-ol	C_7_H_15_OH	111-70-6	116.2	25.7–27.25^94^	0.822		WP,^262^ DVT^295^
AK23	hexane-1,2-diol	C_6_H_14_O_2_	6920-22-5	118.17	23.8^299^	0.951^d^		CR^299^
AK24	hexane-1,6-diol	C_6_H_14_O_2_	629-11-8	118.17		0.96^e^		CR^299^
AK25	hexane-1,5-diol	C_6_H_14_O_2_	928-40-5	118.17	33.9^299^	0.971		CR^299^
AK26	hexane-2,5-diol	C_6_H_14_O_2_	2935-44-6	118.17	31.6^299^	0.961		CR^299^
AK27	4-ethylphenol	C_8_H_10_O	123-07-9	122.16		1.01^f^		DW^260^
AK28	octan-1-ol	C_8_H_17_OH	111-87-5	130.2	25.56–27.9^94^	0.826		NR,^300^ DVT,^295^ WP^297^
AK29	octan-2-ol	C_8_H_17_OH	123-96-6	130.2	25.5–26.7^94^	0.819^g^		WP^297^
AK30	4-propylphenol	C_9_H_12_O	645-56-7	136.19		1.009		DW^260^
AK31	1-naphthol (naphthalen-1-ol)	C_10_H_8_O	90-15-3	144.17		1.28^h^		NR^301^
AK32	2-naphthol (naphthalen-2-ol)	C_10_H_8_O	135-19-3	144.17		1.28		NR^301^
AK33	nonan-1-ol	C_9_H_19_OH	143-08-8	144.25	27–28.3^94^	0.828		WP^297^
AK34	nonan-5-ol	C_9_H_19_OH	623-93-8	144.25		0.822		WP^297^
AK35	4-*tert*-butylphenol	C_10_H_14_O	98-54-4	150.22		0.908^i^		DW^260^
AK36	4-s-butylphenol	C_10_H_14_O	99-71-8	150.22		0.986		DW^260^
AK37	2,3-dihydroxynaphthalene	C_10_H_8_O_2_	92-44-4	160.17		1.3^p^		NR^301^

* Data measured at 20–25 °C.

** Data measured at 15–25 °C.

*** Reported in atmospheric aerosols; WP = Whilhelmy plate; PD = pending droplet (shape of a droplet); DVT = drop volume tensiometry, NR = Nouy ring; DN = drop number; DW = drop weight; BP = bubble pressure; CR = capillary rise; ^sa^37 wt.% in H_2_O, Sigma-Aldrich (pure: 0.815 at 253.15 K^248^).

a Roth, Safety Data Sheet acc. to Regulation (EC) No. 1907/2006 (REACH), Phenol ≥99%, Ph.Eur., crystalline, 3215, 2020, Version 5.0.

b Sigma-Aldrich, Safety Data Sheet according to Regulation (EC) No. 1907/2006, 2-Hexanol, 128570, 2023, Version 6.3.

c Sigma-Aldrich, Safety Data Sheet according to Regulation (EC) No. 1907/2006, p-Cresol, C85751, 2023, Version 7.2.

d Sigma-Aldrich, Safety Data Sheet according to Regulation (EC) No. 1907/2006, 1,2-Hexanediol, 213691, 2022, Version 6.7.

e Sigma-Aldrich, Safety Data Sheet according to Regulation (EC) No. 1907/2006, 1,6-Hexanediol for synthesis, 804308, 2023, Version 6.8.

f Sigma-Aldrich, Safety Data Sheet according to Regulation (EC) No. 1907/2006, 4-Ethylphenol, E44205, 2023, Version 8.6.

g Sigma-Aldrich, Safety Data Sheet according to Regulation (EC) No. 1907/2006, 2-Octanol, O4504, 2023, Version 6.6.

h Sigma-Aldrich, Safety Data Sheet according to Regulation (EC) No. 1907/2006, 1-Naphthol for synthesis, 822289, 2023, Version 6.15.

i Sigma-Aldrich, Safety Data Sheet according to Regulation (EC) No. 1907/2006, 4-tert-Butylphenol, B99901, 2024, Version 6.11.

p Predicted data from ChemSpider (RSC) generated using the ACD/Laboratories Percepta Platform - PhysChem Module version 14.00.

**Table 3:** List of the Binary Mixtures of Sugars and Amines in Water Included in This Inventory

n.	Common name (IUPAC name)	Brut formula	CAS n.	MW (g mol^–1^)	*σ_pure_ (mN m^–1^)	**ρ (g cm^–3^)^248^	***Atmos. ?	Method
SA1	colamine (2-aminoethan-1-ol)	C_2_H_7_NO	141-43-5	61.08	48.95,^302^ 48.10,^303^ 48.30,^304^ 48.3–49.24^94^	1.018	Y^196^	WP,^302^ PD^303^
SA2	pyrrolidine (prolamine)	C_4_H_9_N	123-75-1	71.12	29.75,^305^ 29.65,^304^ 29.23–29.65^94^	0.859		WP^305^
SA3	glycine (aminoacetic acid)	C_2_H_5_NO_2_	56-40-6	75.07		1.161	Y^196^	PD,^306^ DVT^307^
SA4	threamine (1-aminopropan-2-ol)	C_3_H_9_NO	78-96-6	75.11	37.38	0.973^a^		PD^308^
SA5	3-aminopropan-1-ol	C_3_H_9_NO	156-87-6	75.11	43.90,^308^ 44.7^304^	0.982		PD^308^
SA6	2-(methylamino)ethan-1-ol	C_3_H_9_NO	109-83-1	75.11	35.28^309^	0.937		WP^309,310^
SA7	piperidine (pyridine)	C_5_H_11_N	110-89-4	85.15	29.56,^305^ 29.48^304^	0.861		WP^305^
SA8	*N*,*N*′-dimethylethane-1,2-diamine	C_4_H_12_N_2_	110-70-3	88.15	26.4^311^	0.828		WP^311^
SA9	dl-alanine (2-aminopropanoic acid)	C_3_H_7_NO_2_	302-72-7 (dl-alanine) 56-41-7 (l-alanine)	89.09		1.432, 1.424	Y^196^	PD,^306^ DVT^307^
SA10	β-alanine (3-aminopropanoic acid)	C_3_H_7_NO_2_	107-95-9	89.09		1.437	Y^196^	DVT^312^
SA11	2-(ethylamino)ethan-1-ol	C_4_H_11_NO	110-73-6	89.14	32.21^309^	0.914		WP^309^
SA12	2-amino-2-methylpropan-1-ol	C_4_H_11_NO	124-68-5	89.14	31.37^302^	0.934		WP^302^
SA13	2-(dimethylamino)ethan-1-ol	C_4_H_11_NO	108-01-0	89.14	31.5^313^	0.887		CR^313^
SA14	cyclohexanamine	C_6_H_13_N	108-91-8	99.17	32.4,^314^ 31.81,^304^ 31.51–31.54^94^	0.819		NR^314^
SA15	piperidic acid (4-aminobutanoic acid)	C_4_H_9_NO_2_	56-12-2	103.12		1.1^p^		DVT^312^
SA16	dl-2-aminobutanoic acid	C_4_H_9_NO_2_	2835-81-6	103.12		1.230		DVT^307^
SA17	1-dimethylaminopropan-2-ol	C_5_H_13_NO	108-16-7	103.16	24.0^315^	0.837		WP^315^
SA18	diolamine (2,2′-azanediyldi(ethan-1-ol))	C_4_H_11_NO_2_	111-42-2	105.14	47.21^316^	1.097		WP,^316,317^ PD^318^
SA19	5-aminopentanoic acid	C_5_H_11_NO_2_	660-88-8	117.15		1.1^p^		DVT^312^
SA20	dl-norvaline (2-aminopentanoic acid)	C_5_H_11_NO_2_	760-78-1	117.15		1.1^p^		DVT^307^
SA21	methyl diethanolamine (2,2′-(methylazanediyl)di(ethan-1-ol))	C_5_H_13_NO_2_	105-59-9	119.16	38.90,^319^ 38.3,^313^ 37.29,^320^ 39.8^94^	1.043		WP,^317,319^ CR,^313^ PD^318,320^
SA22	2-amino-2-ethyl-1,3-propanediol	C_5_H_13_NO_2_	115-70-8	119.16		1.099		CR^321^
SA23	2-amino-2-hydroxymethyl-propane-1,3-diol	C_4_H_11_NO_3_	77-86-1	121.14		1.32^b^		PD^322^
SA24	erythritol (2R,3S)-butane-1,2,3,4-tetrol	C_4_H_10_O_4_	149-32-6	122.12		1.451	Y^192^	DVT^323^
SA25	6-aminohexanoic acid	C_6_H_13_NO_2_	60-32-2	131.17		1.0^p^		DVT^312^
SA26	dl-norleucine (2-aminohexanoic acid)	C_6_H_13_NO_2_	616-06-8	131.17		1.172		DVT^307^
SA27	l-leucine ((S)-2-amino-4-methylpentanoic acid)	C_6_H_13_NO_2_	61-90-5	131.17		1.293	Y^196^	WP,^324^ DVT^325^
SA28	hexamethylenetetramine (1,3,5,7-tetraazaadamantane)	C_6_H_12_N_4_	100-97-0	140.18		1.331^c^		WP^326^
SA29	trolamine (2,2′,2″-nitrilotri(ethan-1-ol))	C_6_H_15_NO_3_	102-71-6	149.19	45.95,^316^ 45.95–48^94^	1.124		WP^316^
SA30	xylitol (meso-xylitol)	C_5_H_12_O_5_	87-99-0	152.15		1.52^d^		DVT^323^
SA31	levoglucosan ((1R,2S,3S,4R,5R)-6,8- dioxabicyclo[3.2.1]octane-2,3,4-triol)	C_6_H_10_O_5_	498-07-7	162.14		1.6^46^	Y^192,193,199^	WP,^48,52^ PD^46,47^
SA32	l-phenylalanine	C_9_H_11_NO_2_	63-91-2	165.19		1.2^p^	Y^196^	PD^306^
SA33	d-(+)-glucose ((2R,3S,4R,5R)-2,3,4,5,6-pentahydroxyhexanal)	C_6_H_12_O_6_	50-99-7	180.16		1.54^43^		WP,^48,327^ DVT^323^
SA34	d-(+)-galactose ((2R,3S,4S,5R)-2,3,4,5,6-pentahydroxyhexanal)	C_6_H_12_O_6_	59-23-4	180.16		1.5^e^		WP^48^
SA35	inositol (1,2,3,4,5,6-hexahydroxycyclohexane)	C_6_H_12_O_6_	87-89-8	180.16		1.752^f^		DVT^323^
SA36	sorbitol ((2S,3R,4R,5R)-hexane-1,2,3,4,5,6-hexol)	C_6_H_14_O_6_	50-70-4	182.17		1.489	Y^192^	DVT^323^
SA37	d-(+)-maltose ((3R,4R,5S,6R)-6-(hydroxymethyl)-5-[[(2R,3R,4S,5S,6R)-3,4,5-trihydroxy-6-(hydroxymethyl)oxan-2-yl]oxy]oxane-2,3,4-triol)	C_12_H_22_O_11_	69-79-4	342.3		1.8^p^		WP^48^
SA38	sucrose (β-d-fructofuranosyl α-d-glucopyranoside)	C_12_H_22_O_11_	57-50-1	342.3		1.581	Y^192^	WP^48^

* Data measured at 20–25 °C.

** Data measured at 15–25 °C.

*** Reported in atmospheric aerosols; WP = Whilhelmy plate; PD = pending droplet (shape of a droplet); DVT = drop volume tensiometry, NR = Nouy ring; CR = capillary rise.

a Sigma-Aldrich, Safety Data Sheet according to Regulation (EC) No. 1907/2006, 1-Amino-2-propanol, 110248, 2023, Version 7.4.

b Supelco, Safety Data Sheet according to Regulation (EC) No. 1907/2006, Tris(hydroxymethyl)aminomethane 77-86-1, 102408, 2023, Version 6.6.

c Sigma-Aldrich, Safety Data Sheet according to Regulation (EC) No. 1907/2006, Hexamethylenetetramine, 398160, 2023, Version 6.1.

d Sigma-Aldrich, Safety Data Sheet according to Regulation (EC) No. 1907/2006, Xylitol, X3375, 2023, Version 6.5.

e Roth, Safety Data Sheet acc. to Regulation (EC) No. 1907/2006 (REACH), d(+)-Galactose ≥98%, 4987, 2020, Version GHS 3.0.

f Sigma-Aldrich, Safety Data Sheet according to Regulation (EC) No. 1907/2006, myo-Inositol, I5125, 2023, Version 6.7.

p Predicted data from ChemSpider (RSC) generated using the ACD/Laboratories Percepta Platform - PhysChem Module version 14.00.

**Table 4:** List of the Binary Mixtures of Synthetic and Biological Surfactants in Water Included in This Inventory

n.	Type	Common name (IUPAC Name)	Brut formula	CAS n.	MW (g mol^–1^)	*σ_min_ (mN m^–1^)	**ρ (g/cm^3^)	***Atmos. ?	Method
SB1	anionic surfactant	SDS (sodium dodecyl sulfate)	NaC_12_H_25_SO_4_	151-21-3	288.38	39.1,^328^ 38.3,^329^ 39.6,^330^ 37.6,^331^ 34.06^68^	0.998^330^^a^		NR,^329^ WP,^328,331^ DN,^330^ PD^68^
SB2	cationic surfactant	DTAB (dodecyltrimethylammonium bromide)	C_15_H_34_BrN	1119-94-4	308.34	38.7,^332^ 40.0,^333^ 37.0,^334^ 37.7^331^	0.998^335^^b^		WP,^331,332^ DN,^334^ PD^333^
SB3	cationic surfactant	CTAB (cetyltrimethylammonium bromide)	C_19_H_42_BrN	57-09-0	364.45	36.5,^336^ 36.3,^337^ 37.5,^329^ 41.6,^331^ 36.6,^60^	0.9964^338^^c^		DW,^336^ PB,^337^ NR,^60,329^ WP^331^
SB4	anionic surfactant	AOT (sodium bis(2-ethylhexyl)sulfosuccinate)	C_20_H_36_Na_2_O_7_S	577-11-7	444.56	29.5,^339^ 27.3,^340^ 32.5,^341^ 29.2,^342^ 26.1^343^	1.140^344^		DVT,^339^ WP,^340,343^ NR,^341,342^
SB5	nonionic surfactant	Triton X114 ((1,1,3,3-tetramethylbutyl)phenyl-polyethylene glycol)	(C_2_H_4_O)*_n_*C_14_H_22_O, *n* = 7 or 8	9036-19-5	∼537	29.1,^345^ 31.1,^346^ 28.4,^347^ 30.8,^329^ 29.0^348^	1.058^d^		WP,^345^ NR,^329,346,347^ PD^348^
SB6	nonionic surfactant	Brij35 (polyoxyethylene lauryl ether)	(C_2_H_4_O)_*n*_C_12_H_26_O, *n* = 23	9002-92-0	1198.57	44.0,^349^ 43.6,^350^ 43.5,^342^ 42.7^351^ 44.91^68^	1.05^e^		NR,^342,349^ WP,^350,351^ PD^68^
SB7	biological surfactant	monorhamnolipid	C_26_H_48_O_9_	37134-61-5	504.3	25.3,^352^ 30,^69^ 27.9^353^	1.06^69^ 0.998211^353^		NR^69,352,353^
SB8	Biological surfactant	dirhamnolipid	C_32_H_58_O_13_	4348-76-9	650.4	28.8,^352^ 32.0,^69^ 27.9^353^	1.06^69^ 0.998211^353^		NR^69,352,353^
SB9	biological surfactant	surfactin from *Bacillus subtilis*	C_53_H_93_N_7_O_13_	24730-31-2	1036.3	31.9,^354^ 31.1,^355^ 29.0,^161^ 26.6^356^	1^p^		NR,^354^ DVT,^355^ PD^161^ WP^356^
SB10	biological surfactant	syringafactin B/C from *Xanthomonas* and *Pseudomonas*	C_55_H_10_1O_13_N_9_		1094.75	25.0^245^			PD^245^
SB11	biological surfactant	viscosin from *Pseudomonas*	C_54_H_95_N_9_O_16_	27127-62-4	1125.69	30.6,^357^ 27.6,^358^ ∼25^245^	1.2^p^		NR,^357,358^ PD^245^

* Data measured at 20–25 °C.

** Data measured at 15–25 °C.

*** Reported in atmospheric aerosols; WP = Whilhelmy plate; PD = pending droplet (shape of a droplet); DW = drop weight; DVT = drop volume tensiometry, NR = Nouy ring; DN = drop number; PB = Pending bubble.

a Density of an aqueous solution of SDS at 0.014 M [25 °C].^330^

b Density of an aqueous solution of DTAB at 0.005 M [25 °C].^335^

c Density of an aqueous solution of CTAB at 0.001 M [25 °C].^338^

d Sigma-Aldrich, Safety Data Sheet according to Regulation (EC) No. 1907/2006, Triton X-114, X114, 2023, Version 6.11.

e Sigma-Aldrich, Safety Data Sheet according to Regulation (EC) No. 1907/2006, Brij 35 for synthesis, 801962, 2023, Version 6.10.

p Predicted data from ChemSpider (RSC) generated using the ACD/Laboratories Percepta Platform - PhysChem Module version 14.00.

**Table 5:** List of the Binary Mixtures of Macromolecules in Water Included in This Inventory

n.	Type	Common name (IUPAC Name)	MW (g mol^–1^)	*σ_min_ (mN m^–1^)	**ρ (g/cm^3^)	***Atmos. ?	Method
MA1	macromolecules	SRFA (Suwannee river fulvic acid)	570^43^	64.6,^41^ 52.0,^46^ 38.2,^44^ 51.9,^47^ 44.7^48^	1.5^43^		PD,^41,44,46,47^ WP^48^
MA2	macromolecules	NAFA (Nordic aquatic fulvic acid)	4266^359^	63.4,^41^ 55.2,^49^ 52.5^50^	1.5^b^		PD,^49,50^ NR^41^
MA3	macromolecules	humic acid (commercial)	226.14^a^	48.1,^51^ 52.5–65.8,^48^ 58.7,^52^ 57.5^360^	1.5^b^		WP,^48,52^ NR^51,360^
MA4	macromolecules	HULIS (humic like substances)	∼507 (410–610)^44^	49.6,^42^ ∼48.1 (41.8–53.0),^41^ 41.4–42.9^44^	1.6^43^	Y	NR,^41^ PD^42,44^
MA5	macromolecules	EPS (extracellular polymeric substances)	∼158000 (62.4–213.1 kDa)^361^	52,^53^ 66.6,^54^ 59.6–61.5^55^			NR^53−55^

* Data measured at 20–25 °C.

** Data measured at 15–25 °C.

*** Reported in atmospheric aerosols; WP = Whilhelmy plate; PD = pending droplet, NR = Nouy ring.

a Commercial Humic acid sodium salt C_9_H_8_Na_2_O_4_ 68131-04-4 Thermo Scientific Chemicals.

b Extrapolated from the surface tension of SRFA.^43^

### 3.1 Techniques Applicable to Bulk Samples (>1 μL)

#### 3.1.1 Force Measurement (WP, NR, DNP)

**Figure 5:**
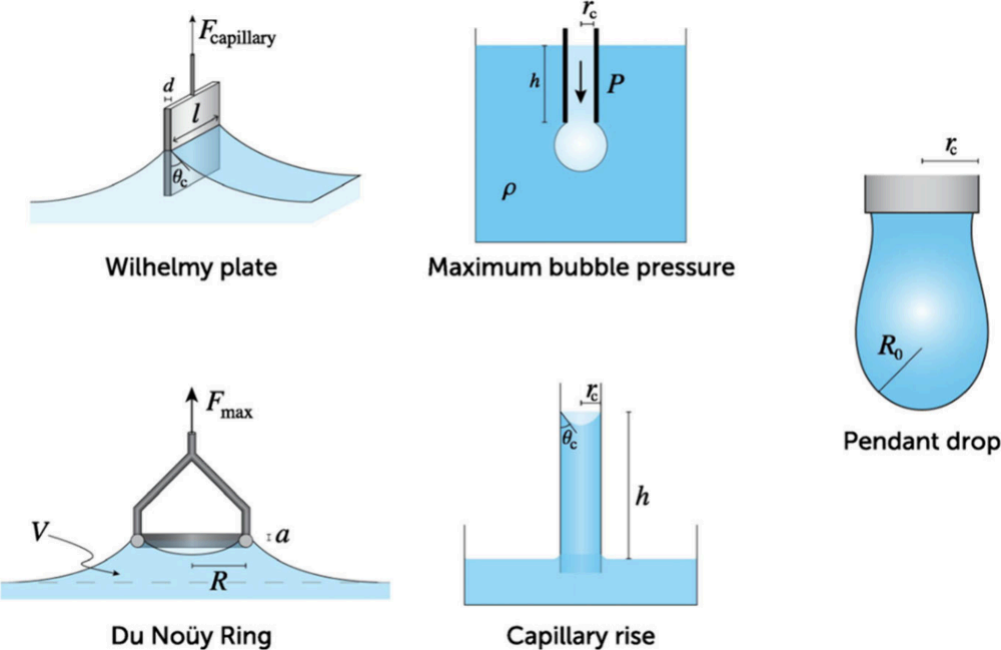
Schematics of various experimental techniques used to determine the surface tension. Adapted with permission from ref (169). Crown copyright 2015 Elsevier Ltd.

Some of the oldest and most common techniques to measure the surface tension of liquids, which are also those requiring the largest sample volume (>mL), are based on dropping a metal object, a plate (Wilhelmy Plate, WP), a ring (Du Noüy Ring, NR) (Figure 5), or a thin rod (Du Noüy-Padday, DNP) in the liquid of interest and measuring the force, *F*, necessary to pull it up, usually with an electrobalance:
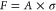
7where *A* is the surface area of the object in contact with the liquid. Thus, these techniques depend on the surface area of the object in contact with liquid *A*, and might require some correction factors to account for limited wettability. The uncertainties on the measurements with these techniques have been reported to be of the order of 0.1 mN m^–1^.^147^ However, the required sample volumes are at least in the mL range. These techniques have been used to measure the surface tension of a large number of binary aqueous mixtures of reference compounds of atmospheric relevance (organic acids, etc.) in the laboratory (see Section 4 and refs (48, 148−150)).

#### 3.1.2 Gravitational Equilibrium (CR, PD, PB, DVT, DW, DN, BP)

A vast class of techniques for the measurement of the surface tension of liquids is based on equilibrating the surface tension forces, *F*, applied to a sample with gravity, i.e., with the sample weight. They include the Capillary Rise (CR), Pendant Droplet (PD, also referred to as “Pending droplet” or “Hanging droplet”), Pendant Bubble (PB), Drop Volume Tensiometry (DVT), Drop Weight (DW), Drop Number (DN), and Bubble Pressure (BP) techniques. In these techniques, the surface tension is determined from the Young–Laplace equation, expressing the capillary pressure, Δ*P*, across an interface of area *A*, thus corresponding to a force, *F*, resulting from the surface tension, σ:
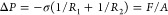
8where *R*_1_ and *R*_2_ are the two main radii of curvature, in the case of a nonspherical surface. Equilibrating the force *F* with the sample weight thus gives
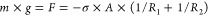
9where *m* is the mass of the sample and *g* the gravitational constant.

There are many variations on this approach. The one requiring the most volume samples (≥mL) is the Capillary Rise technique (CR, Figure 5), which consists in dipping a capillary tube into the liquid of interest and measuring the height of the liquid, h, in the capillary tube. In this approach, the adhesion forces result in the formation of a meniscus at the interface, so that both *R*_1_ and *R*_2_ can be replaced by *R* in eq 9 and *A*/*R* = π *r* cos θ, where *r* is the radius of the liquid column and θ is the contact angle between the liquid and the capillary (often neglected). Expressing *m* as a function of the liquid density, ρ, and volume of the liquid column (*h* × π *r*^2^) in eq 9 thus allows σ to be determined. This technique has the advantage to convert small forces into a significant height, *h*, thus lowering the uncertainties compared with direct force measurements such as WP and NR. CR has thus mostly been used for the investigation of artificial mixtures of atmospheric relevance, such as in ref (151).

Other variants of this approach consist in forming a small droplet of the liquid of interest at the tip of a needle or a capillary tube and equilibrating it against its own weight. A very common method is the Pendant Droplet (PD, Figure 5), in which the shape of the droplet is measured with a camera to determine the radii *R*_1_ and *R*_2_. Comparing the droplet shape with its weight using the Young–Laplace equation (eq 9) provides the value of σ.^152^ The Drop Volume Tensiometry (DVT), Drop Weight (DW), and Drop Number (DN)^153^ approaches are based on the same principle than PD, the droplet being formed at the extremity of a capillary and equilibrated with the surface tension force by eq 9 at the time of its detachment. However, instead of measuring the shape of the droplet, the DW and DN techniques consist of measuring the combined weight or volume resulting from several droplets, thus avoiding the need of a camera. The DVT technique is similar to PD, as the volume of the droplet is measured with a camera just before its detachment, but is usually employed for droplets formed inside another liquid. Other approaches that are also based on eq 9 consist of forming a small gas bubble inside of the liquid of interest and measuring either its shape with a camera (Pendant Bubble, PB) or determining its pressure from its curvature (Bubble Pressure, BP, Figure 5).

Several of these gravitational techniques, in particular PD, DW, and DVT, require relatively small volume samples (<100 mL) and, thus, are applicable to atmospheric samples. The techniques based on the formation of bubbles in a liquid, such as DW and PB, require at least several tens of milliliters of samples and thus can be applied to fog or cloudwater samples. DW has thus been used to measure the surface tension of atmospheric fogwater^154^ and PB for fog and cloudwater.^155,156^ The PD is the technique requiring the least sample volume (<μL) and also limits the contacts between the sample and laboratory vessels (thus potential contamination) and thus are most applicable to atmospheric aerosol samples, as illustrated by several studies.^157−168^

### 3.2 Techniques Applicable to Individual Particles (<μL)

#### 3.2.1 Airborne Particles: Electrodynamic Balance, Optical Tweezers, and Optical Traps (OTs)

**Figure 6:**
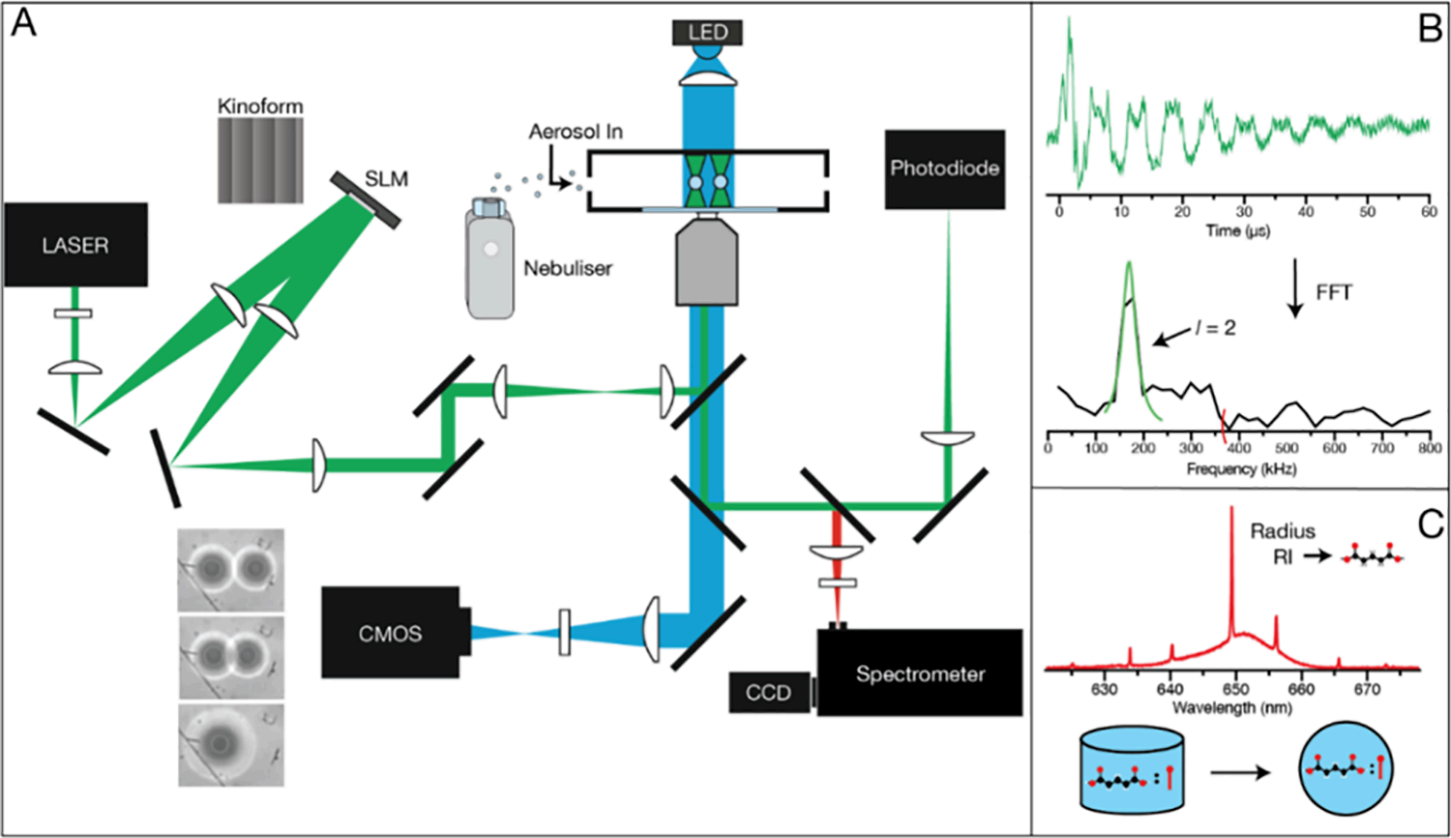
Illustration of an optical tweezer/optical trap: A) Two optical traps are generated using a kinoform on a spatial light modulator (SLM). Once the droplets are confined to the traps, they are moved together to coalesce; B) Elastically scattered light of the event collected with a photodiode and a fast-Fourier transform (FFT) providing the oscillation frequency of the surface modes from which the surface tension is determined; C) Cavity-enhanced Raman spectrum collected with the spectrograph yielding, after analysis of the spectrum with Mie Theory, the radius and refractive index of the droplet resulting from coalescence. The concentration of the cosolute is determined from the refractive index. Reproduced with permission from ref (180). Copyright 2023 American Chemical Society. Licensed under CC-BY 4.0.

Techniques to isolate individual particles with a diameter between 10 and 200 μm in air or microfluidic systems and determine their surface tension have been developed as early as the 1990s.^170−172^ Airborne particles can be isolated in a small domain by combing oscillating and static electric fields in electrodynamic balance,^168^ or by levitating them on a focused laser beam or stabilizing them between two crossing beams in optical traps and optical tweezers (Figure 6).^24,173−181^ In these techniques, the surface tension of the particles is usually determined by inducing first a deformation of the particle, then by monitoring the resulting oscillations from the backscattered light or Quasi-Elastic-Laser Scattering (QELS),^168,181,182^ from which the surface tension is obtained. Initial deformation and surface oscillations have thus been generated by applying a pulsed electric field,^172^ coalescing two particles inside the optical trap,^174,175,178,180,181^ or by applying thermal fluctuations.^182^ These techniques have the advantage of avoiding contacts between the particles and any surface in the instruments and of being noninvasive, thus avoiding contaminations. The individual particles can be stabilized long enough to vary the RH and study complete adsorption isotherms. The uncertainties reported on the surface tension measurements with these techniques are reported to be of the order of ±1 mN m^–1^,^174^ thus substantially larger than with the large-volume techniques for bulk samples because of the inherent difficulties in studying micrometer-size particles. Potential drawbacks of these techniques are that, because of the induced oscillations, they might be measuring a dynamic σ rather than a static one and the analysis performed to determine the surface tension must involve some assumptions on the dynamic behavior of the particle material (density, viscosity, compressibility, etc.). However, by measuring the surface tension of particles in the 5–10 μm size range, these techniques are among the only ones allowing investigating some fundamental aspects, such as the importance of bulk-to-surface partitioning on the surface tension. These techniques also allow one to study other properties of the particles.^177^ For instance, they could be used to study the role of σ in condensation/evaporation, in nucleation processes, or in the exchange of compounds between the particles and air discussed in Section 2. They are also the most promising techniques to determine another essential parameter related to surface tension: the concentration of the surfactant in individual particles (see Section 6). This information is key in many investigations of the surface tension of particles but particularly challenging to measure. This could potentially be done by adding specific dyes to the particles, that would complex with the surfactant molecules, and measuring their signatures with optical spectroscopies.

#### 3.2.2 Deposited Particles: Nanotensiometry, Atomic Force Microscopy (AFM)

**Figure 7:**
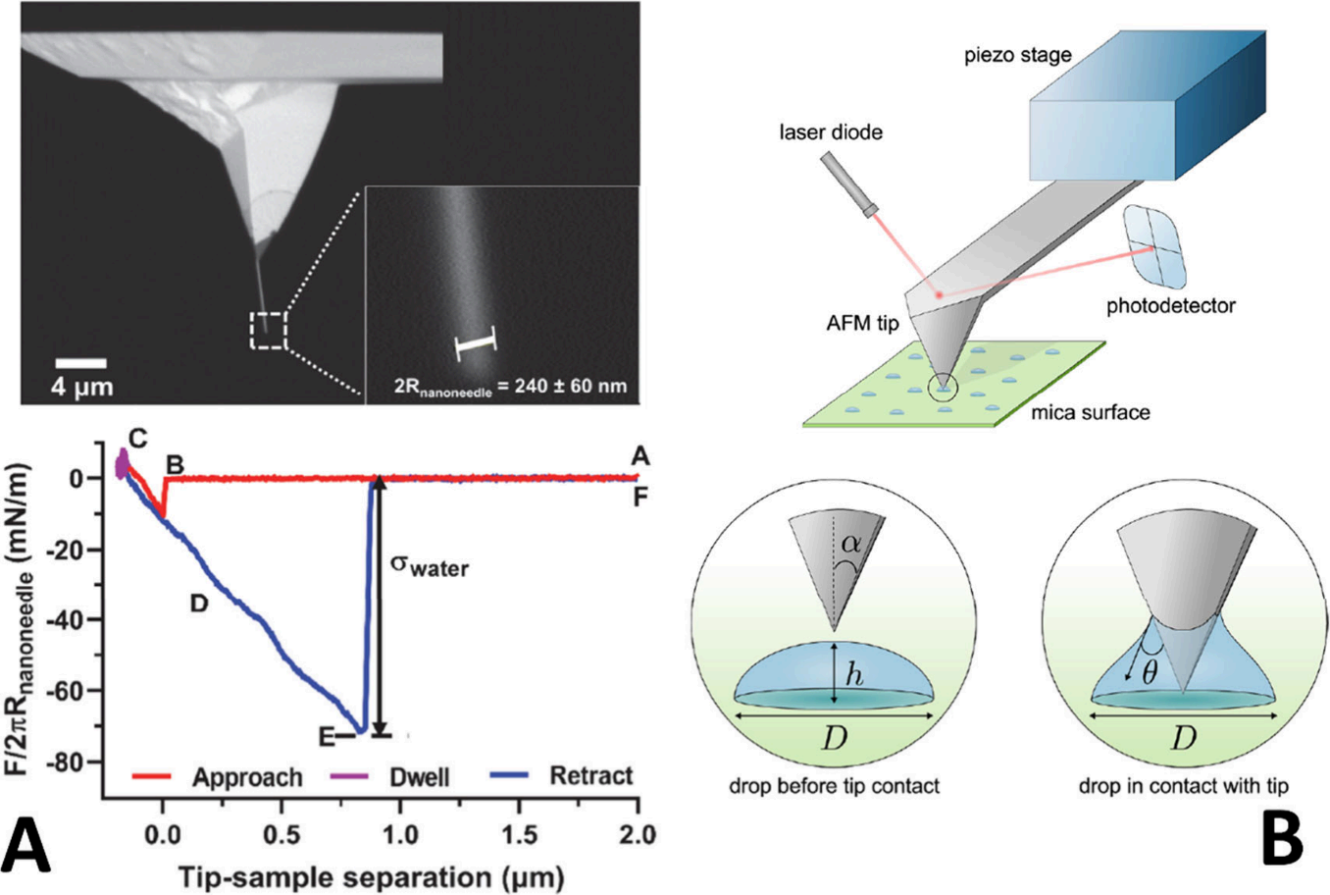
Illustration of surface tension measurements by AFM. A) Top: zoom on the nanoneedle used to probe the surface; bottom: example of force curves obtained from the measurements, from which the surface tension is determined. Reproduced with permission from ref (186). Copyright 2021 American Chemical Society. B) Illustration of the interactions between the AFM tip and microdroplets deposited on a surface. Reproduced with permission from ref (189). Copyright 2023 American Chemical Society. Licensed under CC-BY 4.0.

Another family of techniques allowing the measurements of the surface tension of individual particles are those based on nanotensiometry, i.e., the same principle as the Wilhelmy plate (WP) and Du Noüy ring (NR) techniques, but at microscopic scale: to drop the tip (diameter < a few μm) of an Atomic Force Microscopy (AFM, Figure 7)) instrument or a nanoneedle (typically several 100 of nm in diameter),^183^ in the sample of interest and measure the force necessary to retrieve it. Atomic Force Microscopy, which is used routinely to study submicrometer details on surfaces, has also been used to measure the surface tension of small surfaces.^144^ Over the last 10 years it has been used to measure the surface tension of submicrometer atmospherically relevant particles,^183−188^ as summarized in ref (6). The uncertainties on the surface tension values obtained with such techniques are reported to be on the order of ±0.5 mN m^–1^. These techniques are the only ones able to investigate particles well below the micrometer-size. It has also the advantages of being a direct determination of σ, i.e., with few intermediate steps and corrections, and to measure a static σ rather than a dynamic one. Its main drawback, however, is that the droplets are not airborne but necessarily deposited on a surface (substrate), thus increasing the risks of contamination of the sample. Potential further development and applications of these different techniques are discussed in Section 6.

## 4 Adsorption Isotherms for Organic/Water Mixtures of Atmospheric Relevance

### 4.1 Scope of the Inventory

The increase of interest for the surface tension of atmospheric particles over the last 15 years has led to the development of a number of models trying to predict it.^7−14^ However, the experimental data on the surface tension of atmospherically relevant organic compounds in water, which are needed to validate such models or develop relationships between molecular structure and surface tension, are widely dispersed in the literature. To facilitate these works, this section presents a unique inventory of experimental adsorption isotherms reported in the literature for more than 130 aqueous mixtures of organic compounds. Complete lists of the organic compounds included in this inventory are given in Tables 1, 2, 3, 4, and 5: organic acids (Table 1), alcohols, aldehydes, and ketones (Table 2), amines, amino acids, and sugars, (Table 3), amphiphilic compounds in Table 4, and macromolecular compounds (Table 5). Most of these data was measured at 20–25 °C, unless indicated otherwise. The uncertainties reported on these measurements are generally less than ±1% or ±0.2 mN m^–1^. The complete isotherm data are provided in Sections S2 and S3 of the Supporting Information, reporting all the data sets found in the literature for each compound, those originally published being shown in bold. All the isotherms are presented as a function of both concentration, *C* (M), and molar fraction, *x*, of the compound, and the conversion made between these units are presented in Section S1. Whenever available in the literature, other units, such as mass fraction, are also provided in the SI.

The corresponding adsorption isotherm curves are presented in Figures 8−17, where symbols represent experimental data points reported in the literature (when only one data set was available for the mixture). When several data sets were available, curves interpolating at best the data sets were calculated (see next paragraph), which are represented by smooth lines in the Figures.

**Figure 8:**
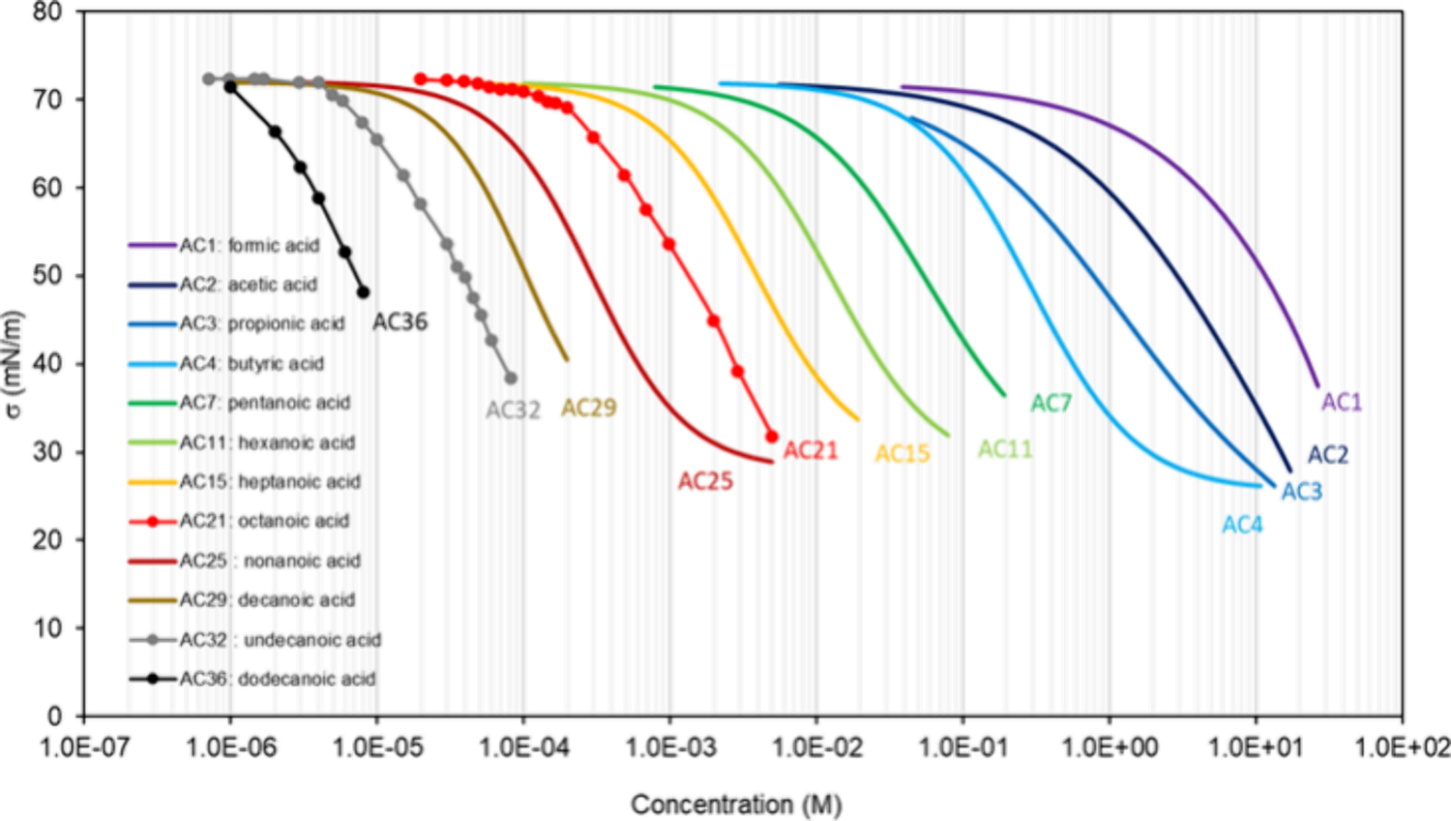
Adsorption isotherms for binary mixtures in water of linear organic monoacids. The curves represented by solid lines are recommended values based on multiple data sets (see SI and text), while those with symbols represent single experimental data sets.

**Figure 9:**
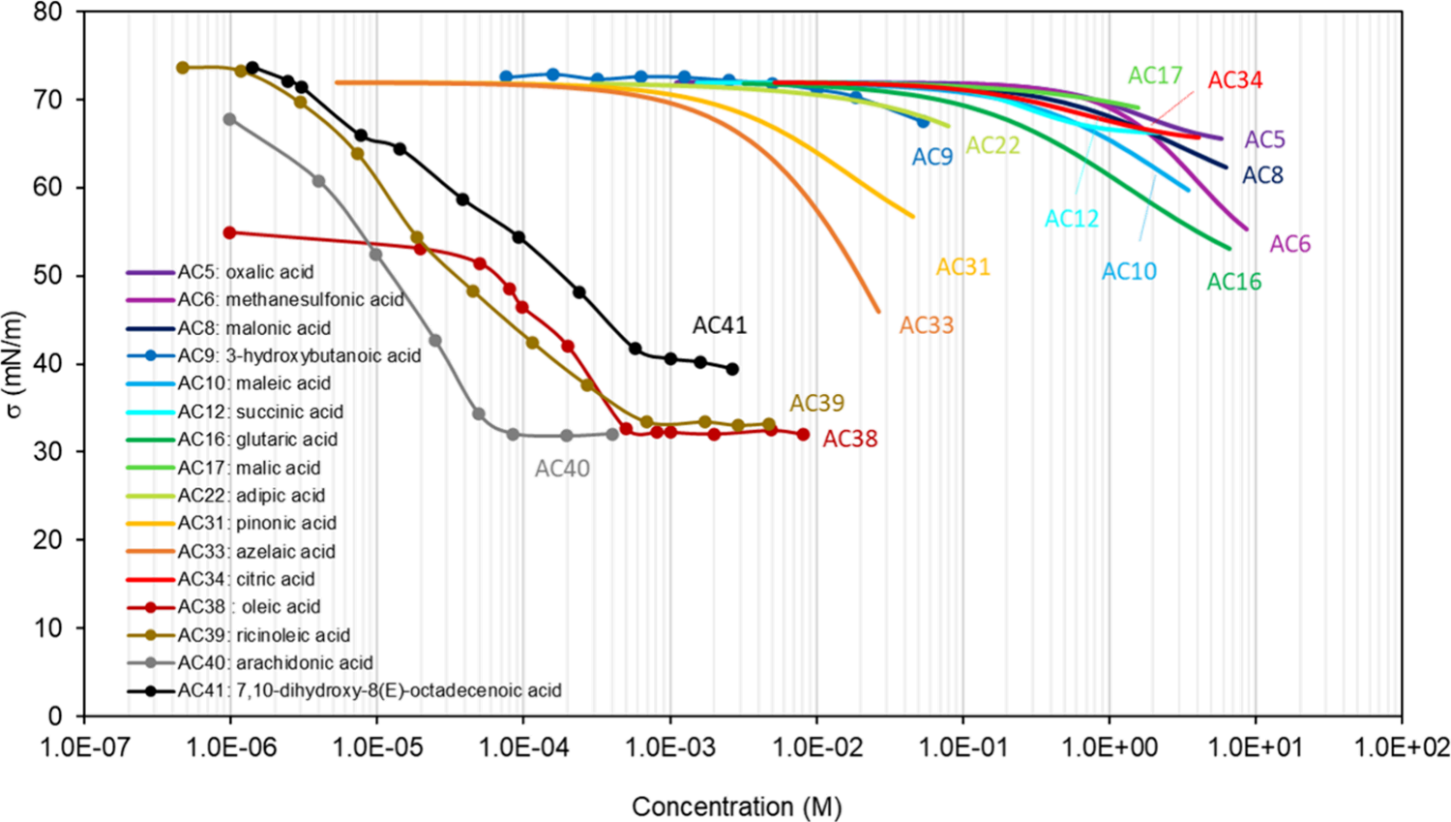
Adsorption isotherms for binary mixtures in water of organic diacids, triacids, unsaturated acids, and other substituted acids. The curves represented by solid lines are recommended values based on multiple data sets (see SI and text), while those with symbols represent single experimental data sets.

**Figure 10:**
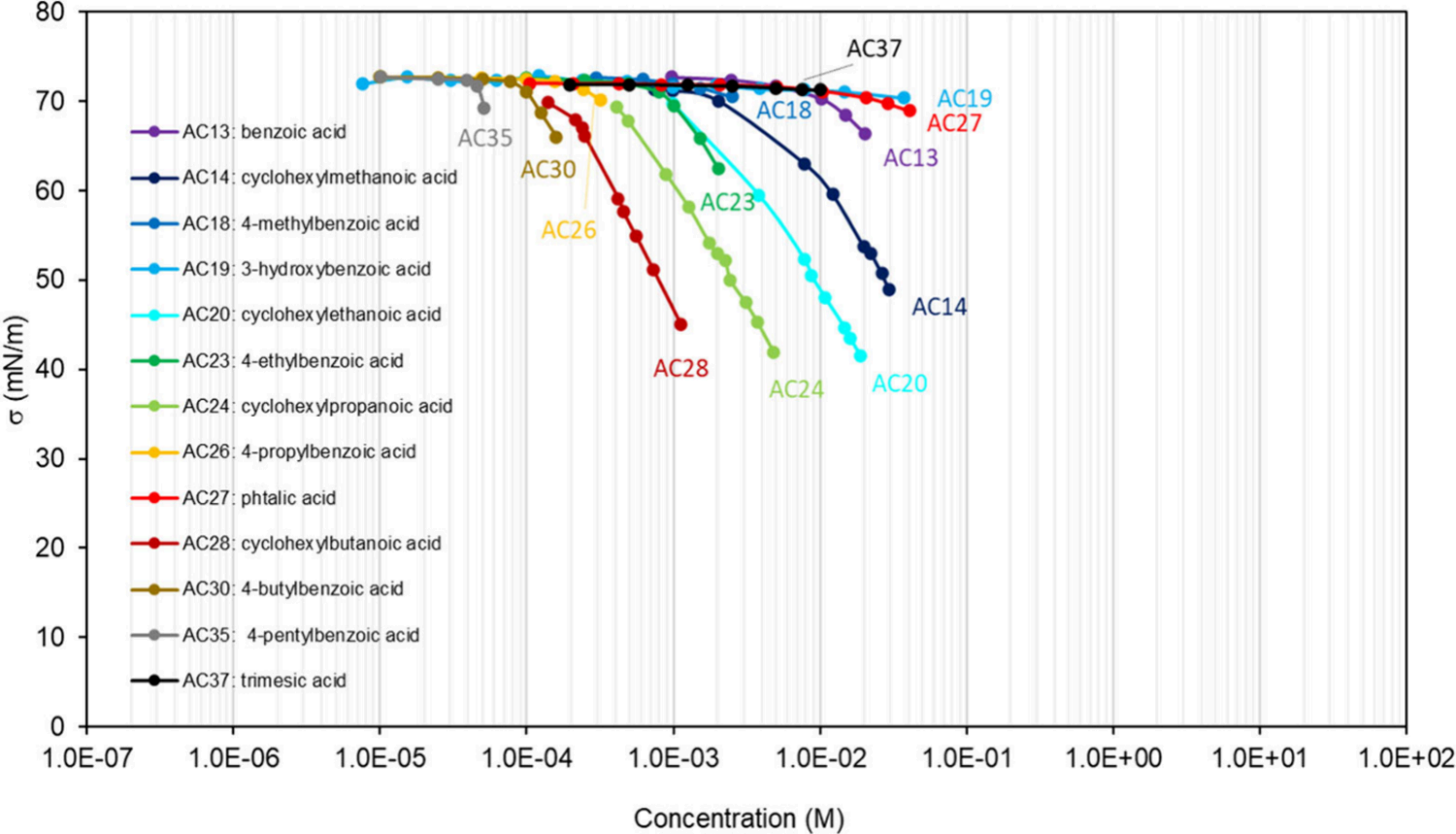
Adsorption isotherms for aromatic and cyclic acids. The curves represented by solid lines are recommended values based on multiple data sets (see SI and text), while those with symbols represent single experimental data sets.

**Figure 11:**
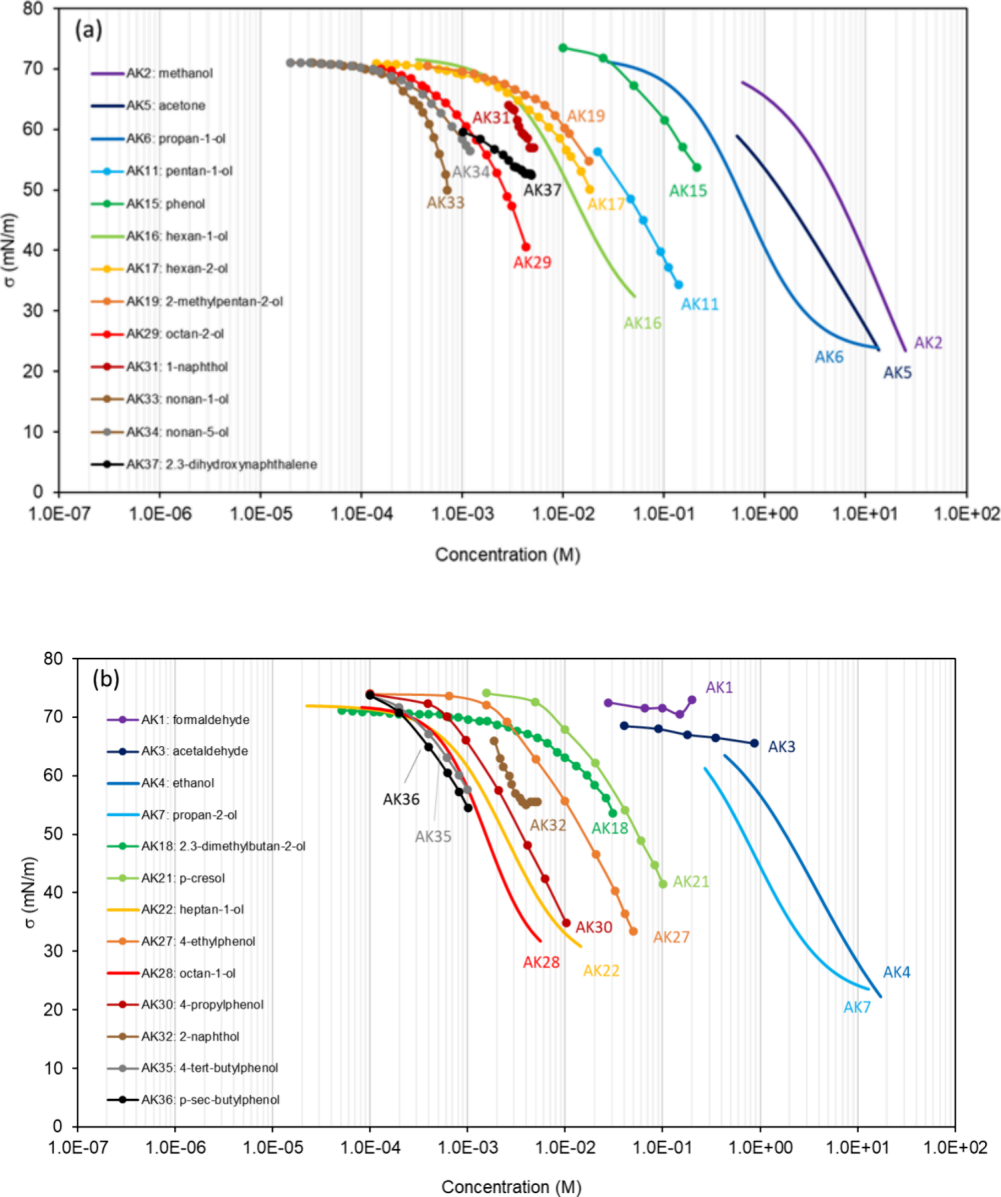
Adsorption isotherms for binary mixtures in water of aldehydes, ketones, and monoalcohols. The curves represented by solid lines are recommended values based on multiple data sets (see SI and text), while those with symbols represent single experimental data sets.

**Figure 12:**
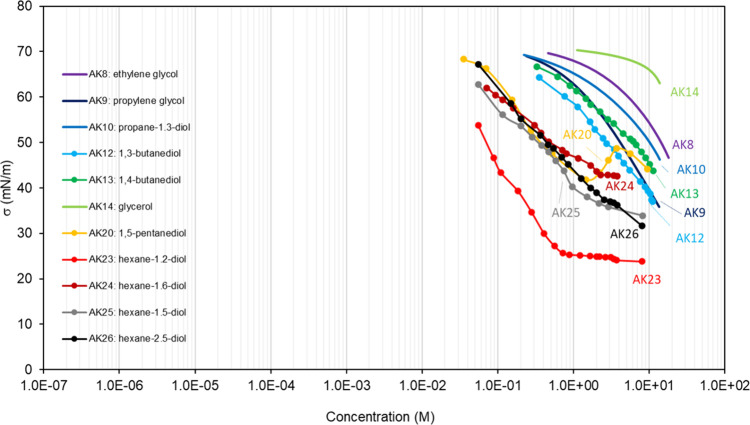
Adsorption isotherms for binary mixtures in water of di- and trialcohols. Curves represented by solid lines are recommended values for multiple data sets (see SI and text), while those with symbols represent single experimental data sets.

**Figure 13:**
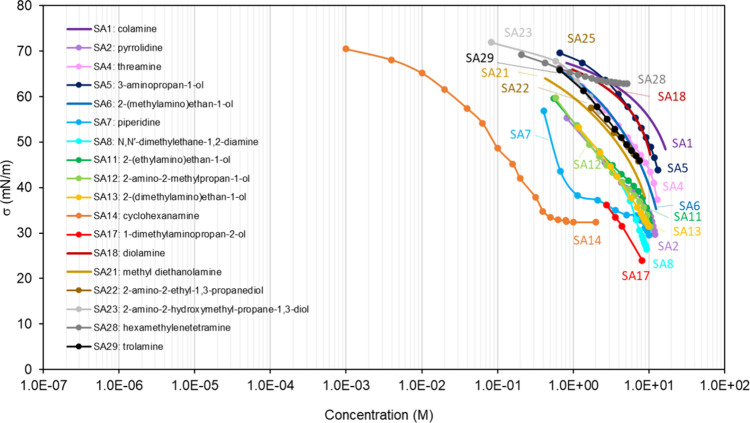
Adsorption isotherms for binary mixtures in water of amines. The curves represented by solid lines are recommended values based on multiple data sets (see SI and text), while those with symbols represent single experimental data sets.

**Figure 14:**
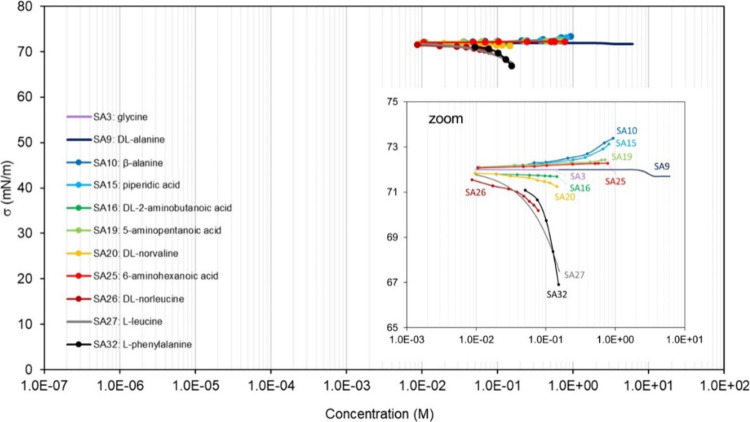
Adsorption isotherms for binary mixtures in water of amino acids. The curves represented by solid lines are recommended values based on multiple data sets (see SI and text), while those with symbols represent single experimental data sets.

**Figure 15:**
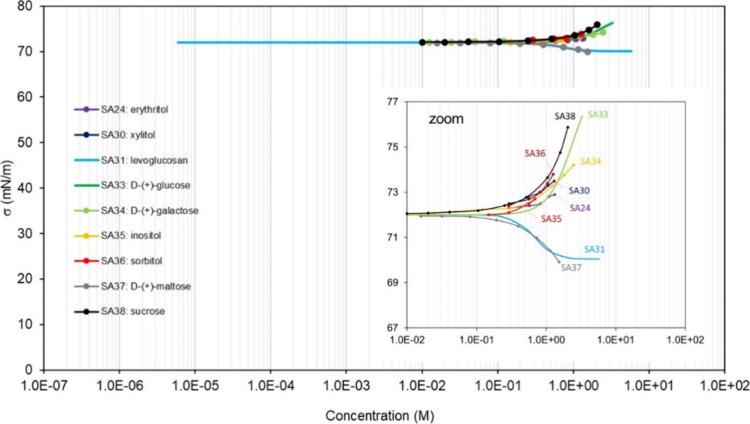
Adsorption isotherms for binary mixtures in water of sugars. The curves represented by solid lines are recommended values based on multiple data sets (see SI and text), while those with symbols represent single experimental data sets.

**Figure 16:**
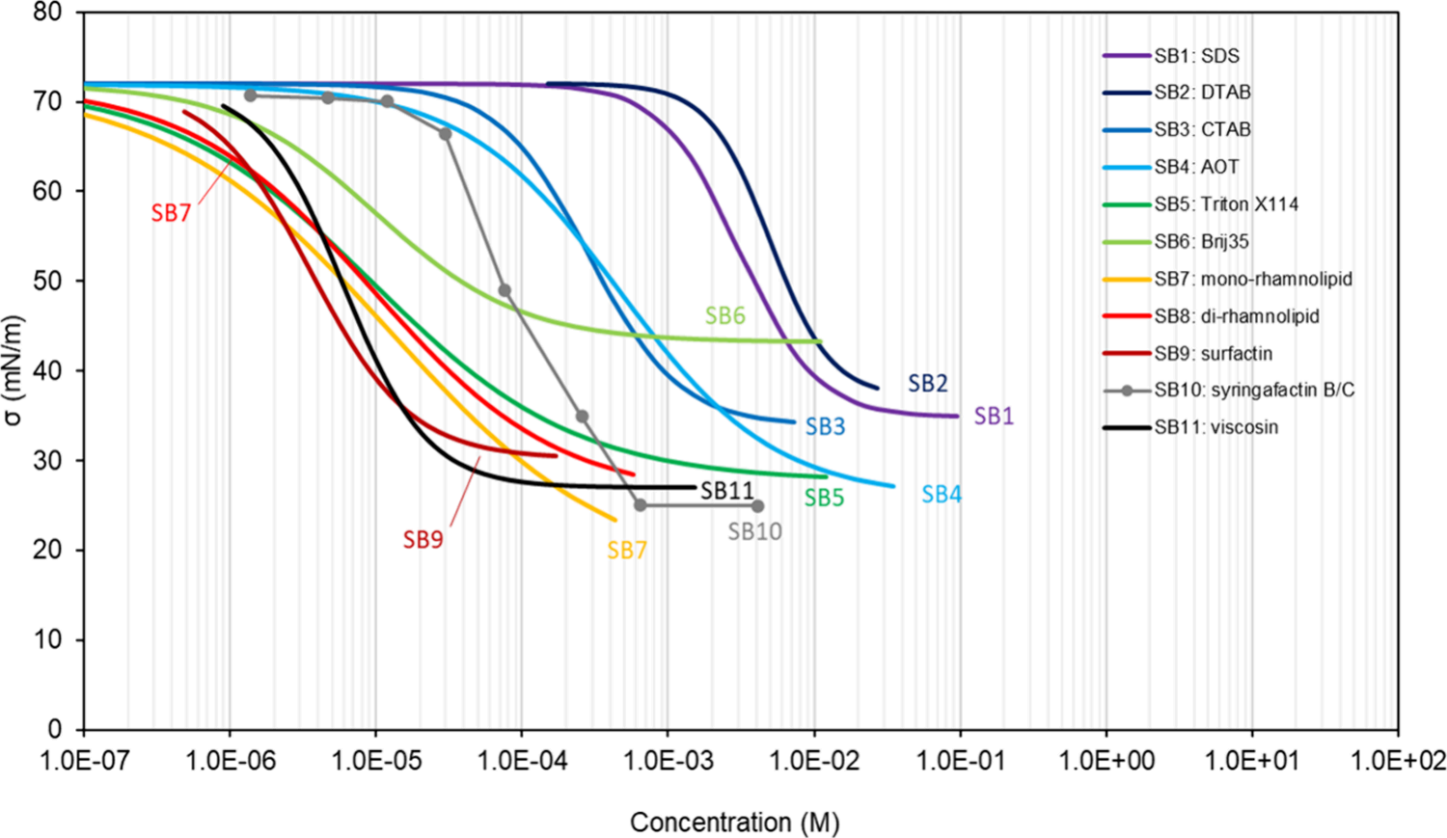
Adsorption isotherms for binary mixtures in water of amphiphilic and macromolecular compounds (synthetic and biological surfactants). The curves represented by solid lines are recommended values based on multiple data sets (see SI and text), while those with symbols represent single experimental data sets.

**Figure 17:**
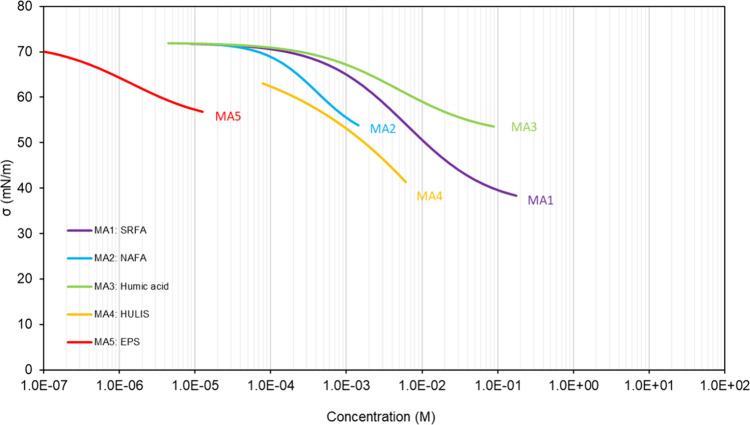
Adsorption isotherms for binary mixtures in water of macromolecules. The curves represented by solid lines are recommended values based on multiple data sets (see SI and text), while those with symbols represent single experimental data sets.

#### 4.1.1 Modeling and Recommended Curves for the Surface Tension Isotherms

For the mixtures for which more than one data set is reported in the literature, curves interpolating at best the different data sets were calculated and are recommended in the SI. These interpolated curves were obtained by performing, for each substance, nonlinear least-squares fits of the data set to a Sigmoid curve, according to the model developed by Kleinheins et al.^14^ (see also Section S2.1):
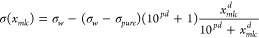
10with σ_w_ the surface tension of pure water, σ_pure_ the surface tension of the pure substance, *p* and *d* optimized parameters (position of the inflection and distance of the estimated CMC, critical micellar concentration, from the inflection point, respectively), and *x*_mlc_ the molar fraction. For substances where σ_pure_ is not known, this value was determined by fitting together with parameters *p* and *d*.

Nonlinear least-squares fits (RMSE, root mean squared error), 95% confidence intervals on the fit parameters, and the 95% confidence band were obtained using the module “kmpfit” of the Python package “Kapteyn”.^190^ The curves thus obtained are recommended only over the concentration range covered by the corresponding experimental data sets.

### 4.2 Compounds Included in the Inventory

Thousands of organic compounds are thought to be present in atmospheric aerosols, although different analytical techniques are only able to identify specific subclasses of compounds.^191^ Merging different studies reporting the analysis of PM2.5 and PM10 aerosols from remote regions of the Amazonia,^192^ rural regions of Europe^193,194^ and the USA,^195^ and urban areas of China,^196,197^ India,^198^ and the USA^195,199^ resulted in a list of ∼50 most abundant organic compounds in atmospheric aerosols, for which adsorption isotherms were searched in priority in the literature. Adsorption isotherms were found for about 25 of these compounds, marked with a “Y” in the “Atmos. ?” columns of Tables 1−5. However, for some relatively common compounds, such as glyoxal, glyoxylic acid, glyceric acid, pyruvic acid, pinic acid, and β-caryophillinic acid, no adsorption isotherms could be found, and the latter need potentially to be determined in future studies. In addition, more complex compounds, such as oxidation products of isoprene and terpenes, while shown to display some surface tension effects,^200^ are not included in this inventory because their structures are not completely elucidated. Furthermore, it can be seen in Table 2 that relatively little information is found in the literature on the adsorption isotherms for aldehydes and ketones in water, which could also be the subject of future studies. The inventory also includes some macromolecular compounds (C atoms > 20, Table 5), for which the molecular structure is not fully elucidated but which have been reported in atmospheric aerosols, such as Humic Like Substances (HULISs) and their commercial references, SRFA and NAFA fulvic acids and commercial humic substances, as well as Extracellular Polymeric Substances (EPSs) found in marine aerosols.

In addition to the organic compounds of direct relevance for atmospheric aerosols, over a hundred other organic acids, alcohols, amines, and sugars, for which the adsorption isotherm in water was reported in the literature, are also included in the inventory. The motivation for including all of these compounds is that they provide a wide range of molecular structures and carbon chain length, which is useful to link molecular structure and surface activity in models (see discussion below). Finally, the isotherms for a few amphiphilic compounds are also included in this inventory (Table 4) as they are an important class of compounds to take into account when investigating surface tension.

However, nonamphiphilic compounds with low solubility in water, such as alkanes, alkenes, and reduced aromatic and polyaromatic compounds, were not included in the inventory, even though they can be present in atmospheric particles, because they are not expected to act significantly as surfactants, especially at their aerosol concentrations.

### 4.3 Trends Observed in the Inventory: “Weak” vs “Strong” Surfactants

The adsorption isotherms presented in Figures 8−17 provide some general trends on the efficiency of different types of organic compounds in reducing the surface tension of aqueous mixtures.

#### 4.3.1 Lowering or Increasing the Surface Tension

Nearly all the organic compounds presented in Figures 8−17 tend to lower the surface tension of aqueous mixtures, albeit at large concentration, which was expected as most of them are semisoluble in water (cf. Section 1.2.1). Only the most soluble compounds, sugars (Figure 15), tend to increase the surface tension by forming hydrogen bonds with the water molecules (Section 1.2.1). Note, however, that this hydrogen-bonding effect is rather modest, with an increase of surface tension of δσ ≤ 5 mN m^–1^ relative to pure water, even at very large concentrations (C ≥ 0.5 M).

#### 4.3.2 Role of the Carbon Chain Length

The isotherms in Figures 8−15 clearly show that, within each class of compound, the efficiency of semisoluble organic molecules in lowering the surface tension of aqueous mixtures increases with the organic chain length. This was expected as the increase of surfactant efficiency with the organic (hydrophobic) chain length has been established for a long time.^29,30,201^ This is especially clear for the organic acids in Figure 8−10, for which the isotherms shift progressively toward lower concentrations as the chain length increases, and the alcohols in Figure 11 and, to a lesser extent, the amines and amino acids in Figures 13 and 14. Thus, in order to discuss the relative surfactant efficiency of different classes of compounds, it is necessary to compare compounds with identical organic chain length.

#### 4.3.3 Role of the Molecular Structure and Type of Substituents

**Figure 18:**
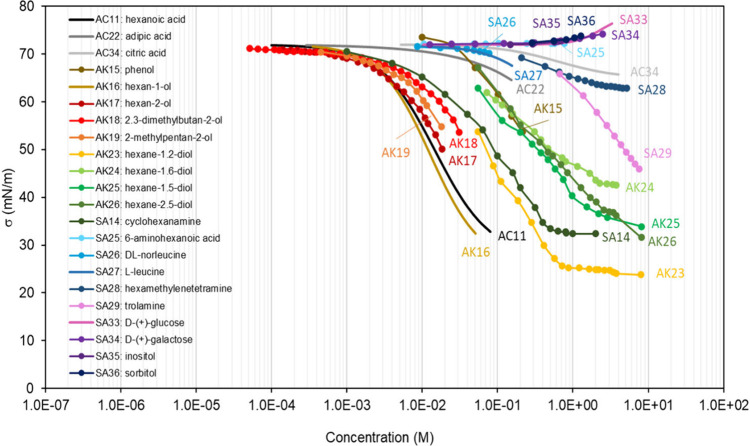
Comparison of the adsorption isotherms for nonamphiphilic organic compounds with 6 C atoms, evidencing the effect of different organic substituents on the surface tension of aqueous solutions. The curves represented by solid lines are recommended values based on multiple data sets (see SI and text), while those with symbols represent single experimental data sets.

Comparing the isotherms for the molecules with 6 carbon atoms in Figure 18 shows the relative surfactant efficiencies of different classes of organic molecules and substituents. The objective of the present inventory is only to gather all of these data for future studies but not to propose an in-depth analysis of it. However, the general trends displayed in Figure 18 can be briefly discussed. The effects of different compounds on the surface tension, Δσ, can be compared at a given concentration, for instance, C = 0.1 M, for which most isotherms are available. The most efficient surfactant in Figure 18 appears to be hexan-1-ol (AK16), with Δσ(0.1 M) ∼ 50 mN m^–1^. Then, for hexanoic acid (AC11) Δσ(0.1 M) ∼ 40 mN m^–1^, for the aromatic phenol (AK15) Δσ(0.1 M) ∼ 10 mN m^–1^ and for amines and amino acids, such as amino hexanoic acid (SA25), and leucine (SA26, SA27) it is negligible (Δσ(0.1 M) ∼ 0 mN m^–1^). Finally, the C6-sugars, glucose (SA33), galactose (SA24), inositol (SA35), and sorbitol (SA36), have a negligible effect at C = 0.1 M but tend to increase the surface tension at larger concentrations. Thus, between molecules with the same organic chain length, the general trend in surfactant efficiency in aqueous solutions is linear alcohols > linear acids ≫ linear amines and amino acids > sugars.

Within each class of compounds, the presence of multiple substituents (double or triple acids or alcohols), different isomers, or branching on the organic chain also affects the surfactant efficiency. For instance, for the diacid adipic acid (AC22) Δσ (0.1 M) ∼ 10 mN m^–1^ and for the triacid citric acid (AC34), Δσ (0.1 M) ∼ 0 clearly showing that the number of acid groups in the molecule decreases the surface tension effect. A similar effect is observed with the alcohols, the hexanediol isomers (AK23, AK24, AK25, AK26) having all smaller surface tension effects than hexan-1-ol, with Δσ (0.1 M) ∼ 15–25 mN m^–1^. Interestingly, there are large differences between the surfactant effects of the different isomers, hexan-1,2-diol (AK23) having much larger surfactant effects than the 1,5, 1,6, and 2,5 isomers. Finally, branched monoalcohols such as dimethyl butan-2-ol (AK18) and methyl pentan-2-ol (AK19) have slightly smaller effects than their linear counterpart hexan-1-ol, with Δσ (0.1 M) ∼ 30–40 mN m^–1^.

These simple comparisons evidence the importance of the molecular structure of nonamphiphilic organic compounds on their surface tension effect in aqueous solutions. The data presented in this section can thus be used in future studies to establish relationships between the molecular structure of nonamphiphilic organic compounds and their surface tension effects and, potentially, with other properties such as their solubility or polarity.

#### 4.3.4 Compounds of Potential Importance in Activated CCN and Cloud Formation

The isotherms presented in this section give some general ideas about the types of compound that can affect cloud droplet formation at activation and thus on those that should be taken into account or ignored when investigating these processes. At activation, CCN have typically undergone a volume increase due to water uptake by a factor 1000, thus a dilution of their components by the same factor. This implies that, in order for organic compound to have some significant effect (i.e., Δσ ≥ 10 mN m^–1^) at activation, their effect needs to be achieved for concentrations of C ≤ 10^–3^ M. Applying this criterion (Δσ ≥ 10 mN m^–1^ with *c* ≤ 10^–3^ M) to the isotherms in Figures 8−15 show that only molecules with at least 7 or 8 C atoms for the linear acids and alcohols, and at least 9 or 10 C atoms for amines and amino acids (based on the curve for the C9-phenylalanine, SA32) are likely to affect the surface tension at activation. By contrast, nearly all of the amphiphilic compounds in Figure 16 fulfill this criterion, except SDS (SB1) and DTAB (SB2).

Future investigations on cloud droplet formation should thus probably focus on such organic compounds, i.e., nonamphiphilic compounds with at least 8 or 9 C atoms and amphiphilic compounds except SDS and DTAB. The criterion Δσ ≥ 10 mN m^–1^ with C ≤ 10^–3^ M can thus be considered as an empirical distinction between “weak” and “strong” surfactants with respect to cloud droplet formation.

## 5 Current Knowledge of the Surface Tension of Atmospheric Particles

As underlined above, at the time of publication of this review, the surface tension of atmospheric particles has not been measured yet, although the techniques recently developed should soon converge toward such measurements. Different methodological approaches have thus been used to estimate the surface tension of atmospheric particles or determine the concentration of surfactants in such particles. Such measurements are summarized in Table 6 while the different methodologies used to determine the surface tension are described below. We distinguish two main types of approaches: the “top-down” ones, which consist in determining the surface tension of aerosol particles or populations based on direct or in situ atmospheric measurements (i.e., thus excluding any chemical treatment of the samples beside drying) and the “bottom-up” approaches, which consist in characterizing the surfactants extracted from atmospheric aerosols and calculating all the other relevant factors to estimate the average surface tension of the aerosol population. Bottom-up approaches are more frequent than top-down ones, both because few measurements techniques allow us to determine the surface tension of atmospheric particle populations and also because bottom-up approaches follow the historical step-by-step progression from first evidencing the presence of surfactants in atmospheric aerosols, to characterizing their properties, to characterizing all the other factors contributing to the surface tension.

**Table 6:** Surface Tension, CMC, and Concentration of Aerosols from Atmospheric Samples Reported in the Literature (Articles in Chronological Order)^a^

Sample type	Particle diameter	Location	Surfactant concentration	Extraction method/sample treatment	CMC (M)	Minimum surface tension σ_min_ (mN m^–1^)	Tensiometry method
fog water		Dübendorf, Zwitzerland		filtration		60.9	DVT^154^
fog water		Po Valley, Italy		evaporation		58.8	PB^155^
fine aerosols	<1.5 μm	Po Valley, Italy		water extraction		62.3	PB^156^
fog water		Po Valley, Italy		filtration		55.5	
cloud water		Puy de Dôme, France				72.6	
cloud water		Tenerife, Canary Island Spain				72.7	
cloud water		Rax, European Alps, Austria				62	NR^362^
semiurban aerosols	coarse mode (>1 μm)	Norwich, England	AS (MBAS): ∼1.95 (0.0–5.67) μmol g^–1^	water extraction		68.5–71.2	PD^157^
			AS (EVAS): ∼11 (1.9–21.9) μmol g^–1^				
			CS (DBAS): ∼0.17 (0.00–1.22) μmol g^–1^				
	fine mode (<1 μm)		AS (MBAS): ∼4.7 (0.57–14.3) μmol g^–1^				
			AS (EVAS): ∼23.9 (4.4–699) μmol g^–1^				
			CS (DBAS): ∼0.31 (0.00–1.45) μmol g^–1^				
	total suspended solid (TSP)		AS (MBAS): ∼1.2 (0.28–3.07) μmol g^–1^				
			AS (EVAS): 7.8 (2.4–9.5) μmol g^–1^				
atmospheric aerosols	stage 1: 0.2–0.5 mm	Jeju Island, Korea		water extraction		stage 1: 71.8	PD^158^
	stage 2: 0.5–1.5 mm					stage 2: 71.4	
	stage 3: 1.5–5.5 mm					stage 3: 71.9	
	stage 4: 5.5–10 mm					stage 4: 72.0	
cloud water	I: 7–11 mm			evaporation		I: 69.1	
	III: > 17 mm					III: 64.4	
semiurban aerosols	PM_10_	Bangi, Malaysia	AS (MBAS): 2.5 (2.1–3.4) μmol g^–1^	water extraction		67.2	PD^160^
			AS (EVAS): 12.6 (10.4–39.0) μmol g^–1^				
			CS (DBAS): 0.06 (0.00–0.16) μmol g^–1^				
urban aerosols	PM_10_	Penang, Malaysia	AS (MBAS): 4.7 (1.1–6.7) μmol g^–1^			∼68.5	
			AS (EVAS): 13.6 (4.6–18.1) μmol g^–1^				
			CS (DBAS): 0.15 (0.03–0.30) μmol g^–1^				
Amazonian aerosol (during dry, transition and wet periods)	5 stages from 0.05 to 10 μm	Rondônia, Brazil		water extraction		50.3	PB/PD^159^
fine biomass burning particulate matter	PM_2.5_	Georgia (Augusta and Columbus), USA		water extraction		59	PD^64^
temperate forest aerosols	PM_10_	Hyytiälä, Finland		water extraction		∼59.6	PD^161^
Amazonian forest aerosols	PM_10_	Manaus, Brazil				53.1	
temperate forest aerosols	PM_10_	Hyytiälä, Finland		double extraction (water + silicone microextraction)		28.5	
coastal aerosols	PM_2.5_	Aspvreten, Sweden				29.0	
Amazonian forest aerosols	PM_10_	Manaus, Brazil				27.3	
urban aerosols	PM_10_	Grenoble, France		double extraction (water + silicone microextraction)		∼30 (summer)	PD^162^
						35–45 (winter)	
forested/coastal aerosol	PM_2.5_	Aspvreten, Sweden				∼40	
urban aerosols	PM_10_	Grenoble, France		double extraction (water + silicone microextraction)		28–42	PD^22^
coastal aerosols	PM_2.5_	Askö, Sweden	TS: 3.8 (2.7–14.3) × 10^–2^ M	double extraction (water + one SPE cartridge)	^*m*^1.34 (0.49–2.45) × 10^–4^	^*m*^33.7 (32.1–39.8)	PD^163^
atmospheric fog and cloudwater samples		Italy (Po Valley), North East Atlantic (Tenerife), Korea		evaporation		40	PD^164^
coastal aerosols	PM_1_	Rogoznica, Croatia	TS: 22.2 (7.2–134.3) × 10^–3^ M	wouble extraction (water + one SPE cartridge)	^*m*^1.90 (0.45–3.30) × 10^–4^	^*m*^36.0 (29.8–46.0)	PD^165^
urban aerosols	PM_1_	Lyon, France	TS: 73.0 (12.8–1333.1) × 10^–3^ M		^*m*^12.00 (0.34–92.0) × 10^–4^	^*m*^34.8 (27.0–47.1)	
remote (edge of the boreal forest) aerosols	PM_1_	Pallas, Finland	TS: 48.6 (8.9–1110.4) × 10^–3^ M		^*m*^1.60 (0.57–8.20) × 10^–4^	^*m*^35.0 (28.2–48.7)	
aerosols from estuarine water environment	0.560–1 μm and 1.–3.2 μm size bins	Skidaway Island, USA		double extraction (water + two SPE cartridges)		53.4	PD^166^
marine influenced aerosols	submicrometer (4 sizes ranges)	Skidaway Island, USA		double extraction (water + two SPE cartridges)		36.8 (34.8–39.5)	PD^167^
	supermicrometer (6 size ranges)					42.2 (37.5–48.3)	
mixed (marine/continental) influenced aerosols	submicrometer (4 size ranges)					40.4 (38.5–42.7)	
	supermicrometer (6 size ranges)					40.8 (37.9–45.9)	
urban aerosols	<0.95 μm	Nagoya, Japan		water extraction		48.3–55.4	QELS^168^

a (*m*) median; AS (MBAS): anionic surfactants by methylene blue active substances colorimetric method; AS (EVAS): anionic surfactants by ethyl violet active substances colorimetric method; CS (DBAS): cationic surfactants by disulfine blue active substances colorimetric method; TS: Total surfactant fraction: anionic surfactants + cationic surfactants + nonionic surfactants; TOC total organic compounds; WSOC: water-soluble organic carbon Content; ; PM10, PM2.5, PM1: particulate matter with diameter below 10 μm, 2.5 μm, and 1 μm, respectively; TSP: total suspended solids; SPE: solid phase extraction; NR: Nouy ring; DVT: Drop Volume Tensiometry; PB = pending bubble; PD = pending droplet (shape of a droplet); QELS: quasi-elastic light scattering.^168^

### 5.1 Bottom-Up Approaches

#### 5.1.1 Evidencing and Characterizing Surfactants in Atmospheric Particles

**Figure 19:**
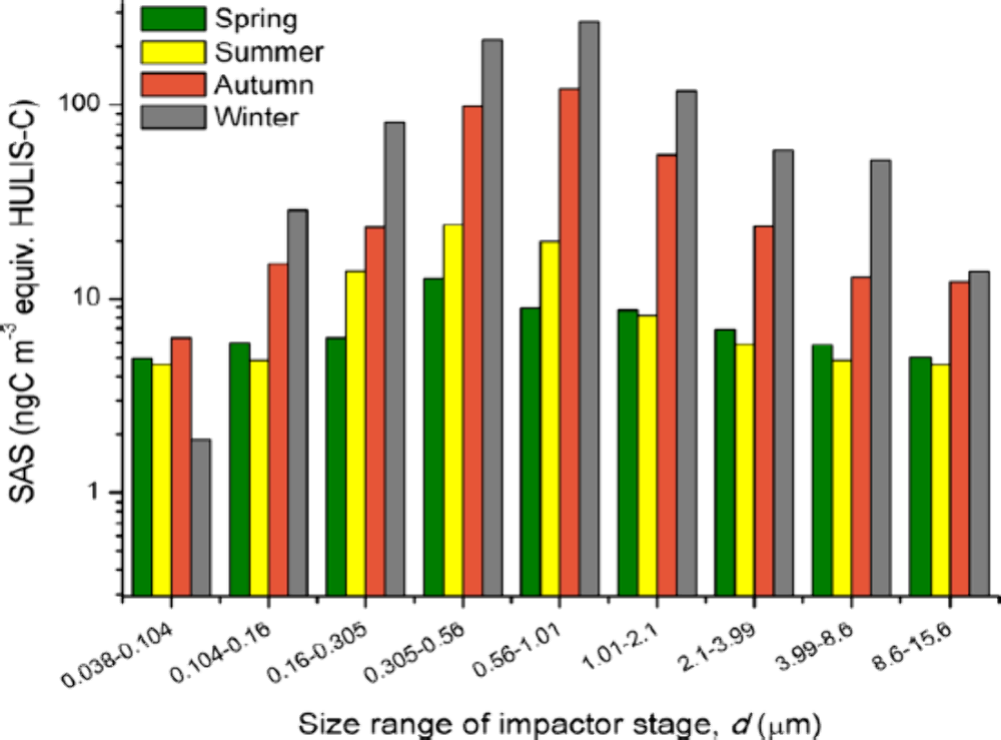
Seasonal distribution of the mass of surface-active compounds (SAS) throughout different size fractions of aerosols at an urban site in Ljubljana, Slovenia. Reproduced with permission from ref (227). Copyright 2018 American Chemical Society.

Interestingly, the identification and measurement of surfactants in the natural environments^202^ and in atmospheric waters and aerosols both started in the 1990s and early 2000s.^41,44,64,154−156,158,159,161,162,164^ For the atmospheric samples, these measurements proceeded essentially by extracting specific fractions from the samples: water-soluble fraction,^154−156,158,159,164^ humic-like substances,^41,44^ hydrophobic and hydrophilic fractions,^64^ or total amphiphilic fraction.^161,162,166,167^ However, quantifying their effects on the surface tension in term of absolute concentration (i.e., determining their adsorption isotherms) took more time, as the exact molecules responsible for the surface tension effects could not be isolated in these early studies (except for humic-like substances).^41,44^ The development of methods to measure the concentration of surfactants in atmospheric aerosols using dyes and colorimetric techniques^157,160,203−225^ or electrochemical techniques (Figure 19),^226,227^ not only further confirmed the presence of surfactants in atmospheric particles, but has allowed to identify some of their sources,^208,212,214^ or to characterize their distribution in different aerosol size fractions (Figure 19).^227^

Combining these quantification techniques with extractions targeting specifically amphiphilic compounds then allowed to establish the adsorption isotherms for the amphiphilic fractions of PM2.5 and PM1 aerosols from urban, rural, and remote regions of the atmosphere^163,165,228^ and for seawater-generated aerosols.^229^ These isotherms display CMC that are typically in the range 5 × 10^–5^–10^–3^ M, thus indicating that, without other effects (mixing or bulk-to-surface partitioning), these surfactant fractions should be able to reduce the surface tension of forming water droplets even at activation.

#### 5.1.2 Mixing Effects with Other Aerosol Components

Once atmospheric surfactants are characterized, the next factor potentially affecting their contribution to the surface tension of the aerosol particles is their mixing with other aerosol components. A number of studies have investigated the effects of adding inorganic salts, NaCl or (NH_4_)_2_SO_4_ to aqueous solutions containing either semisoluble organic compounds,^49,61−63,65−68^ humic substances,^41^ or amphiphilic surfactants.^60,68−74^ To a few exceptions,^66,107^ they report a significant reduction of the surface tension due to the addition of salt. This effect was observed not only with bulk solutions but also with submicrometer particles, for which measurements of CN/CCN numbers^49,67^ or CCN growth factors^64,67,78^ indicated a reduction of the critical supersaturation, which could be unambiguously attributed to a surface tension reduction rather than hygroscopicity effects. Some of these studies even reported a reduction of the critical supersaturation to values that were lower than with the organic particles or salt particles, thus evidencing synergistic effects.^49,64^ As explained in Section 1.2.5, this surface tension reduction is due to the salting out of the surfactants to the surface of the liquid.^15,68^

Fewer studies have explored the effects of mixing amphiphilic surfactants with organic aerosol components, such as organic acids. In other fields of application, investigations of aqueous mixtures of SDS^230^ and CTAB^231^ with ascorbic acid reported a reduction of the CMC upon addition of ascorbic acid. More recently, investigations of aqueous mixtures of Triton X100, Brij35, and CTAC with oxalic and glutaric acid reported some nonideal behavior in term of surface tension, i.e., a surface tension for the mixture which was lower than expected from the sum of the contributions of the two components and, in some cases, some synergistic effects.^68^ As organic acids, such as oxalic, succinic, or glutaric acids, are very common in atmospheric particles, more of their mixtures with amphiphilic compounds must be studied. In particular, these mixing effects should be best studied with surfactants extracted from authentic atmospheric samples rather than with reference compounds.

Based on the observations made so far, it seems that mixing surfactants with major aerosol components such as inorganic salts and organic acids would further decrease the surface tension. However, more of such mixtures would need to be studied to confirm these trends. These mixing effects would also need to be investigated with surfactants extracted from atmospheric samples and not only with reference compounds.

#### 5.1.3 Evidencing Surface Tension Depression and Bulk-to-Surface Partitioning Effects in Microscopic Droplets

Other effects that need to be investigated experimentally and taken into account when estimating the surface tension of atmospheric particles by bottom-up approaches are bulk-to-surface partitioning effects. Few experiments have been able to explore these effects and seem to yield contradictory conclusions. Experiments in which submicrometer particles (mean radius ∼250 nm) of (NH_4_)_2_SO_4_ were exposed to organic vapors (methylglyoxal and acetaldehyde) reported a decrease of the critical supersaturation based on CN/CCN measurements, thus showing that surface tension effects overcome the potential bulk-to-surface partitioning effects.^110^ Similarly, submicrometer particles (150–650 nm in diameter) coated with organic compounds (oxidation products from terpenes) displayed an increase of their growth factors measured with a custom-made chamber at a Relative Humidity (RH) up to 99.9%, which was unambiguously attributed to a surface tension depression,^10,232−234^ and thereby showing the dominant effect of surface tension over bulk-to-surface partitioning. The critical saturation, measured from CN/CCN numbers, for submicrometer particles containing hydrophilic surfactants from biomass burning aerosol was shown to be reduced by adding (NH_4_)_2_SO_4_ to below the critical saturation of pure (NH_4_)_2_SO_4_ particles, thus clearly evidencing surface tension effects over hygroscopic effets^64^ and either the absence or limited impact of bulk-to-surface partitioning effects.

Recent studies of the surface tension of ∼5–12 μm-diameter particles with optical tweezers, on the other hand, reported more contrasted results. For aqueous solutions of semisoluble organic compounds such as glutaric acid,^174^ and some amphiphilic surfactants such as octyl-β-d-1-thioglucopyranodide (OTG)^180^ a good agreement is reported between with the surface tension of the microscopic particles and large-volume samples. However, for other amphiphilic surfactants, such as Triton X100,^178^ TWEEN20, the glycol ethers C_16_E_8_, C_12_E_5_, C_10_E_8_, and C_14_E_6_, and their mixtures with NaCl and glutaric acid,^180^ the surface tension of the microscopic particles was significantly larger than that of large-volume samples of the same mixtures, which was attributed to bulk-to-surface partitioning effects.

The contrasted results obtained with different types of experiments do not allow one to conclude on the importance of bulk-to-surface partitioning effects on the surface tension yet. As discussed in Section 6, it is possible that some of these discrepancies result from the different techniques used to produce the particles in these experiments.

### 5.2 Top-Down Determination of the Surface Tension of Atmospheric Particles

Until now, the only examples of top-down determination of the surface tension of atmospheric particles are based on the measurements of atmospheric CCN growth factors and numbers. However, as mentioned in Section 2, because such measurements are rather unsensitive to surface tension, only a limited number of them have reported estimates of the surface tension of atmospheric particles: CCN growth measured in the tropical Atlantic Ocean and Central Germany indicated that these particles might have a surface tension between 50 and 70 m Nm^–1^ at activation,^111,112^ and recent CCN measurements in Southern China for newly formed particles report σ ∼ 60 mN m^–1^ above activation (supersaturation = 1%).^113^

Other types of measurements could be applied to atmospheric samples for the top-down determination of the surface tension in the future. Those include, for instance, the development of techniques to measure the vaporization enthalpy of atmospheric samples, a quantity known to be correlated to surface tension. More sophisticated techniques, such as surface-specific spectroscopies, Sum Frequency Generation^235^ or Second harmonic Scattering,^236^ could also be employed and use some vibrational properties of the surface molecules in atmospheric samples as a proxy for the surface tension. These approaches can only be applied to aerosol samples (for instance deposited on a substrate) and not individual particles but would provide overall values for the surface tension of the particle population, i.e., including mixing, partitioning, and all other relevant effects, unlike bottom-up approaches.

## 6 Conclusions and Perspectives

After being ignored or questioned for decades,^8,108,111,112,237,238^ surface tension depression in atmospheric particles has been shown by the many measurements reported in this review to be likely to occur and worth further investigating, in particular by developing sophisticated new techniques.^9,164,239,240^ However, major gaps remain in the knowledge of the surface tension of atmospheric particles and of its role in atmospheric processes. The following sections propose some potential future directions of research that could help to fill these gaps.

### 6.1 The Technical Challenges

Although the main objective when studying the surface tension of atmospheric particles is to answer scientific questions about atmospheric processes, the lack of techniques to measure the surface tension of small particles and to investigate related properties has been a major hurdle in the progress of this field of investigation. These technical limits are thus discussed first, focusing on a few major objectives.

#### 6.1.1 Measuring the Surface Tension of Individual Atmospheric Particles

At the time of this review, the most important technical objective in this field of investigation should probably be to achieve direct measurements of the surface tension of individual atmospheric particles, as this would allow us to determine realistically the importance of this parameter in atmospheric processes and bypass all the uncertainties involved in bottom-up estimates. Such direct measurements could potentially be achieved by further developing the techniques that are already used to investigate the surface tension of individual particles (Section 3.2), such as optical tweezers or nanotensiometry (e.g., AFM). Besides direct measurements on individual particles, other “top-down” approaches, i.e., determining the surface tension of atmospheric particles without any (or only minimal) chemical treatment (cf. Section 5), would also be very valuable. As discussed in Section 5.2, techniques and approaches to be developed could be based, for instance, on measuring the vaporization enthalpy of atmospheric samples, as this parameter is known to be closely linked to the surface tension.^141,142^ Since surface tension reflects molecular interactions at the surface, it is possible that some vibrational properties of the surface molecules could be used as a proxy for the surface tension. Or, if this is not the case for the surfactants alone, then adding dyes forming complexes with the surfactants might display such vibrational signatures. If so, surface-specific spectroscopies, such as Sum Frequency Generation^235^ or Second Harmonic Scattering,^236^ could be employed to determine the surface tension of particle populations. Unfortunately, neither the measurement of vaporization enthalpy nor surface-specific spectroscopies are applicable to individual particles; they require sample masses of the scale of a particle population. And, in the case of the spectroscopic techniques, the particles would have to be deposited on a substrate. However, such approaches would be helpful, as they would provide net surface tension values for the particle population, i.e., including all mixing and partitioning effects (albeit averaged over the population).

#### 6.1.2 Measuring the Surfactant Concentration in Individual Particles

Another parameter that is essential for the understanding and prediction of the surface tension of atmospheric particles but represents a major technical challenge is the surfactant concentration in the individual particles. Until now, only population-averaged surfactant concentration can be determined based on laborious extraction and colorimeric or electrochemical approaches (see Section 5.1.1). The techniques the most likely to be further developed into measuring surfactant concentration in individual particles are those used today to study individual particles: electrodynamic balance or optical tweezers or AFM. The quantification of surfactant molecules at the surface could be based on emission or scattering spectroscopies, with or without the addition of surfactant-complexing dyes.

#### 6.1.3 Generating Artificial Particles of Controlled Composition

**Figure 20:**
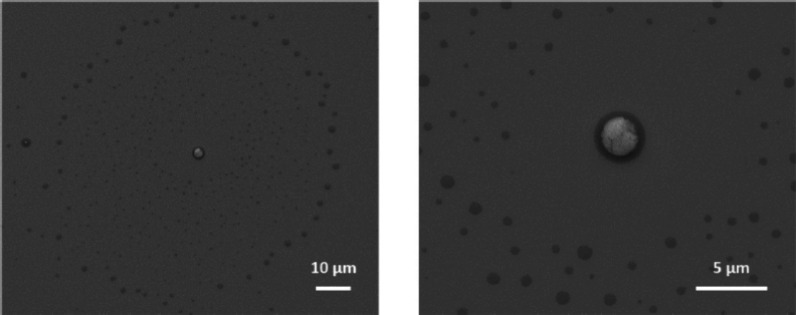
Impaction of particles generated by nebulizing an aqueous solution of (NH_4_)_2_SO_4_ 0.1 M/Triton X114, 0.1 M onto a silicon wafer. A salt particle (∼3 μm) is visible at the center of the picture, and the dark spots are the surfactant. The spot patterns around the particle show that most of the surfactant from the solution is ejected outside the particles during the evaporation step of the nebulization. Picture obtained with a scanning electron microscope (ESEM, Quattro, ThermoFisher Scientific, USA).^241^

Last but not least, making substantial progress in the fundamental understanding of surface tension and its role in atmospheric processes would require the ability to produce micrometer- or submicrometer-sized particles in a controlled way in the laboratory, i.e., with a known size and surfactant concentration. While generating aqueous particles with a controlled concentration of inorganic salts is routinely done with nebulization or atomization techniques, it is much more difficult to control the particle surfactant content with such techniques. This is because the amount of surfactant transferred to the particles during the breakdown of the bulk surface into small droplets in the evaporation process is uncontrolled. Such a separation between the inorganic particles and the organic surfactant was evidenced, for instance, by the electron microscopic analyses (SEM-EDX) of mixed ammonium sulfate–Triton X114 solutions nebulized onto silicon wafers.^241^ These analyses showed that the surfactant (characterized by a majority of C atoms) was largely ejected from the ammonium sulfate particles (characterized by a majority of N- and S atoms) during nebulization (Figure 20).^241^ The difficulty in generating controlled particles is worsened by the absence of technique allowing us to verify the surfactant concentration in the generated particles. Thus, the development of better particle-generation techniques goes hand in hand with those techniques to measure surfactant concentration in individual particles. Until such individual particle techniques are developed, those based on colorimetric or electrochemical techniques (Section 5.1.1) could be applied to verify the surfactant concentration in laboratory-generated particles. Alternative particle-generation techniques, minimizing evaporation, could also be explored, such as the use of particle dispensers, as for instance in ref.^168^ An approach that ensure the best transfer of surfactant to the particles is probably that consisting in coating inorganic seed particles with organic surfactants by condensation.^10,110,232−234^ Unfortunately, this approach is difficult to apply to amphiphilic surfactants as they are not volatile. However, it can probably be attempted with a wide range of semisoluble (and semivolatile) organic surfactants. As emphasized above, generating controlled particles is essential for the fundamental understanding of surface tension effects in small particles. In particular, the different conclusions reported by different studies on the importance of bulk-to-surface partitioning on surface tension (Section 5.1.3) could result from using different particle-generation techniques. The different techniques used in these studies include the condensation of organic surfactants onto inorganic seeds,^10,110,232−234^ and others atomization/nebulization of aqueous mixtures,^178,180^ thus most likely resulting in very different surfactant contents in the particles.

### 6.2 The Scientific Challenges

#### 6.2.1 A Better Knowledge of the Properties of Atmospheric Surfactants

**Figure 21:**
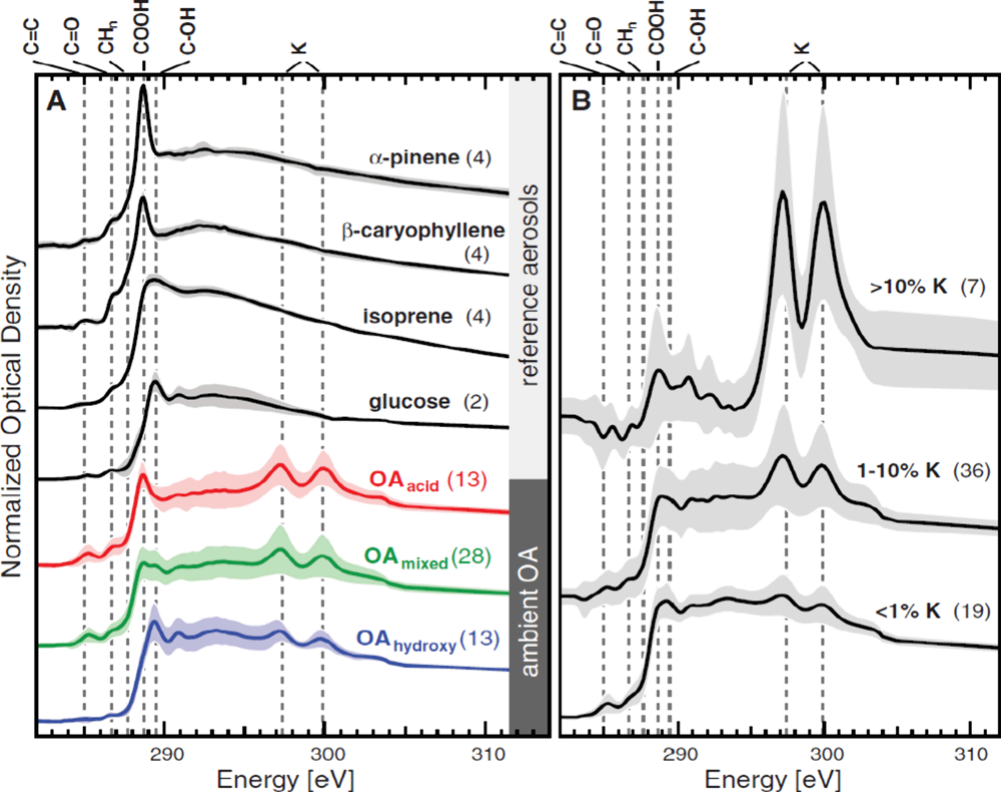
Identification of the Organic Groups at the surface of individual particles with STXM-NEXAFS. A) Laboratory-generated Secondary Organic Aerosol from terpenes and isoprene oxidation; B) Amazonian organic aerosols with different potassium mass fractions. Reproduced from ref (243). Copyright 2012 American Association for the Advancement of Science.

Besides addressing important scientific questions regarding the role of surface tension in atmospheric processes, many properties of atmospheric surfactants are still unknown and would require investigations in order to better understand and predict their impacts. Among the most important aspects to investigate is the identification of the compounds responsible for most of the surface tension depression in atmospheric particles and of their molecular structures. Although these compounds might vary between different regions, such identification would considerably simplify the investigation and prediction of their effects by identifying specific molecules or proxies that could be studied in the laboratory and by enabling targeted identification and characterization of their sources. Identifying the compounds responsible for most of the surface tension depression could proceed by isolating different extracts from atmospheric aerosols and comparing their surface tension. This has been done, for instance, for biomass burning aerosols,^64^ from which hydrophilic and hydrophobic organic fractions were extracted. While both fractions displayed some surface-active properties (with σ = 68 and 35 mN m^–1^, respectively), the hydrophobic fraction was the most surface-active. Identifying the compounds having the strongest surface-active contribution would then require further chemical analysis of this hydrophobic fraction, probably with separative techniques (gas or liquid chromatography). Alternative approaches to determine the type of surfactant having the most effect on the surface tension of atmospheric particles could be by investigating the presence of specific organic groups in the molecules present at the surface of particles using surface-specific spectroscopies.^235,236^ X-ray spectroscopy methods, such as scanning transmission X-ray microscopy, or STXM,^242^ sometimes combined with near-edge X-ray absorption fine structure analysis (STXM-NEXAFS),^243^ have thus been able to identify some specific organic groups at the surface of individual micrometer and submicrometer aerosol particles (Figure 21). Recently, X-ray photoelectron spectroscopy (XPS) investigation of aqueous solutions containing surface-active organics (carboxylic acids, alkyl-amines) were able to measure some important molecular information such as the surface enrichment of the organics and changes in the preferential orientation of the molecules at the surface, hydrophobic alkyl chains pointing increasingly outward from the solution.^244^ Such techniques are very promising for the characterization of the compounds acting on the surface tension of the droplets. A complete identification of the surfactant molecular structure would, however, probably require full (bulk) chemical analysis and would be a challenging task in itself. Such analyses have only been recently attempted.^166^

#### 6.2.2 A Better Understanding of the Role of Surface Tension in Cloud Droplet Formation

As indicated in Section 2.1, cloud droplet formation is, by far, the process in which the role of the surface tension of particles has been the most studied. It is also one of the most important atmospheric processes, both for the sustainability of life on Earth, human activities, and climate. Yet, the role of surface tension in cloud droplet formation has not been established yet. Some potential future directions of research that could help in elucidating these questions have been presented in Section 2.5. Besides the development of techniques to measure the surface tension of individual particles, allowing one to quantify its importance in cloud droplet formation, more global approaches are proposed, such as developing methods to collect separately CCN or cloudwater from interstitial aerosols and comparing the surface tension of the different samples. Even more global approaches could be based on establishing statistical relationships, such as correlations or causality relationships,^140^ between long-term measurements of cloud properties and of surface tension in aerosols at given locations. The advantage of the latter approach is that, provided that the data is available, it would allow investigating the effects of surface tension on specific cloud properties such as the droplet number, size distribution, cloud lifetime, etc.

#### 6.2.3 A Better Understanding of the Role of Surface Tension in Other Atmospheric Processes

As underlined in Section 2, there are several other atmospheric processes in which the surface tension is expected to play a role, based on observations in other fields of science, but which remain almost entirely to be studied in the case of atmospheric particles. This includes the role of surface tension in the nucleation of new particles and in the uptake and release of gases by atmospheric particles. As indicated in Section 2.5, many aspects of these processes could be studied in the laboratory using model compounds. Understanding the role of surface tension in these processes could thus improve the prediction of the nucleation of new particles in the atmosphere and of the chemical evolutions of aerosol particles.

### 6.3 The Societal Challenges

Besides the scientific questions, the atmospheric processes in which surface tension is expected to play a role are linked to a range of environmental issues, such as water precipitation or drought, air quality, and global warming. Some research efforts could thus address these issues more directly to get a better understanding of their causes and mechanisms and perhaps identify some solutions.

#### 6.3.1 Water Precipitation

If surface tension plays a significant role in cloud droplet formation, it is expected to affect the size distribution of cloud droplets and, therefore, the precipitation (rain) probability or frequency. Thus, for instance, investigating the type of surfactants affecting precipitation in some regions and having a knowledge of their sources in the local biosphere or microbiosphere,^245^ might help locally to identify solutions (in term of land use) to curb or mitigate recurring drought events. Another example is cloud seeding, to which numerous communities throughout the world are resorting today,^246^ and which represent substantial investments. While such solutions need to be used sparingly and only as emergency measures, understanding the role of surface tension in these processes could help make them less polluting by replacing silver iodide or other salts with less polluting seeds. Understanding the role of surface tension could also make cloud seeding more efficient, thus reducing the number of flights or shots necessary and also reducing the environmental impact.

#### 6.3.2 Fog-Haze Pollution

An air quality issue in which surface tension might also play a role are the recurring fog-haze events in Asia^247^ and other parts of the World, characterized by a strong water uptake by atmospheric particles. Understanding the potential role of surface tension in the large water uptake of the particles could possibly help to find measures to reduce the duration or intensity of such events, for instance, by favoring the growth and precipitation of the particles.

In conclusion, studying the surface tension of atmospheric particles and its role in atmospheric processes is a relatively young field of research, which has emerged over the last two decades. The number of studies and published articles on these topics has increased tremendously over these last decades, with even the formation of a small community around these questions in the last years. However, many scientific questions remain to be answered, and many technical challenges need to be overcome to answer them. It is, therefore, a promising and exciting topic for the future at the interface between fundamental science and environmental issues.

## Supporting Information Available

Section S1 (pp. S6–S7): Concentration conversions used in the inventory. Section S2 (pp. S8–S10): Determination of recommended values for the surface tension isotherms. Section S3 (pp. S11–S208): Adsorption isotherm data and recommendations. (PDF)
