# Supplementary material for: Measuring the Surface Tension of Atmospheric Particles and Relevant Mixtures to Better Understand Key Atmospheric Processes
Source: Chem Rev. 2024 Aug 23;124(19):10924–63. doi: 10.1021/acs.chemrev.4c00173 (PMC11467905; doi:10.1021/acs.chemrev.4c00173)
Supplement: Supplementary file 1 — cr4c00173_si_001.pdf [file cr4c00173_si_001.pdf]

Supplementary Information on the manuscript

Measuring the Surface Tension of Atmospheric Particles and Relevant Mixtures to Better Understand Key Atmospheric Processes

*By Manuella El Haber,[1] Violaine Gérard,[1,†] Judith Kleinheins,[2] Corinne Ferronato,[1] and Barbara Nozière[3]**

[1]Institut de Recherches sur l'Environnement et la Catalyse de Lyon (IRCELYON), CNRS and Université Lyon 1, Villeurbanne, 69626, France

[2]Institute for Atmospheric and Climate Science, ETH Zürich, Universitätstrasse 16, 8092 Zürich, Switzerland.

[3]Department of Chemistry, KTH Royal Institute of Technology, Stockholm, 114 28, Sweden

[†]Now at Laboratoire Eurofins Hydrologie Est, Eurofins Environnement France, 54320 Maxéville, France.

Contents

S1. Concentration conversions used in the inventory S6

S1.1: Molar concentration as a function of molality S7

S1.2: Molar concentration as a function of molar fraction S7

S1.3: Molar fraction as a function of molar concentration S7

S1.4: Molar concentration as a function of mass fraction S7

S1.5: Molar concentration as a function of volume fraction S7

S1.6: Molar concentration as a function of mass per volume fraction S7

S1.7: Molar fraction as a function of molar fraction per moles of water S7

S2. Determination of recommended values for the surface tension isotherms S8

S2.1. Least-square fit to Sigmoid model S8

S2.2 Optimized parameters obtained in the calculations S9

S3. Adsorption isotherm data and recommendations S11

S3.1. Organic acids S11

AC1: formic acid (methanoic acid) S11

AC2: acetic acid (ethanoic acid) S13

AC3: propionic acid (propanoic acid) S15

AC4: butyric acid (butanoic acid) S17

AC5: oxalic acid (ethanedioic acid) S19

AC6: methanesulfonic acid S21
AC7: valeric acid (pentanoic acid) S23
AC8: malonic acid (propanedioic acid) S25
AC9: β-hydroxybutyric acid (3-hydroxybutanoic acid) S28
AC10: maleic acid ((2Z)-but-2-enedioic acid) S29
AC11: caproic acid (hexanoic acid) S31
AC12: succinic acid (butanedioic acid) S33
AC13: benzoic acid (benzenecarboxylic acid) S36
AC14: cyclohexylmethanoic acid (cyclohexanecarboxylic acid) S37
AC15: enanthic acid (heptanoic acid) S38
AC16: glutaric acid (pentanedioic acid) S40
AC17: malic acid (2-hydroxybutanedioic acid) S44
AC18: p-toluic acid (4-methylbenzoic acid) S46
AC19: 3-hydroxybenzoic acid S47
AC20: cyclohexylethanoic acid S48
AC21: caprylic acid (octanoic acid) S49
AC22: adipic acid (hexanedioic acid) S50
AC23: 4-ethylbenzoic acid S52
AC24: cyclohexylpropanoic acid S53
AC25: pelargonic acid (nonanoic acid) S54
AC26: 4-propylbenzoic acid S56
AC27: phthalic acid (benzene-1,2-dicarboxylic acid) S57
AC28: cyclohexylbutanoic acid S58
AC29: capric acid (decanoic acid) S59
AC30: 4-butylbenzoic acid S61
AC31: pinonic acid (3-acetyl-2,2-dimethylcyclobutyl)acetic acid) S62
AC32: undecylic acid (undecanoic acid) S65
AC33: azelaic acid (nonanedioic acid) S66
AC34: citric acid (2-hydroxypropane-1,2,3-tricarboxylic acid) S68
AC35: 4-pentylbenzoic acid S70
AC36: lauric acid (dodecanoic acid) S71
AC37: trimesic acid (benzene-1,3,5-tricarboxylic acid) S72
AC38: oleic acid ((9Z)-octadec-9-enoic acid) S73

AC39: ricinoleic acid ((9Z,12R)-12-hydroxyoctadec-9-enoic acid) S75
AC40: arachidonic acid ((5Z,8Z,11Z,14Z)-icosa-5,8,11,14-tetraenoic acid) S76
AC41: 7,10-dihydroxy-8(E)-octadecenoic acid S77
S3.2 Aldehydes. ketones. Alcohols S78
AK1: formaldehyde (methanal) S78
AK2: methanol S79
AK3: acetaldehyde (ethanal) S81
AK4: ethanol S82
AK5: acetone (propan-2-one) S84
AK6: propan-1-ol S86
AK7: propan-2-ol S89
AK8: ethylene glycol (ethane-1,2-diol) S91
AK9: propylene glycol (propane-1,2-diol) S93
AK10: propan-1,3-diol S95
AK11: pentan-1-ol S97
AK12: 1,3-butanediol S98
AK13: 1,4-butanediol S99
AK14: glycerol (propane-1,2,3-triol) S100
AK15: phenol (benzenol) S102
AK16: hexan-1-ol S103
AK17: hexan-2-ol S105
AK18: 2,3-dimethylbutan-2-ol S106
AK19: 2-methylpentan-2-ol S107
AK20: 1,5-pentanediol S108
AK21: p-cresol (4-methylbenzenol) S109
AK22: heptan-1-ol S110
AK23: hexane-1,2-diol S112
AK24: hexane-1,6-diol S113
AK25: hexane-1,5-diol S114
AK26: hexane-2,5-diol S115
AK27: 4-ethylphenol S116
AK28: octan-1-ol S117
AK29: octan-2-ol S119

AK30: 4-propylphenol S120
AK31: 1-naphthol (naphthalen-1-ol) S121
AK32: 2-naphthol (naphthalen-2-ol) S122
AK33: nonan-1-ol S123
AK34: nonan-5-ol S124
AK35: 4-tert-butylphenol S125
AK36: 4-sec-butylphenol S126
AK37: 2,3-dihydroxynaphthalene S127
S3.3 Amines. sugars S128
SA1: colamine (2-aminoethan-1-ol) S128
SA2: pyrrolidine (prolamine) S130
SA3: glycine (aminoacetic acid) S131
SA4: threamine (1-aminopropan-2-ol) S133
SA5: 3-aminopropan-1-ol S134
SA6: 2-(methylamino)ethan-1-ol S135
SA7: piperidine (pyridine) S137
SA8: N.N′-dimethylethane-1,2-diamine S138
SA9: DL-alanine (2-aminopropanoic acid) S139
SA10: β-alanine (3-aminopropanoic acid) S141
SA11: 2-(ethylamino)ethan-1-ol S142
SA12: 2-amino-2-methylpropan-1-ol S143
SA13: 2-(dimethylamino)ethan-1-ol S144
SA14: cyclohexanamine S145
SA15: piperidic acid (4-aminobutanoic acid) S146
SA16: DL-2-aminobutanoic acid S147
SA17: 1-dimethylaminopropan-2-ol S148
SA18: diolamine (2,2′-azanediyldi(ethan-1-ol)) S149
SA19: 5-aminopentanoic acid S151
SA20: DL-norvaline (2-aminopentanoic acid) S152
SA21: methyl diethanolamine (2.2′-(methylazanediyl)di(ethan-1-ol)) S153
SA22: 2-amino-2-ethyl-1,3-propanediol S155
SA23: 2-amino-2-hydroxymethyl-propane-1,3-diol S156
SA24: erythritol ((2R,3S)-butane-1,2,3,4-tetrol) S157

SA25: 6-aminohexanoic acid S158

SA26: DL-norleucine (2-aminohexanoic acid) S159

SA27: L-leucine ((S)-2-amino-4-methylpentanoic acid) S160

SA28: hexamethylenetetramine (1,3,5,7-tetraazaadamantane) S162

SA29: trolamine (2,2′,2″-nitrilotri(ethan-1-ol)) S163

SA30: xylitol (meso-Xylitol) S164

SA31: levoglucosan ((1R,2S,3S,4R,5R)-6,8- dioxabicyclo[3.2.1]octane-2,3,4-triol) S165

SA32: L-phenylalanine S167

SA33: D-(+)-glucose ((2R,3S,4R,5R)-2,3,4,5,6-pentahydroxyhexanal) S168

SA34: D-(+)-galactose ((2R,3S,4S,5R)-2,3,4,5,6-pentahydroxyhexanal) S170

SA35: inositol (1,2,3,4,5,6-hexahydroxycyclohexane) S171

SA36: sorbitol ((2S,3R,4R,5R)-hexane-1,2,3,4,5,6-hexol) S172

SA37: D-(+)-maltose ((3R,4R,5S,6R)-6-(hydroxymethyl)-5-[[(2R,3R,4S,5S,6R)-3,4,5-trihydroxy-6-(hydroxymethyl)oxan-2-yl]oxy]oxane-2,3,4-triol) S173

SA38: sucrose (β-D-fructofuranosyl α-D-glucopyranoside) S174

S3.4 Synthetic and biological surfactants S175

SB1: SDS (sodium dodecyl sulfate) S175

SB2: DTAB (dodecyltrimethylammonium bromide) S177

SB3: CTAB (cetyltrimethylammonium bromide) S179

SB4: AOT (sodium bis(2-ethylhexyl)sulfosuccinate) S182

SB5: Triton X114 ((1,1,3,3-tetramethylbutyl)phenyl-polyethylene glycol) S184

SB6: Brij35 (polyoxyethylene lauryl ether) S186

SB7: mono-rhamnolipid S188

SB8: di-rhamnolipid S190

SB9: surfactin *from Bacillus subtilis* S192

SB10: syringafactin B/C *from Xanthomonas and Pseudomonas* S194

SB11: viscosin *from Pseudomonas* S195

S3.5 Macromolecules S197

MA1: SRFA (Suwannee river fulvic acid) S197

MA3: Humic acid S201

MA4: HULIS (Humic acid like substances) extracted from atmospheric aerosols S203

MA5: EPS (Extracellular polymeric substances) S206

S1. Concentration conversions used in the inventory

Table S1 : Physical quantity, symbols, units and equation used for the calculations

Physical quantity	Symbol	Unit	Equation	Equation number
**Mass**	m	kg		
**Density**	ρ	kg/L	$\rho = \frac{m}{V}$	(S1)
**Molecular mass**	M	g/mol		
**Amount of substance**	n	mol		
**Volume**	V	L	$V_{solution} \approx V_{water} + V_{mlc}$	(S2)
**Molar concentration**	C	mol/L (=M)	$C_{mlc} = \frac{n_{mlc}}{V_{solution}}$	(S3)
**Molality (Molar concentration per mass of solvent)**	b	mol/kg_w	$b_{mlc} = \frac{n_{mlc}}{m_{water}}$	(S4)
**Molar fraction**	x	No unit	$x_{mlc} = \frac{n_{mlc}}{n_{water} + n_{mlc}}$	(S5)
**Mass fraction**	xw	No unit	$xw_{mlc} = \frac{m_{mlc}}{m_{water} + m_{mlc}}$	(S6)
**Volume fraction**	%v/v	%, No unit	$\%v/v_{mlc} = \frac{V_{mlc}}{V_{solution}}$	(S7)
**Mass per volume fraction**	%m/v_w	%, g/L	$\%m/v = \frac{m_{mlc}}{V_{water}}$	(S8)
**Molar fraction per moles of water**	f	No unit	$f_{mlc} = \frac{n_{mlc}}{n_{water}}$	(S9)

mlc: molecule

S1.1: Molar concentration as a function of molality

From equations (S1), (S3) and (S4), the following equation is obtained:

$$C_{mlc} = \frac{b_{mlc}}{\frac{b_{mlc} * M_{mlc}}{\rho_{mlc}} + \frac{1}{\rho_{water}}} \quad \text{(S10)}$$

S1.2: Molar concentration as a function of molar fraction

From equations (S1), (S2), (S3) and (S5), the following equation is obtained:

$$C_{mlc} \approx \frac{1}{\frac{M_{mlc}}{\rho_{mlc}} + \left(\frac{1 - x_{mlc}}{x_{mlc}}\right) * \frac{M_{water}}{\rho_{water}}} \quad \text{(S11)}$$

S1.3: Molar fraction as a function of molar concentration

From equations (S1), (S2), (S3) and (S5), the following equation is obtained:

$$x_{mlc} \approx \frac{1}{\frac{\rho_{water}}{M_{water}}\left(\frac{1}{C_{mlc}} - \frac{M_{mlc}}{\rho_{mlc}}\right) + 1} \quad \text{(S12)}$$

S1.4: Molar concentration as a function of mass fraction

From equations (S1), (S2), (S3) and (S6), the following equation is obtained:

$$C_{mlc} \approx \frac{1}{M_{mlc}} * \frac{xw_{mlc} * \rho_{mlc} * \rho_{water}}{xw_{mlc} * \rho_{water} + \rho_{mlc} * (1 - xw_{mlc})} \quad \text{(S13)}$$

S1.5: Molar concentration as a function of volume fraction

From equations (S1), (S2), (S3) and (S7), the following equation is obtained:

$$C_{mlc} = \frac{\rho_{mlc}}{M_{mlc}} * \%\frac{\mathrm{v}}{\mathrm{v}} * 100 \quad \text{(S14)}$$

S1.6: Molar concentration as a function of mass per volume fraction

From equations (S1), (S2), (S3) and (S8), and assuming $V_{solution} \approx V_{water}$, the following equation is obtained:

$$C_{mlc} \approx \frac{1}{M_{mlc}} * \%\frac{\mathrm{m}}{\mathrm{v}_{water}} * 100 \quad \text{(S15)}$$

S1.7: Molar fraction as a function of molar fraction per moles of water

From equations (S5) and (S9), the following equation is obtained:

$$x_{mlc} = \frac{f_{mlc}}{1 + f_{mlc}} \quad \text{(S16)}$$

S2. Determination of recommended values for the surface tension isotherms

S2.1. Least-square fit to Sigmoid model

For the substances for which several experimental datasets were available in the literature, recommendations are made in this inventory for an isotherm curve representing at best all these datasets. This recommended curve was calculated by fitting the datasets to a sigmoid curve using least-square regressions. Sigmoid curves were chosen as best representative of adsorption isotherms, according to the model developed by *Kleinheins et al. 2023* (Phys. Chem. Chem. Phys., 2023, 25, 11055):

$$\sigma(x_{mlc}) = \sigma_w - (\sigma_w - \sigma_{pure})(10^{pd} + 1)\frac{x_{mlc}^d}{10^{pd} + x_{mlc}^d} \tag{S17}$$

With σ_w the surface tension of pure water, σ_{pure} the surface tension of the pure substance, p and d optimized parameters (position of the inflection and distance of the estimated CMC, critical micellar concentration, from the inflection point, respectively), x_{mlc} the molar fraction. For substances where σ_{pure} is not known, this value was determined by fitting together with the parameters p and d.

The non-linear least square regressions (RMSE, root mean squared error) and calculations of the 95 % confidence intervals were performed using the module "kmpfit" of the Python package "Kapteyn" (Terlouw, J. P. and Vogelaar, M. G. R., Kapteyn Astronomical Institute, Groningen, *Kapteyn Package, version 3.4*, dec **2014**, Available from \url{http://www.astro.rug.nl/software/kapteyn/).

The optimal values obtained for the parameters of the sigmoid curves in these calculations are given in Table S2 below.

The sigmoid curves then obtained are recommended as a best fit of the experimental datasets, but only within the concentration range covered by experimental data. The surface tension isotherms vs. the concentration were plotted by converting the molar fraction into concentration (equation S11).

S2.2 Optimized parameters obtained in the calculations

Table S2: Modelling parameters of the fitted surface tension isotherms (equation 17). Comp.: compound number, σ_w : surface tension of water at 25°C, σ_{pure}: surface tension of pure compound, $\Delta\sigma_{pure}$: error on σ_{pure} for a 95% confidence interval in case σ_{pure} was fitted, p and d, optimized parameters, Δp: error on p for a 95% confidence interval, Δd: error on d for a 95% confidence interval, RMSE: root mean squared error, x_{mlc} min and x_{mlc} max: minimum and maximum molar fraction of the experimental data representing the validated range of the fit.

Comp.	σ_w (mN/m)	σ_{pure} (mN/m)	$\Delta\sigma_{pure}$ (mN/m)	p	Δp	d	Δd	RMSE (mN/m)	x_{mlc} min	x_{mlc} max
**AC1**	72	37.5		-0.33	0.14	0.72	0.07	0.7	7.00E-04	1.00E+00
**AC2**	72	27.9		-1.05	0.04	0.74	0.04	0.7	1.00E-04	1.00E+00
**AC3**	72	26.2		-1.74	0.03	0.76	0.05	1.0	8.00E-04	1.00E+00
**AC4**	72	26.2		-2.28	0.02	1.18	0.08	1.4	4.00E-05	1.00E+00
**AC5**	72	64.8	2.7	-1.53	0.32	1.42	0.65	0.8	1.98E-05	1.28E-01
**AC6**	72	53.0		-1.15	0.06	1.33	0.27	1.8	1.80E-03	1.00E+00
**AC7**	72	26.7		-2.99	0.01	1.05	0.04	0.8	1.43E-05	3.49E-03
**AC8**	72	59.8	2.2	-1.33	0.27	0.91	0.22	0.5	1.73E-04	1.58E-01
**AC10**	72	54.0	4.6	-1.41	0.37	0.90	0.30	1.0	1.55E-04	1.08E-01
**AC11**	72	27.5		-3.64	0.04	1.20	0.12	2.7	1.80E-06	1.45E-03
**AC12**	72	66.2	1.3	-2.30	0.15	1.73	0.62	0.9	2.72E-05	4.81E-02
**AC15**	72	28.9		-4.16	0.02	1.28	0.09	1.2	8.80E-07	3.48E-04
**AC16**	72	51.4	1.9	-1.70	0.16	0.82	0.15	1.4	5.76E-05	2.39E-01
**AC17**	72	63.4	7.1	-0.87	1.78	0.68	0.39	0.4	1.34E-04	8.70E-02
AC22	*72*	*-176.7*	*236.8*	*11.31*	*416783<br>0.96*	*0.60*	*0.07*	*0.4*	*5.22E-06*	*2.80E-03*
**AC25**	72	27.9	1.9	-5.28	0.03	1.34	0.12	2.3	5.25E-08	9.01E-05
**AC29**	72	28.9	11.8	-5.74	0.18	1.51	0.34	1.6	1.79E-08	3.57E-06
**AC31**	72	50.6	11.4	-3.50	0.54	0.94	0.40	1.7	9.48E-08	8.30E-04
AC33	*72*	*10.9*	*96.8*	*-3.17*	*1.37*	*0.89*	*0.48*	*1.7*	*9.56E-08*	*4.82E-04*
**AC34**	72	65.2	1.7	-1.96	0.32	1.06	0.69	1.2	9.36E-05	1.21E-01
**AC38**	72	32.2	3.7	-7.00	0.21	0.87	0.30	6.1	3.33E-10	1.81E-04
**AK2**	72	23.5		-0.77	0.04	0.93	0.06	1.2	1.10E-02	1.00E+00
**AK4**	72	22.2		-1.27	0.02	0.88	0.05	1.1	8.00E-03	1.00E+00
**AK5**	72	23.5		-1.34	0.04	0.74	0.06	1.0	9.90E-03	1.00E+00
**AK6**	72	24.0		-1.94	0.02	1.28	0.10	2.5	5.00E-04	1.00E+00
**AK7**	72	23.5		-1.81	0.05	1.13	0.13	1.4	5.00E-03	1.00E+00
**AK8**	72	46.6		-0.55	0.07	0.76	0.05	0.4	8.40E-03	1.00E+00
**AK9**	72	35.9		-1.11	0.03	0.87	0.06	0.9	4.30E-03	1.00E+00
**AK10**	72	46.3		-0.86	0.25	0.68	0.17	1.6	4.00E-03	1.00E+00
**AK14**	72	63.0		19.83	344928<br>60.92	0.43	0.05	0.8	2.13E-02	1.00E+00
**AK16**	72	25.8	5.6	-3.64	0.02	1.29	0.08	1.0	6.30E-06	9.30E-04

**AK22**	72	26.6	11.8	-4.35	0.05	1.30	0.17	2.2	4.03E-07	2.60E-04
**AK28**	72	27.2		-4.55	0.06	1.73	0.43	4.5	1.46E-06	1.00E-04
**SA1**	72	48.5		0.00	0.70	0.53	0.14	0.7	1.50E-02	1.00E+00
**SA3**	72	72.1	0.0	-0.53	0.00	4.57	0.00	0.1	1.75E-04	2.74E-03
**SA6**	72	35.3		-0.70	0.17	0.69	0.11	0.8	1.25E-02	1.00E+00
**SA9**	72	71.7	0.3	-1.24	0.76	11.55	149.72	0.2	1.76E-04	1.52E-01
**SA18**	72	47.2		-0.15	1.19	0.52	0.23	1.2	1.87E-02	1.00E+00
**SA21**	72	38.2		-1.05	0.17	0.60	0.11	1.3	8.00E-03	1.00E+00
SA27	*72*	*42.5*	*208.0*	*-1.87*	*3.66*	*1.12*	*0.73*	*0.5*	*1.80E-04*	*2.86E-03*
**SA31**	72	70.0	1.3	-1.90	0.55	2.75	6.50	0.8	1.07E-07	1.99E-01
**SA33**	72	77.7	3.5	-1.29	0.31	2.11	1.19	0.4	1.87E-04	8.84E-02
**SB1**	72	34.9	1.7	-4.26	0.04	1.65	0.22	2.3	1.80E-10	1.77E-03
**SB2**	72	37.0	1.1	-4.05	0.03	2.08	0.23	1.3	2.70E-06	9.13E-04
**SB3**	72	33.9	1.4	-5.29	0.04	1.40	0.12	1.6	1.79E-10	1.31E-04
**SB4**	72	25.8	2.6	-5.08	0.09	0.82	0.15	3.8	1.80E-09	6.36E-04
**SB5**	72	27.6	2.1	-6.77	0.14	0.62	0.12	2.9	1.81E-10	2.17E-04
**SB6**	72	43.2	1.8	-6.75	0.12	0.88	0.22	2.8	1.80E-09	3.80E-04
**SB7**	72	16.7	8.1	-6.65	0.29	0.56	0.12	3.0	7.20E-12	7.85E-06
**SB8**	72	25.8	4.7	-6.76	0.17	0.68	0.16	3.0	5.57E-12	1.05E-05
**SB9**	72	30.3	6.7	-7.20	0.21	1.27	0.68	8.3	8.68E-09	3.04E-06
**SB11**	72	27.0	5.6	-6.97	0.16	1.51	0.77	8.1	1.60E-08	2.76E-05
**MA1**	72	36.1	5.8	-3.96	0.25	0.78	0.20	2.4	1.58E-07	3.36E-03
**MA2**	72	50.1	5.6	-5.13	0.23	1.26	0.42	1.0	3.19E-07	2.63E-05
**MA3**	72	51.5	11.7	-4.06	0.92	0.74	0.67	5.5	7.96E-08	1.61E-03
MA4	*72*	*-45.2*	*1144.3*	*-2.29*	*25.78*	*0.33*	*0.71*	*5.7*	*1.42E-06*	*1.09E-04*
**MA5**	72	54.1	7.1	-7.59	0.59	0.79	0.44	2.3	3.84E-11	2.28E-07

Note: For data *in italic*, the modelling is reliable only in the range of the experimental data. Therefore, the modelling should be used only in the experimental data range (*i.e.* between x_{mlc} min and x_{mlc} max)

S3. Adsorption isotherm data and recommendations

In this review, for each molecule, the original concentrations, the molar concentrations in mol L^{-1} and the molar fractions are given.

Original concentration data are given in **bold**.

The temperature used in the cited studies was reported at 20-25°C (except if specified otherwise).

The uncertainties on the data from literature are ≤ 1% and ≤ 0.2 mN/m for the surface tensions.

The densities and molecular masses used for the calculations are given in Tables 1 to 5 in the main article.

The references of the cited articles can be found in the main article.

S3.1. Organic acids

AC1: formic acid (methanoic acid)

Alvarez et al. 1997 **Table**			Granados et al. 2006 **Table**			***Recommended***		
**C (M)**	**Molar fraction**	**σ (mN/m)**	**C (M)**	**Molar fraction**	**σ (mN/m)**	***C (M)***	***Molar fraction***	***σ (mN/m)***
0.00E-00	**0.00E-00**	72.01	3.89E-02	**7.00E-04**	71.48	*3.89E-02*	*7.00E-04*	*71.5*
1.09E+00	**2.00E-02**	68.11	8.32E-02	**1.50E-03**	71.14	*5.69E-02*	*1.03E-03*	*71.3*
2.23E+00	**4.20E-02**	64.82	1.55E-01	**2.80E-03**	70.65	*8.34E-02*	*1.50E-03*	*71.1*
3.37E+00	**6.50E-02**	61.91	3.70E-01	**6.70E-03**	69.45	*1.22E-01*	*2.20E-03*	*70.9*
4.50E+00	**8.90E-02**	59.33	5.82E-01	**1.06E-02**	68.59	*1.79E-01*	*3.23E-03*	*70.5*
5.67E+00	**1.15E-01**	57.07	8.41E-01	**1.54E-02**	67.53	*2.62E-01*	*4.74E-03*	*70.1*
6.91E+00	**1.44E-01**	55.21	1.49E+00	**2.77E-02**	65.46	*3.83E-01*	*6.94E-03*	*69.5*
9.37E+00	**2.07E-01**	51.68	2.34E+00	**4.42E-02**	63.13	*5.59E-01*	*1.02E-02*	*68.7*
1.19E+01	**2.81E-01**	48.88	3.87E+00	**7.55E-02**	59.92	*8.15E-01*	*1.49E-02*	*67.8*
1.46E+01	**3.70E-01**	46.18	5.44E+00	**1.10E-01**	57.29	*1.19E+00*	*2.19E-02*	*66.6*
1.74E+01	**4.77E-01**	43.73	7.32E+00	**1.54E-01**	55.23	*1.72E+00*	*3.20E-02*	*65.1*
2.03E+01	**6.10E-01**	41.31	9.26E+00	**2.04E-01**	52.74	*2.48E+00*	*4.69E-02*	*63.2*
2.33E+01	**7.79E-01**	39.14	1.26E+01	**3.00E-01**	49.18	*3.55E+00*	*6.88E-02*	*61.0*
2.65E+01	**1.00E+00**	37.03	1.59E+01	**4.18E-01**	47.25	*5.05E+00*	*1.01E-01*	*58.4*
			1.82E+01	**5.10E-01**	45.73	*7.07E+00*	*1.48E-01*	*55.4*
			2.04E+01	**6.13E-01**	43.41	*9.73E+00*	*2.17E-01*	*52.1*
			2.21E+01	**7.06E-01**	41.42	*1.31E+01*	*3.18E-01*	*48.5*
			2.39E+01	**8.16E-01**	40.12	*1.71E+01*	*4.65E-01*	*44.8*
			2.51E+01	**8.97E-01**	39.13	*2.17E+01*	*6.82E-01*	*41.0*
			2.65E+01	**1.00E+00**	38.17	*2.65E+01*	*1.00E+00*	*37.5*

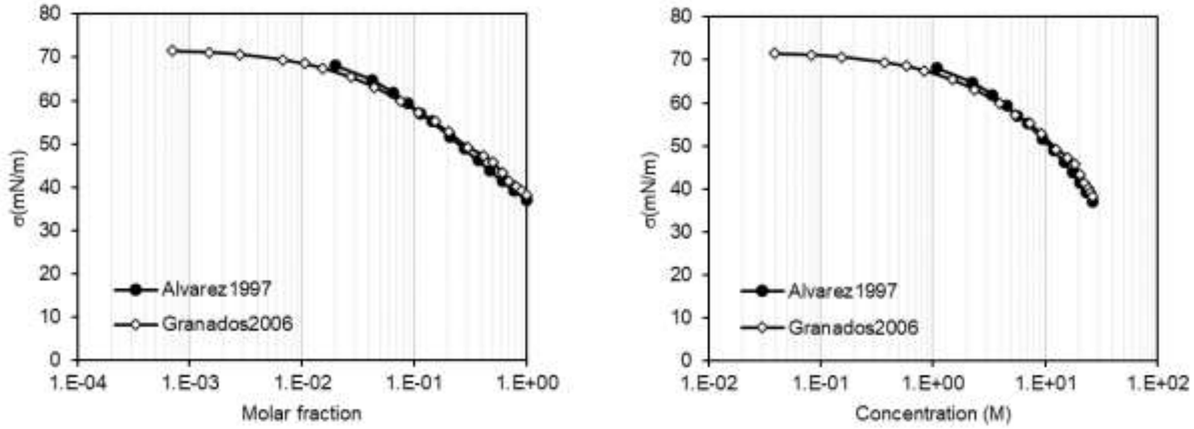

Fig. S1 (a): Comparison of the experimental data for formic acid / water mixtures.

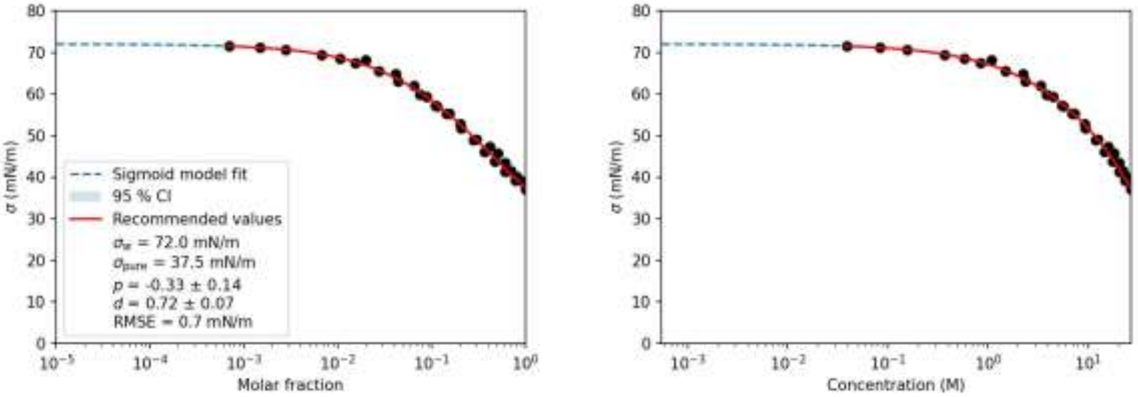

Fig. S1 (b): Surface tension fit with the Sigmoid model (*Kleinheins et al. 2023*) for formic acid / water mixtures. Solid red line: model fit inside the concentration range covered by experimental data, reported as recommended values. Blue shading: fit parameters with 95 % confidence interval (CI). RMSE: root mean squared error. Markers: data used for fitting.

Comment: N/A.

AC2: acetic acid (ethanoic acid)

Alvarez et al. 1997 **Table**			Granados et al. 2006 **Table**			***Recommended***		
**C (M)**	**Molar fraction**	**σ (mN/m)**	**C (M)**	**Molar fraction**	**σ (mN/m)**	***C (M)***	***Molar fraction***	***σ (mN/m)***
0.00E+00	**0.00E+00**	72.01	5.55E-03	**1.00E-04**	71.79	*5.55E-03*	*1.00E-04*	*71.7*
8.59E-01	**1.60E-02**	61.72	2.77E-02	**5.00E-04**	71.18	*9.02E-03*	*1.62E-04*	*71.5*
1.66E+00	**3.20E-02**	55.58	3.88E-02	**7.00E-04**	71.04	*1.46E-02*	*2.64E-04*	*71.3*
2.50E+00	**5.00E-02**	51.24	4.44E-02	**8.00E-04**	70.84	*2.38E-02*	*4.28E-04*	*71.0*
3.37E+00	**7.00E-02**	48.01	6.65E-02	**1.20E-03**	70.37	*3.86E-02*	*6.95E-04*	*70.6*
4.21E+00	**9.10E-02**	45.44	3.29E-01	**6.00E-03**	66.17	*6.26E-02*	*1.13E-03*	*70.0*
5.07E+00	**1.14E-01**	43.46	3.61E-01	**6.60E-03**	65.76	*1.01E-01*	*1.83E-03*	*69.2*
6.79E+00	**1.67E-01**	40.34	6.44E-01	**1.19E-02**	62.57	*1.64E-01*	*2.98E-03*	*68.1*
8.52E+00	**2.31E-01**	37.62	7.44E-01	**1.38E-02**	61.57	*2.66E-01*	*4.83E-03*	*66.6*
1.03E+01	**3.10E-01**	35.79	1.24E+00	**2.34E-02**	58.26	*4.29E-01*	*7.85E-03*	*64.6*
1.20E+01	**4.12E-01**	33.82	1.35E+00	**2.57E-02**	57.45	*6.89E-01*	*1.27E-02*	*62.0*
1.38E+01	**5.45E-01**	32.03	2.06E+00	**4.04E-02**	53.14	*1.10E+00*	*2.07E-02*	*58.8*
1.56E+01	**7.30E-01**	29.76	2.58E+00	**5.16E-02**	50.64	*1.74E+00*	*3.36E-02*	*55.1*
1.74E+01	**1.00E+00**	27.12	3.56E+00	**7.46E-02**	47.25	*2.71E+00*	*5.46E-02*	*50.8*
			4.87E+00	**1.09E-01**	43.43	*4.12E+00*	*8.86E-02*	*46.2*
			4.95E+00	**1.11E-01**	43.39	*6.08E+00*	*1.44E-01*	*41.7*
			6.30E+00	**1.51E-01**	40.12	*8.58E+00*	*2.34E-01*	*37.4*
			7.21E+00	**1.81E-01**	39.24	*1.15E+01*	*3.79E-01*	*33.6*
			8.47E+00	**2.29E-01**	38.17	*1.46E+01*	*6.16E-01*	*30.4*
			1.02E+01	**3.05E-01**	36.50	*1.74E+01*	*1.00E+00*	*27.9*
			1.20E+01	**4.13E-01**	34.80			
			1.35E+01	**5.16E-01**	33.23			
			1.45E+01	**6.07E-01**	31.87			
			1.54E+01	**7.04E-01**	30.32			
			1.62E+01	**8.07E-01**	29.32			
			1.69E+01	**9.08E-01**	28.42			
			1.74E+01	**1.00E+00**	27.08			

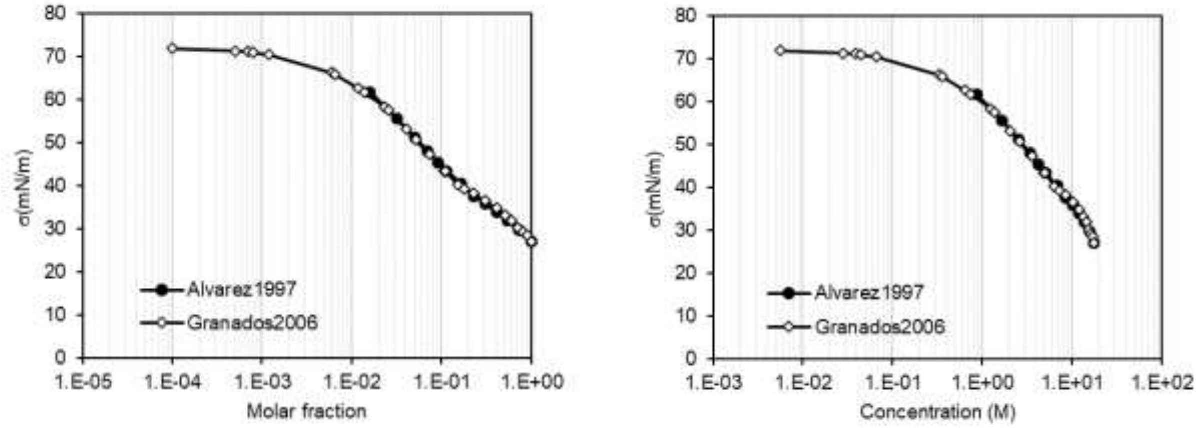

Fig. S2 (a): Comparison of the experimental surface tension data for acetic acid / water mixtures.

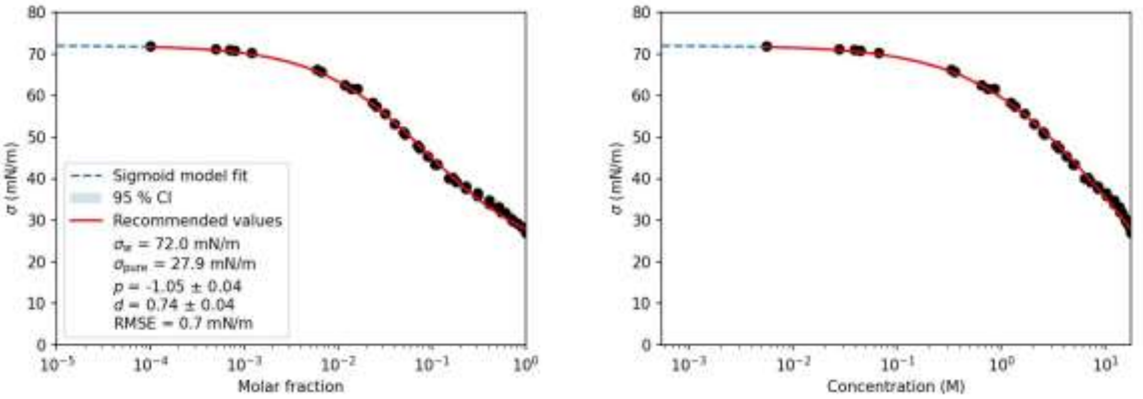

Fig. S2 (b): Surface tension fit with the Sigmoid model (*Kleinheins et al. 2023*) for acetic acid / water mixtures. Solid red line: model fit inside the concentration range covered by experimental data, reported as recommended values. Blue shading: fit parameters with 95 % confidence interval (CI). RMSE: root mean squared error. Markers: data used for fitting.

Comment: N/A

AC3: propionic acid (propanoic acid)

Alvarez et al. 1997 **Table**			Granados et al. 2006 **Table**			Suarez et al. 2011 **Table**		
**C (M)**	**Molar fraction**	**σ (mN/m)**	**C (M)**	**Molar fraction**	**σ (mN/m)**	**C (M)**	**Molar fraction**	**σ (mN/m)**
0.00E+00	**0.00E+00**	72.01	4.43E-02	**8.00E-04**	68.52	0.00E+00	**0.00E+00**	71.81
6.94E-01	**1.30E-02**	52.44	8.84E-02	**1.60E-03**	66.47	4.66E-01	**8.61E-03**	52.32
1.33E+00	**2.60E-02**	44.62	1.60E-01	**2.90E-03**	63.66	7.74E-01	**1.46E-02**	49.33
2.02E+00	**4.10E-02**	40.44	2.41E-01	**4.40E-03**	60.98	1.08E+00	**2.08E-02**	46.22
2.68E+00	**5.70E-02**	37.76	3.48E-01	**6.40E-03**	57.91	1.65E+00	**3.27E-02**	41.92
3.37E+00	**7.50E-02**	35.92	5.54E-01	**1.03E-02**	53.54	2.21E+00	**4.55E-02**	39.50
4.02E+00	**9.40E-02**	33.63	9.41E-01	**1.79E-02**	48.16	2.58E+00	**5.44E-02**	37.77
5.39E+00	**1.40E-01**	32.76	1.39E+00	**2.72E-02**	44.12	3.03E+00	**6.60E-02**	35.73
6.72E+00	**1.96E-01**	31.47	2.27E+00	**4.70E-02**	38.89	3.36E+00	**7.47E-02**	35.18
8.04E+00	**2.67E-01**	30.43	2.87E+00	**6.18E-02**	36.93	3.79E+00	**8.69E-02**	34.71
9.37E+00	**3.62E-01**	29.52	4.18E+00	**9.89E-02**	34.71	4.52E+00	**1.09E-01**	34.04
1.07E+01	**4.93E-01**	28.58	6.84E+00	**2.02E-01**	32.25	5.06E+00	**1.28E-01**	33.65
1.20E+01	**6.86E-01**	27.51	8.56E+00	**3.01E-01**	31.12	5.64E+00	**1.49E-01**	32.97
1.33E+01	**1.00E+00**	26.17	9.81E+00	**4.00E-01**	30.19	6.16E+00	**1.71E-01**	32.76
			1.08E+01	**5.01E-01**	29.36	6.42E+00	**1.82E-01**	32.51
			1.15E+01	**6.04E-01**	28.56	1.33E+01	**1.00E+00**	26.19
			1.21E+01	**7.02E-01**	27.91			
			1.24E+01	**7.67E-01**	27.52			
			1.29E+01	**8.78E-01**	26.92			
			1.33E+01	**1.00E+00**	26.15			

Recommended		
C (M)	***Molar fraction***	***σ (mN/m)***
4.43E-02	*8.00E-04*	*67.9*
6.44E-02	*1.16E-03*	*66.7*
9.36E-02	*1.69E-03*	*65.2*
1.36E-01	*2.47E-03*	*63.4*
1.97E-01	*3.59E-03*	*61.2*
2.86E-01	*5.22E-03*	*58.6*
4.13E-01	*7.60E-03*	*55.7*
5.94E-01	*1.11E-02*	*52.5*
8.51E-01	*1.61E-02*	*49.1*
1.21E+00	*2.34E-02*	*45.7*
1.71E+00	*3.41E-02*	*42.4*
2.38E+00	*4.97E-02*	*39.2*
3.27E+00	*7.23E-02*	*36.4*
4.38E+00	*1.05E-01*	*34.0*
5.73E+00	*1.53E-01*	*31.9*
7.26E+00	*2.23E-01*	*30.2*
8.89E+00	*3.24E-01*	*28.8*
1.05E+01	*4.72E-01*	*27.7*
1.20E+01	*6.87E-01*	*26.8*
1.33E+01	*1.00E+00*	*26.2*

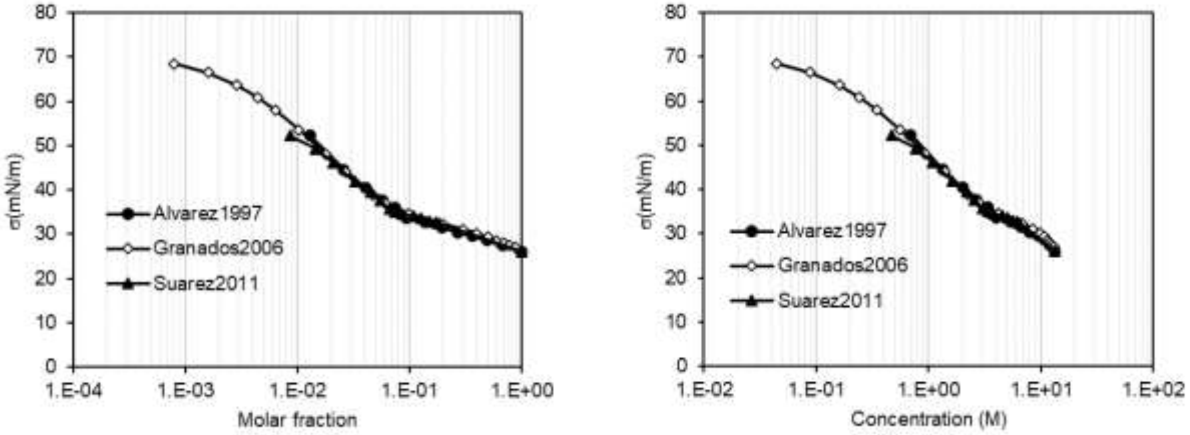

Fig. S3 (a): Comparison of the experimental surface tension data for propionic acid / water mixtures.

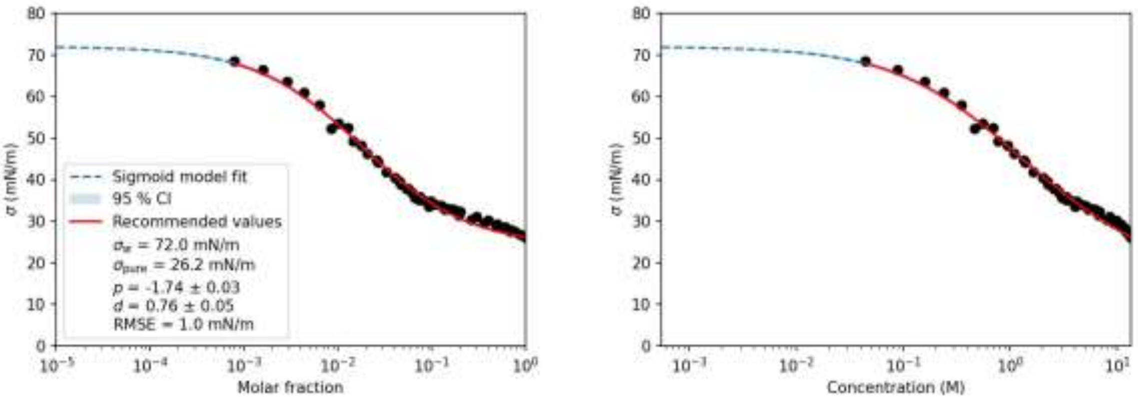

Fig. S3 (b): Surface tension fit with the Sigmoid model (*Kleinheins et al. 2023*) for propionic acid / water mixtures. Solid red line: model fit inside the concentration range covered by experimental data, reported as recommended values. Blue shading: fit parameters with 95 % confidence interval (CI). RMSE: root mean squared error. Markers: data used for fitting.

Comment: N/A

AC4: butyric acid (butanoic acid)

Donaldson et al. 1999 **Graph (experimental points)**			Granados et al. 2006 **Table**			Suarez et al. 2011 **Table**		
**C (M)**	**Molar fraction**	**σ (mN/m)**	**C (M)**	**Molar fraction**	**σ (mN/m)**	**C (M)**	**Molar fraction**	**σ (mN/m)**
**0.00E+00**	0.00E+00	72.0	2.22E-03	**4.00E-05**	71.12	0.00E+00	**0.00E+00**	71.81
**1.09E-01**	1.98E-03	62.8	5.55E-03	**1.00E-04**	70.47	2.59E-01	**4.75E-03**	50.45
**1.09E-01**	1.98E-03	61.5	3.33E-02	**6.00E-04**	66.32	3.54E-01	**6.55E-03**	44.39
**1.09E-01**	1.98E-03	58.9	8.83E-02	**1.60E-03**	60.32	4.84E-01	**9.03E-03**	41.18
**2.16E-01**	3.95E-03	56.2	1.54E-01	**2.80E-03**	55.12	5.32E-01	**9.98E-03**	39.94
**2.16E-01**	3.95E-03	55.0	3.04E-01	**5.60E-03**	46.34	6.72E-01	**1.27E-02**	37.14
**2.16E-01**	3.95E-03	53.6	4.04E-01	**7.50E-03**	42.12	7.51E-01	**1.43E-02**	35.57
**3.20E-01**	5.91E-03	49.7	7.15E-01	**1.36E-02**	35.88	8.61E-01	**1.66E-02**	33.96
**3.20E-01**	5.91E-03	49.1	1.03E+00	**2.01E-02**	31.94	1.63E+00	**3.35E-02**	28.85
**4.24E-01**	7.88E-03	45.1	1.48E+00	**2.99E-02**	29.24	2.12E+00	**4.52E-02**	28.46
**4.24E-01**	7.88E-03	44.4	2.22E+00	**4.80E-02**	28.48	2.61E+00	**5.83E-02**	28.32
**4.24E-01**	7.88E-03	43.2	2.66E+00	**5.96E-02**	28.35	3.56E+00	**8.72E-02**	28.25
**5.25E-01**	9.83E-03	43.2	3.54E+00	**8.65E-02**	28.23	3.92E+00	**9.95E-02**	28.25
**5.25E-01**	9.83E-03	41.9	4.02E+00	**1.03E-01**	28.15	4.32E+00	**1.15E-01**	28.20
**6.23E-01**	1.18E-02	40.5	6.14E+00	**2.03E-01**	27.92	4.79E+00	**1.34E-01**	28.12
**6.23E-01**	1.18E-02	39.9	7.43E+00	**3.00E-01**	27.73	4.99E+00	**1.43E-01**	28.09
**7.20E-01**	1.37E-02	38.0	8.22E+00	**3.81E-01**	27.66	5.67E+00	**1.77E-01**	28.01
**8.15E-01**	1.56E-02	36.7	9.08E+00	**5.04E-01**	27.56	1.08E+01	**1.00E+00**	26.21
			9.46E+00	**5.76E-01**	27.42			
			1.00E+01	**7.04E-01**	27.13			
			1.03E+01	**8.02E-01**	26.90			
			1.06E+01	**9.19E-01**	26.51			
			1.08E+01	**1.00E+00**	26.19			

Recommended		
C (M)	***Molar fraction***	***σ (mN/m)***
2.22E-03	*4.00E-05*	*71.9*
3.79E-03	*6.82E-05*	*71.7*
6.45E-03	*1.16E-04*	*71.5*
1.10E-02	*1.98E-04*	*71.1*
1.87E-02	*3.37E-04*	*70.3*
3.18E-02	*5.75E-04*	*68.9*
5.42E-02	*9.79E-04*	*66.5*
9.21E-02	*1.67E-03*	*62.6*
1.56E-01	*2.84E-03*	*57.1*
2.64E-01	*4.85E-03*	*50.2*
4.44E-01	*8.26E-03*	*43.1*
7.39E-01	*1.41E-02*	*37.0*
1.21E+00	*2.40E-02*	*32.6*
1.94E+00	*4.08E-02*	*29.8*
3.00E+00	*6.96E-02*	*28.1*
4.42E+00	*1.19E-01*	*27.2*
6.12E+00	*2.02E-01*	*26.7*
7.89E+00	*3.44E-01*	*26.4*
9.51E+00	*5.87E-01*	*26.2*
1.08E+01	*1.00E+00*	*26.2*

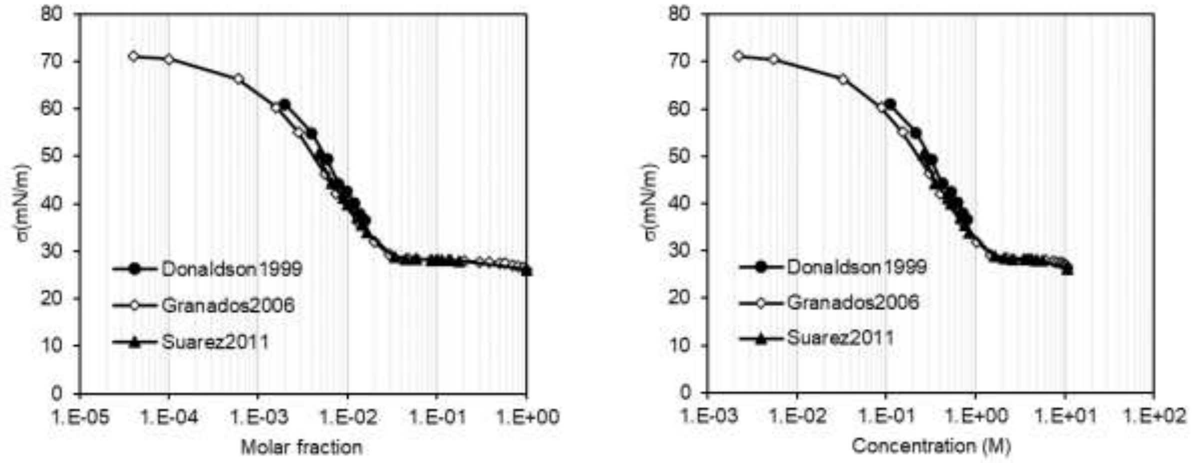

Fig. S4 (a): Comparison of the experimental surface tension data for butyric acid / water mixtures.

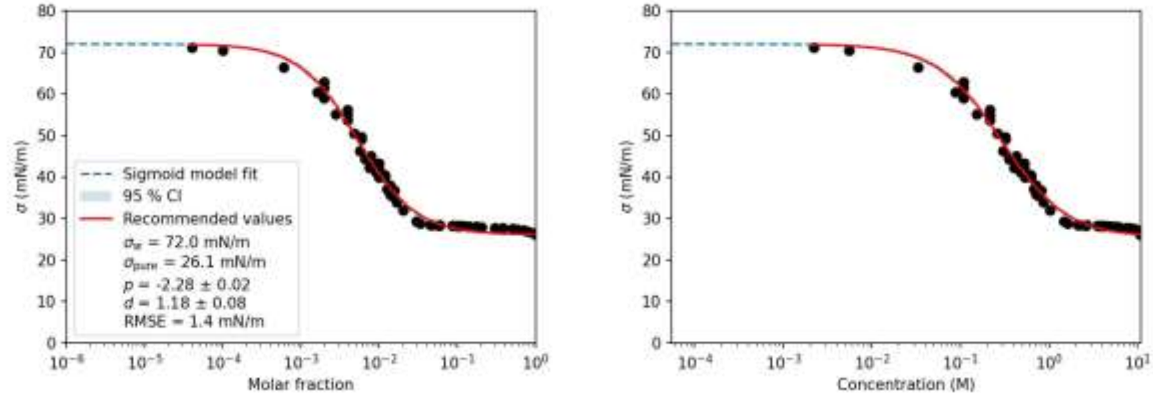

Fig. S4 (b): Surface tension fit with the Sigmoid model (*Kleinheins et al. 2023*) for butyric acid / water mixtures. Solid red line: model fit inside the concentration range covered by experimental data, reported as recommended values. Blue shading: fit parameters with 95 % confidence interval (CI). RMSE: root mean squared error. Markers: data used for fitting.

Comment: N/A

AC5: oxalic acid (ethanedioic acid)

Hyvarinen et al. 2006 **Table**			Topping et al. 2007 **Graph (experimental points)**				Varga et al. 2007 **Table**			
**C (M)**	**Molar fraction**	**σ (mN/m)**	**C (M)**	**Molar fraction**	**Mass fraction**	**σ (mN/m)**	**C (M)**	**Molar fraction**	**Mass fraction (%)**	**σ (mN/m)**
9.42E-02	**1.70E-03**	71.6	3.34E-03	6.02E-05	**3.01E-04**	72.0	5.57E-02	1.00E-03	**0.5**	71
1.88E-01	**3.40E-03**	71.4	1.17E-02	2.10E-04	**1.05E-03**	71.5	1.12E-01	2.02E-03	**1**	71
2.76E-01	**5.00E-03**	71.2	2.26E-02	4.07E-04	**2.03E-03**	71.3	5.69E-01	1.04E-02	**5**	71
3.68E-01	**6.70E-03**	71.0	3.44E-02	6.19E-04	**3.09E-03**	71.4				
4.55E-01	**8.30E-03**	70.7	6.20E-02	1.12E-03	**5.57E-03**	70.6				
5.36E-01	**9.80E-03**	70.7	1.24E-01	2.24E-03	**1.11E-02**	71.5				
6.27E-01	**1.15E-02**	70.5	3.08E-01	5.60E-03	**2.74E-02**	72.1				
6.59E-01	**1.21E-02**	70.7	3.69E-01	6.71E-03	**3.27E-02**	71.5				
7.13E-01	**1.31E-02**	70.3	7.31E-01	1.34E-02	**6.38E-02**	71.1				
7.92E-01	**1.46E-02**	70.1	1.11E+00	2.06E-02	**9.53E-02**	70.4				

Mahiuddin et al. 2008 **Graph (experimental points)**			Booth et al. 2009 **Table**			Aumann et al. 2010 **Graph (experimental points)**		
**C (M)**	**Molar fraction**	**σ (mN/m)**	**C (M)**	**Molar fraction**	**σ (mN/m)**	**C (M)**	**Molar fraction**	**σ (mN/m)**
**0.00E+00**	0.00E+00	72.0	2.21E-01	**4.00E-03**	70.3	**2.30E-03**	4.14E-05	72.0
**2.00E-02**	3.60E-04	71.7	5.47E-01	**1.00E-02**	68.6	**4.60E-03**	8.28E-05	72.0
**6.00E-02**	1.08E-03	72.1	2.23E+00	**4.30E-02**	66.5	**9.10E-03**	1.64E-04	71.9
**1.60E-01**	2.89E-03	71.9	5.88E+00	**1.28E-01**	65.8	**1.86E-02**	3.35E-04	71.9
**2.00E-01**	3.62E-03	71.9				**4.55E-02**	8.20E-04	71.9
**2.60E-01**	4.72E-03	71.9				**1.14E-01**	2.06E-03	71.7
**3.70E-01**	6.73E-03	71.7				**2.32E-01**	4.21E-03	71.5
**4.70E-01**	8.58E-03	71.6				**4.61E-01**	8.41E-03	71.1
**7.30E-01**	1.34E-02	71.3				**6.55E-01**	1.20E-02	70.8
**8.00E-01**	1.47E-02	71.1				**9.22E-01**	1.71E-02	70.3

Lee et al. 2014 **Table**			El Haber et al. 2023 **Table**			***Recommended***		
**C (M)**	**Molar fraction**	**σ (mN/m)**	**C (M)**	**Molar fraction**	**σ (mN/m)**	***C (M)***	***Molar fraction***	***σ (mN/m)***
**5.00E-02**	9.01E-04	72.4	**0.00E+00**	0.00E+00	73.87	*1.10E-03*	*1.98E-05*	*72.0*
**1.00E-01**	1.81E-03	72.4	**1.10E-03**	1.98E-05	73.25	*1.75E-03*	*3.14E-05*	*72.0*
**3.00E-01**	5.45E-03	71.6	**5.80E-03**	1.04E-04	73.54	*2.77E-03*	*4.99E-05*	*72.0*
**5.00E-01**	9.13E-03	71.1	**1.00E-02**	1.80E-04	72.98	*4.40E-03*	*7.91E-05*	*72.0*
			**1.90E-02**	3.42E-04	73.53	*6.97E-03*	*1.26E-04*	*72.0*
			**2.60E-02**	4.68E-04	72.92	*1.11E-02*	*1.99E-04*	*72.0*
			**3.70E-02**	6.67E-04	72.88	*1.76E-02*	*3.16E-04*	*72.0*
			**5.70E-02**	1.03E-03	72.71	*2.79E-02*	*5.02E-04*	*72.0*
			**7.60E-02**	1.37E-03	72.94	*4.42E-02*	*7.96E-04*	*72.0*
			**1.20E-01**	2.17E-03	71.63	*7.01E-02*	*1.26E-03*	*71.9*
			**1.90E-01**	3.44E-03	72.00	*1.11E-01*	*2.01E-03*	*71.8*
			**3.80E-01**	6.92E-03	70.09	*1.76E-01*	*3.18E-03*	*71.7*
			**5.70E-01**	1.04E-02	69.78	*2.78E-01*	*5.05E-03*	*71.5*
			**7.60E-01**	1.40E-02	68.59	*4.40E-01*	*8.01E-03*	*71.0*

	**9.20E-01**	1.70E-02	68.47	*6.92E-01*	*1.27E-02*	*70.3*
				1.09E+00	*2.02E-02*	*69.3*
				1.69E+00	*3.20E-02*	*68.2*
				2.61E+00	*5.08E-02*	*67.0*
				3.96E+00	*8.07E-02*	*66.1*
				5.88E+00	*1.28E-01*	*65.5*

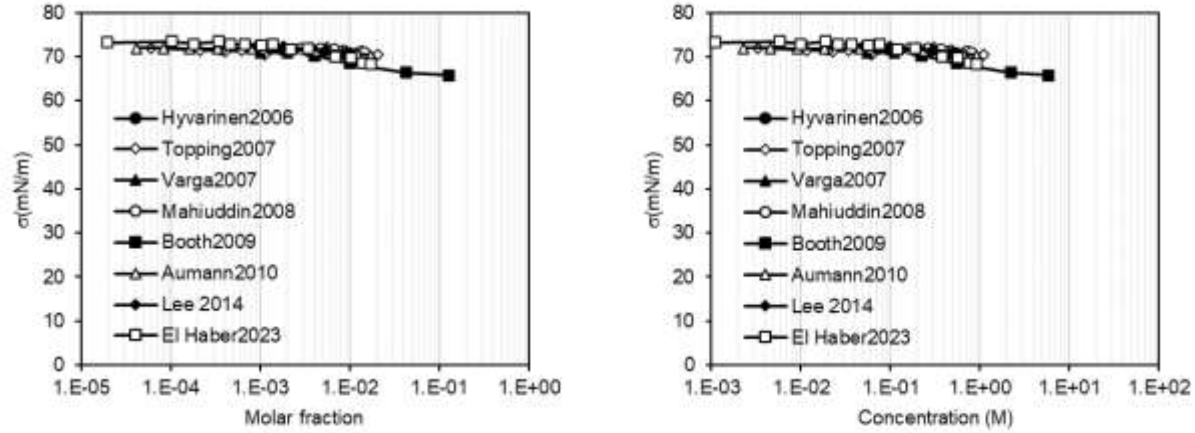

Fig. S5 (a): Comparison of the experimental surface tension data for oxalic acid / water mixtures.

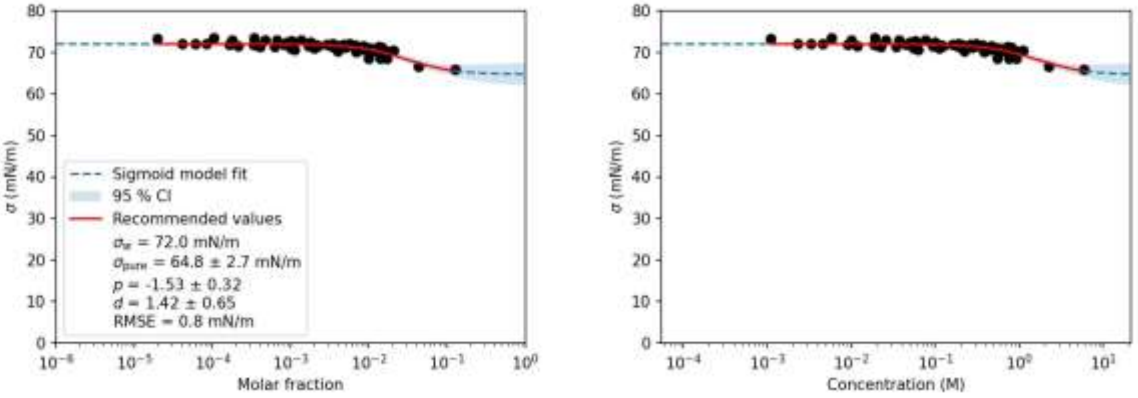

Fig. S5 (b): Surface tension fit with the Sigmoid model (*Kleinheins et al. 2023*) for oxalic acid / water mixtures. Solid red line: model fit inside the concentration range covered by experimental data, reported as recommended values. Blue shading: fit parameters with 95 % confidence interval (CI). RMSE: root mean squared error. Markers: data used for fitting.

Comment: N/A

AC6: methanesulfonic acid

Hoppel et al. 1987 **Table**		
**C (M)**	**Molar fraction**	**σ (mN/m)**
9.95E-02	**1.80E-03**	71.8
1.98E-01	**3.60E-03**	71.3
2.96E-01	**5.40E-03**	70.8
3.87E-01	**7.10E-03**	70.4
4.83E-01	**8.90E-03**	69.9
6.67E-01	**1.24E-02**	69.1
9.40E-01	**1.77E-02**	67.9
1.37E+00	**2.63E-02**	66.3
1.77E+00	**3.48E-02**	64.8
2.15E+00	**4.31E-02**	63.6
2.51E+00	**5.12E-02**	62.6
2.85E+00	**5.93E-02**	61.7
3.18E+00	**6.72E-02**	60.9
3.78E+00	**8.26E-02**	59.6
4.32E+00	**9.75E-02**	58.5
4.81E+00	**1.12E-01**	57.7
5.27E+00	**1.26E-01**	57.1
5.68E+00	**1.39E-01**	56.5
6.07E+00	**1.53E-01**	56.1
6.75E+00	**1.78E-01**	55.4
7.34E+00	**2.01E-01**	54.9
7.85E+00	**2.24E-01**	54.5
8.07E+00	**2.34E-01**	54.2
8.70E+00	**2.65E-01**	54.0
9.53E+00	**3.10E-01**	53.6
1.02E+01	**3.51E-01**	53.4
1.07E+01	**3.87E-01**	53.2
1.11E+01	**4.19E-01**	53.1

Allen et al. 2001 **Graph (experimental points)**		
**C (M)**	**Molar fraction**	**σ (mN/m)**
0.00E+00	**0.00E+00**	71.5
1.06E+00	**2.00E-02**	70.0
1.78E+00	**3.50E-02**	69.0
2.01E+00	**4.00E-02**	67.5
2.46E+00	**5.00E-02**	67.0
3.09E+00	**6.50E-02**	65.0
4.41E+00	**1.00E-01**	62.5
5.99E+00	**1.50E-01**	60.5
7.30E+00	**2.00E-01**	59.0
9.35E+00	**3.00E-01**	56.0
1.09E+01	**4.00E-01**	55.5
1.30E+01	**6.00E-01**	54.5
1.44E+01	**8.00E-01**	54.0
1.54E+01	**1.00E+00**	53.0

Lund Myhre et al. 2004 **Table**			
**C (M)**	**Molar fraction**	**Mass fraction**	**σ (mN/m)**
1.04E-01	1.89E-03	**1.00E-02**	71.72
5.29E-01	9.76E-03	**5.00E-02**	71.09
1.08E+00	2.04E-02	**1.00E-01**	70.16
2.83E+00	5.88E-02	**2.50E-01**	67.59
6.21E+00	1.58E-01	**5.00E-01**	61.59
9.43E+00	3.04E-01	**7.00E-01**	59.00
1.52E+01	9.49E-01	**9.90E-01**	54.52

Recommended		
C (M)	***Molar fraction***	***σ (mN/m)***
9.95E-02	*1.80E-03*	*71.9*
1.39E-01	*2.51E-03*	*71.8*
1.93E-01	*3.50E-03*	*71.6*
2.68E-01	*4.88E-03*	*71.5*
3.72E-01	*6.81E-03*	*71.2*
5.15E-01	*9.50E-03*	*70.7*
7.11E-01	*1.32E-02*	*70.1*
9.79E-01	*1.85E-02*	*69.2*
1.34E+00	*2.58E-02*	*68.0*
1.83E+00	*3.59E-02*	*66.4*
2.46E+00	*5.01E-02*	*64.5*
3.28E+00	*6.99E-02*	*62.4*
4.32E+00	*9.75E-02*	*60.2*

5.58E+00	*1.36E-01*	*58.3*
7.05E+00	*1.90E-01*	*56.6*
8.70E+00	*2.64E-01*	*55.4*
1.04E+01	*3.69E-01*	*54.4*
1.22E+01	*5.14E-01*	*53.8*
1.39E+01	*7.17E-01*	*53.3*
1.54E+01	*1.00E+00*	*53.0*

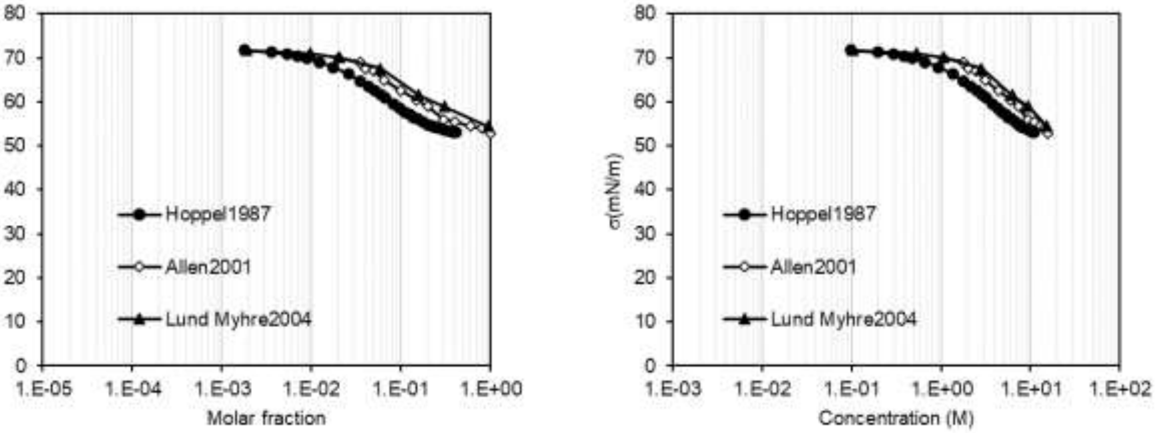

Fig. S6 (a): Comparison of the experimental surface tension data for methanesulfonic acid / water mixtures.

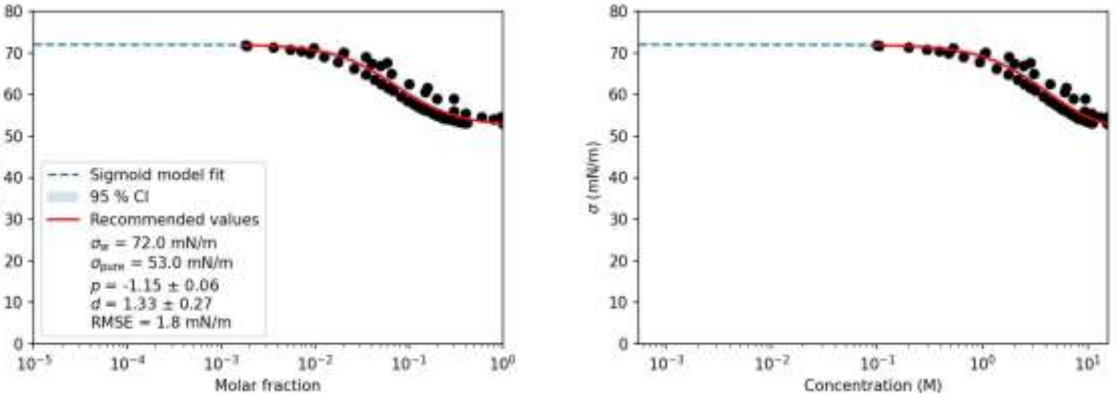

Fig. S6 (b): Surface tension fit with the Sigmoid model (*Kleinheins et al. 2023*) for methanesulfonic acid / water mixtures. Solid red line: model fit inside the concentration range covered by experimental data, reported as recommended values. Blue shading: fit parameters with 95 % confidence interval (CI). RMSE: root mean squared error. Markers: data used for fitting.

Comment: N/A

AC7: valeric acid (pentanoic acid)

Lunkenheimer et al. 2003 **Graph (experimental points)**			Suarez et al. 2011 **Table**			***Recommended***		
**C (M)**	**Molar fraction**	**σ (mN/m)**	**C (M)**	**Molar fraction**	**σ (mN/m)**	***C (M)***	***Molar fraction***	***σ (mN/m)***
**7.92E-04**	1.43E-05	72.1	0.00E+00	**0.00E+00**	71.81	*7.94E-04*	*1.43E-05*	*71.5*
**9.72E-04**	1.75E-05	71.8	8.88E-03	**1.60E-04**	64.74	*1.06E-03*	*1.91E-05*	*71.3*
**1.46E-03**	2.63E-05	71.5	2.66E-02	**4.80E-04**	57.26	*1.42E-03*	*2.55E-05*	*71.1*
**1.96E-03**	3.53E-05	71.3	3.10E-02	**5.60E-04**	56.03	*1.89E-03*	*3.41E-05*	*70.8*
**3.16E-03**	5.69E-05	70.3	4.59E-02	**8.30E-04**	52.37	*2.53E-03*	*4.55E-05*	*70.4*
**4.91E-03**	8.84E-05	68.9	5.47E-02	**9.90E-04**	50.13	*3.37E-03*	*6.08E-05*	*69.8*
**7.73E-03**	1.39E-04	66.9	6.68E-02	**1.21E-03**	47.42	*4.51E-03*	*8.11E-05*	*69.1*
**9.81E-03**	1.77E-04	66.0	8.27E-02	**1.50E-03**	45.23	*6.02E-03*	*1.08E-04*	*68.1*
**1.46E-02**	2.63E-04	63.0	9.53E-02	**1.73E-03**	44.11	*8.04E-03*	*1.45E-04*	*66.9*
**1.94E-02**	3.50E-04	61.0	1.08E-01	**1.96E-03**	42.61	*1.07E-02*	*1.93E-04*	*65.4*
**2.89E-02**	5.22E-04	57.4	1.20E-01	**2.19E-03**	41.11	*1.43E-02*	*2.58E-04*	*63.4*
**4.21E-02**	7.61E-04	52.8	1.42E-01	**2.59E-03**	39.26	*1.91E-02*	*3.45E-04*	*61.1*
**7.70E-02**	1.40E-03	46.7	1.55E-01	**2.83E-03**	38.07	*2.55E-02*	*4.60E-04*	*58.4*
**9.67E-02**	1.76E-03	44.3	1.64E-01	**2.99E-03**	37.70	*3.41E-02*	*6.15E-04*	*55.3*
**1.35E-01**	2.46E-03	36.9	1.78E-01	**3.26E-03**	36.48	*4.54E-02*	*8.21E-04*	*52.0*
			1.91E-01	**3.49E-03**	35.62	*6.06E-02*	*1.10E-03*	*48.6*
			9.14E+00	**1.00E+00**	26.63	*8.08E-02*	*1.47E-03*	*45.2*
						1.08E-01	*1.96E-03*	*42.0*
						1.43E-01	*2.61E-03*	*39.0*
						1.91E-01	*3.49E-03*	*36.5*

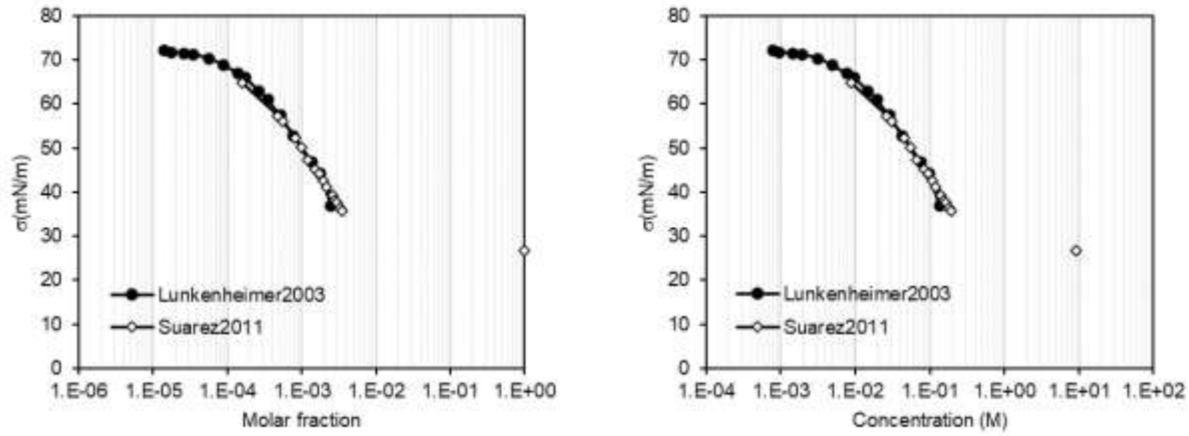

Fig. S7 (a): Comparison of the experimental surface tension data for valeric acid / water mixtures.

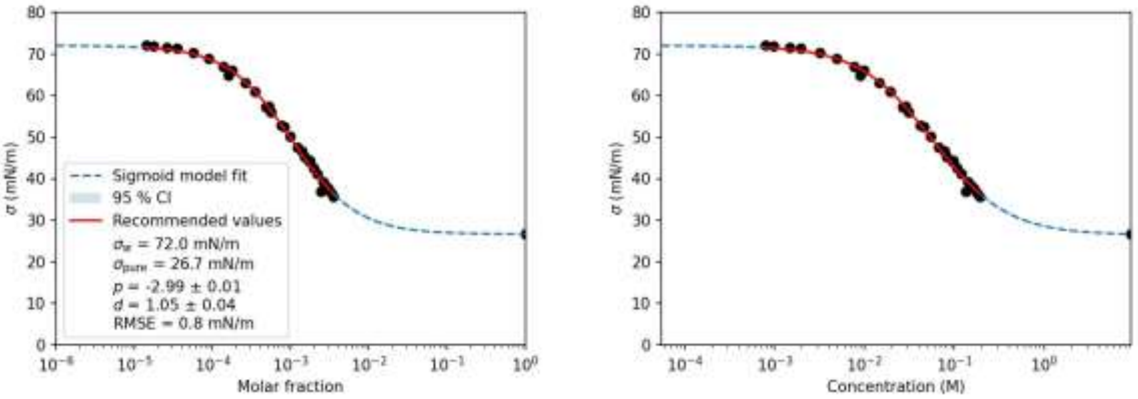

Fig. S7 (b): Surface tension fit with the Sigmoid model (*Kleinheins et al. 2023*) for valeric acid / water mixtures. Solid red line: model fit inside the concentration range covered by experimental data, reported as recommended values. Blue shading: fit parameters with 95 % confidence interval (CI). RMSE: root mean squared error. Markers: data used for fitting.

Comment

Lunkenheimer et al. 2003: aqueous solutions of valeric acid in 0.005M HCl.

AC8: malonic acid (propanedioic acid)

Hyvarinen et al. 2006 **Table**		
**C (M)**	**Molar fraction**	**σ (mN/m)**
5.42E-01	**1.00E-02**	69.2
9.05E-01	**1.70E-02**	68.5
1.31E+00	**2.50E-02**	66.7
1.69E+00	**3.30E-02**	66.7
2.02E+00	**4.00E-02**	65.6
2.46E+00	**5.00E-02**	65.6
2.68E+00	**5.50E-02**	65.5
3.10E+00	**6.50E-02**	65.0
3.30E+00	**7.00E-02**	64.2
3.69E+00	**8.00E-02**	63.5
3.99E+00	**8.80E-02**	64.2
4.21E+00	**9.40E-02**	63.5
4.67E+00	**1.07E-01**	62.7
5.17E+00	**1.22E-01**	62.3
5.64E+00	**1.37E-01**	62.2
6.00E+00	**1.49E-01**	62.6
6.26E+00	**1.58E-01**	62.2

Topping et al. 2007 **Graph (experimental points)**			
**C (M)**	**Molar fraction**	**Mass fraction**	**σ (mN/m)**
0.00E+00	0.00E+00	**0.00E+00**	71.4
1.88E-02	3.40E-04	**1.96E-03**	70.4
2.83E-02	5.10E-04	**2.94E-03**	72.3
6.30E-02	1.14E-03	**6.54E-03**	71.3
2.45E-01	4.45E-03	**2.52E-02**	70.7
5.75E-01	1.06E-02	**5.85E-02**	69.6
1.91E+00	3.78E-02	**1.85E-01**	66.1
4.63E+00	1.06E-01	**4.06E-01**	64.3

Varga et al. 2007 **Table**			
**C (M)**	**Molar fraction**	**Mass fraction (%)**	**σ (mN/m)**
9.61E-03	1.73E-04	**0.1**	71
4.81E-02	8.68E-04	**0.5**	71
9.65E-02	1.74E-03	**1**	71
4.90E-01	9.02E-03	**5**	70
1.00E+00	1.89E-02	**10**	68
3.26E+00	6.90E-02	**30**	65
4.55E+00	1.03E-01	**40**	64

Booth et al. 2009 **Table**		
**C (M)**	**Molar fraction**	**σ (mN/m)**
9.95E-02	**1.80E-03**	71.1
1.98E-01	**3.60E-03**	70.4
3.93E-01	**7.20E-03**	69.3
5.84E-01	**1.08E-02**	68.3
7.72E-01	**1.44E-02**	67.1
9.56E-01	**1.80E-02**	67.1
4.07E+00	**9.00E-02**	57.1
5.58E+00	**1.35E-01**	54.1
6.86E+00	**1.80E-01**	52.4
9.05E+00	**2.79E-01**	49.0

Lee et al. 2014 **Table**		
**C (M)**	**Molar fraction**	**σ (mN/m)**
**5.00E-02**	9.02E-04	72.1
**1.00E-01**	1.81E-03	71.9
**3.00E-01**	5.48E-03	70.9
**5.00E-01**	9.21E-03	70.0
**1.00E+00**	1.89E-02	67.7
**3.00E+00**	6.26E-02	63.9

Morris et al. 2015 **Graph (experimental points)**		
**C (M)**	**Molar fraction**	**σ (mN/m)**
**3.10E+01**		52
**3.90E+01**		51
**4.30E+01**		52
**5.00E+01**		50
**6.20E+01**		48
**7.50E+01**		44
**8.20E+01**		42

Recommended		
C (M)	***Molar fraction***	***σ (mN/m)***
9.61E-03	*1.73E-04*	*71.9*
1.38E-02	*2.48E-04*	*71.9*
1.97E-02	*3.55E-04*	*71.9*
2.82E-02	*5.08E-04*	*71.8*
4.03E-02	*7.27E-04*	*71.7*
5.76E-02	*1.04E-03*	*71.6*

8.24E-02	*1.49E-03*	*71.5*
1.18E-01	*2.13E-03*	*71.3*
1.68E-01	*3.05E-03*	*71.0*
2.40E-01	*4.37E-03*	*70.7*
3.42E-01	*6.26E-03*	*70.2*
4.86E-01	*8.96E-03*	*69.7*
6.90E-01	*1.28E-02*	*69.0*
9.74E-01	*1.84E-02*	*68.2*
1.37E+00	*2.63E-02*	*67.2*
1.91E+00	*3.76E-02*	*66.2*
2.63E+00	*5.39E-02*	*65.1*
3.58E+00	*7.71E-02*	*64.1*
4.79E+00	*1.10E-01*	*63.2*
6.26E+00	*1.58E-01*	*62.3*

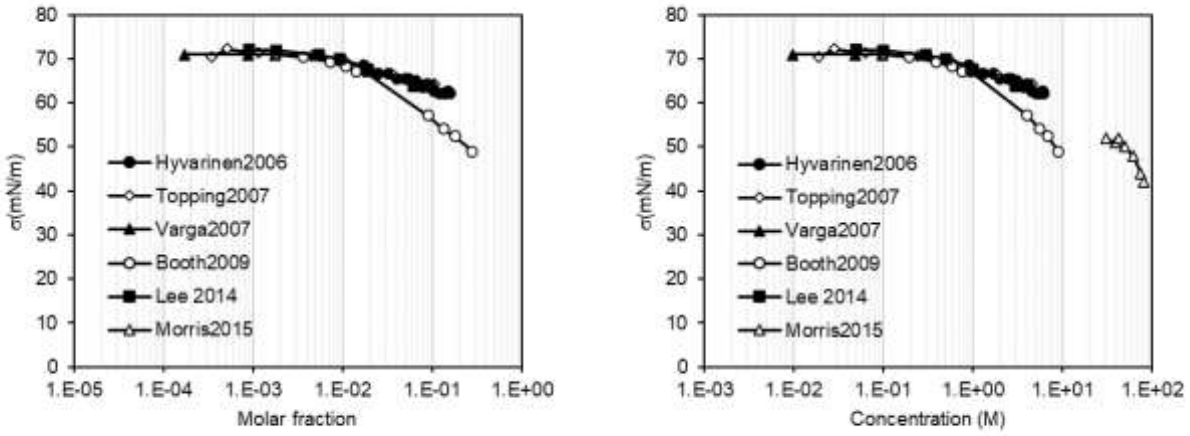

Fig. S8 (a): Comparison of the experimental surface tension data for malonic acid / water mixtures.

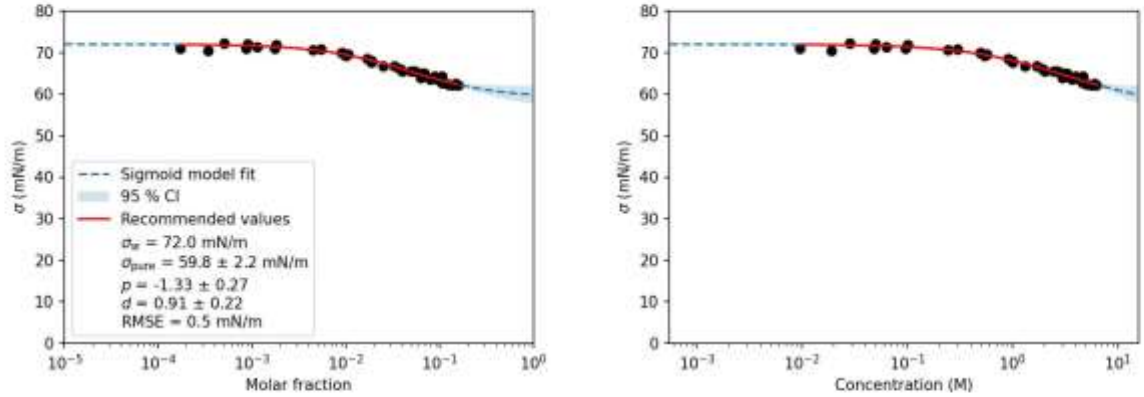

Fig. S8 (b): Surface tension fit with the Sigmoid model (*Kleinheins et al. 2023*) for malonic acid / water mixtures. Solid red line: model fit inside the concentration range covered by experimental data, reported as recommended values. Blue shading: fit parameters with 95 % confidence interval (CI). RMSE: root mean squared error. Markers: data used for fitting.

Comment

The data from *Morris et al. 2015* were not included for the fitting, because the concentrations used in their study (AFM study) are near to the saturation solubility and beyond a molar fraction of 1.

The data from *Booth et al. 2009* were not included for the fitting, because they differ from the other surface tension isotherms.

AC9: β-hydroxybutyric acid (3-hydroxybutanoic acid)

Tuckermann et al. 2004 **Graph (experimental points)**			
**C (M)**	**Molar fraction**	**C (mg/mL)**	**σ (mN/m)**
7.59E-05	1.37E-06	**7.90E-03**	72.6
1.58E-04	2.85E-06	**1.65E-02**	72.9
3.17E-04	5.71E-06	**3.30E-02**	72.3
6.34E-04	1.14E-05	**6.60E-02**	72.7
1.26E-03	2.27E-05	**1.31E-01**	72.6
2.51E-03	4.51E-05	**2.61E-01**	72.2
5.00E-03	9.01E-05	**5.21E-01**	71.9
9.99E-03	1.80E-04	**1.04E+00**	71.2
1.86E-02	3.36E-04	**1.94E+00**	70.3
5.37E-02	9.70E-04	**5.59E+00**	67.5

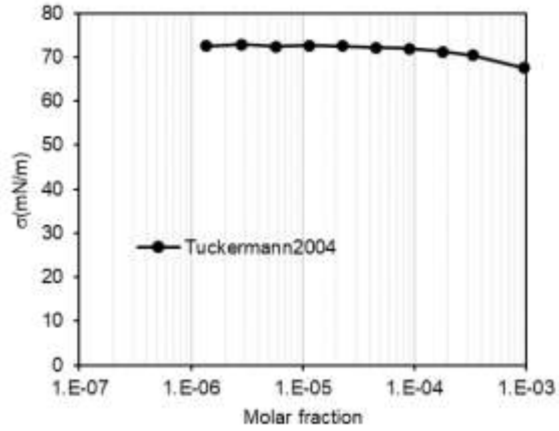

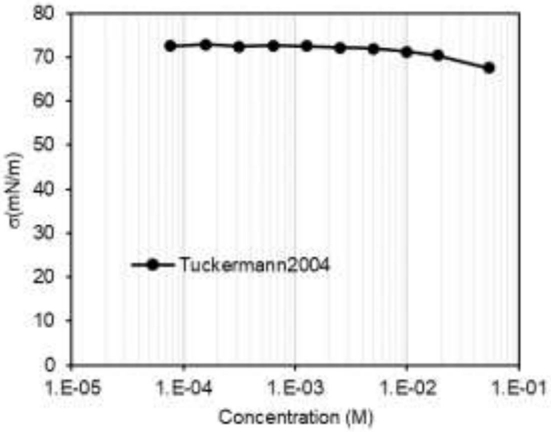

Fig. S9: Experimental surface tension data for β-Hydroxybutyric acid / water mixtures.

Comment: N/A

AC10: maleic acid ((2Z)-but-2-enedioic acid)

Hyvarinen et al. 2006 **Table**			Topping et al. 2007 **Graph (experimental points)**				Varga et al. 2007 **Table**			
**C (M)**	**Molar fraction**	**σ (mN/m)**	**C (M)**	**Molar fraction**	**Mass fraction**	**σ (mN/m)**	**C (M)**	**Molar fraction**	**Mass fraction (%)**	**σ (mN/m)**
4.34E-01	**8.00E-03**	68.0	0.00E+00	0.00E+00	**0.00E+00**	71.4	8.62E-03	1.55E-04	**0.1**	71
6.22E-01	**1.16E-02**	67.6	2.31E-02	4.17E-04	**2.68E-03**	71.2	4.32E-02	7.79E-04	**0.5**	71
8.98E-01	**1.70E-02**	65.9	1.01E-01	1.83E-03	**1.17E-02**	70.2	8.65E-02	1.56E-03	**1**	71
1.37E+00	**2.67E-02**	64.1	2.16E-01	3.93E-03	**2.48E-02**	70.3	4.39E-01	8.10E-03	**5**	69
1.86E+00	**3.74E-02**	61.4	4.74E-01	8.76E-03	**5.39E-02**	67.5	8.95E-01	1.69E-02	**10**	67
1.87E+00	**3.76E-02**	61.4	5.04E-01	9.34E-03	**5.73E-02**	68.4	2.91E+00	6.23E-02	**30**	62
2.36E+00	**4.89E-02**	62.2	9.60E-01	1.82E-02	**1.07E-01**	64.4	4.05E+00	9.37E-02	**40**	61
2.73E+00	**5.79E-02**	61.2	1.72E+00	3.42E-02	**1.86E-01**	64.6				
2.82E+00	**6.00E-02**	62.3	1.93E+00	3.89E-02	**2.07E-01**	63.2				
2.92E+00	**6.27E-02**	60.4	3.89E+00	8.92E-02	**3.87E-01**	59.5				
3.44E+00	**7.64E-02**	58.8	4.07E+00	9.44E-02	**4.02E-01**	60.8				
3.75E+00	**8.50E-02**	60.3								
4.03E+00	**9.32E-02**	59.7								
4.52E+00	**1.08E-01**	59.5								

Mahiuddin et al. 2008 **Graph (experimental points)**			***Recommended***		
**C (M)**	**Molar fraction**	**σ (mN/m)**	***C (M)***	***Molar fraction***	***σ (mN/m)***
**0.00E+00**	0.00E+00	72.0	*8.61E-03*	*1.55E-04*	*71.9*
**3.00E-02**	5.41E-04	71.7	*1.21E-02*	*2.19E-04*	*71.8*
**2.50E-01**	4.56E-03	69.7	*1.71E-02*	*3.09E-04*	*71.8*
**5.00E-01**	9.25E-03	68.0	*2.42E-02*	*4.36E-04*	*71.7*
**8.00E-01**	1.51E-02	66.2	*3.41E-02*	*6.15E-04*	*71.6*
**1.00E+00**	1.90E-02	65.0	*4.81E-02*	*8.68E-04*	*71.4*
**1.50E+00**	2.94E-02	63.2	*6.78E-02*	*1.23E-03*	*71.2*
**2.00E+00**	4.04E-02	61.9	*9.55E-02*	*1.73E-03*	*70.9*
**2.50E+00**	5.22E-02	59.9	*1.35E-01*	*2.44E-03*	*70.5*
**3.00E+00**	6.47E-02	59.2	*1.89E-01*	*3.44E-03*	*70.1*
**3.50E+00**	7.80E-02	58.1	*2.66E-01*	*4.86E-03*	*69.4*
**4.00E+00**	9.23E-02	56.9	*3.73E-01*	*6.86E-03*	*68.7*
**4.25E+00**	9.98E-02	56.9	*5.22E-01*	*9.68E-03*	*67.7*
**4.50E+00**	1.08E-01	56.8	*7.29E-01*	*1.37E-02*	*66.6*
			1.01E+00	*1.93E-02*	*65.4*
			1.40E+00	*2.72E-02*	*64.0*
			1.91E+00	*3.84E-02*	*62.5*
			2.58E+00	*5.42E-02*	*61.1*
			3.45E+00	*7.65E-02*	*59.7*
			4.51E+00	*1.08E-01*	*58.4*

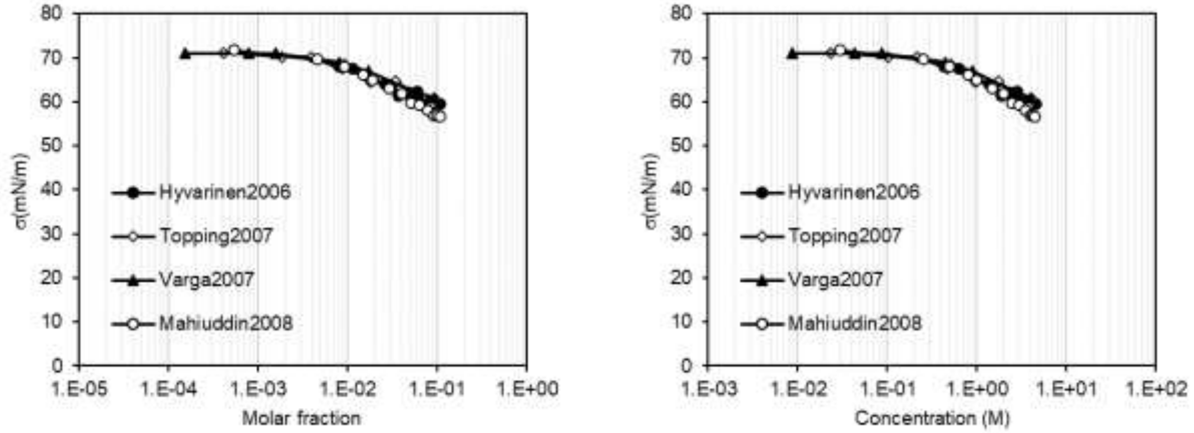

Fig. S10 (a): Comparison of the experimental surface tension data for maleic acid / water mixtures.

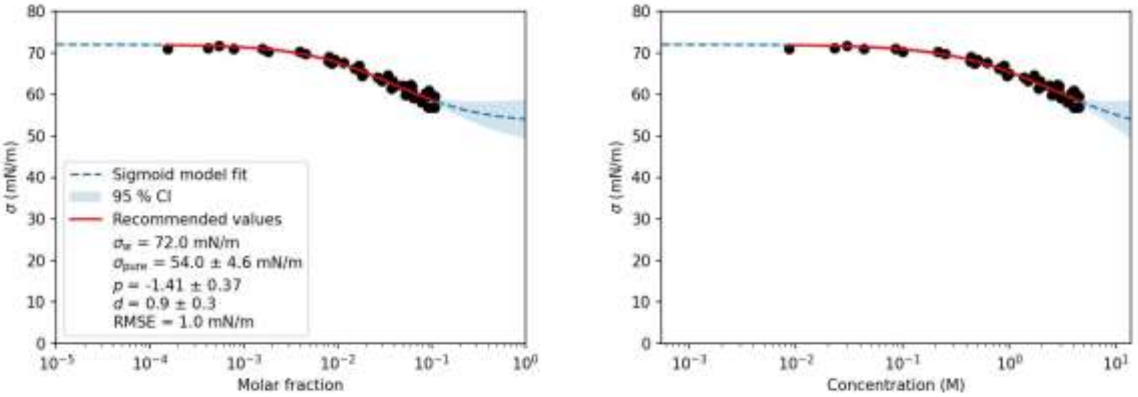

Fig. S10 (b): Surface tension fit with the Sigmoid model (*Kleinheins et al. 2023*) for maleic acid / water mixtures. Solid red line: model fit inside the concentration range covered by experimental data, reported as recommended values. Blue shading: fit parameters with 95 % confidence interval (CI). RMSE: root mean squared error. Markers: data used for fitting.

Comment: N/A

AC11: caproic acid (hexanoic acid)

Demou et al. 2002 **Graph (experimental points)**			Lunkenheimer et al. 2003 **Graph (experimental points)**			Suarez et al. 2011 **Table**		
**C (M)**	**Molar fraction**	**σ (mN/m)**	**C (M)**	**Molar fraction**	**σ (mN/m)**	**C (M)**	**Molar fraction**	**σ (mN/m)**
**1.57E-03**	2.82E-05	72.0	**1.95E-04**	3.51E-06	72.1	0.00E+00	**0.00E+00**	71.81
**2.78E-03**	5.01E-05	63.3	**2.93E-04**	5.27E-06	71.8	4.44E-03	**8.00E-05**	62.69
**4.08E-03**	7.34E-05	60.1	**4.82E-04**	8.68E-06	71.2	8.88E-03	**1.60E-04**	56.02
**5.33E-03**	9.61E-05	54.7	**6.69E-04**	1.20E-05	71.0	1.28E-02	**2.30E-04**	51.80
**7.84E-03**	1.41E-04	50.7	**9.61E-04**	1.73E-05	70.4	1.77E-02	**3.20E-04**	47.98
**1.04E-02**	1.88E-04	47.9	**1.37E-03**	2.47E-05	69.4	2.05E-02	**3.70E-04**	46.17
**1.56E-02**	2.81E-04	43.6	**1.94E-03**	3.49E-05	67.9	2.55E-02	**4.60E-04**	43.54
**2.07E-02**	3.74E-04	40.9	**2.89E-03**	5.20E-05	65.2	2.82E-02	**5.10E-04**	42.62
**2.59E-02**	4.68E-04	36.0	**4.82E-03**	8.68E-05	61.3	3.43E-02	**6.20E-04**	40.60
**3.07E-02**	5.55E-04	34.4	**6.63E-03**	1.19E-04	58.0	3.82E-02	**6.90E-04**	39.02
**3.62E-02**	6.53E-04	31.7	**9.13E-03**	1.65E-04	54.1	4.26E-02	**7.70E-04**	37.36
**4.13E-02**	7.47E-04	30.2	**1.89E-02**	3.41E-04	46.4	4.64E-02	**8.40E-04**	36.28
**4.64E-02**	8.40E-04	29.6	**2.85E-02**	5.15E-04	40.9	5.03E-02	**9.10E-04**	35.23
			**4.88E-02**	8.83E-04	34.0	6.07E-02	**1.10E-03**	33.17
						6.35E-02	**1.15E-03**	32.94
						7.93E+00	**1.00E+00**	27.51

Kaluarachchi et al. 2021 (AFM) **Graph (experimental points)**			Kaluarachchi et al. 2021 (bulk) **Graph (experimental points)**			***Recommended***		
**C (M)**	**Molar fraction**	**σ (mN/m)**	**C (M)**	**Molar fraction**	**σ (mN/m)**	***C (M)***	***Molar fraction***	***σ (mN/m)***
**0.00E-00**	0.00E-00	72.0	**0.00E-00**	0.00E-00	72.0	*1.00E-04*	*1.80E-06*	*71.9*
**1.00E-04**	1.80E-06	71.2	**1.00E-04**	1.80E-06	71.2	*1.42E-04*	*2.56E-06*	*71.8*
**1.00E-03**	1.80E-05	72.5	**1.00E-03**	1.80E-05	70.8	*2.02E-04*	*3.64E-06*	*71.7*
**3.00E-03**	5.40E-05	61.8	**3.00E-03**	5.40E-05	65.1	*2.88E-04*	*5.18E-06*	*71.5*
**6.00E-03**	1.08E-04	65.7	**6.00E-03**	1.08E-04	60.9	*4.09E-04*	*7.36E-06*	*71.3*
**1.00E-02**	1.80E-04	57.1	**1.00E-02**	1.80E-04	54.0	*5.82E-04*	*1.05E-05*	*70.9*
**2.00E-02**	3.61E-04	46.4	**2.00E-02**	3.61E-04	47.1	*8.27E-04*	*1.49E-05*	*70.4*
**4.00E-02**	7.23E-04	38.4	**4.00E-02**	7.23E-04	39.5	*1.18E-03*	*2.12E-05*	*69.6*
**6.00E-02**	1.09E-03	33.4	**6.00E-02**	1.09E-03	34.8	*1.67E-03*	*3.01E-05*	*68.4*
**8.00E-02**	1.45E-03	31.1	**8.00E-02**	1.45E-03	30.3	*2.38E-03*	*4.28E-05*	*66.7*
						3.38E-03	*6.09E-05*	*64.5*
						4.81E-03	*8.66E-05*	*61.4*
						6.84E-03	*1.23E-04*	*57.7*
						9.73E-03	*1.75E-04*	*53.3*
						1.38E-02	*2.49E-04*	*48.6*
						1.96E-02	*3.54E-04*	*44.0*
						2.79E-02	*5.04E-04*	*39.9*
						3.97E-02	*7.17E-04*	*36.5*
						5.63E-02	*1.02E-03*	*33.9*
						7.99E-02	*1.45E-03*	*31.9*

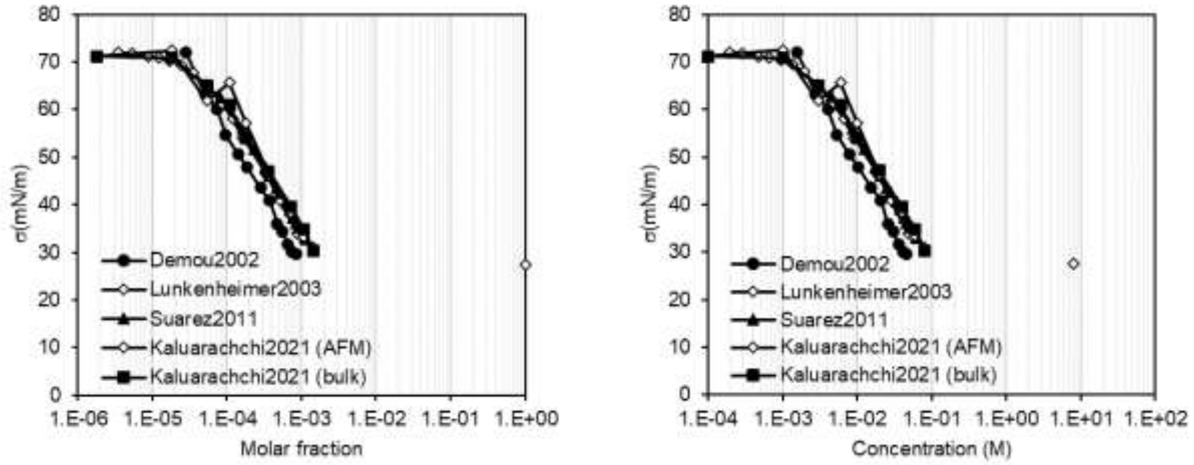

Fig. S11 (a): Comparison of the experimental surface tension data for caproic acid / water mixtures.

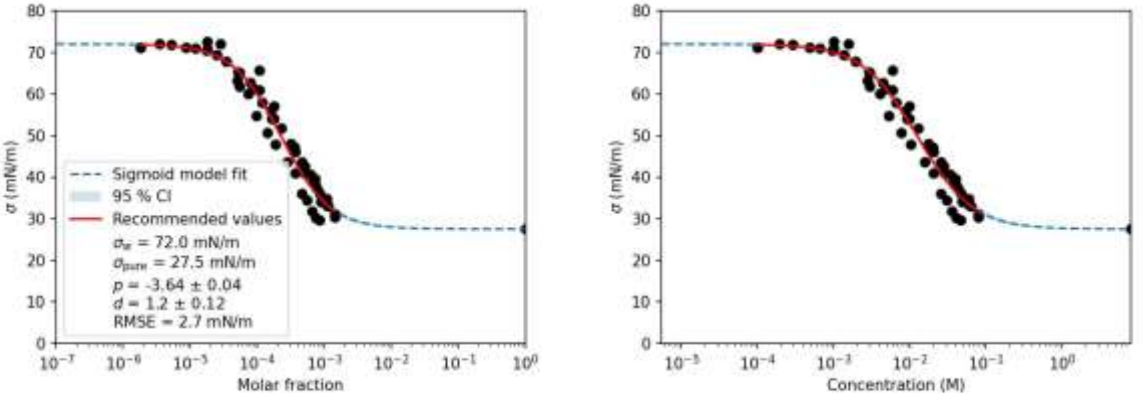

Fig. S11 (b): Surface tension fit with the Sigmoid model (*Kleinheins et al. 2023*) for hexanoic acid / water mixtures. Solid red line: model fit inside the concentration range covered by experimental data, reported as recommended values. Blue shading: fit parameters with 95 % confidence interval (CI). RMSE: root mean squared error. Markers: data used for fitting.

Comment:

Lunkenheimer et al. 2003: aqueous solutions of caproic acid in 0.005M HCl.

AC12: succinic acid (butanedioic acid)

Gaman et al. 2004 **Graph (experimental points)**			Hyvarinen et al. 2006 **Table**			Svenningsson et al. 2006 **Graph (experimental points)**			
**C (M)**	**Molar fraction**	**σ (mN/m)**	**C (M)**	**Molar fraction**	**σ (mN/m)**	**C (M)**	**Molar fraction**	**Molality (mol/kg)**	**σ (mN/m)**
7.27E-02	1.31E-03	72.2	6.09E-02	**1.1E-03**	70.9	2.79E-03	5.03E-05	**2.79E-03**	71.8
1.09E-01	1.97E-03	71.8	1.21E-01	**2.2E-03**	70.0	1.50E-02	2.69E-04	**1.50E-02**	71.7
2.52E-01	4.60E-03	70.1	1.54E-01	**2.8E-03**	69.8	7.34E-02	1.33E-03	**7.39E-02**	72.1
7.01E-01	1.31E-02	67.5	1.81E-01	**3.3E-03**	69.4	2.90E-01	5.31E-03	**2.96E-01**	71.5
			2.46E-01	**4.5E-03**	68.9	8.47E-01	1.60E-02	**9.04E-01**	70.0
			3.06E-01	**5.6E-03**	68.5	2.32E+00	4.81E-02	**2.80E+00**	67.4
			3.64E-01	**6.7E-03**	68.1				
			3.86E-01	**7.1E-03**	67.7				
			4.23E-01	**7.8E-03**	67.6				
			4.81E-01	**8.9E-03**	67.3				
			5.38E-01	**1.0E-02**	66.6				
			5.75E-01	**1.1E-02**	66.9				
			5.96E-01	**1.1E-02**	66.4				
			5.99E-01	**1.1E-02**	66.7				

Topping et al. 2007 **Graph (experimental points)**				Varga et al. 2007 **Table**				Mahiuddin et al. 2008 **Graph (experimental points)**		
**C (M)**	**Molar fraction**	**Mass fraction**	**σ (mN/m)**	**C (M)**	**Molar fraction**	**Mass fraction (%)**	**σ (mN/m)**	**C (M)**	**Molar fraction**	**σ (mN/m)**
0.00E+00	0.00E+00	**0.00E+00**	72.7	8.47E-03	1.53E-04	**0.1**	71	**0.00E+00**	0.00E+00	72.1
4.13E-03	7.44E-05	**4.88E-04**	72.6	4.24E-02	7.65E-04	**0.5**	71	**3.00E-02**	5.41E-04	71.6
6.20E-03	1.12E-04	**7.32E-04**	70.7	8.50E-02	1.54E-03	**1**	70	**5.00E-02**	9.03E-04	71.4
1.40E-02	2.52E-04	**1.65E-03**	71.1					**1.00E-01**	1.81E-03	70.6
1.80E-02	3.25E-04	**2.13E-03**	73.2					**1.50E-01**	2.72E-03	70.1
2.43E-02	4.38E-04	**2.87E-03**	69.8					**2.00E-01**	3.64E-03	69.8
6.11E-02	1.10E-03	**7.20E-03**	70.5					**2.50E-01**	4.57E-03	69.2
7.20E-02	1.30E-03	**8.48E-03**	72.5					**3.00E-01**	5.49E-03	68.5
2.08E-01	3.78E-03	**2.43E-02**	70.0					**3.60E-01**	6.62E-03	68.4
2.17E-01	3.96E-03	**2.54E-02**	71.0					**3.60E-01**	6.62E-03	68.1
6.44E-01	1.20E-02	**7.40E-02**	68.4					**4.00E-01**	7.37E-03	68.1
								**4.50E-01**	8.31E-03	67.7
								**5.00E-01**	9.26E-03	67.6
								**5.00E-01**	9.26E-03	67.3
								**5.50E-01**	1.02E-02	67.1
								**5.50E-01**	1.02E-02	66.9
								**6.00E-01**	1.12E-02	66.8
								**6.50E-01**	1.22E-02	66.6
								**6.70E-01**	1.25E-02	66.4

Vanhanen et al. 2008 **Graph (experimental points)**			Aumann et al. 2010 **Graph (experimental points)**			Booth et al. 2009 **Table**		
**C (M)**	**Molar fraction**	**σ (mN/m)**	**C (M)**	**Molar fraction**	**σ (mN/m)**	**C (M)**	**Molar fraction**	**σ (mN/m)**
8.29E-02	**1.50E-03**	70.8	**1.51E-03**	2.72E-05	72.0	1.05E-01	**1.90E-03**	70.0
1.65E-01	**3.00E-03**	70.0	**3.11E-03**	5.60E-05	71.9	5.12E-01	**9.50E-03**	66.5
2.46E-01	**4.50E-03**	69.3	**6.15E-03**	1.11E-04	71.8	1.00E+00	**1.91E-02**	66.1
3.27E-01	**6.00E-03**	68.7	**1.22E-02**	2.20E-04	71.7			
4.07E-01	**7.50E-03**	68.0	**3.05E-02**	5.50E-04	71.5			
			**7.64E-02**	1.38E-03	70.9			
			**1.51E-01**	2.75E-03	69.9			
			**3.11E-01**	5.69E-03	68.6			
			**4.26E-01**	7.85E-03	67.4			
			**5.99E-01**	1.12E-02	66.4			

Lee et al. 2014 **Table**			Werner et al. 2016 **Graph (experimental points)**			***Recommended***		
**C (M)**	**Molar fraction**	**σ (mN/m)**	**C (M)**	**Molar fraction**	**σ (mN/m)**	***C (M)***	***Molar fraction***	***σ (mN/m)***
**5.00E-02**	9.03E-04	71.8	0.00E+00	**0.00E+00**	73.8	*1.51E-03*	*2.72E-05*	*72.0*
**1.00E-01**	1.81E-03	71.3	9.94E-02	**1.80E-03**	72.3	*2.24E-03*	*4.03E-05*	*72.0*
**3.00E-01**	5.49E-03	68.8	1.98E-01	**3.60E-03**	71.0	*3.32E-03*	*5.98E-05*	*72.0*
**5.00E-01**	9.26E-03	68.1	2.46E-01	**4.50E-03**	70.8	*4.92E-03*	*8.86E-05*	*72.0*
			3.00E-01	**5.50E-03**	70.3	*7.29E-03*	*1.31E-04*	*72.0*
			3.91E-01	**7.20E-03**	69.5	*1.08E-02*	*1.95E-04*	*72.0*
						1.60E-02	*2.88E-04*	*72.0*
						2.37E-02	*4.28E-04*	*71.9*
						3.51E-02	*6.34E-04*	*71.8*
						5.20E-02	*9.39E-04*	*71.7*
						7.70E-02	*1.39E-03*	*71.4*
						1.14E-01	*2.06E-03*	*71.0*
						1.68E-01	*3.06E-03*	*70.2*
						2.48E-01	*4.54E-03*	*69.3*
						3.66E-01	*6.72E-03*	*68.3*
						5.37E-01	*9.96E-03*	*67.5*
						7.84E-01	*1.48E-02*	*66.9*
						1.14E+00	*2.19E-02*	*66.6*
						1.63E+00	*3.25E-02*	*66.4*
						2.32E+00	*4.81E-02*	*66.3*

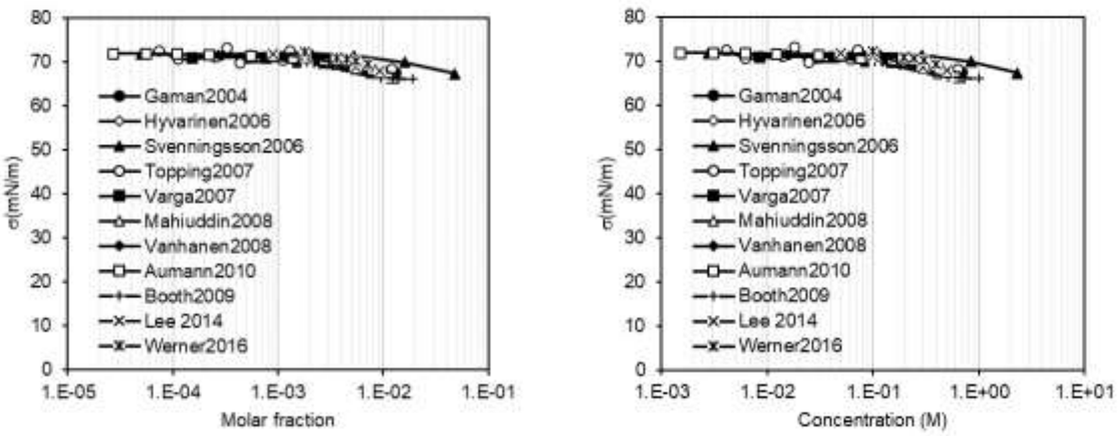

Fig. S12 (a): Comparison of the experimental surface tension data for succinic acid / water mixtures.

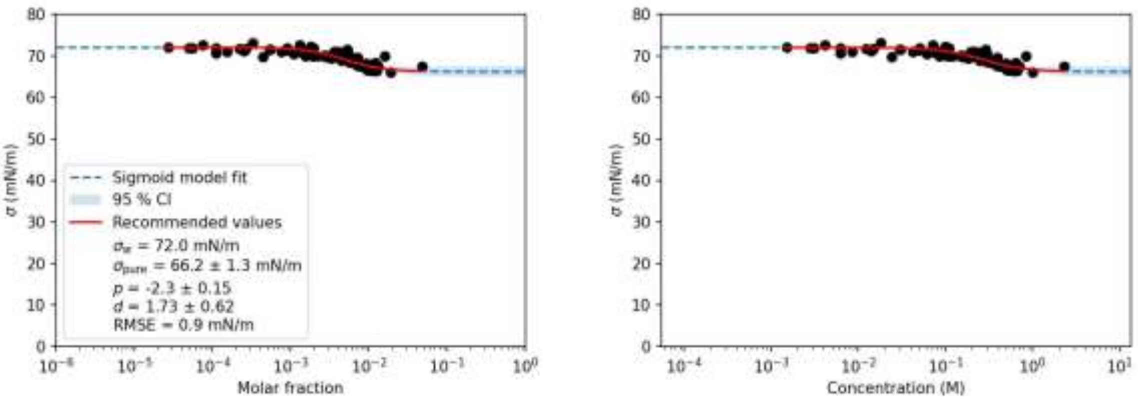

Fig. S12 (b): Surface tension fit with the Sigmoid model (*Kleinheins et al. 2023*) for succinic acid / water mixtures. Solid red line: model fit inside the concentration range covered by experimental data, reported as recommended values. Blue shading: fit parameters with 95 % confidence interval (CI). RMSE: root mean squared error. Markers: data used for fitting.

Comment:

Werner et al. 2016 : data reported at 10°C.

AC13: benzoic acid (benzenecarboxylic acid)

Dynarowicz 1994	**Graph (experimental points)**		
**C (M)**	**Molar fraction**	**logC (M)**	**σ (mN/m)**
9.69E-04	1.75E-05	**-3.01E+00**	72.7
2.46E-03	4.42E-05	**-2.61E+00**	72.4
4.96E-03	8.92E-05	**-2.30E+00**	71.7
1.02E-02	1.84E-04	**-1.99E+00**	70.3
1.50E-02	2.70E-04	**-1.83E+00**	68.4
2.04E-02	3.68E-04	**-1.69E+00**	66.4

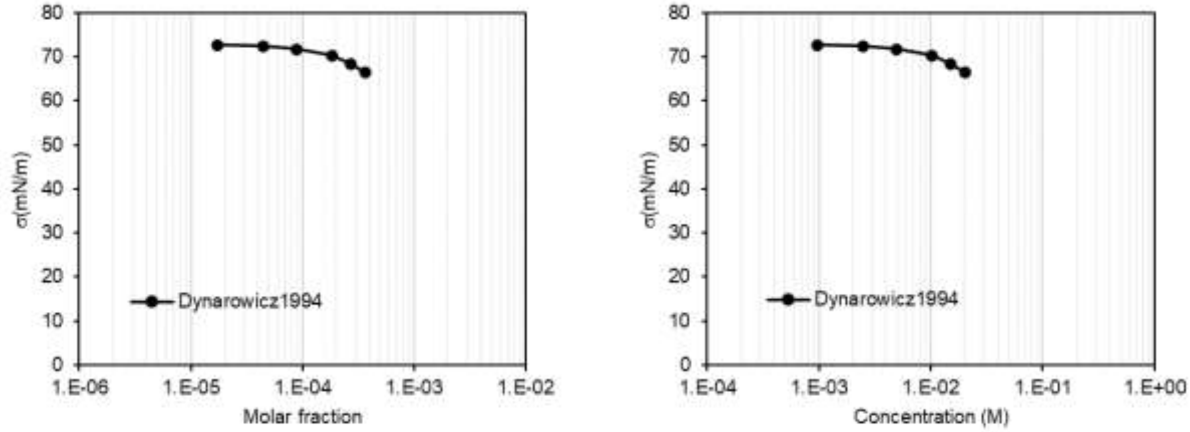

Fig. S13: Experimental surface tension data for benzoic acid / water mixtures.

Comment: N/A

AC14: cyclohexylmethanoic acid (cyclohexanecarboxylic acid)

Skauge et al. 1983		**Graph (experimental points)**	
**C (M)**	**Molar fraction**	**logC (M)**	**σ (mN/m)**
7.49E-04	1.35E-05	**-3.13E+00**	71.3
9.94E-04	1.79E-05	**-3.00E+00**	71.2
2.03E-03	3.65E-05	**-2.69E+00**	70.0
7.83E-03	1.41E-04	**-2.11E+00**	63.0
1.22E-02	2.19E-04	**-1.91E+00**	59.6
2.00E-02	3.60E-04	**-1.70E+00**	53.7
2.22E-02	4.00E-04	**-1.65E+00**	53.0
2.64E-02	4.76E-04	**-1.58E+00**	50.7
2.95E-02	5.32E-04	**-1.53E+00**	49.0

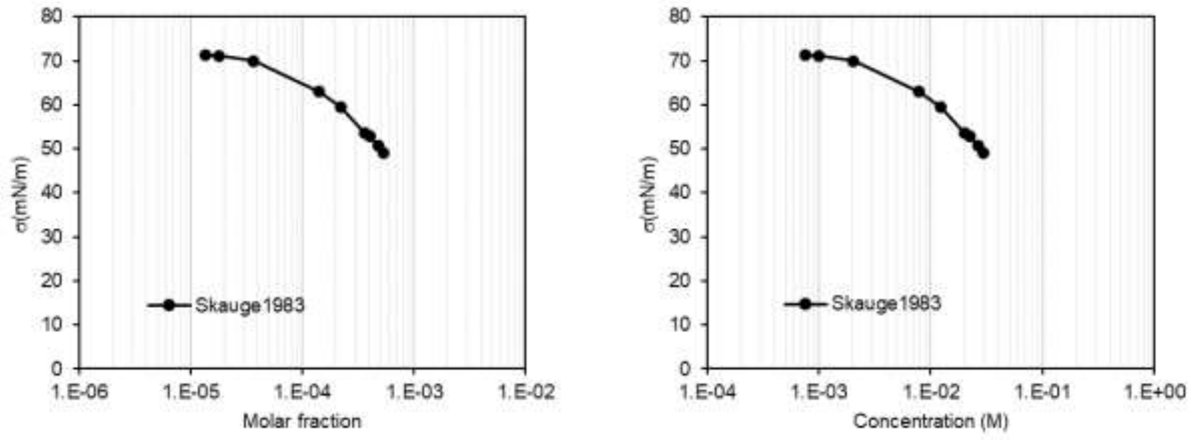

Fig. S14: Experimental surface tension data for cyclohexylmethanoic acid / water mixtures.

Comment: N/A

AC15: enanthic acid (heptanoic acid)

Glinski et al. 1996 **Graph (experimental points)**				Lunkenheimer et al. 2003 **Graph (experimental points)**			***Recommended***		
**C (M)**	**Molar fraction**	**logC (M)**	**σ (mN/m)**	**C (M)**	**Molar fraction**	**σ (mN/m)**	***C (M)***	***Molar fraction***	***σ (mN/m)***
5.05E-05	9.09E-07	**-4.30**	72.7	**4.89E-05**	8.80E-07	72.2	*4.89E-05*	*8.80E-07*	*71.8*
1.49E-04	2.68E-06	**-3.83**	72.0	**6.87E-05**	1.24E-06	72.2	*6.70E-05*	*1.21E-06*	*71.8*
2.46E-04	4.43E-06	**-3.61**	70.8	**9.76E-05**	1.76E-06	72.1	*9.17E-05*	*1.65E-06*	*71.6*
4.80E-04	8.63E-06	**-3.32**	67.7	**1.36E-04**	2.45E-06	72.0	*1.26E-04*	*2.26E-06*	*71.5*
9.68E-04	1.74E-05	**-3.01**	63.6	**1.93E-04**	3.47E-06	71.6	*1.72E-04*	*3.10E-06*	*71.2*
1.41E-03	2.54E-05	**-2.85**	60.4	**2.93E-04**	5.27E-06	71.1	*2.36E-04*	*4.25E-06*	*70.8*
3.12E-03	5.62E-05	**-2.51**	52.7	**4.77E-04**	8.59E-06	69.7	*3.23E-04*	*5.82E-06*	*70.2*
3.94E-03	7.10E-05	**-2.40**	50.6	**6.70E-04**	1.21E-05	68.3	*4.43E-04*	*7.97E-06*	*69.4*
4.89E-03	8.81E-05	**-2.31**	47.1	**9.75E-04**	1.76E-05	65.8	*6.06E-04*	*1.09E-05*	*68.3*
6.18E-03	1.11E-04	**-2.21**	44.4	**1.44E-03**	2.59E-05	62.2	*8.31E-04*	*1.50E-05*	*66.6*
7.82E-03	1.41E-04	**-2.11**	42.1	**1.89E-03**	3.40E-05	59.6	*1.14E-03*	*2.05E-05*	*64.5*
1.01E-02	1.81E-04	**-2.00**	38.6	**2.88E-03**	5.19E-05	55.0	*1.56E-03*	*2.81E-05*	*61.6*
1.27E-02	2.29E-04	**-1.90**	36.0	**4.75E-03**	8.56E-05	49.3	*2.13E-03*	*3.84E-05*	*58.2*
1.56E-02	2.82E-04	**-1.81**	33.5	**6.61E-03**	1.19E-04	45.0	*2.92E-03*	*5.27E-05*	*54.1*
				**9.52E-03**	1.72E-04	40.2	*4.01E-03*	*7.21E-05*	*49.8*
				**1.45E-02**	2.61E-04	34.3	*5.49E-03*	*9.88E-05*	*45.6*
				**1.93E-02**	3.48E-04	30.1	*7.51E-03*	*1.35E-04*	*41.7*
							1.03E-02	*1.85E-04*	*38.4*
							1.41E-02	*2.54E-04*	*35.8*
							1.93E-02	*3.48E-04*	*33.8*

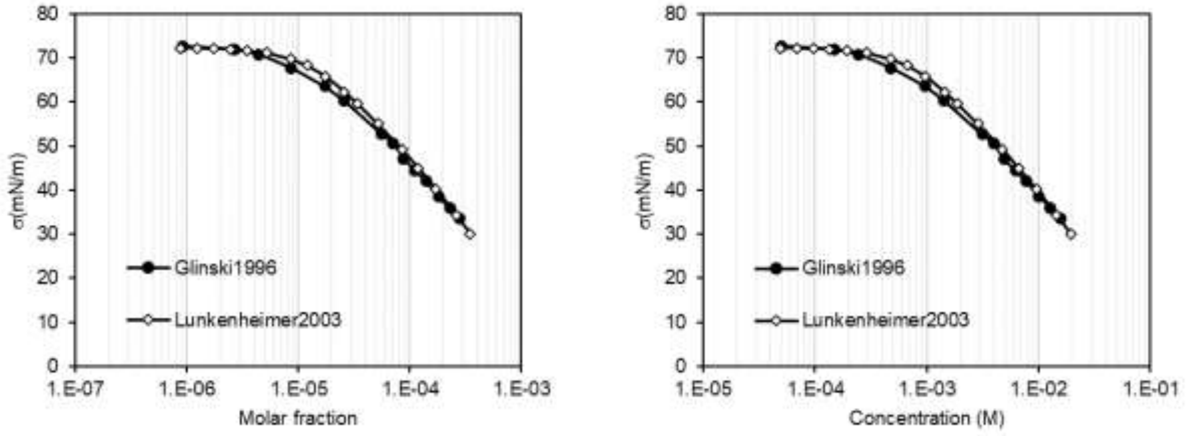

Fig. S15 (a): Comparison of the experimental surface tension data for enanthic acid / water mixtures.

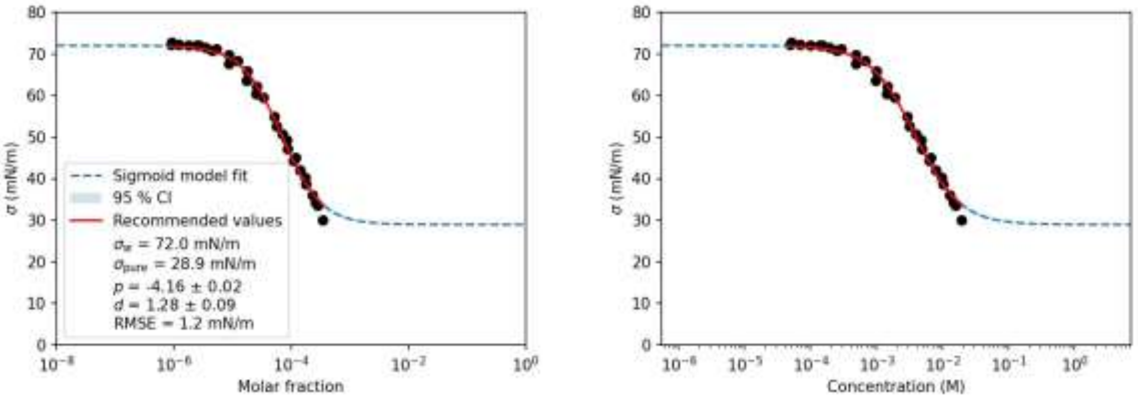

Fig. S15 (b): Surface tension fit with the Sigmoid model (*Kleinheins et al. 2023*) for enanthic acid / water mixtures. Solid red line: model fit inside the concentration range covered by experimental data, reported as recommended values. Blue shading: fit parameters with 95 % confidence interval (CI). RMSE: root mean squared error. Markers: data used for fitting.

Comment: N/A

Lunkenheimer et al. 2003: aqueous solutions of enanthic acid in 0.005M HCl.

AC16: glutaric acid (pentanedioic acid)

Gaman et al. 2004 **Graph (experimental points)**			Topping et al. 2007 **Graph (experimental points)**				Varga et al. 2007 **Table**			
**C (M)**	**Molar fraction**	**σ (mN/m)**	**C (M)**	**Molar fraction**	**Mass fraction**	**σ (mN/m)**	**C (M)**	**Molar fraction**	**Mass fraction (%)**	**σ (mN/m)**
7.39E-02	**1.34E-03**	71.4	8.10E-03	1.46E-04	**1.07E-03**	69.7	7.57E-03	1.36E-04	**0.1**	70
1.47E-01	**2.67E-03**	67.9	1.79E-01	3.27E-03	**2.35E-02**	68.0	3.79E-02	6.84E-04	**0.5**	69
5.69E-01	**1.07E-02**	64.0	3.90E-01	7.22E-03	**5.07E-02**	64.9	7.59E-02	1.37E-03	**1**	69
1.57E+00	**3.21E-02**	60.2	5.11E-01	9.57E-03	**6.62E-02**	64.2	3.84E-01	7.11E-03	**5**	66
4.72E+00	**1.31E-01**	51.9	1.36E+00	2.73E-02	**1.71E-01**	61.9	7.79E-01	1.49E-02	**10**	63
			1.53E+00	3.12E-02	**1.91E-01**	60.4	2.48E+00	5.48E-02	**30**	59
			3.14E+00	7.38E-02	**3.69E-01**	58.0	3.42E+00	8.25E-02	**40**	58
			4.69E+00	1.30E-01	**5.22E-01**	52.0				

Booth et al. 2009 **Table**			Aumann et al. 2010 **Graph (experimental points)**			Lee et al. 2014 **Table**		
**C (M)**	**Molar fraction**	**σ (mN/m)**	**C (M)**	**Molar fraction**	**σ (mN/m)**	**C (M)**	**Molar fraction**	**σ (mN/m)**
8.82E-01	**1.70E-02**	62.8	**4.50E-02**	8.13E-04	71.2	**5.00E-02**	9.03E-04	70.9
2.07E+00	**4.40E-02**	57.2	**8.63E-02**	1.56E-03	70.8	**1.00E-01**	1.81E-03	69.9
3.61E+00	**8.90E-02**	52.2	**1.43E-01**	2.60E-03	69.7	**3.00E-01**	5.52E-03	67.0
			**2.81E-01**	5.17E-03	67.2	**5.00E-01**	9.35E-03	65.1
			**5.70E-01**	1.07E-02	63.2	**1.00E+00**	1.94E-02	62.7
			**9.60E-01**	1.86E-02	59.3	**3.00E+00**	6.95E-02	58.6
			**1.61E+00**	3.28E-02	55.4			
			**2.16E+00**	4.63E-02	54.0			

Morris et al. 2015 **Graph (experimental points)**			Boyer et al. 2016 **Table**			Bzdek et al. 2016 Optical tweezer **Graph (experimental points)**		
**C (M)**	**Molar fraction**	**σ (mN/m)**	**C (M)**	**Molar fraction**	**σ (mN/m)**	**C (M)**	**Molar fraction**	**σ (mN/m)**
**4.48E+01**		42.0	1.73E-01	**3.16E-03**	68.6	**4.00E-01**	7.42E-03	63.0
**4.55E+01**		42.0	5.20E-01	**9.74E-03**	65.0	**5.00E-01**	9.35E-03	63.0
**4.60E+01**		42.5	8.68E-01	**1.67E-02**	62.4	**7.00E-01**	1.33E-02	63.0
**4.70E+01**		45.0	1.68E+00	**3.45E-02**	60.3	**9.00E-01**	1.74E-02	61.5
**4.75E+01**		45.5	2.67E+00	**6.00E-02**	57.9	**1.10E+00**	2.16E-02	60.5
**4.80E+01**		50.0	3.11E+00	**7.27E-02**	57.4	**1.50E+00**	3.04E-02	60.0
**4.85E+01**		51.0	3.53E+00	**8.62E-02**	56.9	**1.70E+00**	3.50E-02	58.8
			4.48E+00	**1.21E-01**	54.6	**1.85E+00**	3.86E-02	57.5
						**2.10E+00**	4.48E-02	57.5
						**2.60E+00**	5.80E-02	57.0
						**2.80E+00**	6.37E-02	57.0
						**2.90E+00**	6.66E-02	57.0
						**3.15E+00**	7.41E-02	55.5
						**3.35E+00**	8.03E-02	55.0
						**3.50E+00**	8.52E-02	55.0
						**3.80E+00**	9.54E-02	57.0
						**4.50E+00**	1.22E-01	53.0
						**4.65E+00**	1.28E-01	54.0

Bzdek et al. 2016 Kruss K100 **Graph (experimental points)**		
**C (M)**	**Molar fraction**	**σ (mN/m)**
**0.00E+00**	0.00E+00	73.0
**1.00E-01**	1.81E-03	69.0
**5.00E-01**	9.35E-03	66.0
**7.00E-01**	1.33E-02	62.0
**1.00E+00**	1.94E-02	61.5
**1.35E+00**	2.70E-02	61.0
**1.60E+00**	3.27E-02	60.0
**2.00E+00**	4.23E-02	59.0
**2.70E+00**	6.08E-02	58.5
**3.00E+00**	6.95E-02	57.0

Boyer et al. 2017 **Graph (experimental points)**			
**C (M)**	**Molar fraction**	**Molality (mol/kg)$^{1/2}$**	**σ (mN/m)**
3.59E-01	6.64E-03	**6.10E-01**	63.2
4.68E-01	8.73E-03	**7.00E-01**	63.2
6.76E-01	1.28E-02	**8.50E-01**	63.8
9.14E-01	1.76E-02	**1.00E+00**	61.8
1.10E+00	2.17E-02	**1.11E+00**	60.8
1.34E+00	2.69E-02	**1.24E+00**	61.1
1.50E+00	3.04E-02	**1.32E+00**	60.6
1.66E+00	3.40E-02	**1.40E+00**	59.3
1.88E+00	3.93E-02	**1.51E+00**	58.4
2.15E+00	4.61E-02	**1.64E+00**	58.5
2.62E+00	5.85E-02	**1.86E+00**	57.4
2.79E+00	6.33E-02	**1.94E+00**	56.9
2.92E+00	6.70E-02	**2.00E+00**	57.5
3.21E+00	7.60E-02	**2.14E+00**	56.7
3.36E+00	8.07E-02	**2.21E+00**	56.2
3.61E+00	8.89E-02	**2.33E+00**	55.8
3.86E+00	9.73E-02	**2.45E+00**	57.7
4.62E+00	1.27E-01	**2.84E+00**	52.0
4.78E+00	1.34E-01	**2.93E+00**	52.6
5.22E+00	1.54E-01	**3.18E+00**	57.1
5.85E+00	1.86E-01	**3.57E+00**	54.6
6.68E+00	2.39E-01	**4.18E+00**	51.6

El haber et al. 2023 **Table**		
**C (M)**	**Molar fraction**	**σ (mN/m)**
**0.00E+00**	0.00E+00	73.78
**3.20E-03**	5.76E-05	73.52
**7.70E-03**	1.39E-04	73.23
**1.80E-02**	3.24E-04	72.31
**1.90E-02**	3.42E-04	72.89
**2.60E-02**	4.69E-04	72.18
**4.70E-02**	8.49E-04	72.27
**5.90E-02**	1.07E-03	71.28
**7.40E-02**	1.34E-03	70.92
**2.80E-01**	5.15E-03	66.00
**5.50E-01**	1.03E-02	64.18
**7.10E-01**	1.35E-02	62.08
**1.40E+00**	2.81E-02	58.05
**2.20E+00**	4.74E-02	54.82
**3.00E+00**	6.95E-02	53.28

Recommended		
C (M)	***Molar fraction***	***σ (mN/m)***
3.20E-03	*5.76E-05*	*71.8*
4.96E-03	*8.93E-05*	*71.7*
7.69E-03	*1.38E-04*	*71.6*
1.19E-02	*2.15E-04*	*71.5*
1.85E-02	*3.33E-04*	*71.3*
2.86E-02	*5.16E-04*	*71.0*
4.43E-02	*8.00E-04*	*70.6*
6.85E-02	*1.24E-03*	*70.0*
1.06E-01	*1.92E-03*	*69.3*
1.64E-01	*2.98E-03*	*68.3*
2.52E-01	*4.62E-03*	*67.1*
3.86E-01	*7.16E-03*	*65.6*
5.90E-01	*1.11E-02*	*63.8*
8.93E-01	*1.72E-02*	*62.0*
1.34E+00	*2.67E-02*	*60.0*
1.96E+00	*4.14E-02*	*58.2*
2.82E+00	*6.41E-02*	*56.5*
3.91E+00	*9.94E-02*	*55.1*
5.23E+00	*1.54E-01*	*54.0*
6.68E+00	*2.39E-01*	*53.1*

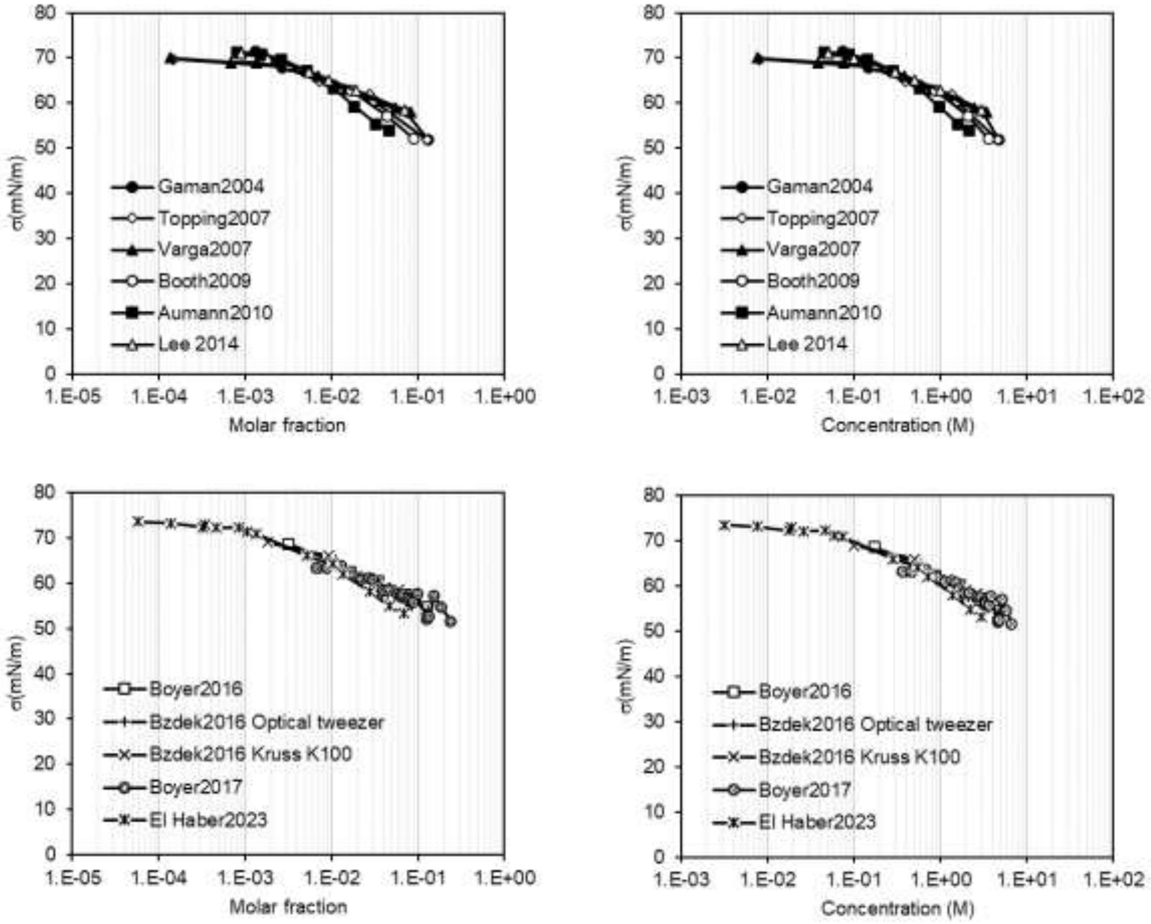

Fig. S16 (a): Comparison of the experimental surface tension data for glutaric acid / water mixtures.

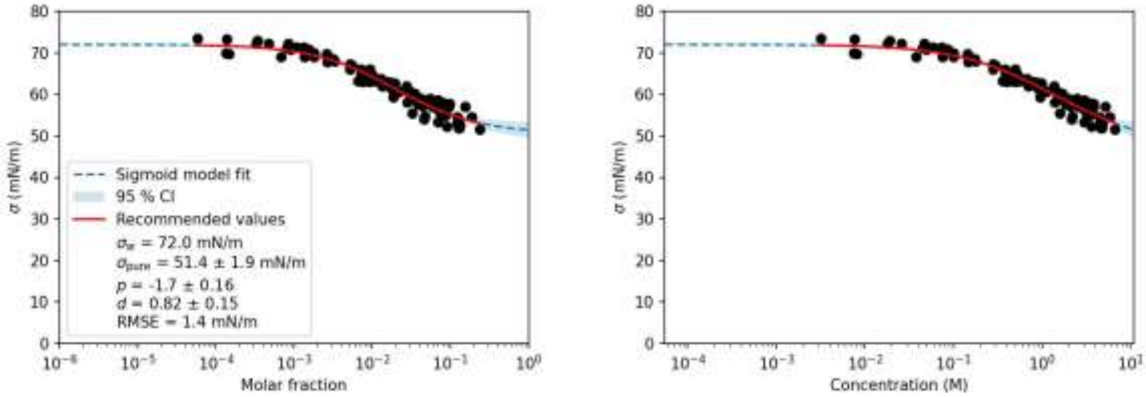

Fig. S16 (b): Surface tension fit with the Sigmoid model (*Kleinheins et al. 2023*) for glutaric acid / water mixtures. Solid red line: model fit inside the concentration range covered by experimental data, reported as recommended values. Blue shading: fit parameters with 95 % confidence interval (CI). RMSE: root mean squared error. Markers: data used for fitting.

Comment

The data from *Morris et al. 2015* were not included for the fitting, because the concentrations used in their study (AFM study) are near to the saturation solubility and beyond a molar fraction of 1.

AC17: malic acid (2-hydroxybutanedioic acid)

Hyvarinen et al. 2006 **Table**		
**C (M)**	**Molar fraction**	**σ (mN/m)**
1.65E-01	**3.0E-03**	70.3
3.79E-01	**7.0E-03**	70.1
7.90E-01	**1.5E-02**	69.8
1.18E+00	**2.3E-02**	69.3
1.59E+00	**3.2E-02**	69.3
2.06E+00	**4.3E-02**	68.4
2.51E+00	**5.4E-02**	68.4
2.66E+00	**5.8E-02**	68.1
3.10E+00	**7.0E-02**	67.8
3.51E+00	**8.2E-02**	67.3
3.67E+00	**8.7E-02**	66.8

Topping et al. 2007 **Graph (experimental points)**			
**C (M)**	**Molar fraction**	**Mass fraction**	**σ (mN/m)**
1.01E-02	1.83E-04	**1.36E-03**	72.1
1.89E-01	3.44E-03	**2.51E-02**	71.4
4.45E-01	8.26E-03	**5.84E-02**	71.0
1.52E+00	3.03E-02	**1.89E-01**	69.4
3.56E+00	8.37E-02	**4.05E-01**	67.7

Varga et al. 2007 **Table**			
**C (M)**	**Molar fraction**	**Mass fraction (%)**	**σ (mN/m)**
7.46E-03	1.34E-04	**0.1**	72
3.74E-02	6.74E-04	**0.5**	72
7.49E-02	1.35E-03	**1**	72
3.80E-01	7.02E-03	**5**	71
7.75E-01	1.47E-02	**10**	70
2.52E+00	5.44E-02	**30**	68
3.51E+00	8.21E-02	**40**	68

Recommended		
C (M)	***Molar fraction***	***σ (mN/m)***
7.44E-03	*1.34E-04*	*71.9*
1.05E-02	*1.88E-04*	*71.9*
1.47E-02	*2.65E-04*	*71.8*
2.07E-02	*3.73E-04*	*71.8*
2.90E-02	*5.24E-04*	*71.8*
4.08E-02	*7.37E-04*	*71.7*
5.73E-02	*1.04E-03*	*71.6*
8.05E-02	*1.46E-03*	*71.5*
1.13E-01	*2.05E-03*	*71.4*
1.58E-01	*2.88E-03*	*71.3*
2.22E-01	*4.05E-03*	*71.1*
3.10E-01	*5.69E-03*	*70.9*
4.32E-01	*8.01E-03*	*70.6*
6.01E-01	*1.13E-02*	*70.3*
8.31E-01	*1.58E-02*	*69.9*
1.14E+00	*2.23E-02*	*69.5*
1.56E+00	*3.13E-02*	*69.1*
2.11E+00	*4.40E-02*	*68.5*
2.80E+00	*6.19E-02*	*68.0*
3.67E+00	*8.70E-02*	*67.4*

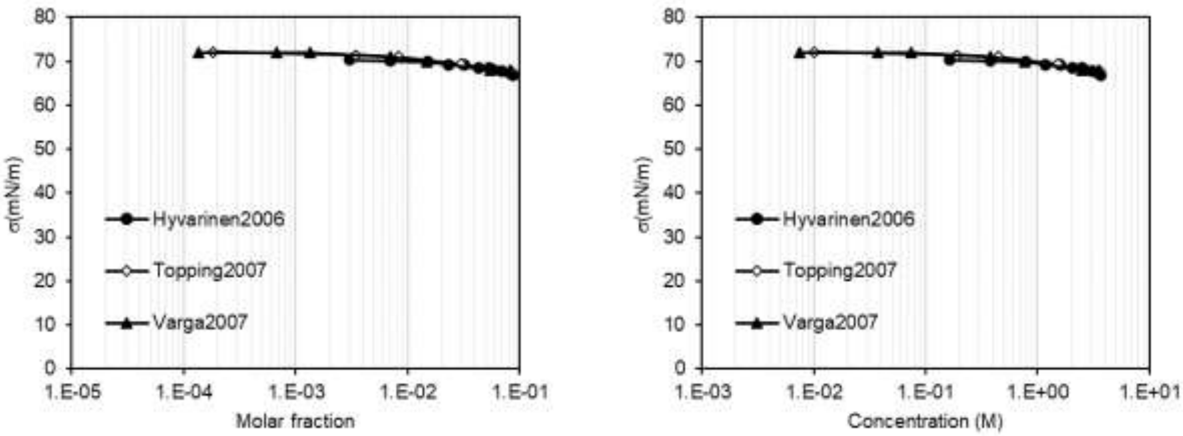

Fig. S17 (a): Comparison of the experimental surface tension data for malic acid / water mixtures.

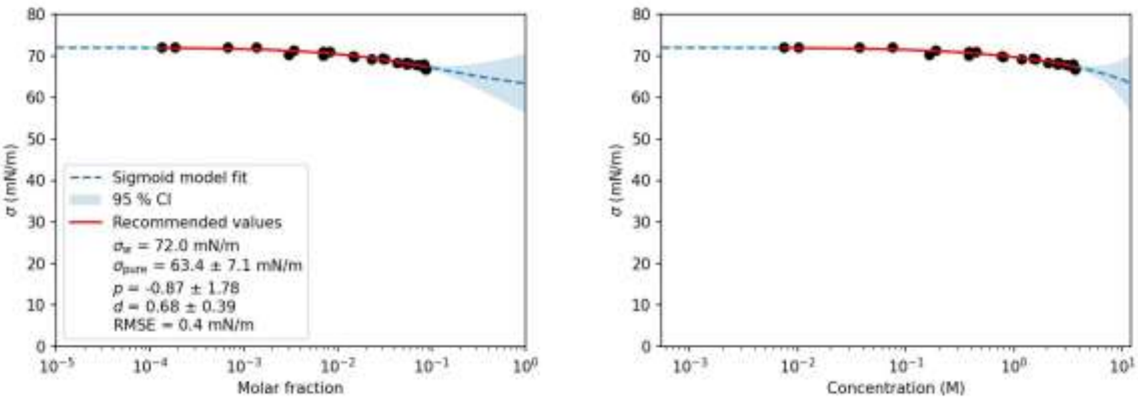

Fig. S17 (b): Surface tension fit with the Sigmoid model (*Kleinheins et al. 2023*) for malic acid / water mixtures. Solid red line: model fit inside the concentration range covered by experimental data, reported as recommended values. Blue shading: fit parameters with 95 % confidence interval (CI). RMSE: root mean squared error. Markers: data used for fitting.

Comment: N/A

AC18: p-toluic acid (4-methylbenzoic acid)

Dynarowicz 1994	**Graph (experimental points)**		
**C (M)**	**Molar fraction**	**logC (M)**	**σ (mN/m)**
2.96E-04	5.32E-06	**-3.53E+00**	72.6
6.16E-04	1.11E-05	**-3.21E+00**	72.5
9.90E-04	1.78E-05	**-3.00E+00**	72.0
1.53E-03	2.75E-05	**-2.82E+00**	71.5
2.51E-03	4.51E-05	**-2.60E+00**	70.5

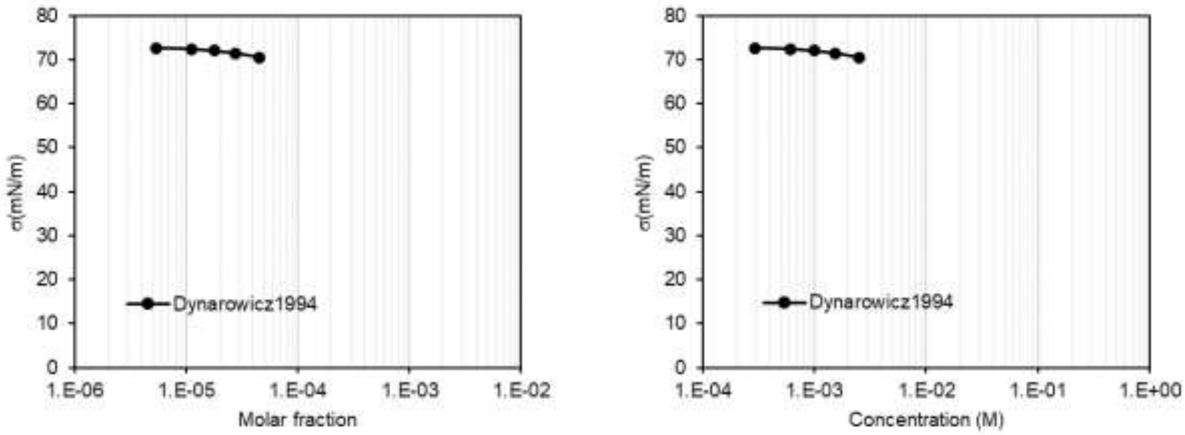

Fig. S18: Experimental surface tension data for p-toluic acid /water mixtures.

Comment: N/A

AC19: 3-hydroxybenzoic acid

Dynarowicz 1994		**Graph (experimental points)**	
**C (M)**	**Molar fraction**	**C (mg/mL)**	**σ (mN/m)**
7.60E-06	1.37E-07	**1.05E-03**	72.0
1.52E-05	2.74E-07	**2.10E-03**	72.7
3.04E-05	5.47E-07	**4.20E-03**	72.3
6.23E-05	1.12E-06	**8.60E-03**	72.3
1.21E-04	2.18E-06	**1.67E-02**	72.9
2.39E-04	4.30E-06	**3.30E-02**	72.3
4.85E-04	8.73E-06	**6.70E-02**	72.2
9.85E-04	1.77E-05	**1.36E-01**	71.6
1.95E-03	3.52E-05	**2.70E-01**	71.8
3.90E-03	7.01E-05	**5.38E-01**	71.4
7.89E-03	1.42E-04	**1.09E+00**	71.3
1.46E-02	2.63E-04	**2.01E+00**	71.1
3.71E-02	6.69E-04	**5.12E+00**	70.4

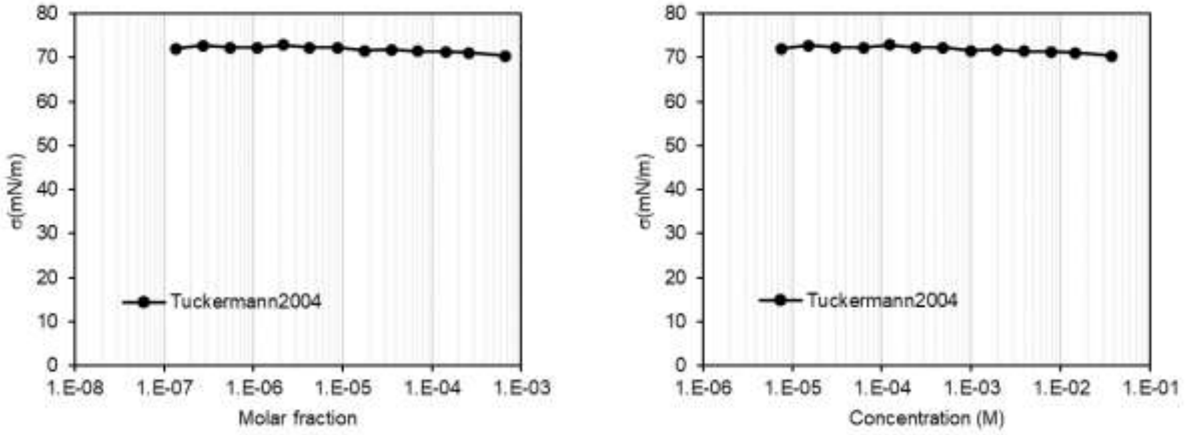

Fig. S19: Experimental surface tension data for 3-hydroxybenzoic acid / water mixtures.

Comment: N/A

AC20: cyclohexylethanoic acid

Skauge et al. 1983	**Graph (experimental points)**		
**C (M)**	**Molar fraction**	**logC (M)**	**σ (mN/m)**
9.98E-04	1.80E-05	**-3.00E+00**	69.7
3.84E-03	6.91E-05	**-2.42E+00**	59.5
7.82E-03	1.41E-04	**-2.11E+00**	52.4
8.70E-03	1.57E-04	**-2.06E+00**	50.6
1.09E-02	1.96E-04	**-1.96E+00**	48.0
1.47E-02	2.66E-04	**-1.83E+00**	44.7
1.61E-02	2.90E-04	**-1.79E+00**	43.5
1.89E-02	3.42E-04	**-1.72E+00**	41.5

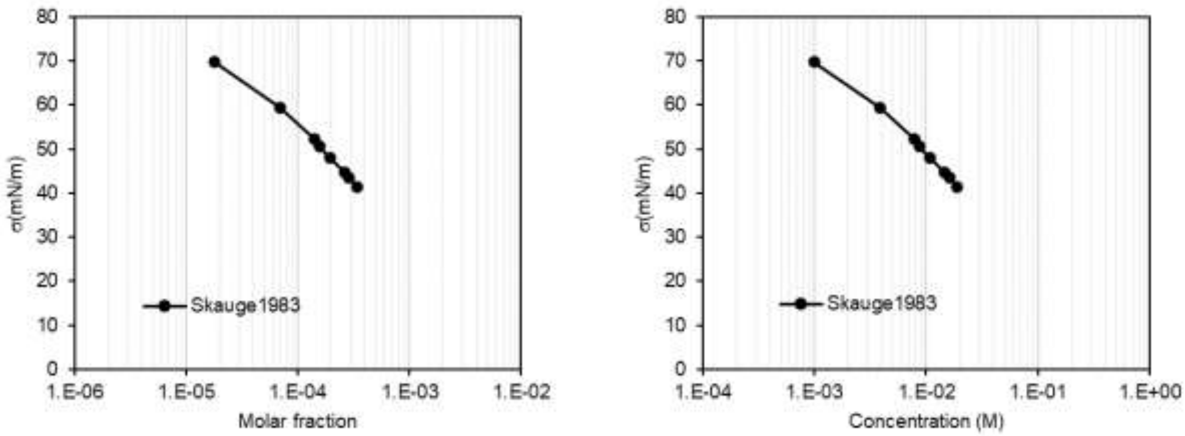

Fig. S20: Experimental surface tension data for cyclohexylethanoic acid / water mixtures.

Comment: N/A

AC21: caprylic acid (octanoic acid)

Lunkenheimer et al. 2003 **Graph (experimental points)**		
**C (M)**	**Molar fraction**	**σ (mN/m)**
**1.98E-05**	3.56E-07	72.3
**3.01E-05**	5.41E-07	72.2
**3.95E-05**	7.10E-07	72.1
**4.89E-05**	8.81E-07	71.8
**5.87E-05**	1.06E-06	71.5
**6.95E-05**	1.25E-06	71.2
**8.33E-05**	1.50E-06	71.1
**9.99E-05**	1.80E-06	70.9
**1.27E-04**	2.28E-06	70.4
**1.47E-04**	2.64E-06	69.8
**1.66E-04**	2.99E-06	69.7
**1.97E-04**	3.55E-06	69.2
**3.00E-04**	5.41E-06	65.8
**4.90E-04**	8.82E-06	61.5
**6.90E-04**	1.24E-05	57.5
**9.82E-04**	1.77E-05	53.6
**1.99E-03**	3.58E-05	45.0
**2.90E-03**	5.22E-05	39.2
**4.96E-03**	8.93E-05	31.8

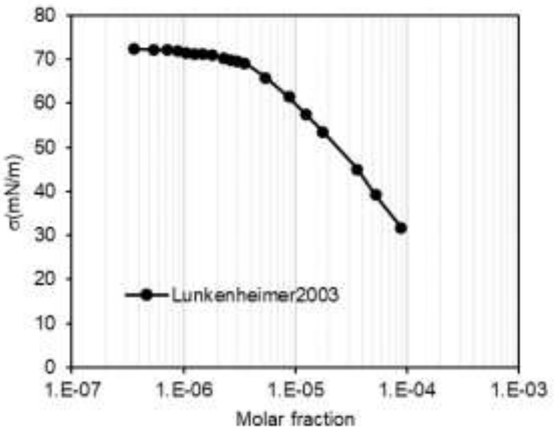

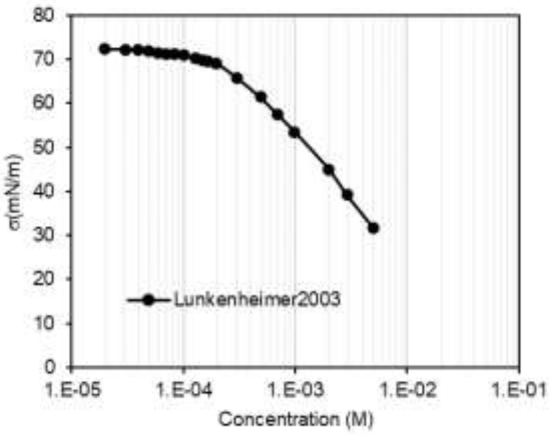

Fig. S21: Experimental surface tension data for caprylic acid / water mixtures.

Comment:

Lunkenheimer et al. 2003: aqueous solutions of caprylic acid in 0.005M HCl.

AC22: adipic acid (hexanedioic acid)

Riipinen et al. 2007 **Table**			Topping et al. 2007 **Graph (experimental points)**				Varga et al. 2007 **Table**			
**C (M)**	**Molar fraction**	**σ (mN/m)**	**C (M)**	**Molar fraction**	**Mass fraction**	**σ (mN/m)**	**C (M)**	**Molar fraction**	**Mass fraction (%)**	**σ (mN/m)**
2.78E-03	**5.00E-05**	71.0	5.40E-03	9.72E-05	**7.89E-04**	71.2	6.84E-03	1.23E-04	**0.1**	70
5.00E-03	**9.00E-05**	71.1	1.78E-02	3.21E-04	**2.60E-03**	69.5	3.43E-02	6.19E-04	**0.5**	69
5.55E-03	**1.00E-04**	70.7	4.25E-02	7.68E-04	**6.20E-03**	68.4	6.86E-02	1.24E-03	**1**	68
1.66E-02	**3.00E-04**	70.0	7.55E-02	1.37E-03	**1.10E-02**	67.4				
2.77E-02	**5.00E-04**	69.6								
3.32E-02	**6.00E-04**	69.3								
4.43E-02	**8.00E-04**	68.3								
4.98E-02	**9.00E-04**	68.1								
7.18E-02	**1.30E-03**	67.4								
8.27E-02	**1.50E-03**	66.7								
9.91E-02	**1.80E-03**	66.4								
1.10E-01	**2.00E-03**	65.4								
1.21E-01	**2.20E-03**	65.5								
1.37E-01	**2.50E-03**	64.7								
1.43E-01	**2.60E-03**	64.7								
1.53E-01	**2.80E-03**	64.6								

Aumann et al. 2010 **Graph (experimental points)**			Booth et al. 2009 **Table**			***Recommended***		
**C (M)**	**Molar fraction**	**σ (mN/m)**	**C (M)**	**Molar fraction**	**σ (mN/m)**	***C (M)***	***Molar fraction***	***σ (mN/m)***
**2.90E-04**	5.22E-06	71.9	1.66E-02	**3.00E-04**	69.5	*2.90E-04*	*5.22E-06*	*71.8*
**5.70E-04**	1.03E-05	71.9	3.32E-02	**6.00E-04**	68.0	*4.04E-04*	*7.27E-06*	*71.8*
**1.14E-03**	2.05E-05	71.8	9.91E-02	**1.80E-03**	67.7	*5.62E-04*	*1.01E-05*	*71.7*
**2.25E-03**	4.05E-05	71.7				*7.82E-04*	*1.41E-05*	*71.7*
**5.83E-03**	1.05E-04	71.5				*1.09E-03*	*1.96E-05*	*71.6*
**1.41E-02**	2.54E-04	70.8				*1.52E-03*	*2.73E-05*	*71.5*
**2.92E-02**	5.26E-04	69.8				*2.11E-03*	*3.80E-05*	*71.4*
**5.73E-02**	1.04E-03	68.2				*2.94E-03*	*5.29E-05*	*71.3*
**8.58E-02**	1.56E-03	66.7				*4.09E-03*	*7.36E-05*	*71.2*
**1.14E-01**	2.08E-03	65.6				*5.69E-03*	*1.02E-04*	*71.0*
						7.92E-03	*1.43E-04*	*70.7*
						1.10E-02	*1.99E-04*	*70.5*
						1.53E-02	*2.76E-04*	*70.1*
						2.13E-02	*3.85E-04*	*69.7*
						2.97E-02	*5.36E-04*	*69.2*
						4.13E-02	*7.46E-04*	*68.6*
						5.74E-02	*1.04E-03*	*67.9*
						7.97E-02	*1.44E-03*	*67.0*
						1.11E-01	*2.01E-03*	*65.9*
						1.53E-01	*2.80E-03*	*64.6*

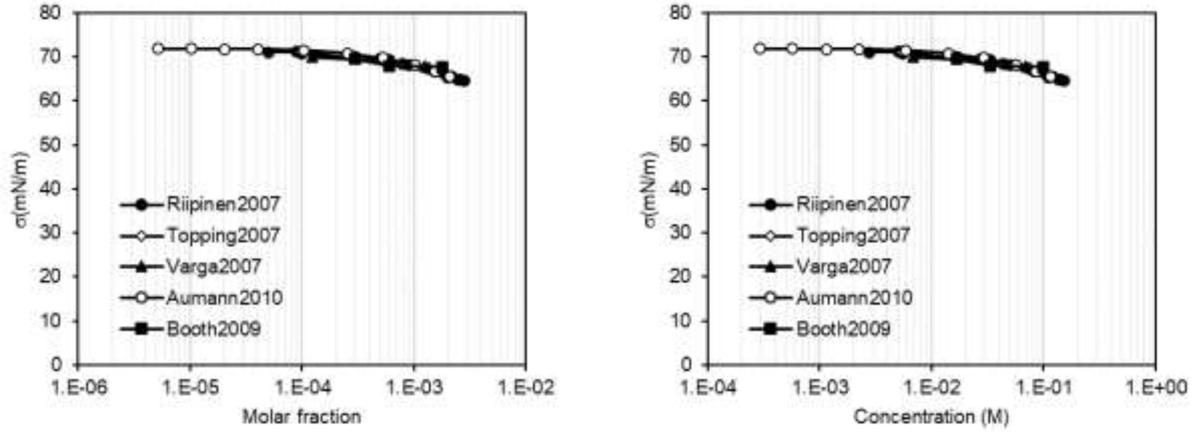

Fig. S22 (a): Comparison of the experimental surface tension data for adipic acid / water mixtures.

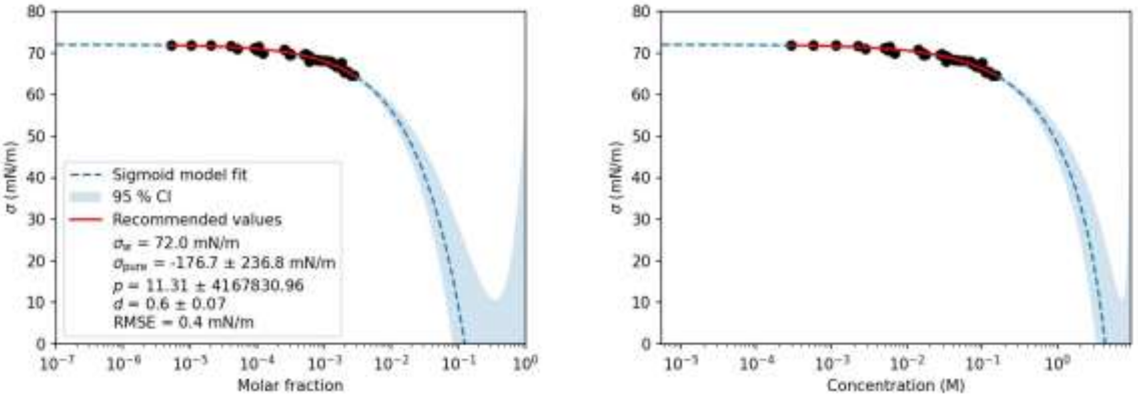

Fig. S22 (b): Surface tension fit with the Sigmoid model (*Kleinheins et al. 2023*) for adipic acid / water mixtures. Solid red line: model fit inside the concentration range covered by experimental data, reported as recommended values. Blue shading: fit parameters with 95 % confidence interval (CI). RMSE: root mean squared error. Markers: data used for fitting. **Note: the modelling is reliable only in the range of the experimental data. Therefore, for this compound, the modelling should be used only in the experimental data range.**

Comment: N/A

AC23: 4-ethylbenzoic acid

Dynarowicz 1994	**Graph (experimental points)**		
**C (M)**	**Molar fraction**	**logC (M)**	**σ (mN/m)**
9.897E-05	1.78E-06	**-4.00E+00**	72.6
2.481E-04	4.47E-06	**-3.61E+00**	72.4
4.904E-04	8.83E-06	**-3.31E+00**	72.0
7.968E-04	1.43E-05	**-3.10E+00**	71.0
1.010E-03	1.82E-05	**-3.00E+00**	69.5
1.511E-03	2.72E-05	**-2.82E+00**	65.9
2.018E-03	3.63E-05	**-2.70E+00**	62.5

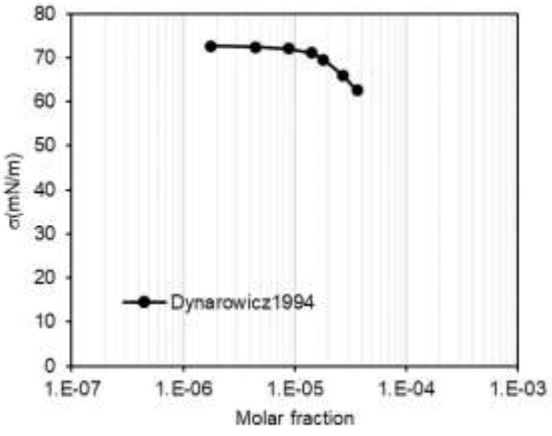

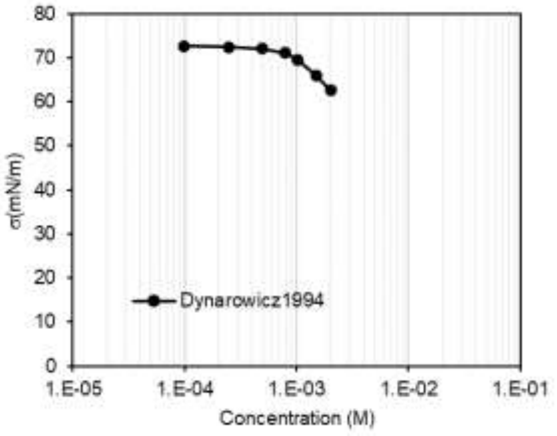

Fig. S23: Experimental surface tension data for 4-Ethylbenzoic acid / water mixtures.

Comment: N/A

AC24: cyclohexylpropanoic acid

Skauge et al. 1983	**Graph (experimental points)**		
**C (M)**	**Molar fraction**	**logC (M)**	**σ (mN/m)**
4.1E-04	7.40E-06	**-3.39E+00**	69.3
4.9E-04	8.87E-06	**-3.31E+00**	67.8
9.0E-04	1.61E-05	**-3.05E+00**	61.8
1.3E-03	2.30E-05	**-2.89E+00**	58.1
1.8E-03	3.16E-05	**-2.76E+00**	54.2
2.0E-03	3.61E-05	**-2.70E+00**	53.0
2.3E-03	4.06E-05	**-2.65E+00**	52.3
2.4E-03	4.41E-05	**-2.61E+00**	50.0
3.1E-03	5.61E-05	**-2.51E+00**	47.5
3.7E-03	6.75E-05	**-2.43E+00**	45.3
4.9E-03	8.75E-05	**-2.31E+00**	41.9

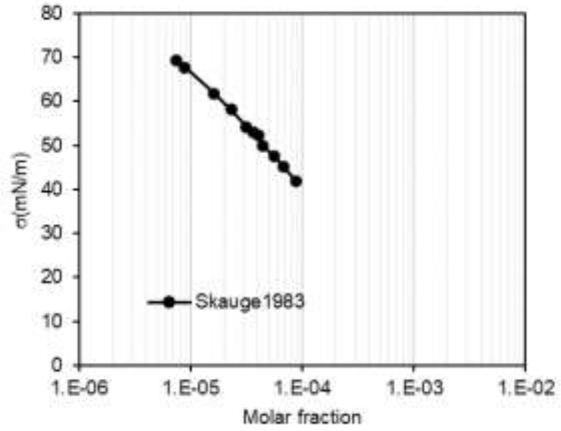

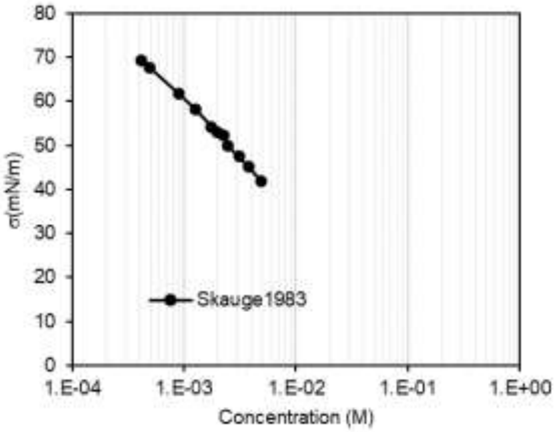

Fig. S24: Experimental surface tension data for cyclohexylpropanoic acid / water mixtures.

Comment: N/A

AC25: pelargonic acid (nonanoic acid)

Lunkenheimer et al. 2003 **Graph (experimental points)**			Fu et al. 2015 **Graph (experimental points)**			Badban et al. 2017 **Graph (experimental points)**		
**C (M)**	**Molar fraction**	**σ (mN/m)**	**C (M)**	**Molar fraction**	**σ (mN/m)**	**C (M)**	**Molar fraction**	**σ (mN/m)**
**2.92E-06**	5.25E-08	72.6	**3.00E-05**	5.40E-07	67.1	**2.95E-05**	5.31E-07	70.6
**4.91E-06**	8.84E-08	72.6	**1.00E-04**	1.80E-06	61.4	**3.97E-05**	7.15E-07	70.1
**6.82E-06**	1.23E-07	72.5	**5.00E-04**	9.00E-06	39.8	**4.90E-05**	8.82E-07	68.9
**9.80E-06**	1.76E-07	72.1	**1.00E-03**	1.80E-05	36.8	**5.90E-05**	1.06E-06	67.7
**1.96E-05**	3.52E-07	71.7	**1.50E-03**	2.70E-05	36.0	**7.99E-05**	1.44E-06	65.0
**2.97E-05**	5.35E-07	71.1	**2.00E-03**	3.60E-05	36.0	**1.00E-04**	1.80E-06	63.0
**4.95E-05**	8.92E-07	69.9	**3.00E-03**	5.40E-05	36.3	**1.17E-04**	2.11E-06	60.7
**6.81E-05**	1.23E-06	67.6	**5.00E-03**	9.01E-05	35.9	**1.40E-04**	2.52E-06	59.9
**9.92E-05**	1.78E-06	63.9				**1.60E-04**	2.88E-06	58.2
**1.49E-04**	2.69E-06	60.1				**1.99E-04**	3.58E-06	55.2
**2.01E-04**	3.62E-06	56.6				**2.46E-04**	4.43E-06	52.9
**3.03E-04**	5.45E-06	52.7				**2.97E-04**	5.35E-06	50.6
**5.00E-04**	9.00E-06	45.5				**3.52E-04**	6.34E-06	48.2
**6.88E-04**	1.24E-05	40.4				**4.00E-04**	7.20E-06	46.5
**9.92E-04**	1.79E-05	34.7				**4.50E-04**	8.10E-06	43.7
						**4.84E-04**	8.71E-06	42.6
						**5.50E-04**	9.90E-06	39.8
						**5.99E-04**	1.08E-05	38.1
						**6.40E-04**	1.15E-05	36.7
						**6.94E-04**	1.25E-05	35.2
						**7.56E-04**	1.36E-05	34.6
						**7.90E-04**	1.42E-05	33.8
						**8.40E-04**	1.51E-05	33.0
						**8.96E-04**	1.61E-05	33.8
						**9.96E-04**	1.79E-05	34.0
						**1.18E-03**	2.12E-05	34.0
						**1.50E-03**	2.70E-05	34.2
						**1.80E-03**	3.24E-05	34.0

Recommended		
C (M)	***Molar fraction***	***σ (mN/m)***
2.92E-06	*5.25E-08*	*71.9*
4.32E-06	*7.77E-08*	*71.8*
6.39E-06	*1.15E-07*	*71.7*
9.45E-06	*1.70E-07*	*71.6*
1.40E-05	*2.52E-07*	*71.3*
2.07E-05	*3.73E-07*	*70.8*
3.06E-05	*5.52E-07*	*70.0*
4.53E-05	*8.16E-07*	*68.6*
6.71E-05	*1.21E-06*	*66.6*
9.93E-05	*1.79E-06*	*63.6*
1.47E-04	*2.65E-06*	*59.4*
2.18E-04	*3.92E-06*	*54.2*
3.22E-04	*5.79E-06*	*48.5*
4.76E-04	*8.58E-06*	*43.0*
7.05E-04	*1.27E-05*	*38.2*

1.04E-03	*1.88E-05*	*34.7*
1.54E-03	*2.78E-05*	*32.2*
2.28E-03	*4.11E-05*	*30.5*
3.38E-03	*6.09E-05*	*29.5*
5.00E-03	*9.01E-05*	*28.9*

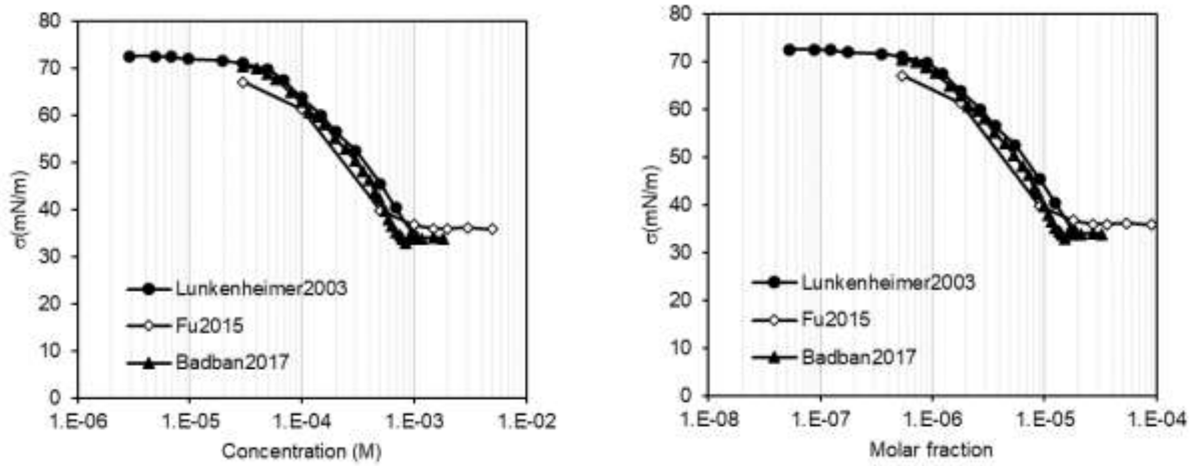

Fig. S25 (a): Comparison of the experimental surface tension data for pelargonic acid / water mixtures.

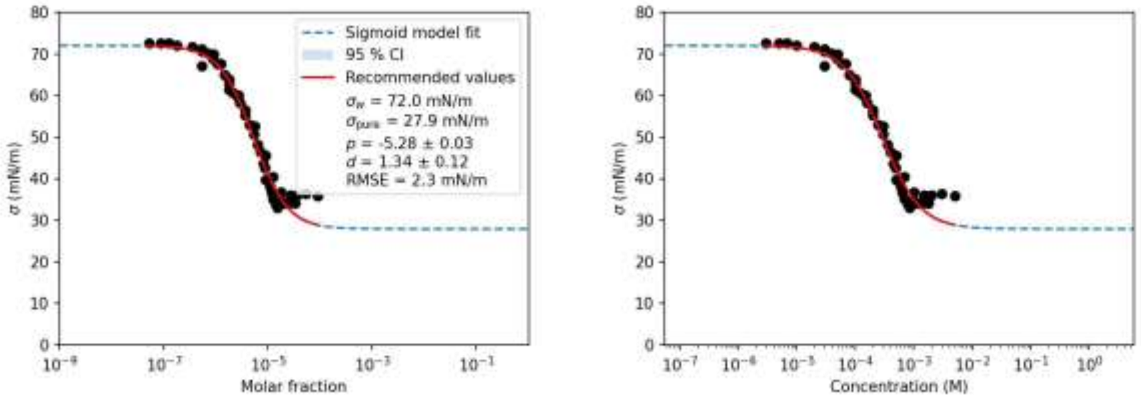

Fig. S25 (b): Surface tension fit with the Sigmoid model (*Kleinheins et al. 2023*) for pelargonic acid / water mixtures. Solid red line: model fit inside the concentration range covered by experimental data, reported as recommended values. Blue shading: fit parameters with 95 % confidence interval (CI). RMSE: root mean squared error. Markers: data used for fitting.

Comment:

Lunkenheimer et al. 2003: aqueous solutions of pelargonic acid in 0.005M HCl.

AC26: 4-propylbenzoic acid

Dynarowicz 1994	**Graph (experimental points)**		
**C (M)**	**Molar fraction**	**logC (M)**	**σ (mN/m)**
1.01E-05	1.82E-07	**-5.00E+00**	72.7
2.48E-05	4.47E-07	**-4.61E+00**	72.7
4.96E-05	8.92E-07	**-4.30E+00**	72.6
9.90E-05	1.78E-06	**-4.00E+00**	72.5
1.54E-04	2.78E-06	**-3.81E+00**	72.2
2.46E-04	4.42E-06	**-3.61E+00**	71.3
3.21E-04	5.78E-06	**-3.49E+00**	70.1

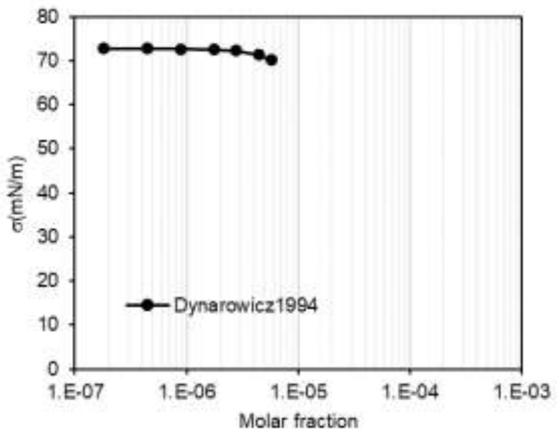

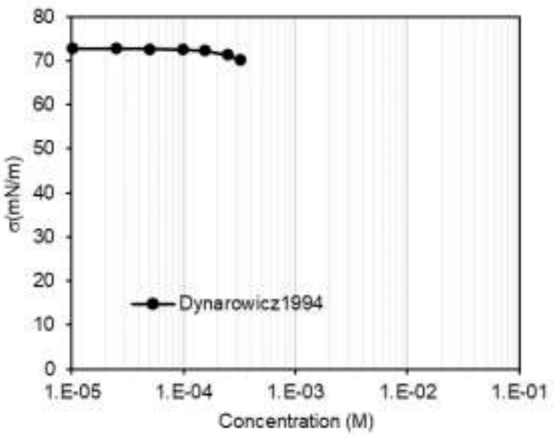

Fig. S26: Experimental surface tension data for 4-propylbenzoic acid / water mixtures.

Comment: N/A

AC27: phthalic acid (benzene-1,2-dicarboxylic acid)

Aumann et al. 2010 **Graph (experimental points)**		
**C (M)**	**Molar fraction**	**σ (mN/m)**
1.04E-04	1.87E-06	72.0
2.07E-04	3.73E-06	71.9
4.23E-04	7.61E-06	72.0
8.27E-04	1.49E-05	71.9
2.08E-03	3.74E-05	71.8
5.11E-03	9.20E-05	71.6
1.04E-02	1.87E-04	71.1
2.08E-02	3.75E-04	70.4
2.88E-02	5.20E-04	69.8
4.08E-02	7.37E-04	68.9

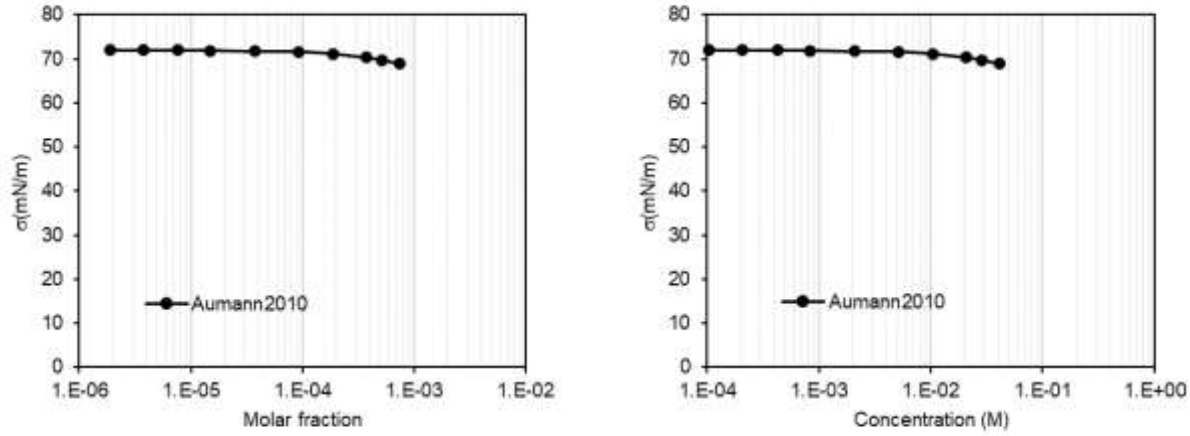

Fig. S27: Experimental surface tension data for phthalic acid / water mixtures.

Comment: N/A

AC28: cyclohexylbutanoic acid

Skauge et al. 1983	**Graph (experimental points)**		
**C (M)**	**Molar fraction**	**logC (M)**	**σ (mN/m)**
1.40E-04	2.52E-06	**-3.85E+00**	69.8
2.14E-04	3.85E-06	**-3.67E+00**	68.0
2.38E-04	4.29E-06	**-3.62E+00**	67.0
2.48E-04	4.47E-06	**-3.61E+00**	66.1
4.20E-04	7.56E-06	**-3.38E+00**	59.1
4.55E-04	8.18E-06	**-3.34E+00**	57.6
5.56E-04	1.00E-05	**-3.26E+00**	54.9
7.29E-04	1.31E-05	**-3.14E+00**	51.1
1.12E-03	2.02E-05	**-2.95E+00**	45.0
1.40E-04	2.52E-06	**-3.85E+00**	69.8
2.14E-04	3.85E-06	**-3.67E+00**	68.0
2.38E-04	4.29E-06	**-3.62E+00**	67.0
2.48E-04	4.47E-06	**-3.61E+00**	66.1

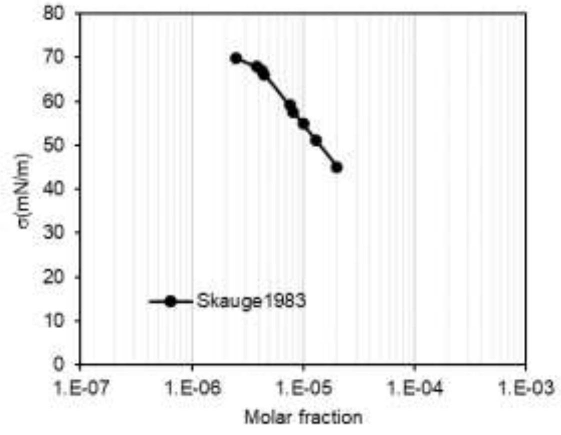

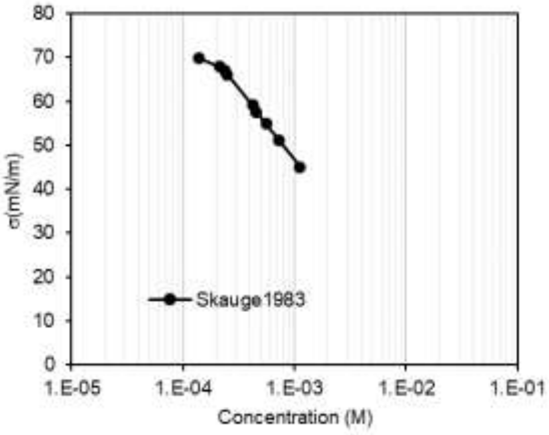

Fig. S28: Experimental surface tension data for cyclohexylbutanoic acid / water mixtures.

Comment: N/A

AC29: capric acid (decanoic acid)

Lunkenheimer et al. 2003 **Graph (experimental points)**			Casandra et al. 2016 **Graph (experimental points)**				***Recommended***		
**C (M)**	**Molar fraction**	**σ (mN/m)**	**C (M)**	**Molar fraction**	**C (mol/cm^3)**	**σ (mN/m)**	***C (M)***	***Molar fraction***	***σ (mN/m)***
**9.96E-07**	1.79E-08	72.5	2.01E-05	3.62E-07	**2.01E-08**	71.3	*9.94E-07*	*1.79E-08*	*72.0*
**1.92E-06**	3.46E-08	72.3	2.42E-05	4.36E-07	**2.42E-08**	71.0	*1.31E-06*	*2.37E-08*	*71.9*
**2.92E-06**	5.25E-08	72.4	3.04E-05	5.47E-07	**3.04E-08**	68.7	*1.74E-06*	*3.13E-08*	*71.9*
**4.91E-06**	8.85E-08	72.1	4.00E-05	7.20E-07	**4.00E-08**	64.9	*2.29E-06*	*4.13E-08*	*71.9*
**6.82E-06**	1.23E-07	71.9	5.03E-05	9.05E-07	**5.03E-08**	62.3	*3.03E-06*	*5.46E-08*	*71.8*
**8.76E-06**	1.58E-07	71.6	6.12E-05	1.10E-06	**6.12E-08**	59.9	*4.01E-06*	*7.21E-08*	*71.7*
**9.81E-06**	1.77E-07	71.4	7.07E-05	1.27E-06	**7.07E-08**	57.4	*5.29E-06*	*9.53E-08*	*71.5*
**1.18E-05**	2.12E-07	70.6	8.50E-05	1.53E-06	**8.50E-08**	54.6	*7.00E-06*	*1.26E-07*	*71.2*
**1.36E-05**	2.45E-07	70.4	1.01E-04	1.82E-06	**1.01E-07**	52.1	*9.25E-06*	*1.66E-07*	*70.9*
**1.54E-05**	2.78E-07	69.3	1.22E-04	2.19E-06	**1.22E-07**	49.3	*1.22E-05*	*2.20E-07*	*70.3*
**1.75E-05**	3.15E-07	68.2	1.50E-04	2.71E-06	**1.50E-07**	45.9	*1.61E-05*	*2.91E-07*	*69.5*
**1.94E-05**	3.49E-07	67.5	1.83E-04	3.29E-06	**1.83E-07**	42.4	*2.13E-05*	*3.84E-07*	*68.3*
**2.43E-05**	4.38E-07	66.1					*2.82E-05*	*5.07E-07*	*66.6*
**2.99E-05**	5.37E-07	64.4					*3.73E-05*	*6.71E-07*	*64.2*
**3.50E-05**	6.30E-07	62.6					*4.92E-05*	*8.86E-07*	*61.2*
**4.02E-05**	7.23E-07	61.1					*6.50E-05*	*1.17E-06*	*57.5*
**5.04E-05**	9.07E-07	58.9					*8.60E-05*	*1.55E-06*	*53.2*
**5.98E-05**	1.08E-06	56.3					*1.14E-04*	*2.04E-06*	*48.7*
**7.10E-05**	1.28E-06	54.3					*1.50E-04*	*2.70E-06*	*44.3*
**9.89E-05**	1.78E-06	49.0					*1.98E-04*	*3.57E-06*	*40.5*
**1.47E-04**	2.65E-06	43.5							
**1.98E-04**	3.57E-06	38.5							

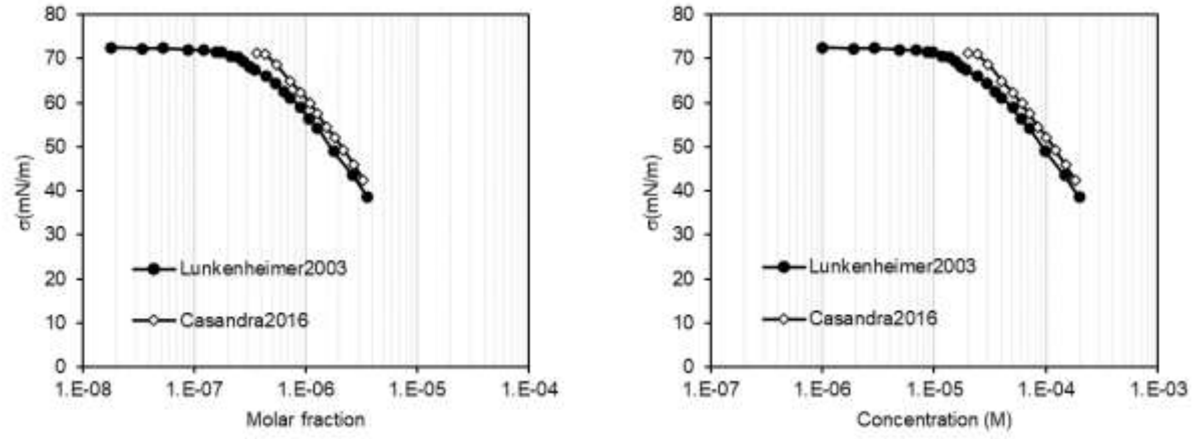

Fig. S29 (a): Comparison of the experimental surface tension data for capric acid / water mixtures.

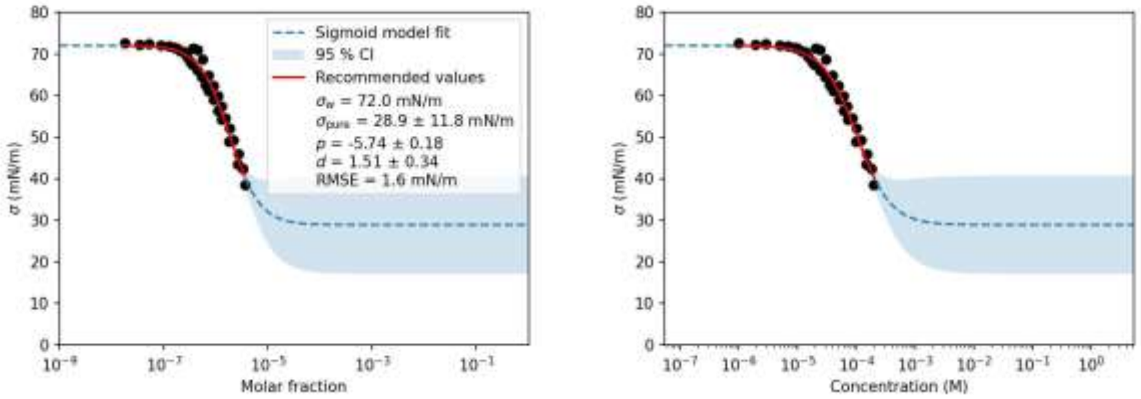

Fig. S29 (b): Surface tension fit with the Sigmoid model (*Kleinheins et al. 2023*) for capric acid / water mixtures. Solid red line: model fit inside the concentration range covered by experimental data, reported as recommended values. Blue shading: fit parameters with 95 % confidence interval (CI). RMSE: root mean squared error. Markers: data used for fitting.

Comment:

Lunkenheimer et al. 2003: aqueous solutions of capric acid in 0.005M HCl.

AC30: 4-butylbenzoic acid

Dynarowicz 1994	**Graph (experimental points)**		
**C (M)**	**Molar fraction**	**logC (M)**	**σ (mN/m)**
1.00E-05	1.80E-07	**-5.00E+00**	72.7
2.48E-05	4.47E-07	**-4.61E+00**	72.7
5.01E-05	9.01E-07	**-4.30E+00**	72.5
7.72E-05	1.39E-06	**-4.11E+00**	72.2
1.00E-04	1.80E-06	**-4.00E+00**	71.0
1.24E-04	2.24E-06	**-3.91E+00**	68.7
1.59E-04	2.86E-06	**-3.80E+00**	66.0

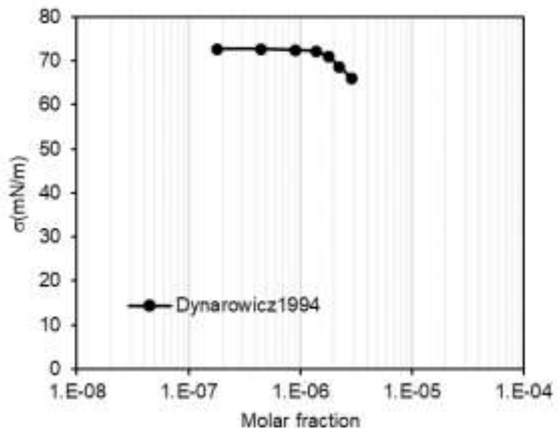

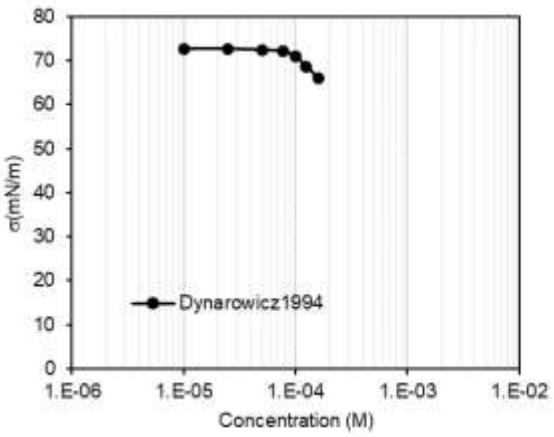

Fig. S30: Experimental surface tension data for 4-butylbenzoic acid / water mixtures.

Comment: N/A

AC31: pinonic acid (3-acetyl-2,2-dimethylcyclobutyl)acetic acid)

Shulman et al. 1996 **Graph (experimental points)**			
**C (M)**	**Molar fraction**	**-Δσ/σ (mN/m)**	**σ (mN/m)**
**9.27E-04**	1.67E-05	**0.010**	72.1
**2.01E-03**	3.61E-05	**0.026**	71.0
**8.49E-03**	1.53E-04	**0.088**	66.9
**1.34E-02**	2.42E-04	**0.115**	65.3
**2.00E-02**	3.61E-04	**0.140**	63.9
**2.56E-02**	4.63E-04	**0.159**	62.8
**3.76E-02**	6.80E-04	**0.189**	61.2
**9.27E-04**	1.67E-05	**0.010**	72.1
**2.01E-03**	3.61E-05	**0.026**	71.0
**8.49E-03**	1.53E-04	**0.088**	66.9
**1.34E-02**	2.42E-04	**0.115**	65.3
**2.00E-02**	3.61E-04	**0.140**	63.9
**2.56E-02**	4.63E-04	**0.159**	62.8
**3.76E-02**	6.80E-04	**0.189**	61.2

Tuckermann et al. 2004 **Graph (experimental points)**			
**C (M)**	**Molar fraction**	**C (mg/mL)**	**σ (mN/m)**
5.27E-06	9.48E-08	**9.70E-04**	72.7
1.03E-05	1.86E-07	**1.90E-03**	72.6
2.17E-05	3.91E-07	**4.00E-03**	72.4
4.13E-05	7.43E-07	**7.60E-03**	72.6
8.14E-05	1.47E-06	**1.50E-02**	72.2
1.68E-04	3.03E-06	**3.10E-02**	72.3
3.25E-04	5.85E-06	**5.99E-02**	72.0
6.95E-04	1.25E-05	**1.28E-01**	71.1
1.36E-03	2.44E-05	**2.50E-01**	70.9
2.65E-03	4.78E-05	**4.89E-01**	68.9
5.38E-03	9.69E-05	**9.91E-01**	66.2
1.09E-02	1.97E-04	**2.01E+00**	62.9
2.71E-02	4.91E-04	**5.00E+00**	57.7

Hyvarinen et al. 2006 **Table**		
**C (M)**	**Molar fraction**	**σ (mN/m)**
1.11E-03	**2.00E-05**	69.5
2.22E-03	**4.00E-05**	66.8
6.11E-03	**1.10E-04**	64.4
9.99E-03	**1.80E-04**	61.4
1.33E-02	**2.40E-04**	61.2
1.88E-02	**3.40E-04**	59
2.27E-02	**4.10E-04**	57.2
2.77E-02	**5.00E-04**	56.3
3.21E-02	**5.80E-04**	55.8
3.59E-02	**6.50E-04**	56.2
4.58E-02	**8.30E-04**	53.9

Topping et al. 2007 **Graph (experimental points)**			
**C (M)**	**Molar fraction**	**Mass fraction**	**σ (mN/m)**
4.94E-04	8.90E-06	**9.11E-05**	72.1
1.95E-03	3.51E-05	**3.59E-04**	69.5
2.32E-03	4.17E-05	**4.27E-04**	69.0
3.30E-03	5.94E-05	**6.08E-04**	66.7
4.26E-03	7.68E-05	**7.85E-04**	66.6
5.92E-03	1.07E-04	**1.09E-03**	66.2
7.82E-03	1.41E-04	**1.44E-03**	65.0
8.25E-03	1.49E-04	**1.52E-03**	64.8
1.27E-02	2.29E-04	**2.34E-03**	62.0
1.56E-02	2.81E-04	**2.87E-03**	61.4
1.91E-02	3.45E-04	**3.52E-03**	59.3
3.12E-02	5.64E-04	**5.74E-03**	57.5

Tuckermann et al. 2007 **Graph (experimental points)**			
**C (M)**	**Molar fraction**	**C (mg/mL)**	**σ (mN/m)**
5.43E-05	9.77E-07	**1.00E-02**	72.0
5.43E-04	9.77E-06	**1.00E-01**	71.5
2.70E-03	4.86E-05	**5.00E-01**	69.0
5.40E-03	9.73E-05	**1.00E+00**	67.0
1.00E-02	1.80E-04	**2.00E+00**	63.0

Varga et al. 2007 **Table**				***Recommended***		
**C (M)**	**Molar fraction**	**Mass fraction (%)**	**σ (mN/m)**	***C (M)***	***Molar fraction***	***σ (mN/m)***
5.43E-03	9.78E-05	**0.1**	67	*5.27E-06*	*9.48E-08*	*72.0*
2.72E-02	4.91E-04	**0.5**	58	*8.49E-06*	*1.53E-07*	*72.0*
				1.37E-05	*2.46E-07*	*72.0*
				2.21E-05	*3.97E-07*	*72.0*
				3.56E-05	*6.41E-07*	*71.9*
				5.74E-05	*1.03E-06*	*71.9*
				9.26E-05	*1.67E-06*	*71.8*
				1.49E-04	*2.69E-06*	*71.8*
				2.41E-04	*4.33E-06*	*71.6*
				3.88E-04	*6.99E-06*	*71.4*
				6.26E-04	*1.13E-05*	*71.1*
				1.01E-03	*1.82E-05*	*70.6*
				1.63E-03	*2.93E-05*	*69.9*
				2.62E-03	*4.72E-05*	*68.9*
				4.23E-03	*7.61E-05*	*67.6*
				6.81E-03	*1.23E-04*	*65.8*
				1.10E-02	*1.98E-04*	*63.6*
				1.77E-02	*3.19E-04*	*61.3*
				2.85E-02	*5.15E-04*	*58.9*
				4.58E-02	*8.30E-04*	*56.8*

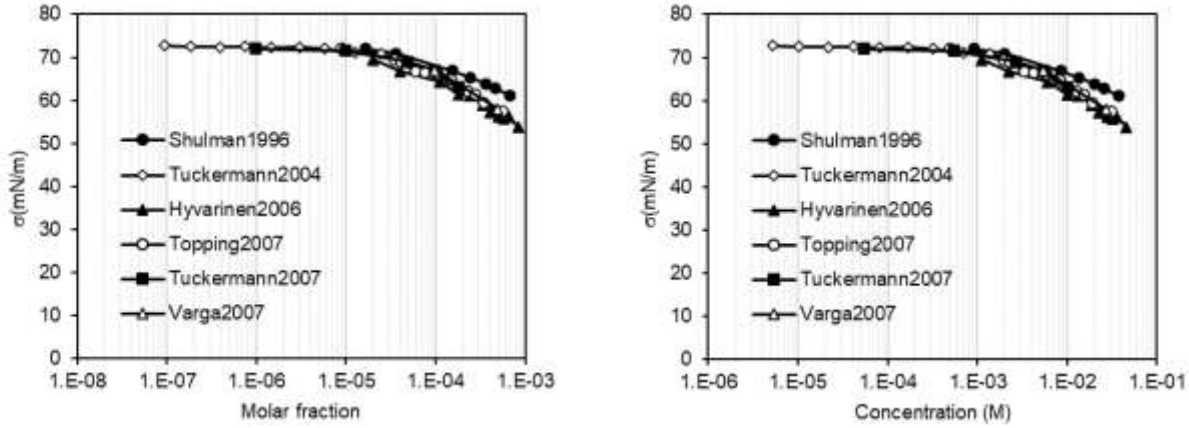

Fig. S31 (a): Comparison of the experimental surface tension data for pinonic acid / water mixtures.

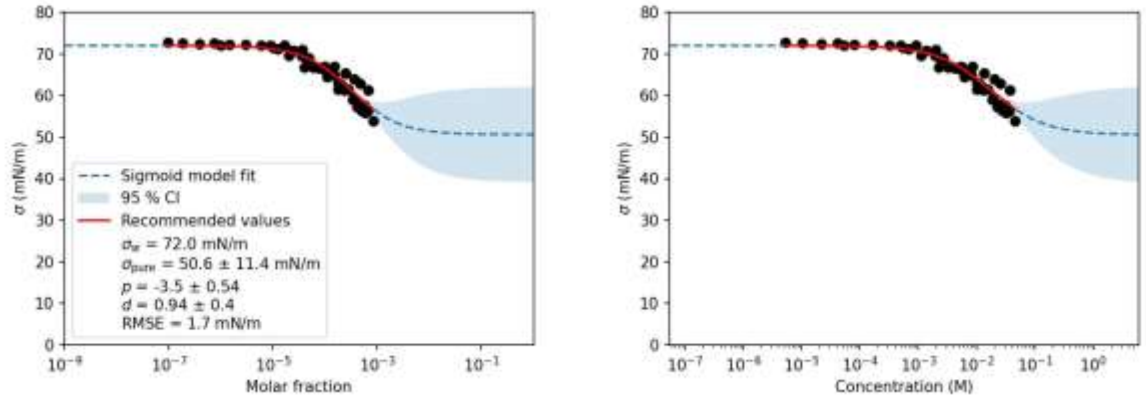

Fig. S31 (b): Surface tension fit with the Sigmoid model (*Kleinheins et al. 2023*) for pinonic acid / water mixtures. Solid red line: model fit inside the concentration range covered by experimental data, reported as recommended values. Blue shading: fit parameters with 95 % confidence interval (CI). RMSE: root mean squared error. Markers: data used for fitting.

Comment:

Shulman et al. 1996: the surface tension has been calculated assuming a surface tension of water at 72.8 mN m^{-1}

AC32: undecylic acid (undecanoic acid)

Lunkenheimer et al. 2003 **Graph (experimental points)**		
**C (M)**	**Molar fraction**	**σ (mN/m)**
**7.01E-07**	1.26E-08	72.4
**9.85E-07**	1.77E-08	72.4
**1.46E-06**	2.63E-08	72.4
**1.68E-06**	3.02E-08	72.4
**2.95E-06**	5.32E-08	71.9
**3.96E-06**	7.13E-08	71.9
**4.92E-06**	8.85E-08	70.6
**5.77E-06**	1.04E-07	69.9
**7.93E-06**	1.43E-07	67.5
**9.95E-06**	1.79E-07	65.5
**1.50E-05**	2.70E-07	61.4
**1.97E-05**	3.55E-07	58.2
**2.97E-05**	5.35E-07	53.6
**3.49E-05**	6.27E-07	51.0
**4.00E-05**	7.19E-07	49.9
**4.48E-05**	8.06E-07	47.6
**5.14E-05**	9.25E-07	45.6
**6.03E-05**	1.09E-06	42.6
**8.11E-05**	1.46E-06	38.4

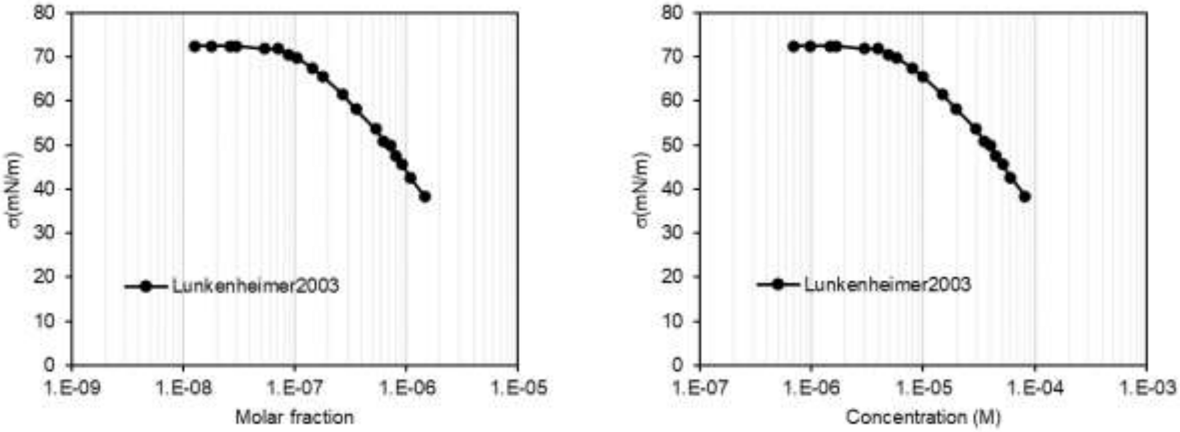

Fig. S32: Experimental surface tension data for undecylic acid /water mixtures.

Comment:

Lunkenheimer et al. 2003: aqueous solutions of undecylic acid in 0.005M HCl.

AC33: azelaic acid (nonanedioic acid)

Tuckermann et al. 2004 **Graph (experimental points)**				Aumann et al. 2010 **Graph (experimental points)**			***Recommended***		
**C (M)**	**Molar fraction**	**C (mg/mL)**	**σ (mN/m)**	**C (M)**	**Molar fraction**	**σ (mN/m)**	***C (M)***	***Molar fraction***	***σ (mN/m)***
5.31E-06	9.56E-08	**1.00E-03**	72.8	**2.00E-04**	3.60E-06	71.7	*5.31E-06*	*9.56E-08*	*72.0*
1.06E-05	1.91E-07	**2.00E-03**	72.8	**5.00E-04**	9.00E-06	71.1	*8.32E-06*	*1.50E-07*	*72.0*
2.13E-05	3.83E-07	**4.00E-03**	73.2	**1.20E-03**	2.16E-05	69.8	*1.30E-05*	*2.35E-07*	*71.9*
4.25E-05	7.65E-07	**8.00E-03**	72.8	**2.50E-03**	4.50E-05	67.7	*2.04E-05*	*3.67E-07*	*71.9*
9.03E-05	1.63E-06	**1.70E-02**	72.84	**5.00E-03**	9.01E-05	65.0	*3.20E-05*	*5.75E-07*	*71.9*
1.75E-04	3.16E-06	**3.30E-02**	72.7	**7.30E-03**	1.32E-04	62.9	*5.01E-05*	*9.01E-07*	*71.8*
3.51E-04	6.31E-06	**6.60E-02**	72.3	**9.50E-03**	1.71E-04	61.2	*7.84E-05*	*1.41E-06*	*71.7*
7.12E-04	1.28E-05	**1.34E-01**	70.7				*1.23E-04*	*2.21E-06*	*71.6*
1.39E-03	2.51E-05	**2.62E-01**	67.6				*1.92E-04*	*3.46E-06*	*71.4*
2.87E-03	5.18E-05	**5.41E-01**	63.5				*3.01E-04*	*5.42E-06*	*71.2*
5.54E-03	9.97E-05	**1.04E+00**	58.8				*4.72E-04*	*8.50E-06*	*70.8*
1.08E-02	1.95E-04	**2.04E+00**	54.3				*7.39E-04*	*1.33E-05*	*70.2*
2.67E-02	4.82E-04	**5.02E+00**	45.8				*1.16E-03*	*2.08E-05*	*69.4*
							1.81E-03	*3.26E-05*	*68.1*
							2.84E-03	*5.11E-05*	*66.4*
							4.45E-03	*8.01E-05*	*64.0*
							6.96E-03	*1.25E-04*	*60.8*
							1.09E-02	*1.96E-04*	*56.7*
							1.71E-02	*3.08E-04*	*51.7*
							2.67E-02	*4.82E-04*	*45.9*

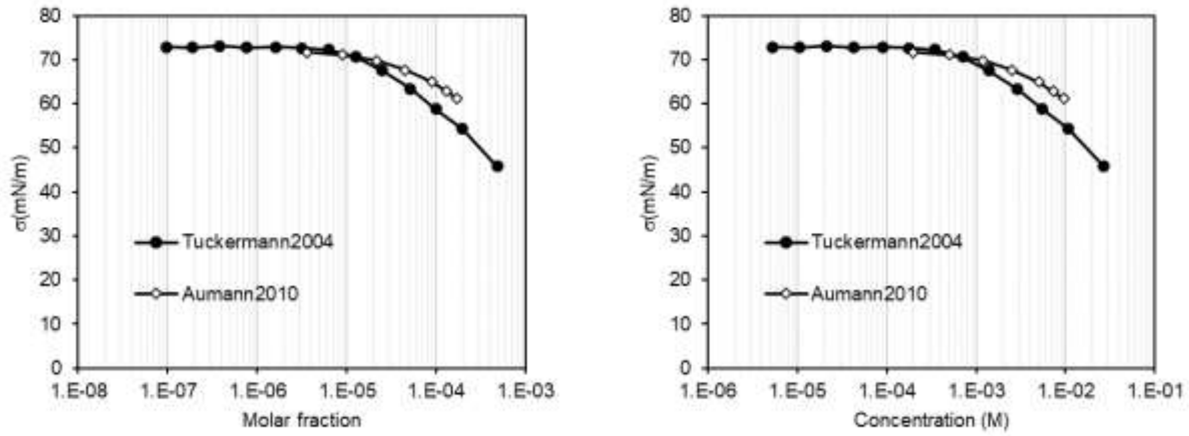

Fig. S33 (a): Comparison of the experimental surface tension data for azelaic acid / water mixtures.

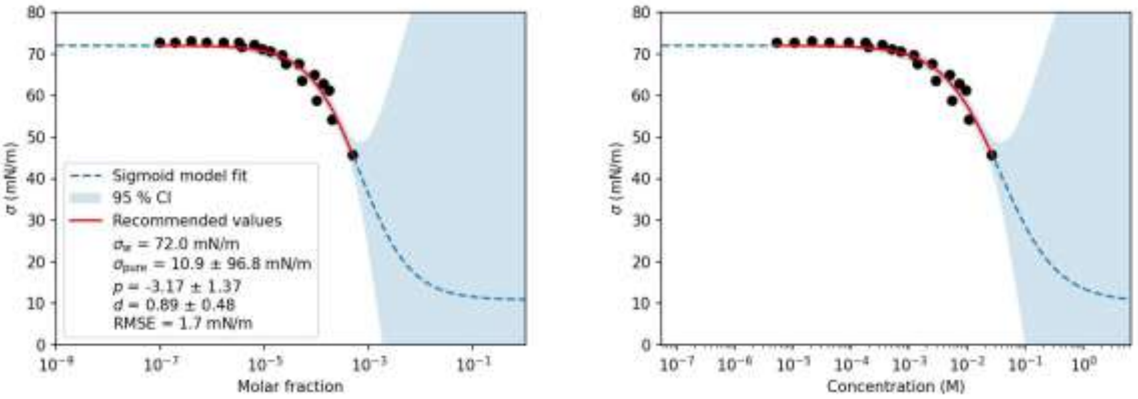

Fig. S33 (b): Surface tension fit with the Sigmoid model (*Kleinheins et al. 2023*) for azelaic acid / water mixtures. Solid red line: model fit inside the concentration range covered by experimental data, reported as recommended values. Blue shading: fit parameters with 95 % confidence interval (CI). RMSE: root mean squared error. Markers: data used for fitting. **Note: the modelling is reliable only in the range of the experimental data. Therefore, for this compound, the modelling should be used only in the experimental data range.**

Comment: N/A

AC34: citric acid (2-hydroxypropane-1,2,3-tricarboxylic acid)

Topping et al. 2007 **Graph (experimental points)**				Varga et al. 2007 **Table**				Mahiuddin et al. 2008 **Graph (experimental points)**		
**C (M)**	**Molar fraction**	**Mass fraction**	**σ (mN/m)**	**C (M)**	**Molar fraction**	**Mass fraction (%)**	**σ (mN/m)**	**C (M)**	**Molar fraction**	**σ (mN/m)**
0.00E+00	0.00E+00	**0.00E+00**	72.0	5.20E-03	9.36E-05	**0.1**	71	**0.00E+00**	0.00E+00	72.1
1.21E-02	2.18E-04	**2.32E-03**	70.4	2.60E-02	4.69E-04	**0.5**	71	**3.00E-02**	5.42E-04	71.9
1.25E-01	2.28E-03	**2.38E-02**	70.5	5.22E-02	9.44E-04	**1**	71	**2.50E-01**	4.61E-03	70.2
2.93E-01	5.42E-03	**5.50E-02**	69.1	2.65E-01	4.90E-03	**5**	69	**5.00E-01**	9.46E-03	69.0
1.02E+00	2.04E-02	**1.82E-01**	66.2	5.40E-01	1.03E-02	**10**	68	**8.00E-01**	1.56E-02	67.4
2.39E+00	5.61E-02	**3.88E-01**	65.2	1.77E+00	3.85E-02	**30**	65	**1.00E+00**	1.99E-02	66.5
				2.47E+00	5.85E-02	**40**	65	**1.25E+00**	2.56E-02	66.0
								**1.50E+00**	3.16E-02	65.2
								**2.00E+00**	4.47E-02	65.2
								**2.50E+00**	5.95E-02	64.9
								**3.00E+00**	7.63E-02	65.0

Boyer et al. 2016 **Table**			Zarska et al. 2016 **Table**				***Recommended***		
**C (M)**	**Molar fraction**	**σ (mN/m)**	**C (M)**	**Molar fraction**	**Mass fraction**	**σ (mN/m)**	***C (M)***	***Molar fraction***	***σ (mN/m)***
2.85E-01	**5.28E-03**	71.5	0.00E+00	0.00E+00	**0.00E+00**	71.61	*5.20E-03*	*9.36E-05*	*72.0*
5.16E-01	**9.78E-03**	70.8	1.02E-01	1.86E-03	**1.95E-02**	70.84	*7.58E-03*	*1.36E-04*	*71.9*
1.09E+00	**2.19E-02**	69.7	2.73E-01	5.04E-03	**5.13E-02**	70.08	*1.10E-02*	*1.99E-04*	*71.9*
2.15E+00	**4.89E-02**	68.3	5.35E-01	1.02E-02	**9.88E-02**	69.48	*1.61E-02*	*2.90E-04*	*71.9*
2.43E+00	**5.73E-02**	68.3	8.08E-01	1.58E-02	**1.46E-01**	68.67	*2.34E-02*	*4.23E-04*	*71.8*
2.79E+00	**6.88E-02**	68	1.10E+00	2.21E-02	**1.94E-01**	67.94	*3.41E-02*	*6.17E-04*	*71.7*
3.22E+00	**8.43E-02**	68	1.42E+00	2.97E-02	**2.46E-01**	67.30	*4.97E-02*	*8.99E-04*	*71.6*
3.96E+00	**1.16E-01**	67.8	1.78E+00	3.87E-02	**3.00E-01**	66.67	*7.23E-02*	*1.31E-03*	*71.4*
			2.09E+00	4.72E-02	**3.46E-01**	66.38	*1.05E-01*	*1.91E-03*	*71.1*
			2.44E+00	5.76E-02	**3.95E-01**	66.07	*1.53E-01*	*2.79E-03*	*70.7*
			2.87E+00	7.18E-02	**4.52E-01**	65.82	*2.21E-01*	*4.06E-03*	*70.2*
			3.25E+00	8.58E-02	**5.00E-01**	65.49	*3.19E-01*	*5.92E-03*	*69.7*
			3.68E+00	1.03E-01	**5.51E-01**	65.03	*4.58E-01*	*8.64E-03*	*69.0*
			3.91E+00	1.14E-01	**5.78E-01**	64.86	*6.55E-01*	*1.26E-02*	*68.3*
			4.06E+00	1.21E-01	**5.95E-01**	64.99	*9.28E-01*	*1.84E-02*	*67.7*
							1.30E+00	*2.68E-02*	*67.1*
							1.79E+00	*3.90E-02*	*66.6*
							2.42E+00	*5.69E-02*	*66.2*
							3.18E+00	*8.30E-02*	*65.9*
							4.06E+00	*1.21E-01*	*65.7*

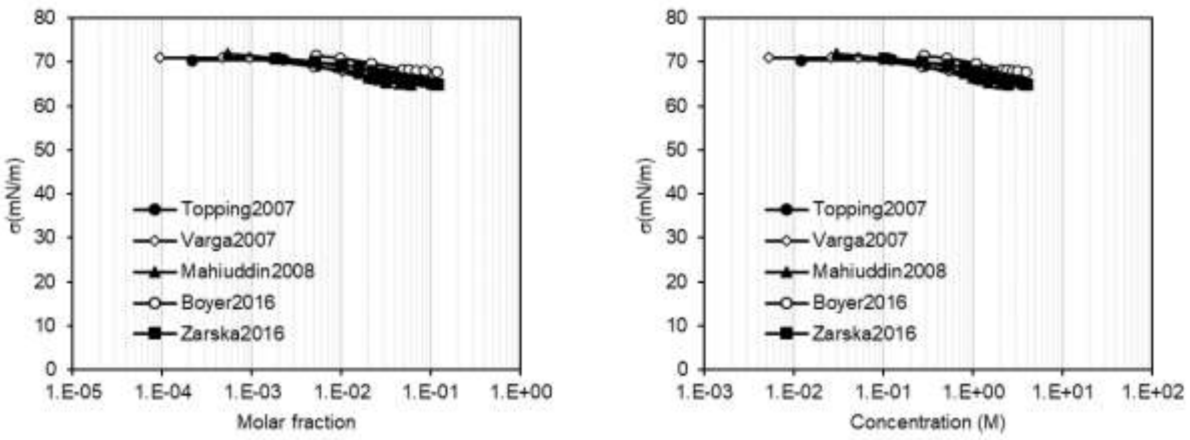

Fig. S34 (a): Comparison of the experimental surface tension data for citric acid / water mixtures.

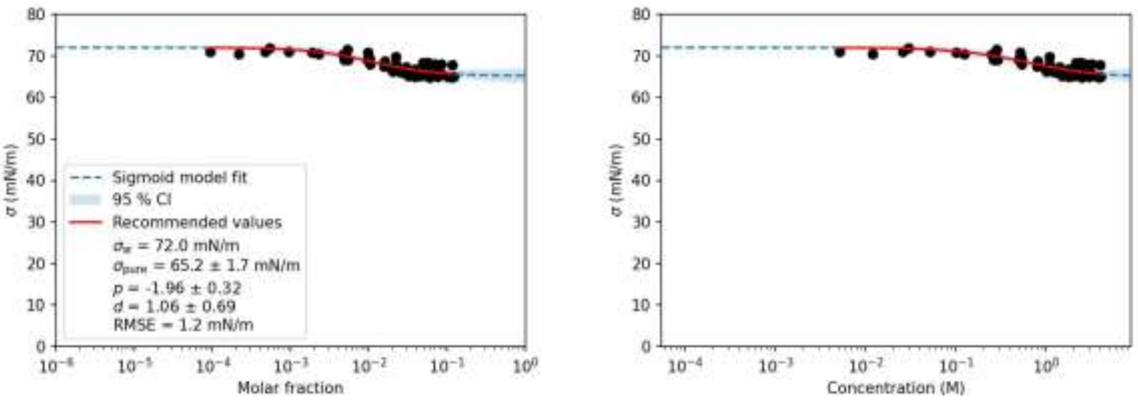

Fig. S34 (b): Surface tension fit with the Sigmoid model (*Kleinheins et al. 2023*) for citric acid / water mixtures. Solid red line: model fit inside the concentration range covered by experimental data, reported as recommended values. Blue shading: fit parameters with 95 % confidence interval (CI). RMSE: root mean squared error. Markers: data used for fitting.

Comment: N/A

AC35: 4-pentylbenzoic acid

Dynarowicz 1994	Graph (experimental points)		
**C (M)**	**Molar fraction**	**logC (M)**	**σ (mN/m)**
1.01E-05	1.82E-07	**-5.00E+00**	72.7
2.48E-05	4.47E-07	**-4.61E+00**	72.5
3.87E-05	6.96E-07	**-4.41E+00**	72.4
4.56E-05	8.21E-07	**-4.34E+00**	71.8
5.11E-05	9.20E-07	**-4.29E+00**	69.3

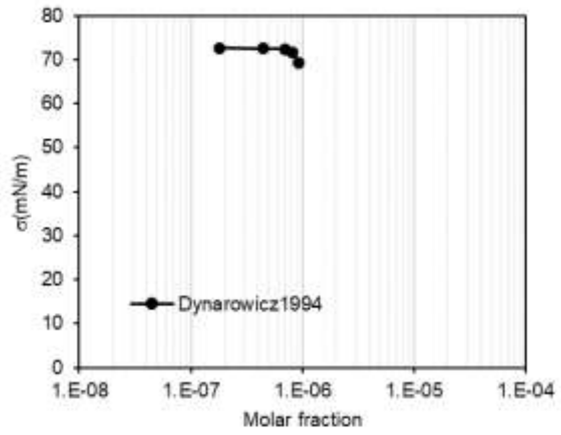

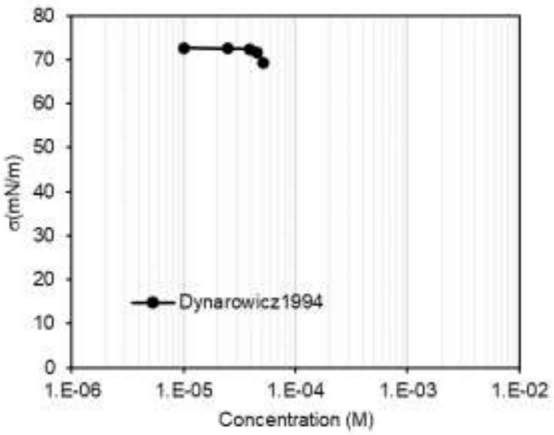

Fig. S35: Experimental surface tension data for 4-pentylbenzoic acid / water mixtures.

Comment: N/A

AC36: lauric acid (dodecanoic acid)

Prosser et al. 2004 **Graph (experimental points)**		
**C (M)**	**Molar fraction**	**σ (mN/m)**
**1.00E-06**	1.80E-08	71.4
**2.00E-06**	3.60E-08	66.3
**2.99E-06**	5.38E-08	62.4
**3.97E-06**	7.15E-08	58.8
**5.99E-06**	1.08E-07	52.7
**8.05E-06**	1.45E-07	48.2

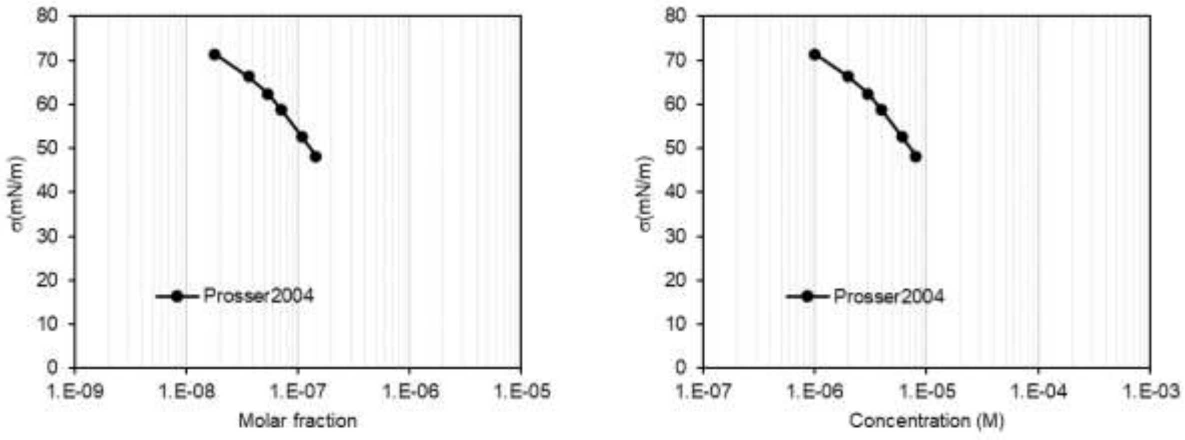

Fig. S36: Experimental surface tension data for lauric acid / water mixtures.

Comment: N/A

AC37: trimesic acid (benzene-1,3,5-tricarboxylic acid)

Aumann et al. 2010 **Graph (experimental points)**		
**C (M)**	**Molar fraction**	**σ (mN/m)**
**1.95E-04**	3.51E-06	71.9
**5.02E-04**	9.04E-06	71.9
**1.26E-03**	2.27E-05	71.8
**2.52E-03**	4.54E-05	71.7
**5.01E-03**	9.02E-05	71.5
**7.51E-03**	1.35E-04	71.4
**1.00E-02**	1.80E-04	71.3

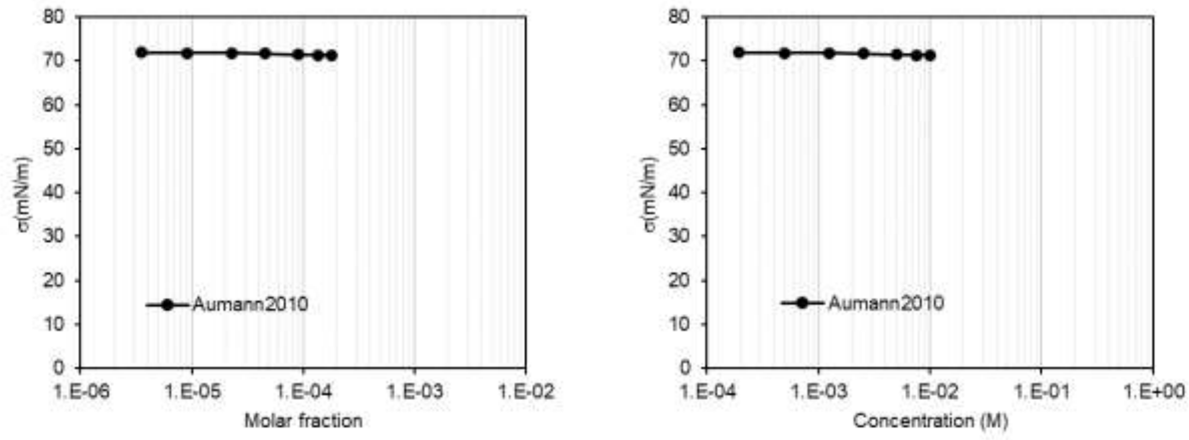

Fig. S37: Experimental surface tension data for trimesic acid / water mixtures.

Comment: N/A

AC38: oleic acid ((9Z)-octadec-9-enoic acid)

Pugh et al. 1984 **Graph (experimental points)**			Theander et al. 2001 **Graph (experimental points)**			Atrafi et al. 2016 **Graph (experimental points)**		
**C (M)**	**Molar fraction**	**σ (mN/m)**	**C (M)**	**Molar fraction**	**σ (mN/m)**	**C (M)**	**Molar fraction**	**σ (mN/m)**
**2.00E-05**	3.60E-07	46.3	**1.85E-08**	3.33E-10	71.0	**1.00E-05**	1.80E-07	44.1
**7.00E-05**	1.26E-06	39.3	**3.73E-08**	6.71E-10	71.0	**6.50E-05**	1.17E-06	38.1
**3.00E-04**	5.40E-06	37.7	**5.57E-08**	1.00E-09	70.8	**2.30E-04**	4.15E-06	37.1
			**9.25E-08**	1.67E-09	70.7	**1.00E-03**	1.80E-05	33.4
			**1.59E-07**	2.87E-09	70.5	**1.00E-02**	1.81E-04	31.2
			**2.54E-07**	4.57E-09	70.5			
			**4.09E-07**	7.37E-09	70.3			
			**6.60E-07**	1.19E-08	69.8			
			**1.04E-06**	1.87E-08	69.0			
			**1.70E-06**	3.05E-08	66.6			
			**2.77E-06**	4.98E-08	62.6			
			**4.39E-06**	7.90E-08	56.5			
			**7.12E-06**	1.28E-07	46.3			
			**1.13E-05**	2.03E-07	36.5			
			**1.83E-05**	3.29E-07	30.9			
			**2.90E-05**	5.22E-07	29.5			
			**4.65E-05**	8.37E-07	28.5			
			**7.45E-05**	1.34E-06	28.2			
			**1.40E-04**	2.52E-06	28.3			
			**2.25E-04**	4.04E-06	28.1			

Yehia et al. 2016 **Graph (experimental points)**			***Recommended***		
**C (M)**	**Molar fraction**	**σ (mN/m)**	***C (M)***	***Molar fraction***	***σ (mN/m)***
**9.71E-07**	1.75E-08	55.0	*1.85E-08*	*3.33E-10*	*71.7*
**1.97E-05**	3.55E-07	53.1	*3.71E-08*	*6.67E-10*	*71.5*
**5.00E-05**	9.00E-07	51.4	*7.43E-08*	*1.34E-09*	*71.1*
**8.00E-05**	1.44E-06	48.6	*1.49E-07*	*2.68E-09*	*70.4*
**9.80E-05**	1.76E-06	46.5	*2.98E-07*	*5.37E-09*	*69.1*
**2.00E-04**	3.60E-06	42.1	*5.98E-07*	*1.08E-08*	*67.0*
**4.97E-04**	8.95E-06	32.7	*1.20E-06*	*2.16E-08*	*63.7*
**8.10E-04**	1.46E-05	32.3	*2.40E-06*	*4.32E-08*	*59.0*
**1.00E-03**	1.80E-05	32.3	*4.81E-06*	*8.66E-08*	*53.3*
**1.98E-03**	3.57E-05	32.1	*9.64E-06*	*1.73E-07*	*47.3*
**4.94E-03**	8.91E-05	32.5	*1.93E-05*	*3.48E-07*	*42.2*
**8.06E-03**	1.45E-04	32.1	*3.87E-05*	*6.96E-07*	*38.4*
			7.75E-05	*1.40E-06*	*35.8*
			1.55E-04	*2.80E-06*	*34.3*
			3.11E-04	*5.60E-06*	*33.4*
			6.24E-04	*1.12E-05*	*32.8*
			1.25E-03	*2.25E-05*	*32.6*
			2.50E-03	*4.51E-05*	*32.4*
			5.01E-03	*9.03E-05*	*32.3*
			1.00E-02	*1.81E-04*	*32.3*

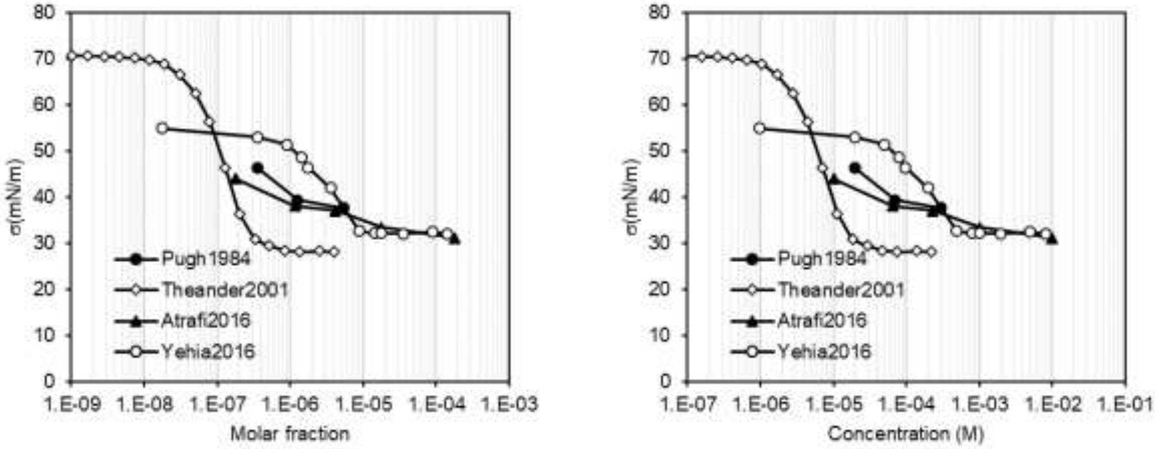

Fig. S38 (a): Experimental surface tension data for oleic acid / water mixtures.

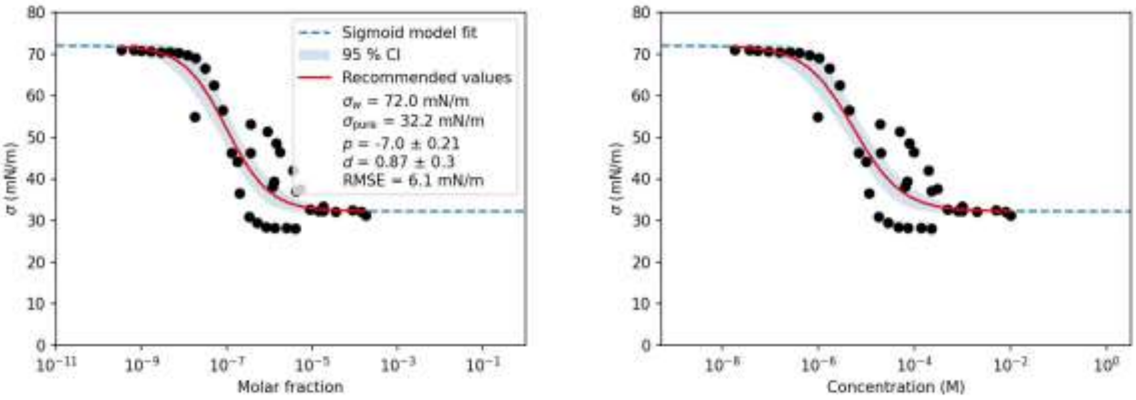

Fig. S38 (b): Surface tension fit with the Sigmoid model (*Kleinheins et al. 2023*) for oleic acid / water mixtures. Solid red line: model fit inside the concentration range covered by experimental data, reported as recommended values. Blue shading: fit parameters with 95 % confidence interval (CI). RMSE: root mean squared error. Markers: data used for fitting.

Comment:

Differences between the different references may come from the purity of the chemicals or the pH of the solution, the surface tension of the oleic acid being sensitive to the pH (*e.g. Pugh et al. 1984, Theander et al. 2001, Atrafi et al. 2016, Yehia et al. 2016*).

Pugh et al. 1984 : surface tension measured vs concentration of sodium oleate, pH = 5.9 - 6.4
Theander et al. 2001 : surface tension measured vs concentration of sodium oleate, pH = 7
Atrafi et al. 2016 : surface tension measured vs concentration of sodium oleate, pH = 6
Yehia et al. 2016 : surface tension measured vs concentration of oleic acid, pH = 6

AC39: ricinoleic acid ((9Z,12R)-12-hydroxyoctadec-9-enoic acid)

Knothe et al. 1993	**Graph (experimental points)**		
**C (M)**	**Molar fraction**	**logC (M)**	**σ (mN/m)**
4.67E-07	8.40E-09	**-6.33**	73.7
1.16E-06	2.09E-08	**-5.93**	73.3
2.96E-06	5.33E-08	**-5.53**	69.7
7.39E-06	1.33E-07	**-5.13**	63.8
1.88E-05	3.39E-07	**-4.73**	54.4
4.50E-05	8.09E-07	**-4.35**	48.3
1.15E-04	2.06E-06	**-3.94**	42.5
2.74E-04	4.93E-06	**-3.56**	37.6
6.97E-04	1.25E-05	**-3.16**	33.4
1.74E-03	3.13E-05	**-2.76**	33.4
2.89E-03	5.21E-05	**-2.54**	33.0
4.72E-03	8.50E-05	**-2.33**	33.2

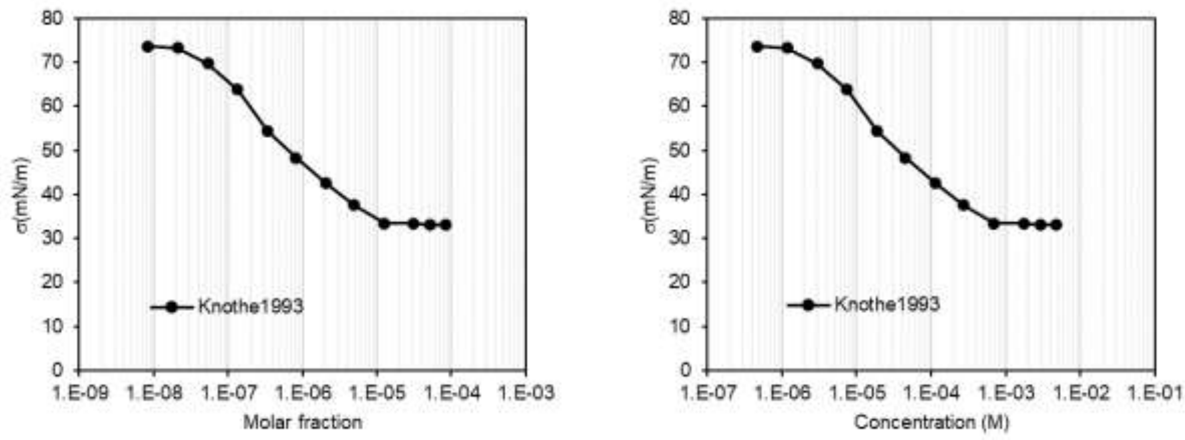

Fig. S39: Experimental surface tension data for ricinoleic acid / water mixtures.

Comment

Aqueous solutions of ricinoleic acid in 1N NaOH

AC40: arachidonic acid ((5Z,8Z,11Z,14Z)-icosa-5,8,11,14-tetraenoic acid)

Yokoyama et al. 1993 **Graph (experimental points)**			
**C (M)**	**Molar fraction**	**logC (mol/m³)**	**σ (mN/m)**
9.82E-07	1.77E-08	**-3.01**	67.8
3.96E-06	7.13E-08	**-2.40**	60.8
9.73E-06	1.75E-07	**-2.01**	52.5
2.48E-05	4.46E-07	**-1.61**	42.6
4.89E-05	8.80E-07	**-1.31**	34.3
8.48E-05	1.53E-06	**-1.07**	32.1
1.97E-04	3.55E-06	**-0.71**	31.8
4.03E-04	7.26E-06	**-0.39**	32.1

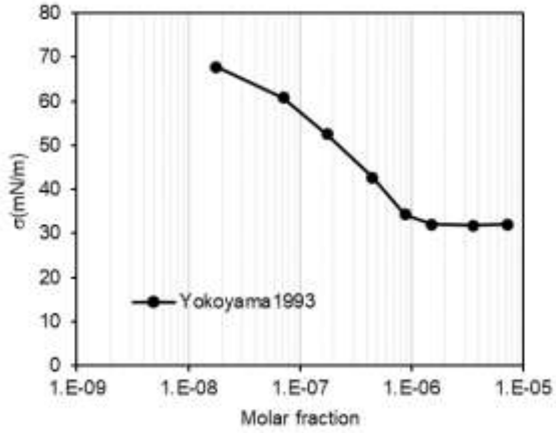

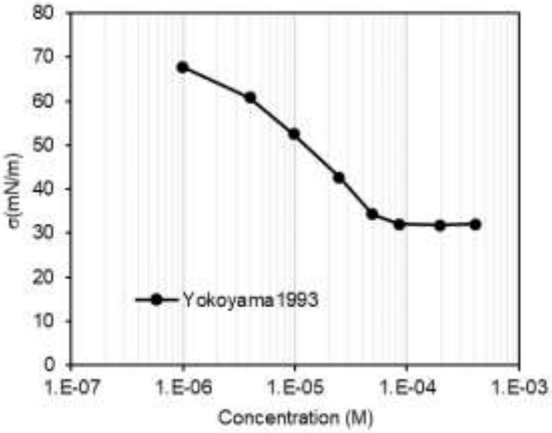

Fig. S40: Experimental surface tension data for arachidonic acid / water mixtures.

Comment:

Yokoyama et al. 1993 : data reported at 30°C.

AC41: 7,10-dihydroxy-8(E)-octadecenoic acid

Knothe et al. 1993	**Graph (experimental points)**		
**C (M)**	**Molar fraction**	**logC (M)**	**σ (mN/m)**
1.41E-06	2.54E-08	**-5.85**	73.7
2.45E-06	4.41E-08	**-5.61**	72.2
3.03E-06	5.45E-08	**-5.52**	71.4
7.71E-06	1.39E-07	**-5.11**	65.9
1.43E-05	2.57E-07	**-4.85**	64.4
3.79E-05	6.83E-07	**-4.42**	58.7
9.26E-05	1.67E-06	**-4.03**	54.4
2.41E-04	4.34E-06	**-3.62**	48.2
5.76E-04	1.04E-05	**-3.24**	41.7
1.00E-03	1.80E-05	**-3.00**	40.6
1.60E-03	2.87E-05	**-2.80**	40.2
2.66E-03	4.79E-05	**-2.58**	39.5

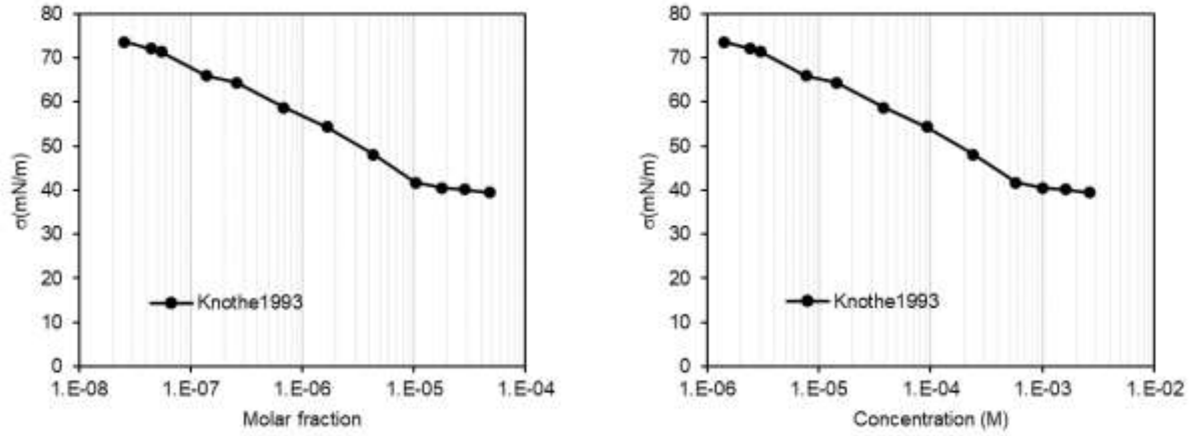

Fig. S41: Comparison of the experimental surface tension data for 7,10-dihydroxy-8(E)-octadecenoic acid / water mixtures.

Comment: N/A

S3.2 Aldehydes. ketones. Alcohols

AK1: formaldehyde (methanal)

Li et al. 2011 **Graph (experimental points)**			
**C (M)**	**Molar fraction**	**Molality (mol/kgw)**	**σ (mN/m)**
0.00E+00	0.00E+00	**0.00E+00**	72.0
2.74E-02	4.94E-04	**2.75E-02**	72.5
6.48E-02	1.17E-03	**6.50E-02**	71.5
9.95E-02	1.79E-03	**1.00E-01**	71.5
1.49E-01	2.69E-03	**1.50E-01**	70.5
1.99E-01	3.58E-03	**2.00E-01**	73.0

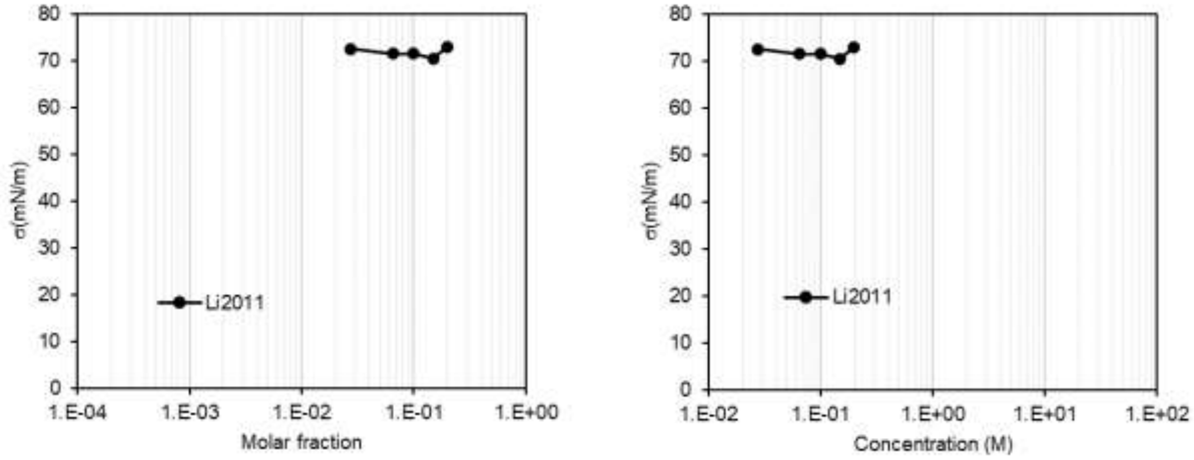

Fig. S42: Experimental surface tension data for formaldehyde / water mixtures.

Comment

Li et al. 2011: the molar fraction was calculated from the density at -20°C (available date).

AK2: methanol

Vázquez et al. 1995 **Table**			Glinski et al. 1998 **Graph (experimental points)**			Belda Maximino 2009 **Table**		
**C (M)**	**Molar fraction**	**σ (mN/m)**	**C (M)**	**Molar fraction**	**σ (mN/m)**	**C (M)**	**Molar fraction**	**σ (mN/m)**
0.00E+00	**0.00E+00**	72.01	0.00E+00	**0.00E+00**	72.2	0.00E+00	**0.00E+00**	72.08
1.55E+00	**2.90E-02**	62.77	1.08E+00	**2.00E-02**	65.0	1.46E+00	**2.72E-02**	63.52
3.05E+00	**5.90E-02**	56.18	2.12E+00	**4.00E-02**	60.0	3.04E+00	**5.88E-02**	56.21
4.49E+00	**9.00E-02**	51.17	2.61E+00	**5.00E-02**	57.0	4.68E+00	**9.42E-02**	50.52
5.92E+00	**1.23E-01**	47.21	4.04E+00	**8.00E-02**	54.0	6.75E+00	**1.43E-01**	44.98
7.33E+00	**1.58E-01**	43.78	4.94E+00	**1.00E-01**	52	8.68E+00	**1.94E-01**	41.45
8.68E+00	**1.94E-01**	41.09	5.80E+00	**1.20E-01**	49.0	1.13E+01	**2.73E-01**	37.12
1.13E+01	**2.73E-01**	36.51	6.21E+00	**1.30E-01**	47.0	1.38E+01	**3.60E-01**	33.35
1.38E+01	**3.60E-01**	32.86	8.89E+00	**2.00E-01**	43.0	1.62E+01	**4.58E-01**	29.96
1.62E+01	**4.58E-01**	29.83	1.06E+01	**2.50E-01**	39.0	1.84E+01	**5.68E-01**	27.48
1.85E+01	**5.68E-01**	27.48	1.21E+01	**3.00E-01**	37.0	2.06E+01	**6.92E-01**	25.64
2.06E+01	**6.92E-01**	25.54	1.48E+01	**4.00E-01**	34.0	2.27E+01	**8.35E-01**	24.06
2.27E+01	**8.35E-01**	23.93	1.71E+01	**5.00E-01**	32.0	2.47E+01	**1.00E+00**	22.14
2.47E+01	**1.00E+00**	22.51	2.07E+01	**7.00E-01**	30.0			
			2.35E+01	**9.00E-01**	26.0			
			2.47E+01	**1.00E+00**	24.0			

Semenov et al. 2014 **Graph (experimental points)**			Basarova et al. 2016 **Table**			***Recommended***		
**C (M)**	**Molar fraction**	**σ (mN/m)**	**C (M)**	**Molar fraction**	**σ (mN/m)**	***C (M)***	***Molar fraction***	***σ (mN/m)***
0.00E+00	**0.00E+00**	72.5	0.00E+00	**0.00E+00**	72.2	*6.03E-01*	*1.10E-02*	*67.8*
1.86E+00	**3.50E-02**	60.5	6.03E-01	**1.10E-02**	68.1	*7.62E-01*	*1.39E-02*	*66.9*
2.12E+00	**4.00E-02**	59.5	1.66E+00	**3.10E-02**	62.2	*9.61E-01*	*1.77E-02*	*65.7*
3.10E+00	**6.00E-02**	55.0	3.15E+00	**6.10E-02**	55.9	*1.21E+00*	*2.24E-02*	*64.4*
6.01E+00	**1.25E-01**	48.5	4.58E+00	**9.20E-02**	51.2	*1.53E+00*	*2.84E-02*	*62.8*
6.42E+00	**1.35E-01**	45.0	6.25E+00	**1.31E-01**	46.7	*1.92E+00*	*3.60E-02*	*61.0*
8.89E+00	**2.00E-01**	41.0	9.28E+00	**2.11E-01**	40.5	*2.40E+00*	*4.57E-02*	*58.9*
9.24E+00	**2.10E-01**	40.0	1.33E+01	**3.41E-01**	35.9	*3.00E+00*	*5.79E-02*	*56.5*
1.27E+01	**3.20E-01**	32.5	1.84E+01	**5.65E-01**	30.3	*3.74E+00*	*7.35E-02*	*53.9*
1.28E+01	**3.25E-01**	32.0	2.06E+01	**6.92E-01**	27.9	*4.63E+00*	*9.31E-02*	*51.0*
1.66E+01	**4.75E-01**	28.5	2.17E+01	**7.62E-01**	26.3	*5.72E+00*	*1.18E-01*	*48.0*
1.71E+01	**5.00E-01**	27.5	2.27E+01	**8.32E-01**	25.2	*7.01E+00*	*1.50E-01*	*44.8*
1.98E+01	**6.40E-01**	25.0	2.37E+01	**9.15E-01**	24.1	*8.52E+00*	*1.90E-01*	*41.7*
2.03E+01	**6.70E-01**	24.5	2.42E+01	**9.60E-01**	23.5	*1.03E+01*	*2.41E-01*	*38.5*
2.24E+01	**8.10E-01**	25.0	2.47E+01	**1.00E+00**	22.7	*1.23E+01*	*3.05E-01*	*35.4*
2.25E+01	**8.20E-01**	24.5				*1.45E+01*	*3.87E-01*	*32.6*
2.40E+01	**9.40E-01**	25.0				*1.69E+01*	*4.91E-01*	*29.9*
2.47E+01	**1.00E+00**	25.0				*1.94E+01*	*6.22E-01*	*27.5*
						2.21E+01	*7.89E-01*	*25.3*
						2.47E+01	*1.00E+00*	*23.5*

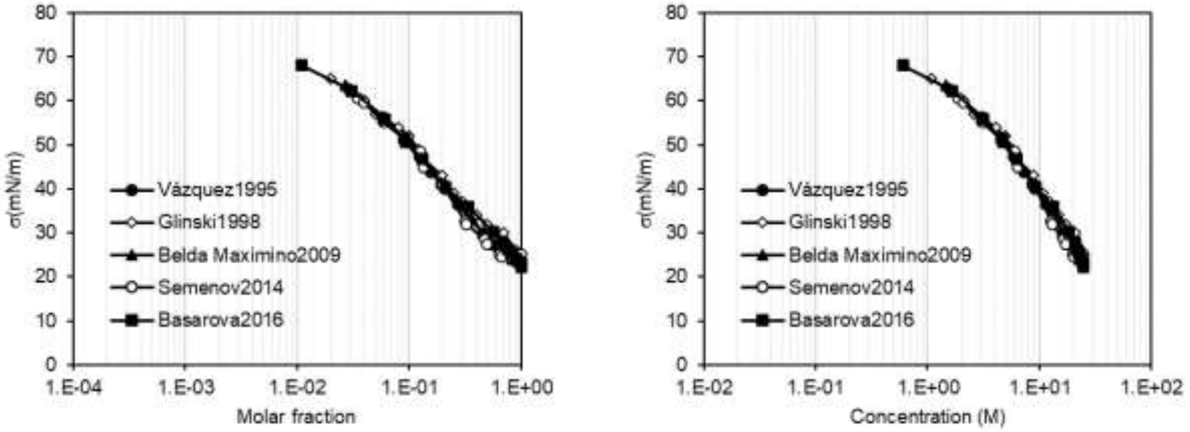

Fig. S43 (a): Comparison of the experimental surface tension data for methanol / water mixtures.

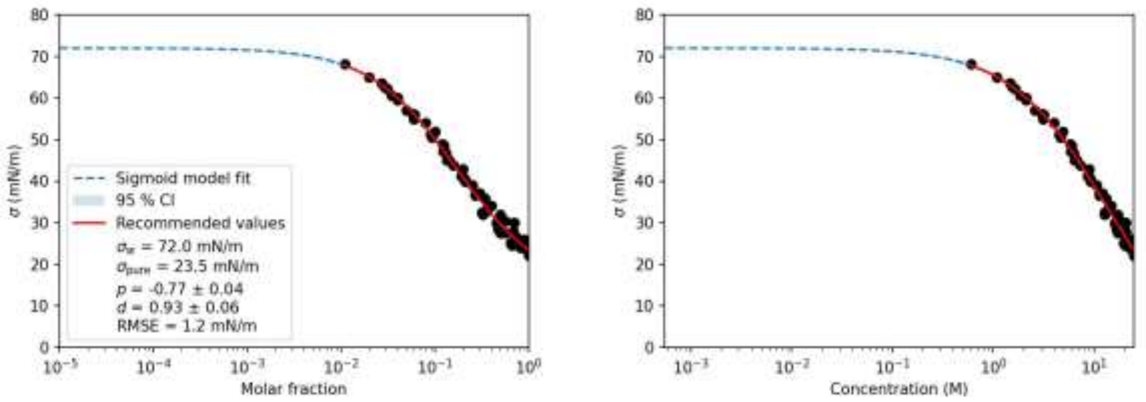

Fig. S43 (b): Surface tension fit with the Sigmoid model (*Kleinheins et al. 2023*) for methanol / water mixtures. Solid red line: model fit inside the concentration range covered by experimental data, reported as recommended values. Blue shading: fit parameters with 95 % confidence interval (CI). RMSE: root mean squared error. Markers: data used for fitting.

Comment:

Glinski et al. 1998 : data reported at 19.85°C.

AK3: acetaldehyde (ethanal)

Li et al. 2011 **Graph (experimental points)**			
**C (M)**	**Molar fraction**	**Molality (mol/kgw)**	**σ (mN/m)**
0.00E+00	0.00E+00	**0.00E+00**	72.0
3.98E-02	7.18E-04	**4.00E-02**	68.5
8.94E-02	1.61E-03	**9.00E-02**	68.0
1.78E-01	3.22E-03	**1.80E-01**	67.0
3.43E-01	6.25E-03	**3.50E-01**	66.5
8.37E-01	1.56E-02	**8.80E-01**	65.5

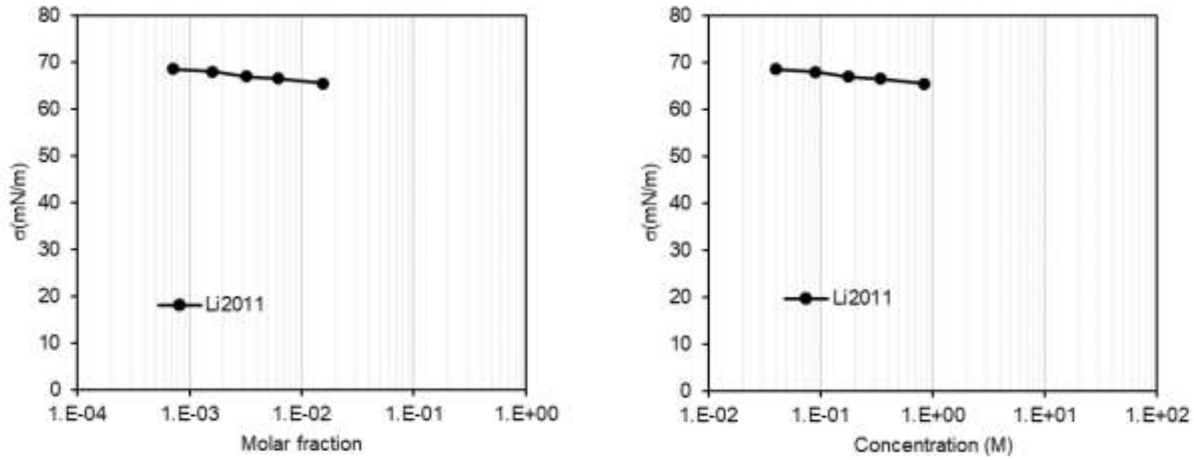

Fig. S44: Experimental surface tension data for acetaldehyde / water mixtures.

Comment: N/A

AK4: ethanol

Ernst et al. 1936 **Table**			
**C (M)**	**Molar fraction**	**Mass fraction**	**σ (mN/m)**
0.00E+00	0.00E+00	**0.00E+00**	72.0
2.11E+00	4.16E-02	**1.00E-01**	46.6
4.12E+00	8.90E-02	**2.00E-01**	37.7
6.02E+00	1.43E-01	**3.00E-01**	32.3
7.83E+00	2.07E-01	**4.00E-01**	29.6
9.55E+00	2.81E-01	**5.00E-01**	28.3
1.12E+01	3.70E-01	**6.00E-01**	26.9
1.28E+01	4.77E-01	**7.00E-01**	26.1
1.42E+01	6.10E-01	**8.00E-01**	25.2
1.57E+01	7.79E-01	**9.00E-01**	24.4
1.70E+01	1.00E+00	**1.00E+00**	22.0

Vázquez et al. 1995 **Table**		
**C (M)**	**Molar fraction**	**σ (mN/m)**
0.00E+00	**0.00E+00**	72.01
1.06E+00	**2.00E-02**	55.73
2.13E+00	**4.20E-02**	47.53
3.15E+00	**6.50E-02**	42.08
4.12E+00	**8.90E-02**	37.97
5.08E+00	**1.15E-01**	35.51
6.05E+00	**1.44E-01**	32.98
7.85E+00	**2.07E-01**	30.16
9.58E+00	**2.81E-01**	27.96
1.12E+01	**3.70E-01**	26.23
1.28E+01	**4.77E-01**	25.01
1.43E+01	**6.10E-01**	23.82
1.58E+01	**7.79E-01**	22.72
1.71E+01	**1.00E+00**	21.82

Glinski et al. 1998 **Graph (experimental points)**		
**C (M)**	**Molar fraction**	**σ (mN/m)**
0.00E+00	**0.00E+00**	72.2
2.04E+00	**4.00E-02**	48.0
2.94E+00	**6.00E-02**	44.0
3.77E+00	**8.00E-02**	40.0
4.54E+00	**1.00E-01**	37.0
5.92E+00	**1.40E-01**	32.0
7.67E+00	**2.00E-01**	30.0
8.90E+00	**2.50E-01**	29.5
9.96E+00	**3.00E-01**	29.0
1.17E+01	**4.00E-01**	28.0
1.31E+01	**5.00E-01**	27.0
1.42E+01	**6.00E-01**	26.0
1.51E+01	**7.00E-01**	25.0
1.59E+01	**8.00E-01**	24.0
1.66E+01	**9.00E-01**	23.0
1.71E+01	**1.00E+00**	22.0

Belda Maximino 2009 **Table**		
**C (M)**	**Molar fraction**	**σ (mN/m)**
0.00E+00	**0.00E+00**	72.08
7.70E-01	**1.43E-02**	60.68
1.91E+00	**3.72E-02**	50.33
4.37E+00	**9.55E-02**	38.22
7.69E+00	**2.01E-01**	31.28
9.94E+00	**2.99E-01**	28.28
1.17E+01	**4.00E-01**	26.48
1.31E+01	**5.01E-01**	25.25
1.42E+01	**5.99E-01**	24.36
1.51E+01	**7.00E-01**	23.63
1.58E+01	**7.91E-01**	23.04
1.66E+01	**9.01E-01**	22.52
1.71E+01	**1.00E+00**	21.72

Khattab et al. 2011 **Table**		
**C (M)**	**Molar fraction**	**σ (mN/m)**
0.00E+00	**0.00E+00**	72.88
1.74E+00	**3.30E-02**	53.43
3.60E+00	**7.20E-02**	43.71
5.50E+00	**1.17E-01**	37.16
7.50E+00	**1.71E-01**	33.88
9.58E+00	**2.36E-01**	31.36
1.18E+01	**3.16E-01**	28.95
1.41E+01	**4.19E-01**	27.45
1.65E+01	**5.52E-01**	26.00
1.90E+01	**7.35E-01**	24.49
2.17E+01	**1.00E+00**	22.85

Basarova et al. 2015 **Table**		
**C (M)**	**Molar fraction**	**σ (mN/m)**
0.00E+00	**0.00E+00**	72.2
4.37E-01	**8.00E-03**	63.1
1.06E+00	**2.00E-02**	56.7
2.13E+00	**4.20E-02**	48.9
3.15E+00	**6.50E-02**	43.4
4.12E+00	**8.90E-02**	39.3
6.08E+00	**1.45E-01**	33.3
9.53E+00	**2.79E-01**	29.1
1.28E+01	**4.74E-01**	26.7
1.42E+01	**5.98E-01**	25.7
1.50E+01	**6.80E-01**	25.1
1.58E+01	**7.82E-01**	24.3
1.64E+01	**8.73E-01**	23.5
1.68E+01	**9.35E-01**	23.0
1.71E+01	**1.00E+00**	22.6

Recommended		
C (M)	***Molar fraction***	***σ (mN/m)***
4.37E-01	*8.00E-03*	*63.5*
5.60E-01	*1.03E-02*	*61.8*
7.17E-01	*1.33E-02*	*59.8*
9.17E-01	*1.71E-02*	*57.6*
1.17E+00	*2.21E-02*	*55.1*
1.49E+00	*2.85E-02*	*52.4*
1.89E+00	*3.68E-02*	*49.6*
2.38E+00	*4.74E-02*	*46.6*
2.98E+00	*6.11E-02*	*43.6*

3.72E+00	*7.88E-02*	*40.7*
4.60E+00	*1.02E-01*	*37.8*
5.62E+00	*1.31E-01*	*35.1*
6.80E+00	*1.69E-01*	*32.7*
8.12E+00	*2.18E-01*	*30.5*
9.57E+00	*2.81E-01*	*28.5*
1.11E+01	*3.62E-01*	*26.8*
1.27E+01	*4.67E-01*	*25.3*
1.42E+01	*6.02E-01*	*24.1*
1.57E+01	*7.76E-01*	*23.1*
1.71E+01	*1.00E+00*	*22.2*

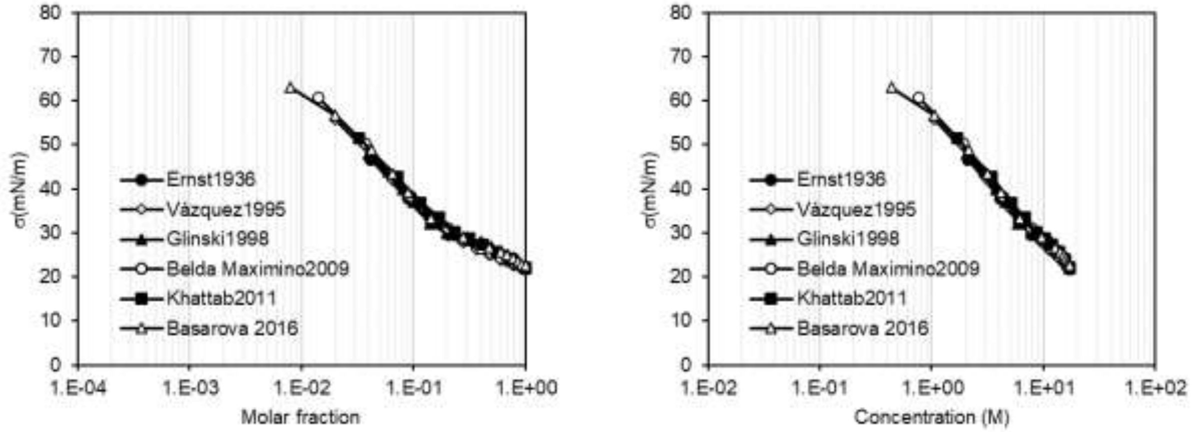

Fig. S45 (a): Comparison of the experimental surface tension data for ethanol / water mixtures.

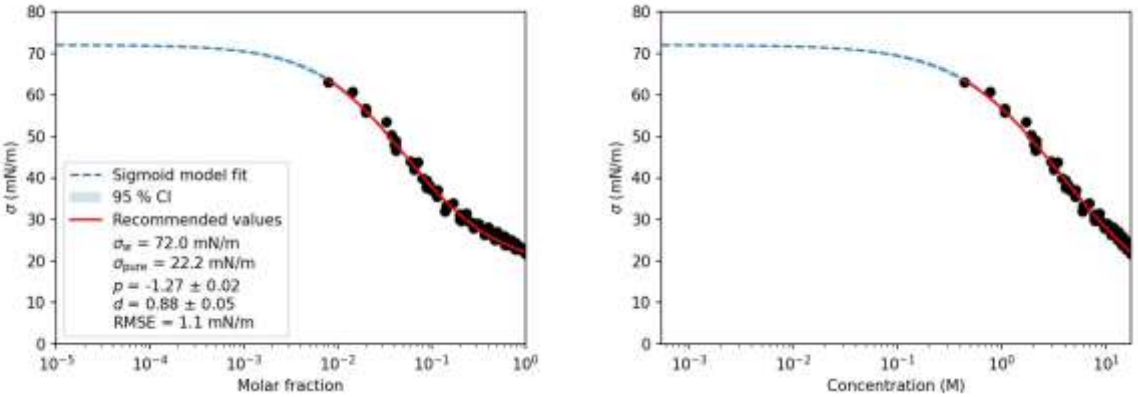

Fig. S45 (b): Surface tension fit with the Sigmoid model (*Kleinheins et al. 2023*) for ethanol / water mixtures. Solid red line: model fit inside the concentration range covered by experimental data, reported as recommended values. Blue shading: fit parameters with 95 % confidence interval (CI). RMSE: root mean squared error. Markers: data used for fitting.

Comment:

Glinski et al. 1998 and *Khattab et al. 2011* : data reported at 19.85°C.

AK5: acetone (propan-2-one)

Howard et al. 1957 **Table**			Toryanik et al. 1977 **Table**			Enders et al. 2007 **Table**		
**C (M)**	**Molar fraction**	**σ (mN/m)**	**C (M)**	**Molar fraction**	**σ (mN/m)**	**C (M)**	**Molar fraction**	**σ (mN/m)**
0.00E+00	**0.00E+00**	71.94	0.00E+00	**0.00E+00**	72.0	0.00E+00	**0.00E+00**	71.98
2.73E+00	**5.80E-02**	42.64	6.89E-01	**1.29E-02**	54.8	5.34E-01	**9.90E-03**	61.04
5.57E+00	**1.46E-01**	33.56	1.96E+00	**3.96E-02**	44.0	2.40E+00	**5.00E-02**	46.14
7.80E+00	**2.50E-01**	29.70	5.68E+00	**1.50E-01**	34.4	4.23E+00	**9.99E-02**	39.07
1.06E+01	**4.70E-01**	26.57	7.58E+00	**2.37E-01**	30.1	5.68E+00	**1.50E-01**	34.41
1.21E+01	**6.77E-01**	24.96	1.09E+01	**5.00E-01**	28.6	6.85E+00	**2.00E-01**	32.21
1.31E+01	**8.92E-01**	23.42	1.25E+01	**7.57E-01**	24.3	8.62E+00	**3.00E-01**	29.35
1.35E+01	**9.86E-01**	22.69	1.35E+01	**1.00E+00**	23.1	9.90E+00	**4.00E-01**	27.98
						1.09E+01	**5.00E-01**	27.04
						1.16E+01	**6.00E-01**	26.03
						1.22E+01	**7.00E-01**	25.44
						1.27E+01	**8.00E-01**	24.51
						1.32E+01	**9.00E-01**	23.80
						1.35E+01	**1.00E+00**	23.02

Semenov et al. 2014 **Graph (experimental points)**			Wu et al. 2019 **Graph (experimental points)**				***Recommended***		
**C (M)**	**Molar fraction**	**σ (mN/m)**	**C (M)**	**Molar fraction**	**Molar ratio $n_{acetone}/n_{H2O}$**	**σ (mN/m)**	***C (M)***	***Molar fraction***	***σ (mN/m)***
0.00E+00	**0.00E+00**	73.0	0.000E+00	0.00E+00	**0.00E+00**	72.5	*5.34E-01*	*9.90E-03*	*59.0*
5.39E-01	**1.00E-02**	59.0	2.304E+00	4.76E-02	**5.00E-02**	65.0	*6.75E-01*	*1.26E-02*	*57.1*
1.05E+00	**2.00E-02**	53.5	3.937E+00	9.09E-02	**1.00E-01**	60.0	*8.51E-01*	*1.61E-02*	*55.1*
2.81E+00	**6.00E-02**	43.5	5.558E+00	1.45E-01	**1.70E-01**	56.5	*1.07E+00*	*2.05E-02*	*52.9*
4.24E+00	**1.00E-01**	37.0	6.096E+00	1.67E-01	**2.00E-01**	55.0	*1.34E+00*	*2.62E-02*	*50.7*
6.85E+00	**2.00E-01**	32.0	6.848E+00	2.00E-01	**2.50E-01**	54.0	*1.68E+00*	*3.34E-02*	*48.3*
9.18E+00	**3.40E-01**	28.0	7.807E+00	2.50E-01	**3.33E-01**	50.0	*2.09E+00*	*4.25E-02*	*45.9*
9.79E+00	**3.90E-01**	27.5	9.088E+00	3.33E-01	**5.00E-01**	47.0	*2.58E+00*	*5.42E-02*	*43.5*
1.05E+01	**4.60E-01**	25.0	1.020E+01	4.29E-01	**7.50E-01**	42.5	*3.16E+00*	*6.91E-02*	*41.2*
1.13E+01	**5.60E-01**	25.0	1.087E+01	5.00E-01	**1.00E+00**	40.0	*3.84E+00*	*8.81E-02*	*38.9*
1.23E+01	**7.20E-01**	25.0	1.162E+01	6.00E-01	**1.50E+00**	36.0	*4.63E+00*	*1.12E-01*	*36.7*
1.24E+01	**7.30E-01**	25.0	1.204E+01	6.67E-01	**2.00E+00**	34.0	*5.51E+00*	*1.43E-01*	*34.6*
1.30E+01	**8.70E-01**	24.5	1.231E+01	7.14E-01	**2.50E+00**	32.0	*6.47E+00*	*1.83E-01*	*32.6*
1.35E+01	**1.00E+00**	24.5	1.250E+01	7.50E-01	**3.00E+00**	31.0	*7.50E+00*	*2.33E-01*	*30.9*
			1.273E+01	8.00E-01	**4.00E+00**	30.0	*8.57E+00*	*2.97E-01*	*29.2*
			1.288E+01	8.33E-01	**5.00E+00**	28.5	*9.66E+00*	*3.78E-01*	*27.8*
			1.298E+01	8.57E-01	**6.00E+00**	28.0	*1.07E+01*	*4.83E-01*	*26.5*
			1.305E+01	8.75E-01	**7.00E+00**	27.5	*1.17E+01*	*6.15E-01*	*25.4*
			1.311E+01	8.89E-01	**8.00E+00**	27.5	*1.27E+01*	*7.84E-01*	*24.4*
			1.315E+01	9.00E-01	**9.00E+00**	27.5	*1.35E+01*	*1.00E+00*	*23.5*
			1.319E+01	9.09E-01	**1.00E+01**	27.5			
			1.322E+01	9.17E-01	**1.10E+01**	25.5			
			1.324E+01	9.23E-01	**1.20E+01**	25.5			
			1.326E+01	9.29E-01	**1.30E+01**	25.5			
			1.329E+01	9.38E-01	**1.50E+01**	25.5			
			1.331E+01	9.41E-01	**1.60E+01**	25.5			
			1.333E+01	9.47E-01	**1.80E+01**	25.5			
			1.334E+01	9.50E-01	**1.90E+01**	25.5			
			1.335E+01	9.52E-01	**2.00E+01**	25.0			

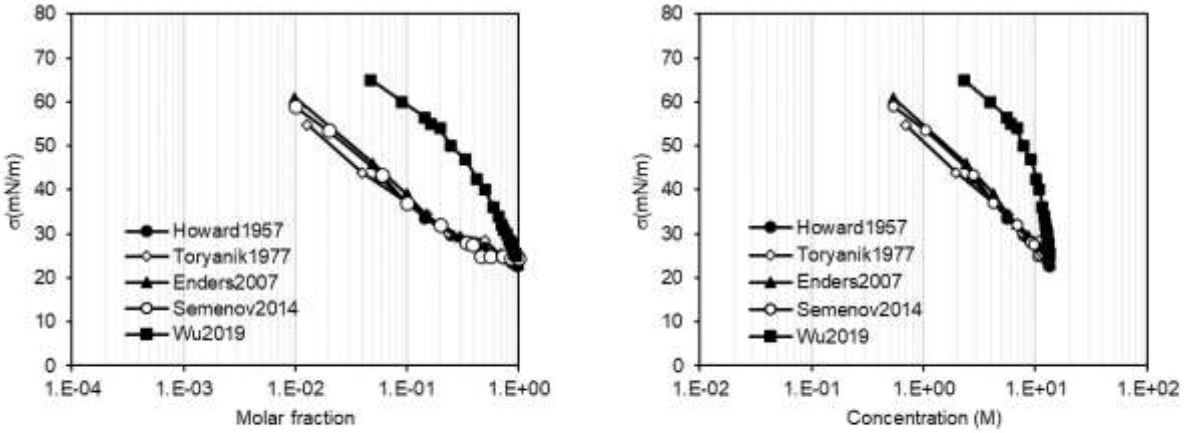

Fig. S46 (a): Comparison of the experimental surface tension data for acetone / water mixtures.

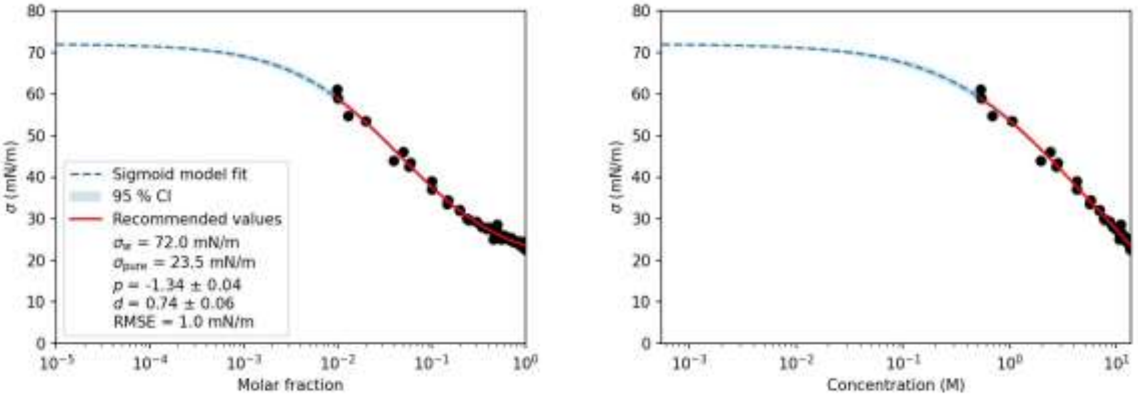

Fig. S46 (b): Surface tension fit with the Sigmoid model (*Kleinheins et al. 2023*) for acetone / water mixtures. Solid red line: model fit inside the concentration range covered by experimental data, reported as recommended values. Blue shading: fit parameters with 95 % confidence interval (CI). RMSE: root mean squared error. Markers: data used for fitting.

Comment

The data from *Wu et al. 2019* were not included for the fitting, because they differ from the other surface tension isotherms.

AK6: propan-1-ol

Vázquez et al. 1995 **Table**			Messow et al. 1998 **Graph (experimental points)**			Donaldson et al. 1999 **Graph (experimental points)**		
**C (M)**	**Molar fraction**	**σ (mN/m)**	**C (M)**	**Molar fraction**	**σ (mN/m)**	**C (M)**	**Molar fraction**	**σ (mN/m)**
0.00E+00	**0.00E+00**	72.01	0.00E+00	**0.00E+00**	72.0	**0.00E+00**	0.00E+00	72.0
8.46E-01	**1.60E-02**	41.83	5.38E-01	**1.00E-02**	50.0	**2.64E-01**	4.82E-03	62.0
1.61E+00	**3.20E-02**	34.32	1.04E+00	**2.00E-02**	39.0	**2.64E-01**	4.82E-03	61.3
2.40E+00	**5.00E-02**	30.36	2.41E+00	**5.03E-02**	31.0	**3.88E-01**	7.14E-03	58.6
3.18E+00	**7.00E-02**	27.84	3.18E+00	**7.00E-02**	28.0	**3.88E-01**	7.14E-03	57.2
3.92E+00	**9.10E-02**	26.64	4.63E+00	**1.13E-01**	26.0	**3.88E-01**	7.14E-03	56.0
4.65E+00	**1.14E-01**	25.98	6.70E+00	**1.96E-01**	26.0	**5.10E-01**	9.46E-03	55.2
6.06E+00	**1.67E-01**	25.26	8.68E+00	**3.10E-01**	26.0	**5.10E-01**	9.46E-03	52.0
7.40E+00	**2.31E-01**	24.8	9.91E+00	**4.12E-01**	26.0	**5.10E-01**	9.46E-03	50.0
8.68E+00	**3.10E-01**	24.49	1.08E+01	**5.08E-01**	26.0	**6.35E-01**	1.19E-02	50.0
9.92E+00	**4.12E-01**	24.08	1.15E+01	**6.02E-01**	26.0	**6.35E-01**	1.19E-02	49.2
1.11E+01	**5.45E-01**	23.86	1.22E+01	**7.19E-01**	26.0	**6.35E-01**	1.19E-02	48.6
1.22E+01	**7.30E-01**	23.59	1.26E+01	**7.99E-01**	26.0	**7.55E-01**	1.42E-02	48.6
1.33E+01	**1.00E+00**	23.28	1.30E+01	**9.07E-01**	26.0	**7.55E-01**	1.42E-02	47.3
			1.33E+01	**1.00E+00**	26.0	**8.72E-01**	1.65E-02	47.3
						**8.72E-01**	1.65E-02	45.4
						**8.72E-01**	1.65E-02	44.9
						**9.85E-01**	1.88E-02	44.6
						**9.85E-01**	1.88E-02	43.3
						**1.10E+00**	2.11E-02	42.5
						**1.10E+00**	2.11E-02	41.3
						**1.21E+00**	2.34E-02	40.8
						**1.21E+00**	2.34E-02	39.1
						**1.43E+00**	2.80E-02	38.5
						**1.43E+00**	2.80E-02	35.8
						**1.64E+00**	3.26E-02	35.4
						**1.64E+00**	3.26E-02	34.8
						**1.84E+00**	3.70E-02	34.4
						**1.84E+00**	3.70E-02	33.0
						**1.84E+00**	3.70E-02	32.0
						**2.03E+00**	4.14E-02	31.8
						**2.03E+00**	4.14E-02	31.2
						**2.22E+00**	4.58E-02	30.6
						**2.22E+00**	4.58E-02	30.0
						**2.58E+00**	5.45E-02	29.0
						**2.58E+00**	5.45E-02	28.0
						**2.91E+00**	6.30E-02	27.8
						**2.91E+00**	6.30E-02	27.1

Demou et al. 2002 **Graph (experimental points)**			Romero et al. 2006 **Table**			Belda Maximino 2009 **Table**		
**C (M)**	**Molar fraction**	**σ (mN/m)**	**C (M)**	**Molar fraction**	**σ (mN/m)**	**C (M)**	**Molar fraction**	**σ (mN/m)**
**0.00E+00**	0.00E+00	70.0	0.00E+00	**0.00E+00**	71.8	0.00E+00	**0.00E+00**	72.08
**1.43E-01**	2.60E-03	62.5	2.77E-02	**5.00E-04**	67.6	2.30E+00	**4.77E-02**	64.26
**2.65E-01**	4.84E-03	55.5	1.43E-01	**2.60E-03**	61.8	4.56E+00	**1.11E-01**	54.19
**5.10E-01**	9.46E-03	45.7	2.57E-01	**4.70E-03**	56.5	6.64E+00	**1.92E-01**	42.31
**7.55E-01**	1.42E-02	34.5	4.86E-01	**9.00E-03**	48.3	8.08E+00	**2.70E-01**	29.28
**1.00E+00**	1.91E-02	31.6	1.04E+00	**2.00E-02**	39.7	9.16E+00	**3.46E-01**	25.64
**1.50E+00**	2.95E-02	27.3	1.97E+00	**4.00E-02**	32.1	9.79E+00	**4.00E-01**	25.52
**2.00E+00**	4.06E-02	26.8	2.60E+00	**5.49E-02**	28.7	1.07E+01	**5.00E-01**	25.41
**2.50E+00**	5.25E-02	26.1	2.80E+00	**6.00E-02**	28.0	1.14E+01	**5.88E-01**	25.23
**3.00E+00**	6.52E-02	25.4	3.14E+00	**6.89E-02**	27.3	1.20E+01	**6.90E-01**	24.20
**3.50E+00**	7.88E-02	24.5	4.22E+00	**1.00E-01**	24.7	1.25E+01	**7.87E-01**	23.85
**4.00E+00**	9.33E-02	24.2	5.64E+00	**1.50E-01**	24.9	1.29E+01	**8.86E-01**	23.57
			6.79E+00	**2.00E-01**	24.6	1.33E+01	**1.00E+00**	23.32
			7.74E+00	**2.50E-01**	24.4			
			8.53E+00	**3.00E-01**	23.2			
			1.03E+01	**4.50E-01**	24.5			
			1.14E+01	**5.86E-01**	23.6			
			1.15E+01	**6.00E-01**	23.4			
			1.23E+01	**7.50E-01**	23.6			
			1.28E+01	**8.50E-01**	23.4			
			1.30E+01	**9.00E-01**	22.4			
			1.33E+01	**1.00E+00**	23.1			

Basarova et al. 2016 **Table**			***Recommended***		
**C (M)**	**Molar fraction**	**σ (mN/m)**	***C (M)***	***Molar fraction***	***σ (mN/m)***
0.00E+00	**0.00E+00**	72.2	*2.77E-02*	*5.00E-04*	*71.2*
3.27E-01	**6.00E-03**	57.0	*4.13E-02*	*7.46E-04*	*70.6*
7.95E-01	**1.50E-02**	45.7	*6.16E-02*	*1.11E-03*	*69.7*
1.61E+00	**3.20E-02**	36.3	*9.18E-02*	*1.66E-03*	*68.3*
2.40E+00	**5.00E-02**	31.1	*1.37E-01*	*2.48E-03*	*66.2*
3.18E+00	**7.00E-02**	28.2	*2.03E-01*	*3.70E-03*	*63.0*
4.65E+00	**1.14E-01**	26.7	*3.01E-01*	*5.51E-03*	*58.6*
7.37E+00	**2.29E-01**	25.9	*4.45E-01*	*8.23E-03*	*53.1*
9.93E+00	**4.13E-01**	25.3	*6.56E-01*	*1.23E-02*	*47.0*
1.10E+01	**5.39E-01**	25.1	*9.61E-01*	*1.83E-02*	*41.0*
1.16E+01	**6.18E-01**	24.9	*1.40E+00*	*2.73E-02*	*35.9*
1.22E+01	**7.32E-01**	24.6	*2.00E+00*	*4.07E-02*	*31.8*
1.28E+01	**8.53E-01**	24.2	*2.83E+00*	*6.08E-02*	*29.0*
1.31E+01	**9.41E-01**	23.9	*3.91E+00*	*9.07E-02*	*27.0*
1.33E+01	**1.00E+00**	23.5	*5.26E+00*	*1.35E-01*	*25.8*
			6.84E+00	*2.02E-01*	*25.0*
			8.55E+00	*3.01E-01*	*24.5*
			1.03E+01	*4.49E-01*	*24.3*
			1.19E+01	*6.70E-01*	*24.1*
			1.33E+01	*1.00E+00*	*24.0*

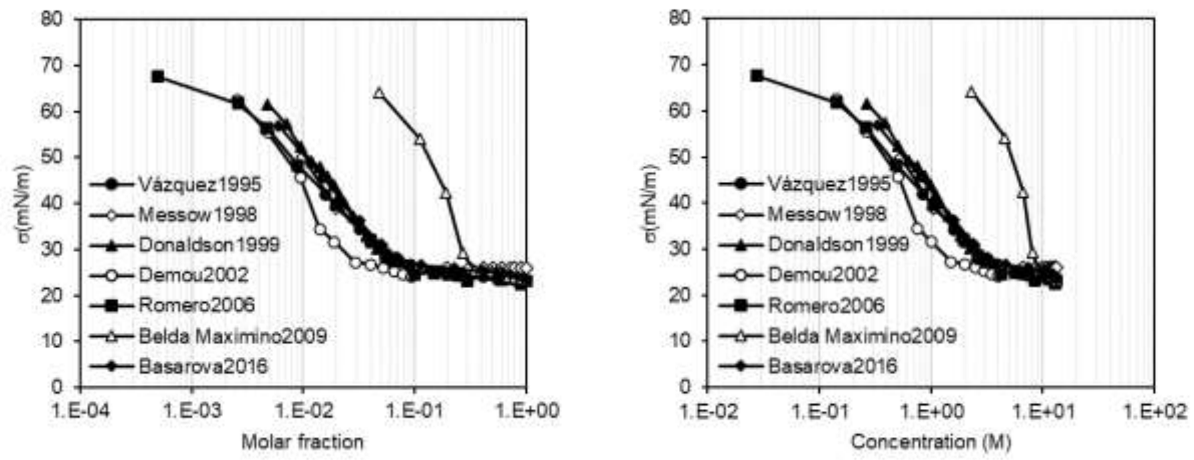

Fig. S47 (a): Comparison of the experimental surface tension data for propan-1-ol / water mixtures.

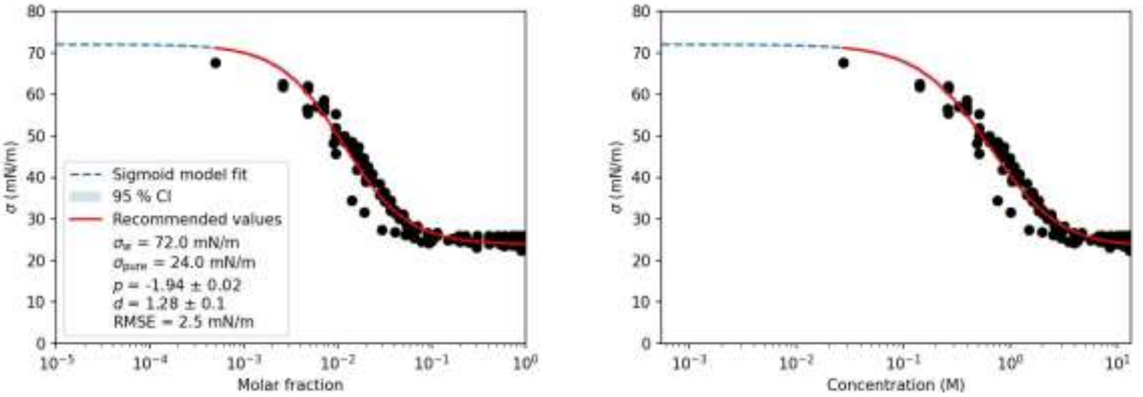

Fig. S47 (b): Surface tension fit with the Sigmoid model (*Kleinheins et al. 2023*) for propan-1-ol / water mixtures. Solid red line: model fit inside the concentration range covered by experimental data, reported as recommended values. Blue shading: fit parameters with 95 % confidence interval (CI). RMSE: root mean squared error. Markers: data used for fitting.

Comment

The data from *Belda Maximino 2009* were not included for the fitting, because they differ from the other surface tension isotherms.

AK7: propan-2-ol

Vázquez et al. 1995 **Table**			Semenov et al. 2014 **Graph (experimental points)**			***Recommended***		
**C (M)**	**Molar fraction**	**σ (mN/m)**	**C (M)**	**Molar fraction**	**σ (mN/m)**	***C (M)***	***Molar fraction***	***σ (mN/m)***
0.00E+00	**0.00E+00**	72.01	0.00E+00	**0.00E+00**	73.0	*2.73E-01*	*5.00E-03*	*61.2*
8.45E-01	**1.60E-02**	49.58	2.73E-01	**5.00E-03**	57.5	*3.59E-01*	*6.61E-03*	*58.4*
1.61E+00	**3.20E-02**	40.42	5.38E-01	**1.00E-02**	52.5	*4.72E-01*	*8.73E-03*	*55.1*
2.39E+00	**5.00E-02**	34.63	1.28E+00	**2.50E-02**	42.5	*6.18E-01*	*1.15E-02*	*51.5*
3.16E+00	**7.00E-02**	30.57	2.79E+00	**6.00E-02**	30.0	*8.07E-01*	*1.53E-02*	*47.7*
3.89E+00	**9.10E-02**	28.28	3.34E+00	**7.50E-02**	30.0	*1.05E+00*	*2.02E-02*	*43.9*
4.61E+00	**1.14E-01**	26.82	4.18E+00	**1.00E-01**	26.5	*1.36E+00*	*2.66E-02*	*40.2*
6.00E+00	**1.67E-01**	25.27	6.51E+00	**1.90E-01**	25.0	*1.75E+00*	*3.52E-02*	*36.9*
7.31E+00	**2.31E-01**	24.26	6.71E+00	**2.00E-01**	24.5	*2.24E+00*	*4.65E-02*	*34.0*
8.55E+00	**3.10E-01**	23.51	8.94E+00	**3.40E-01**	23.5	*2.84E+00*	*6.15E-02*	*31.6*
9.74E+00	**4.12E-01**	22.68	9.18E+00	**3.60E-01**	24.0	*3.57E+00*	*8.13E-02*	*29.6*
1.09E+01	**5.45E-01**	22.14	1.04E+01	**4.80E-01**	23.5	*4.42E+00*	*1.07E-01*	*28.0*
1.20E+01	**7.30E-01**	21.69	1.05E+01	**5.00E-01**	23.5	*5.38E+00*	*1.42E-01*	*26.8*
1.30E+01	**1.00E+00**	21.22	1.18E+01	**7.00E-01**	23.5	*6.46E+00*	*1.88E-01*	*25.8*
			1.19E+01	**7.10E-01**	23.5	*7.60E+00*	*2.48E-01*	*25.1*
			1.25E+01	**8.60E-01**	23.5	*8.78E+00*	*3.28E-01*	*24.6*
			1.30E+01	**1.00E+00**	23.5	*9.95E+00*	*4.33E-01*	*24.2*
						1.11E+01	*5.73E-01*	*23.9*
						1.21E+01	*7.57E-01*	*23.7*
						1.30E+01	*1.00E+00*	*23.5*

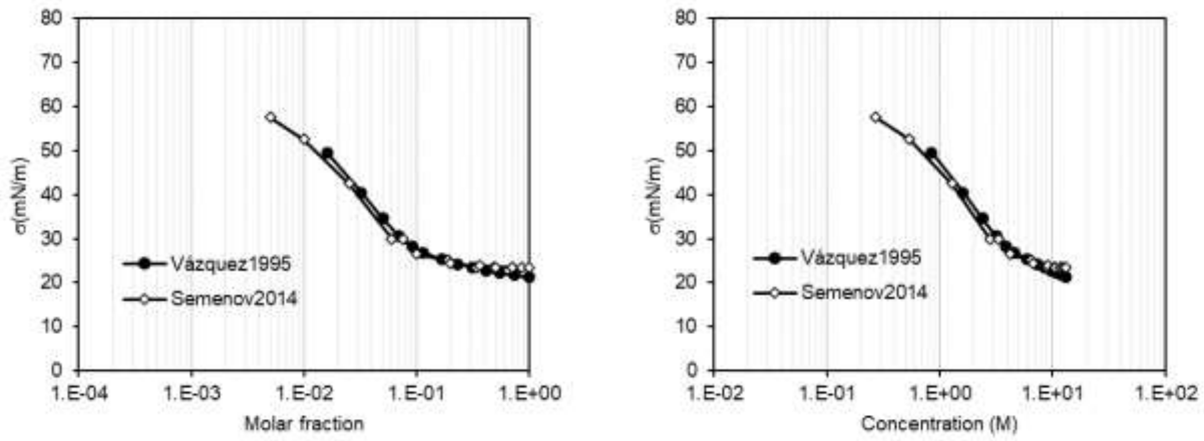

Fig. S48 (a): Experimental surface tension data for propan-2-ol / water mixtures.

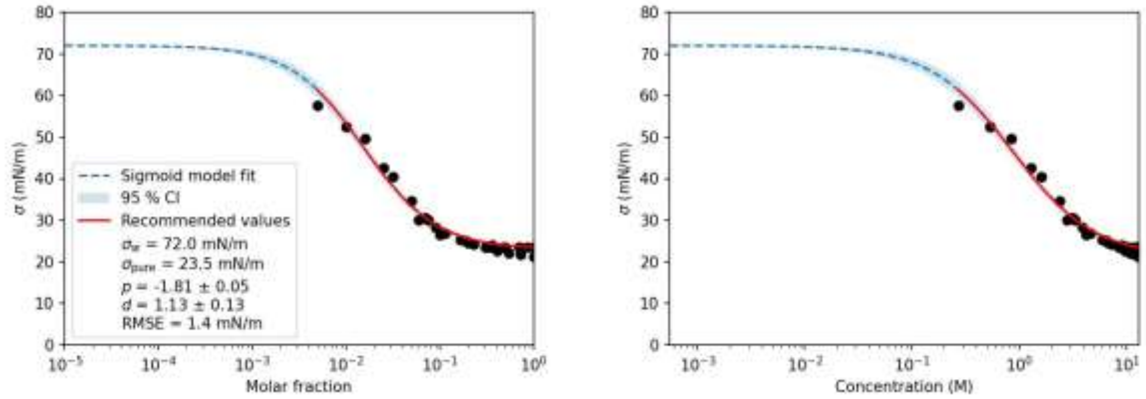

Fig. S48 (b): Surface tension fit with the Sigmoid model (*Kleinheins et al. 2023*) for propan-2-ol / water mixtures. Solid red line: model fit inside the concentration range covered by experimental data, reported as recommended values. Blue shading: fit parameters with 95 % confidence interval (CI). RMSE: root mean squared error. Markers: data used for fitting.

Comment: N/A

AK8: ethylene glycol (ethane-1,2-diol)

Nakanish et al. 1971 **Table**			Messow et al. 1998 **Graph (experimental points)**			***Recommended***		
**C (M)**	**Molar fraction**	**σ (mN/m)**	**C (M)**	**Molar fraction**	**σ (mN/m)**	***C (M)***	***Molar fraction***	***σ (mN/m)***
0.00E+00	**0.00E+00**	71.15	0.00E+00	**0.00E+00**	72.0	*4.59E-01*	*8.40E-03*	*69.7*
4.59E-01	**8.40E-03**	69.33	4.09E+00	**8.70E-02**	61.0	*5.87E-01*	*1.08E-02*	*69.3*
7.82E-01	**1.45E-02**	68.58	6.18E+00	**1.45E-01**	58.0	*7.50E-01*	*1.39E-02*	*68.8*
1.30E+00	**2.46E-02**	67.54	8.44E+00	**2.23E-01**	56.0	*9.57E-01*	*1.79E-02*	*68.1*
1.61E+00	**3.09E-02**	66.84	1.06E+01	**3.19E-01**	53.0	*1.22E+00*	*2.30E-02*	*67.4*
2.36E+00	**4.66E-02**	65.04	1.26E+01	**4.32E-01**	52.0	*1.55E+00*	*2.95E-02*	*66.6*
2.74E+00	**5.50E-02**	64.19	1.46E+01	**5.81E-01**	50.0	*1.96E+00*	*3.80E-02*	*65.7*
3.27E+00	**6.71E-02**	63.37	1.57E+01	**6.96E-01**	49.0	*2.46E+00*	*4.89E-02*	*64.7*
4.30E+00	**9.23E-02**	61.58	1.69E+01	**8.41E-01**	48.0	*3.09E+00*	*6.28E-02*	*63.5*
5.03E+00	**1.12E-01**	60.25	1.74E+01	**9.08E-01**	47.5	*3.84E+00*	*8.08E-02*	*62.2*
5.85E+00	**1.35E-01**	59.54	1.79E+01	**1.00E+00**	47.0	*4.74E+00*	*1.04E-01*	*60.8*
6.73E+00	**1.62E-01**	58.28				*5.80E+00*	*1.34E-01*	*59.3*
8.58E+00	**2.28E-01**	56.02				*7.02E+00*	*1.72E-01*	*57.7*
1.04E+01	**3.07E-01**	54.13				*8.39E+00*	*2.21E-01*	*56.0*
1.21E+01	**4.04E-01**	52.08				*9.90E+00*	*2.84E-01*	*54.4*
1.40E+01	**5.34E-01**	49.99				*1.15E+01*	*3.66E-01*	*52.7*
1.59E+01	**7.21E-01**	48.28				*1.32E+01*	*4.70E-01*	*51.1*
1.79E+01	**1.00E+00**	46.24				*1.48E+01*	*6.05E-01*	*49.5*
						1.64E+01	*7.78E-01*	*48.0*
						1.80E+01	*1.00E+00*	*46.6*

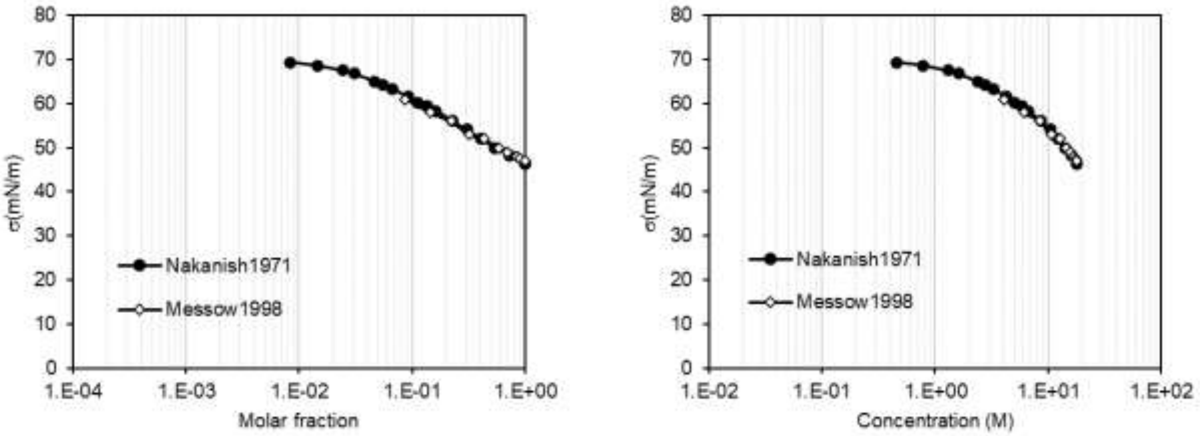

Fig. S49 (a): Comparison of the experimental surface tension data for ethylene glycol / water mixtures.

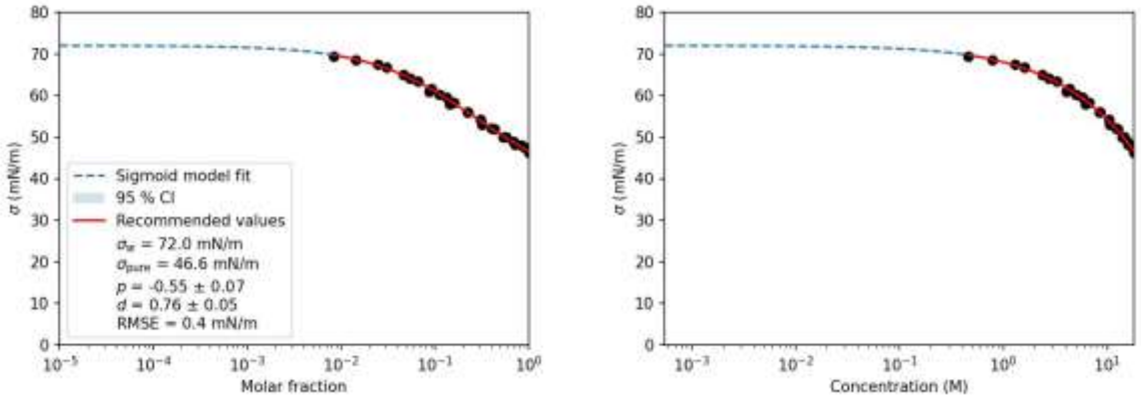

Fig. S49 (b): Surface tension fit with the Sigmoid model (*Kleinheins et al. 2023*) for ethylene glycol / water mixtures. Solid red line: model fit inside the concentration range covered by experimental data, reported as recommended values. Blue shading: fit parameters with 95 % confidence interval (CI). RMSE: root mean squared error. Markers: data used for fitting.

Comment:

Nakanish et al. 1971 : data reported at 30°C.

AK9: propylene glycol (propane-1,2-diol)

Nakanish et al. 1971 **Table**			Hoke et al. 1992 **Table**			Romero et al. 2006 **Table**		
**C (M)**	**Molar fraction**	**σ (mN/m)**	**C (M)**	**Molar fraction**	**σ (mN/m)**	**C (M)**	**Molar fraction**	**σ (mN/m)**
0.00E+00	**0.00E+00**	71.15	2.32E+00	**4.80E-02**	56.7	0.00E+00	**0.00E+00**	71.8
2.36E-01	**4.30E-03**	68.43	4.25E+00	**1.00E-01**	51.0	3.38E-01	**6.20E-03**	68.2
6.53E-01	**1.22E-02**	65.00	7.08E+00	**2.10E-01**	45.8	1.23E+00	**2.37E-02**	61.1
8.97E-01	**1.70E-02**	63.32	8.95E+00	**3.20E-01**	43.2	1.76E+00	**3.52E-02**	57.7
1.06E+00	**2.03E-02**	61.92	1.03E+01	**4.30E-01**	41.0	2.17E+00	**4.44E-02**	57.1
1.51E+00	**2.97E-02**	59.41	1.20E+01	**6.50E-01**	39.4	2.48E+00	**5.17E-02**	55.5
1.97E+00	**3.98E-02**	57.75	1.29E+01	**8.10E-01**	38.5	3.60E+00	**8.10E-02**	52.3
2.39E+00	**4.97E-02**	55.88	1.36E+01	**1.00E+00**	36.6	4.02E+00	**9.30E-02**	50.8
2.89E+00	**6.19E-02**	53.92				4.90E+00	**1.21E-01**	48.3
4.03E+00	**9.34E-02**	50.56				5.40E+00	**1.39E-01**	47.1
4.93E+00	**1.22E-01**	48.13				5.77E+00	**1.53E-01**	45.7
6.71E+00	**1.92E-01**	44.46				6.20E+00	**1.70E-01**	45.6
8.05E+00	**2.62E-01**	41.53				6.71E+00	**1.93E-01**	44.2
8.61E+00	**2.97E-01**	40.28				7.37E+00	**2.24E-01**	43.2
9.42E+00	**3.55E-01**	38.64				7.72E+00	**2.43E-01**	42.8
1.09E+01	**4.97E-01**	37.56				8.67E+00	**3.00E-01**	41.8
1.22E+01	**6.83E-01**	36.26				9.20E+00	**3.38E-01**	40.7
1.36E+01	**1.00E+00**	35.46				9.44E+00	**3.57E-01**	40.3
						9.99E+00	**4.03E-01**	39.4
						1.03E+01	**4.34E-01**	39.0
						1.06E+01	**4.60E-01**	38.1
						1.11E+01	**5.25E-01**	37.5
						1.14E+01	**5.58E-01**	36.8
						1.16E+01	**5.82E-01**	36.6
						1.19E+01	**6.23E-01**	36.1
						1.21E+01	**6.69E-01**	35.8
						1.26E+01	**7.54E-01**	35.6
						1.29E+01	**8.06E-01**	35.6
						1.30E+01	**8.36E-01**	35.6
						1.32E+01	**8.83E-01**	35.6
						1.33E+01	**9.04E-01**	35.6
						1.36E+01	**1.00E+00**	35.6

Recommended		
C (M)	***Molar fraction***	***σ (mN/m)***
2.36E-01	*4.30E-03*	*69.0*
3.13E-01	*5.73E-03*	*68.2*
4.14E-01	*7.63E-03*	*67.3*
5.48E-01	*1.02E-02*	*66.1*
7.22E-01	*1.35E-02*	*64.8*
9.49E-01	*1.80E-02*	*63.2*
1.24E+00	*2.40E-02*	*61.3*
1.62E+00	*3.20E-02*	*59.3*
2.09E+00	*4.26E-02*	*57.1*
2.69E+00	*5.68E-02*	*54.7*
3.41E+00	*7.57E-02*	*52.2*
4.27E+00	*1.01E-01*	*49.7*

5.28E+00	*1.34E-01*	*47.3*
6.41E+00	*1.79E-01*	*45.0*
7.64E+00	*2.38E-01*	*42.9*
8.92E+00	*3.18E-01*	*41.1*
1.02E+01	*4.23E-01*	*39.4*
1.14E+01	*5.63E-01*	*38.0*
1.26E+01	*7.51E-01*	*36.9*
1.36E+01	*1.00E+00*	*35.9*

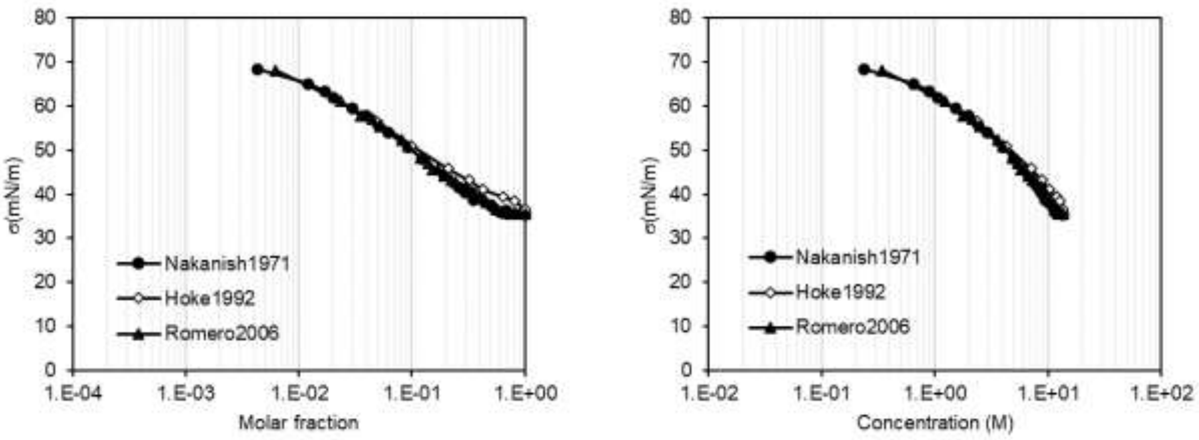

Fig. S50 (a): Comparison of the experimental surface tension data for propylene glycol / water mixtures.

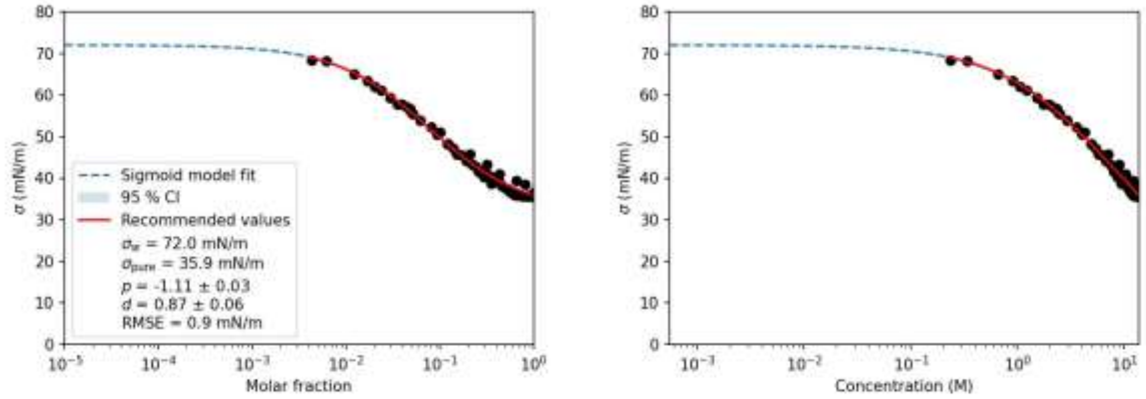

Fig. S50 (b): Surface tension fit with the Sigmoid model (*Kleinheins et al. 2023*) for propylene glycol / water mixtures. Solid red line: model fit inside the concentration range covered by experimental data, reported as recommended values. Blue shading: fit parameters with 95 % confidence interval (CI). RMSE: root mean squared error. Markers: data used for fitting.

Comment:

Nakanish et al. 1971 : data reported at 30°C.

AK10: propan-1,3-diol

Nakanish et al. 1971 **Table**			Romero et al. 2006 **Table**			***Recommended***		
**C (M)**	**Molar fraction**	**σ (mN/m)**	**C (M)**	**Molar fraction**	**σ (mN/m)**	***C (M)***	***Molar fraction***	***σ (mN/m)***
0.00E+00	**0.00E+00**	71.15	0.00E+00	**0.00E+00**	71.8	*2.20E-01*	*4.00E-03*	*69.3*
3.81E-01	**7.00E-03**	68.18	2.20E-01	**4.00E-03**	71.0	*2.92E-01*	*5.35E-03*	*68.7*
4.76E-01	**8.80E-03**	67.38	2.79E-01	**5.10E-03**	70.4	*3.89E-01*	*7.15E-03*	*68.1*
8.18E-01	**1.54E-02**	65.23	1.28E+00	**2.48E-02**	66.8	*5.17E-01*	*9.56E-03*	*67.4*
1.29E+00	**2.50E-02**	62.72	2.41E+00	**5.00E-02**	63.7	*6.84E-01*	*1.28E-02*	*66.6*
1.65E+00	**3.26E-02**	61.74	3.11E+00	**6.74E-02**	62.0	*9.04E-01*	*1.71E-02*	*65.6*
2.14E+00	**4.35E-02**	60.25	4.28E+00	**1.00E-01**	60.3	*1.19E+00*	*2.29E-02*	*64.5*
3.02E+00	**6.51E-02**	57.75	5.75E+00	**1.50E-01**	56.5	*1.56E+00*	*3.06E-02*	*63.4*
3.39E+00	**7.47E-02**	57.10	6.93E+00	**2.00E-01**	54.5	*2.02E+00*	*4.09E-02*	*62.1*
4.03E+00	**9.29E-02**	55.60	8.74E+00	**2.99E-01**	53.6	*2.61E+00*	*5.47E-02*	*60.7*
4.56E+00	**1.09E-01**	54.97	1.01E+01	**3.98E-01**	51.8	*3.33E+00*	*7.31E-02*	*59.2*
5.07E+00	**1.26E-01**	54.11	1.18E+01	**5.93E-01**	49.4	*4.20E+00*	*9.78E-02*	*57.6*
6.75E+00	**1.92E-01**	52.82	1.25E+01	**6.98E-01**	48.7	*5.21E+00*	*1.31E-01*	*56.0*
8.66E+00	**2.94E-01**	51.29	1.38E+01	**1.00E+00**	45.8	*6.36E+00*	*1.75E-01*	*54.4*
1.02E+01	**4.07E-01**	50.39				*7.62E+00*	*2.34E-01*	*52.9*
1.14E+01	**5.40E-01**	49.14				*8.95E+00*	*3.13E-01*	*51.4*
1.31E+01	**8.11E-01**	47.64				*1.03E+01*	*4.18E-01*	*49.9*
1.38E+01	**1.00E+00**	46.95				*1.16E+01*	*5.59E-01*	*48.6*
						1.28E+01	*7.48E-01*	*47.4*
						1.39E+01	*1.00E+00*	*46.3*

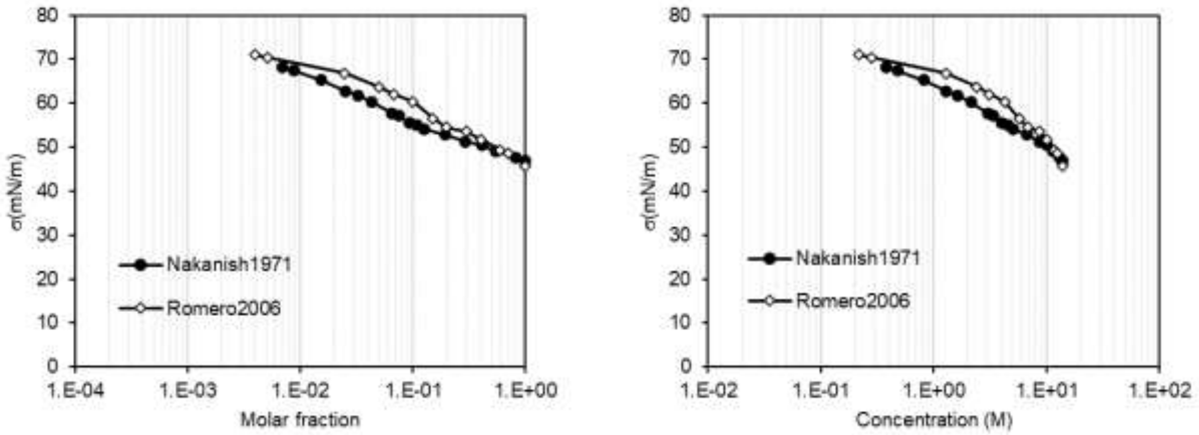

Fig. S51 (a): Comparison of the experimental surface tension data for propan-1,3-diol / water mixtures.

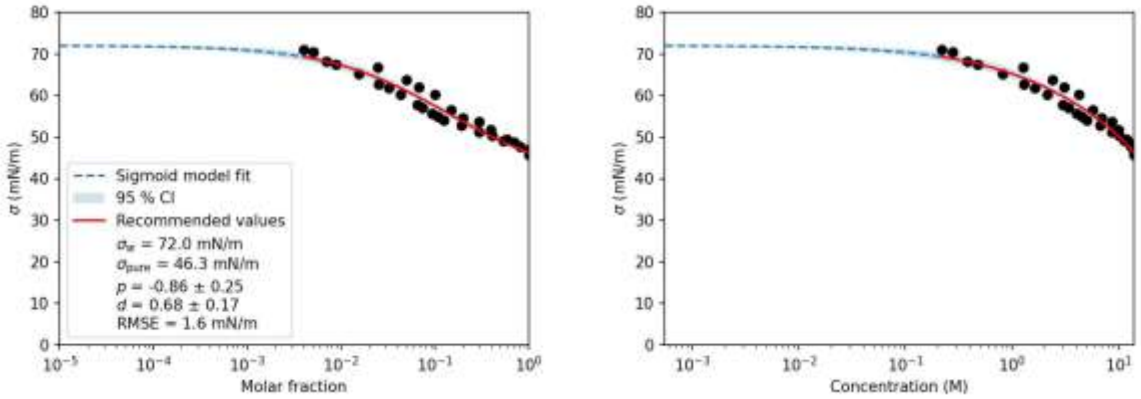

Fig. S51 (b): Surface tension fit with the Sigmoid model (*Kleinheins et al. 2023*) for propan-1.3-diol / water mixtures. Solid red line: model fit inside the concentration range covered by experimental data, reported as recommended values. Blue shading: fit parameters with 95 % confidence interval (CI). RMSE: root mean squared error. Markers: data used for fitting.

Comment:

Nakanish et al. 1971 : data reported at 30°C.

AK11: pentan-1-ol

Romero et al. 2009 **Table**		
**C (M)**	**Molar fraction**	**σ (mN/m)**
0.00E+00	**0.00E+00**	71.81
2.22E-02	**4.00E-04**	56.38
4.65E-02	**8.40E-04**	48.51
6.19E-02	**1.12E-03**	44.98
9.20E-02	**1.67E-03**	39.85
1.12E-01	**2.03E-03**	37.15
1.39E-01	**2.53E-03**	34.31

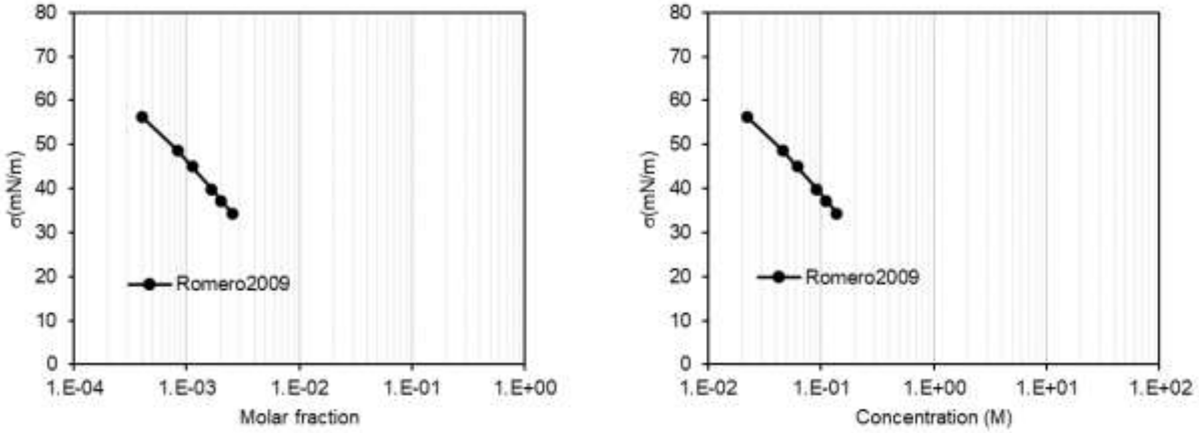

Fig. S52: Experimental surface tension data for Pentan-1-ol / water mixtures.

Comment: N/A

AK12: 1,3-butanediol

Nakanish et al. 1971 **Table**		
**C (M)**	**Molar fraction**	**σ (mN/m)**
0.00E+00	**0.00E+00**	71.15
3.52E-01	**6.50E-03**	64.27
7.57E-01	**1.44E-02**	60.10
1.14E+00	**2.24E-02**	57.85
1.65E+00	**3.37E-02**	54.56
1.90E+00	**3.95E-02**	52.87
2.45E+00	**5.36E-02**	50.91
2.73E+00	**6.10E-02**	49.79
3.44E+00	**8.22E-02**	48.49
3.91E+00	**9.78E-02**	47.04
4.62E+00	**1.24E-01**	45.51
5.54E+00	**1.66E-01**	43.94
7.56E+00	**2.97E-01**	41.41
8.76E+00	**4.24E-01**	40.21
9.58E+00	**5.49E-01**	39.32
1.03E+01	**7.15E-01**	38.73
1.07E+01	**8.22E-01**	37.38
1.12E+01	**1.00E+00**	37.04

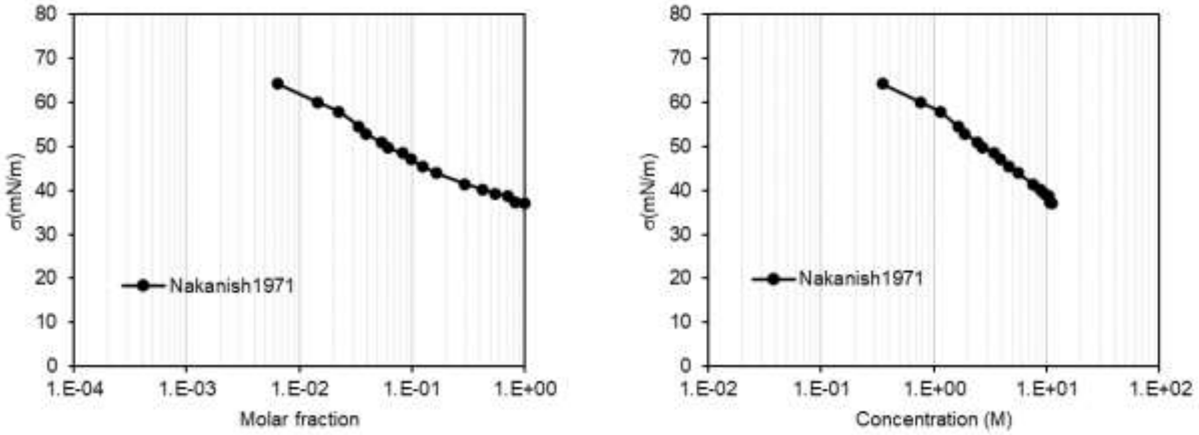

Fig. S53: Experimental surface tension data for 1,4-butanediol / water mixtures.

Comment:

Nakanish et al. 1971 : data reported at 30°C.

AK13: 1,4-butanediol

Nakanish et al. 1971 **Table**		
**C (M)**	**Molar fraction**	**σ (mN/m)**
0.00E+00	**0.00E+00**	71.15
3.26E-01	**6.00E-03**	66.69
6.16E-01	**1.16E-02**	64.47
8.95E-01	**1.72E-02**	62.49
1.09E+00	**2.13E-02**	61.31
1.45E+00	**2.90E-02**	59.64
1.69E+00	**3.46E-02**	58.30
2.26E+00	**4.83E-02**	56.82
2.85E+00	**6.43E-02**	55.05
3.38E+00	**8.00E-02**	54.15
4.50E+00	**1.19E-01**	51.96
5.61E+00	**1.67E-01**	50.73
6.18E+00	**1.97E-01**	50.21
6.71E+00	**2.30E-01**	49.51
7.86E+00	**3.18E-01**	47.95
9.01E+00	**4.46E-01**	46.63
1.01E+01	**6.43E-01**	45.23
1.13E+01	**1.00E+00**	43.79

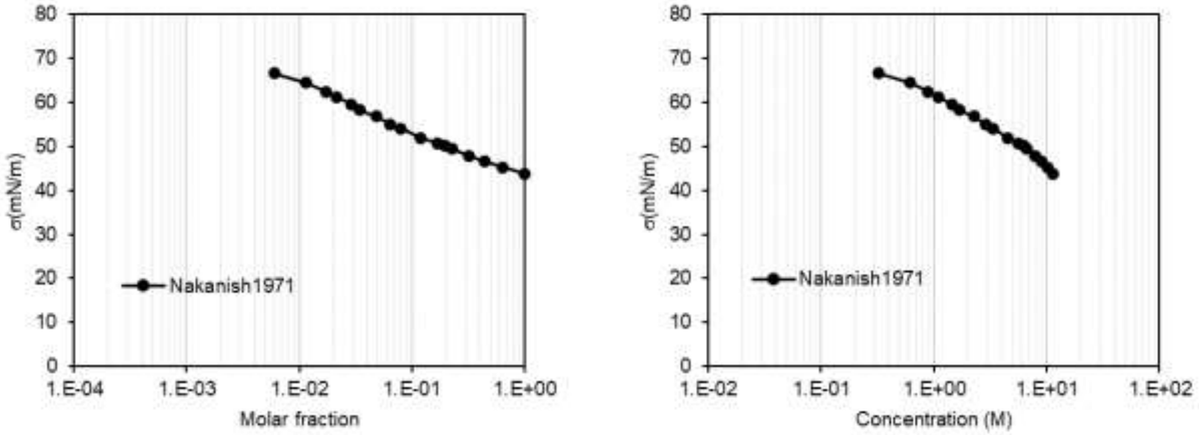

Fig. S54: Experimental surface tension data for 1,4-butanediol / water mixtures.

Comment:

Nakanish et al. 1971 : data reported at 30°C.

AK14: glycerol (propane-1,2,3-triol)

Ernst et al. 1936 **Graph (experimental points)**				Romero et al. 2006 **Table**			Takamura et al. 2012 **Graph (experimental points)**			
**C (M)**	**Molar fraction**	**Mass fraction**	**σ (mN/m)**	**C (M)**	**Molar fraction**	**σ (mN/m)**	**C (M)**	**Molar fraction**	**Mass fraction**	**σ (mN/m)**
0.00E+00	0.00E+00	**0.00E+00**	72.0	0.00E+00	**0.00E+00**	71.8	0.00E+00	0.00E+00	**0.00E+00**	73.2
1.11E+00	2.13E-02	**1.00E-01**	70.5	1.97E+00	**3.97E-02**	69.7	2.27E+00	4.66E-02	**2.00E-01**	71.7
2.27E+00	4.66E-02	**2.00E-01**	69.5	2.29E+00	**4.72E-02**	69.5	4.74E+00	1.15E-01	**4.00E-01**	70.0
3.47E+00	7.73E-02	**3.00E-01**	68.5	3.05E+00	**6.60E-02**	68.8	7.44E+00	2.27E-01	**6.00E-01**	68.5
4.74E+00	1.15E-01	**4.00E-01**	67.9	3.29E+00	**7.24E-02**	68.6	1.01E+01	4.09E-01	**7.80E-01**	67.4
6.06E+00	1.64E-01	**5.00E-01**	67.4	3.97E+00	**9.14E-02**	68.3	1.22E+01	6.64E-01	**9.10E-01**	66.5
7.44E+00	2.27E-01	**6.00E-01**	66.9	4.64E+00	**1.12E-01**	67.9	1.37E+01	1.00E+00	**1.00E+00**	63.4
8.89E+00	3.13E-01	**7.00E-01**	66.5	5.80E+00	**1.53E-01**	67.3				
1.04E+01	4.39E-01	**8.00E-01**	65.7	6.33E+00	**1.75E-01**	67.1				
1.20E+01	6.38E-01	**9.00E-01**	64.5	7.47E+00	**2.29E-01**	66.9				
1.37E+01	1.00E+00	**1.00E+00**	62.5	8.79E+00	**3.07E-01**	66.4				
				1.02E+01	**4.22E-01**	65.8				
				1.09E+01	**4.94E-01**	65.3				
				1.18E+01	**5.99E-01**	64.7				
				1.21E+01	**6.52E-01**	64.4				
				1.25E+01	**7.13E-01**	63.6				
				1.27E+01	**7.51E-01**	63.5				
				1.29E+01	**7.94E-01**	63.5				
				1.34E+01	**9.20E-01**	62.9				
				1.37E+01	**1.00E+00**	63.0				

Recommended		
C (M)	***Molar fraction***	***σ (mN/m)***
1.11E+00	*2.13E-02*	*70.3*
1.34E+00	*2.61E-02*	*70.1*
1.62E+00	*3.19E-02*	*70.0*
1.94E+00	*3.91E-02*	*69.8*
2.32E+00	*4.79E-02*	*69.6*
2.76E+00	*5.87E-02*	*69.4*
3.27E+00	*7.18E-02*	*69.1*
3.85E+00	*8.80E-02*	*68.9*
4.50E+00	*1.08E-01*	*68.6*
5.22E+00	*1.32E-01*	*68.3*
6.01E+00	*1.62E-01*	*67.9*
6.85E+00	*1.98E-01*	*67.5*
7.73E+00	*2.42E-01*	*67.1*
8.64E+00	*2.97E-01*	*66.7*
9.56E+00	*3.63E-01*	*66.2*
1.05E+01	*4.45E-01*	*65.7*
1.14E+01	*5.45E-01*	*65.1*
1.22E+01	*6.67E-01*	*64.4*
1.30E+01	*8.17E-01*	*63.8*
1.37E+01	*1.00E+00*	*63.0*

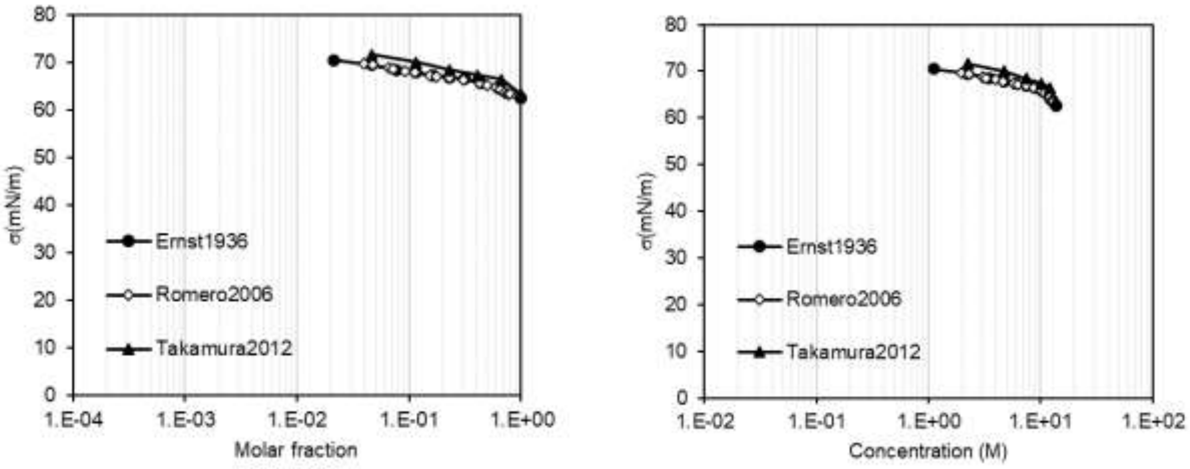

Fig. S55 (a): Comparison of the experimental surface tension data for glycerol / water mixtures.

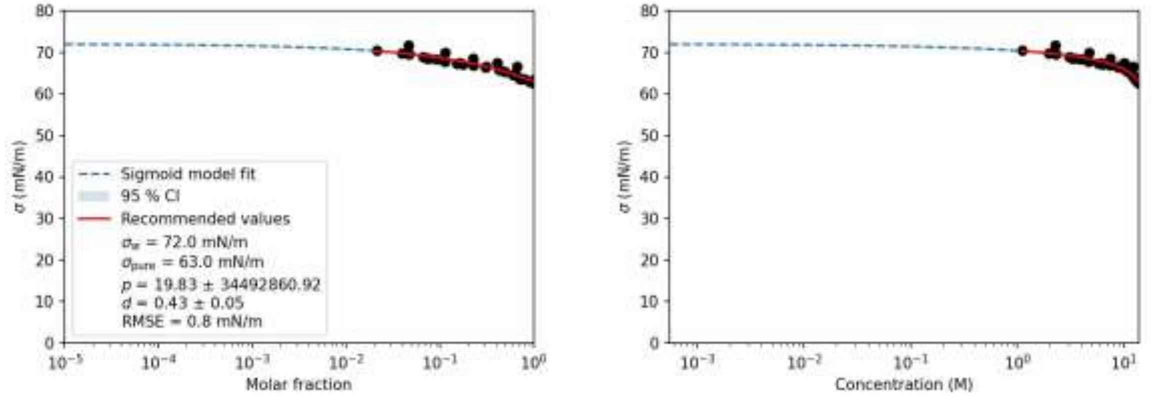

Fig. S55 (b): Surface tension fit with the Sigmoid model (*Kleinheins et al. 2023*) for glycerol / water mixtures. Solid red line: model fit inside the concentration range covered by experimental data, reported as recommended values. Blue shading: fit parameters with 95 % confidence interval (CI). RMSE: root mean squared error. Markers: data used for fitting.

Comment: N/A

AK15: phenol (benzenol)

Dynarowicz 1994	**Graph (experimental points)**		
**C (M)**	**Molar fraction**	**logC (M)**	**σ (mN/m)**
9.91E-03	1.79E-04	**-2.00**	73.6
2.51E-02	4.52E-04	**-1.60**	71.8
5.03E-02	9.08E-04	**-1.30**	67.2
1.02E-01	1.85E-03	**-0.99**	61.6
1.53E-01	2.79E-03	**-0.81**	57.0
2.14E-01	3.90E-03	**-0.67**	53.7

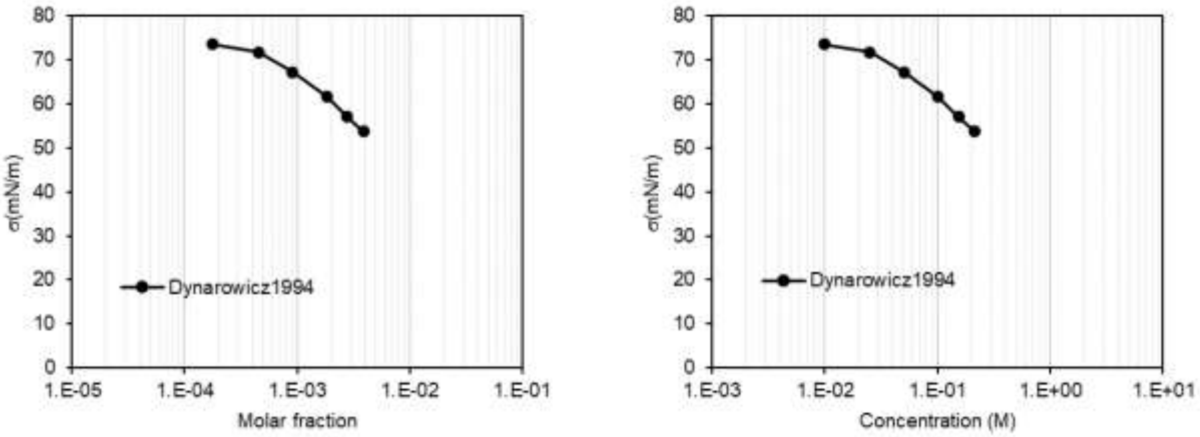

Fig. S56: Experimental surface tension data for phenol / water mixtures.

Comment: N/A

AK16: hexan-1-ol

Romero et al. 2009 **Table**			Le et al. 2012 **Graph (experimental points)**			***Recommended***		
**C (M)**	**Molar fraction**	**σ (mN/m)**	**C (M)**	**Molar fraction**	**σ (mN/m)**	***C (M)***	***Molar fraction***	***σ (mN/m)***
0.00E+00	**0.00E+00**	71.81	**3.50E-04**	6.30E-06	70.5	*3.50E-04*	*6.30E-06*	*71.6*
1.39E-02	**2.50E-04**	47.52	**6.80E-04**	1.22E-05	69.7	*4.55E-04*	*8.19E-06*	*71.4*
2.27E-02	**4.10E-04**	41.29	**1.10E-03**	1.98E-05	69.0	*5.92E-04*	*1.07E-05*	*71.1*
3.38E-02	**6.10E-04**	36.20	**1.30E-03**	2.34E-05	68.4	*7.70E-04*	*1.39E-05*	*70.8*
3.71E-02	**6.70E-04**	34.64	**1.70E-03**	3.06E-05	67.8	*1.00E-03*	*1.80E-05*	*70.3*
4.15E-02	**7.50E-04**	33.20	**2.20E-03**	3.96E-05	66.8	*1.30E-03*	*2.35E-05*	*69.7*
5.14E-02	**9.30E-04**	30.21	**2.60E-03**	4.68E-05	65.5	*1.69E-03*	*3.05E-05*	*68.8*
			**3.40E-03**	6.12E-05	64.1	*2.20E-03*	*3.97E-05*	*67.7*
			**4.30E-03**	7.74E-05	63.0	*2.87E-03*	*5.16E-05*	*66.2*
			**5.30E-03**	9.55E-05	61.2	*3.73E-03*	*6.71E-05*	*64.2*
			**6.90E-03**	1.24E-04	59.4	*4.85E-03*	*8.73E-05*	*61.7*
			**8.75E-03**	1.58E-04	56.2	*6.30E-03*	*1.14E-04*	*58.8*
			**1.08E-02**	1.95E-04	52.6	*8.20E-03*	*1.48E-04*	*55.4*
			**1.10E-02**	1.98E-04	50.0	*1.07E-02*	*1.92E-04*	*51.7*
			**1.60E-02**	2.88E-04	45.8	*1.39E-02*	*2.50E-04*	*47.8*
			**1.70E-02**	3.07E-04	44.6	*1.80E-02*	*3.25E-04*	*43.9*
			**1.95E-02**	3.52E-04	43.2	*2.34E-02*	*4.23E-04*	*40.4*
						3.04E-02	*5.50E-04*	*37.2*
						3.96E-02	*7.15E-04*	*34.6*
						5.14E-02	*9.30E-04*	*32.4*

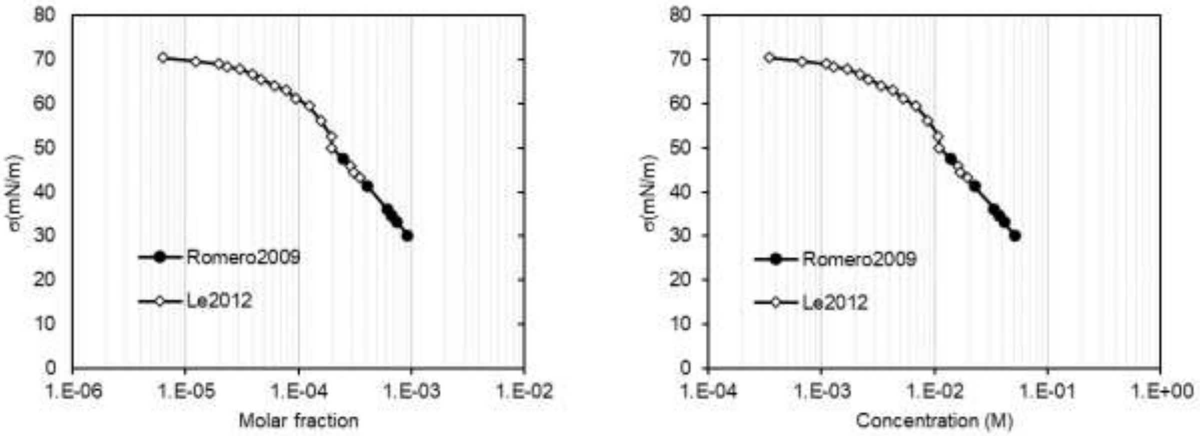

Fig. S57 (a): Comparison of the experimental surface tension data for hexan-1-ol / water mixtures.

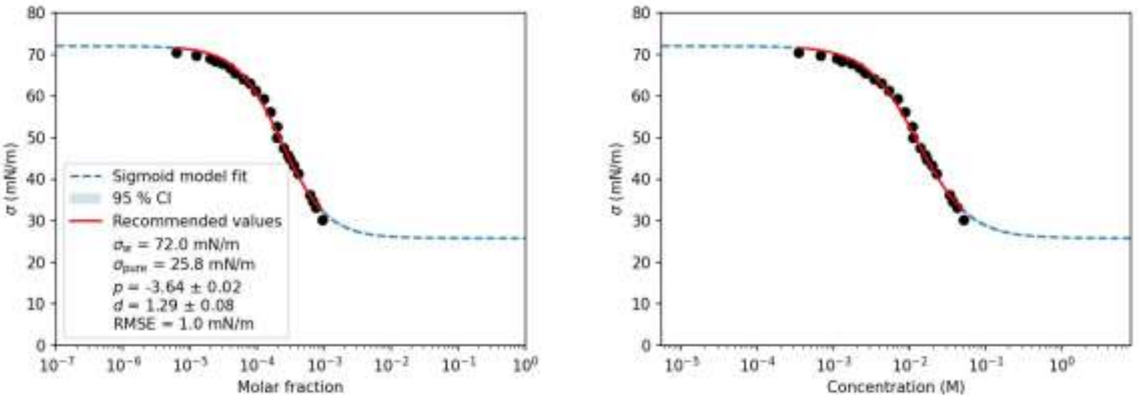

Fig. S57 (b): Surface tension fit with the Sigmoid model (*Kleinheins et al. 2023*) for hexan-1-ol / water mixtures. Solid red line: model fit inside the concentration range covered by experimental data, reported as recommended values. Blue shading: fit parameters with 95 % confidence interval (CI). RMSE: root mean squared error. Markers: data used for fitting.

Comment: N/A

AK17: hexan-2-ol

Le et al. 2012	**Graph (experimental points)**	
**C (M)**	**Molar fraction**	**σ (mN/m)**
**1.40E-04**	2.52E-06	70.9
**1.80E-04**	3.24E-06	70.8
**2.20E-04**	3.96E-06	70.8
**2.80E-04**	5.04E-06	70.6
**3.70E-04**	6.66E-06	70.5
**5.80E-04**	1.04E-05	70.0
**7.00E-04**	1.26E-05	69.7
**8.80E-04**	1.58E-05	69.3
**1.00E-03**	1.80E-05	69.1
**1.40E-03**	2.52E-05	68.4
**1.80E-03**	3.24E-05	67.9
**2.30E-03**	4.14E-05	67.0
**2.80E-03**	5.04E-05	66.1
**3.60E-03**	6.48E-05	64.8
**4.60E-03**	8.28E-05	63.2
**5.70E-03**	1.03E-04	62.1
**7.20E-03**	1.30E-04	60.3
**9.25E-03**	1.67E-04	58.5
**1.08E-02**	1.95E-04	56.6
**1.20E-02**	2.16E-04	55.5
**1.50E-02**	2.70E-04	53.1
**1.85E-02**	3.34E-04	50.1

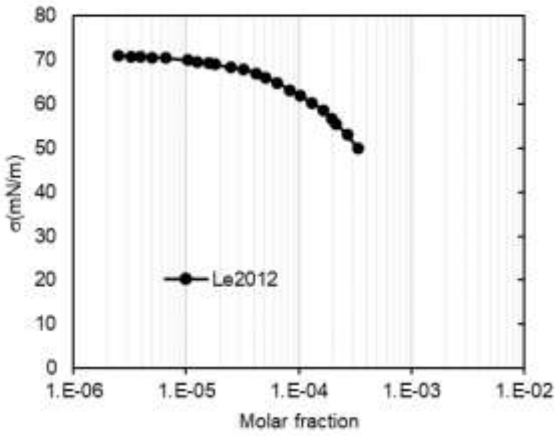

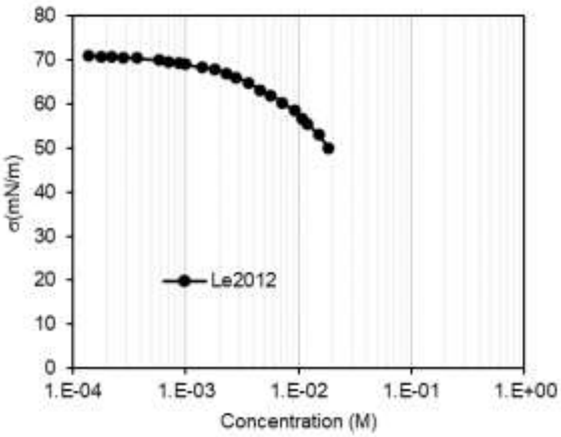

Fig. S58: Experimental surface tension data for hexan-2-ol / water mixtures.

Comment: N/A

AK18: 2,3-dimethylbutan-2-ol

Le et al. 2012	**Graph (experimental points)**	
**C (M)**	**Molar fraction**	**σ (mN/m)**
**5.10E-05**	9.18E-07	71.2
**6.60E-05**	1.19E-06	71.1
**8.30E-05**	1.49E-06	71.0
**1.10E-04**	1.98E-06	71.0
**1.26E-04**	2.27E-06	70.8
**1.60E-04**	2.89E-06	70.6
**1.98E-04**	3.56E-06	70.5
**2.48E-04**	4.46E-06	70.6
**3.20E-04**	5.76E-06	70.5
**4.00E-04**	7.20E-06	70.5
**5.20E-04**	9.36E-06	70.5
**6.50E-04**	1.17E-05	70.0
**8.00E-04**	1.44E-05	70.0
**1.01E-03**	1.82E-05	69.7
**1.32E-03**	2.38E-05	69.3
**1.60E-03**	2.88E-05	69.3
**2.02E-03**	3.63E-05	68.7
**2.61E-03**	4.70E-05	68.3
**3.20E-03**	5.76E-05	67.7
**4.10E-03**	7.38E-05	67.1
**5.20E-03**	9.37E-05	66.4
**6.51E-03**	1.17E-04	65.6
**8.30E-03**	1.50E-04	64.0
**1.00E-02**	1.80E-04	63.1
**1.30E-02**	2.34E-04	61.7
**1.67E-02**	3.01E-04	60.1
**2.00E-02**	3.61E-04	58.4
**2.60E-02**	4.69E-04	56.2
**3.10E-02**	5.60E-04	53.6

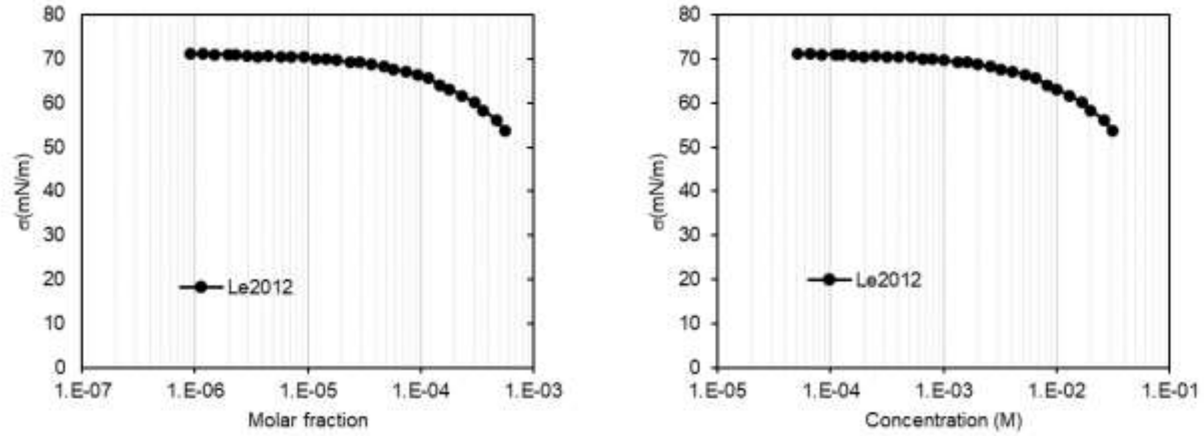

Fig. S59: Experimental surface tension data for 2,3-dimethylbutan-2-ol / water mixtures.

Comment: N/A

AK19: 2-methylpentan-2-ol

Le et al. 2012	Graph (experimental points)	
**C (M)**	**Molar fraction**	**σ (mN/m)**
**4.50E-04**	8.10E-06	70.5
**1.00E-03**	1.80E-05	69.7
**1.31E-03**	2.36E-05	69.2
**1.66E-03**	2.99E-05	68.6
**2.05E-03**	3.69E-05	68.2
**2.64E-03**	4.75E-05	67.6
**3.31E-03**	5.96E-05	66.7
**4.20E-03**	7.56E-05	65.7
**5.30E-03**	9.55E-05	65.0
**6.60E-03**	1.19E-04	64.0
**8.40E-03**	1.51E-04	62.3
**1.04E-02**	1.87E-04	60.2
**1.16E-02**	2.09E-04	59.3
**1.79E-02**	3.23E-04	54.8

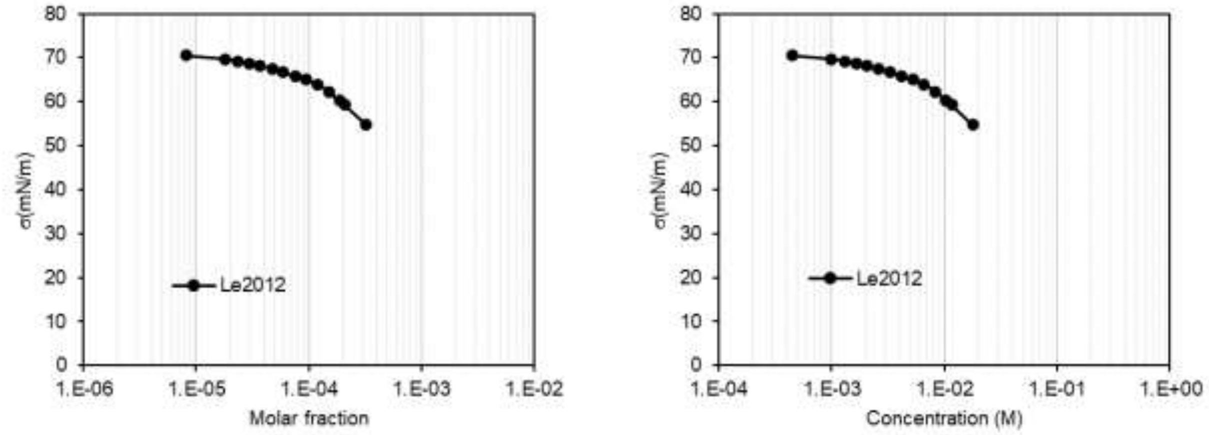

Fig. S60: Experimental surface tension data for 2-methylpentan-2-ol / water mixtures.

Comment: N/A

AK20: 1,5-pentanediol

Glinski et al. 2000a **Graph (experimental points)**		
**C (M)**	**Molar fraction**	**σ (mN/m)**
0.00E+00	**0.00E+00**	72.2
3.51E-02	**6.34E-04**	68.4
6.97E-02	**1.26E-03**	66.2
1.54E-01	**2.82E-03**	59.3
2.72E-01	**5.02E-03**	52.6
5.23E-01	**9.85E-03**	47.5
1.45E+00	**3.00E-02**	41.8
2.22E+00	**4.95E-02**	43.3
2.90E+00	**6.98E-02**	46.1
3.74E+00	**9.98E-02**	48.7
5.65E+00	**2.00E-01**	47.6
9.52E+00	**1.00E+00**	44.2

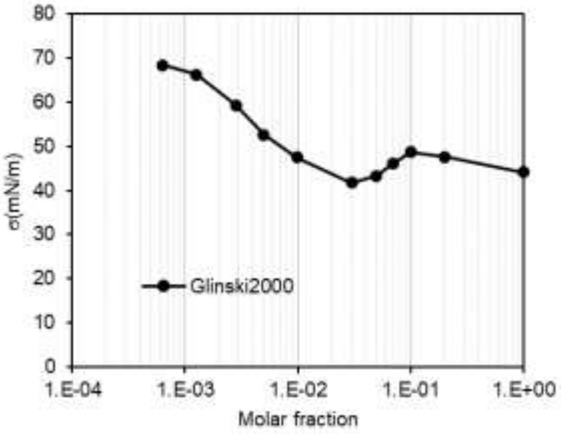

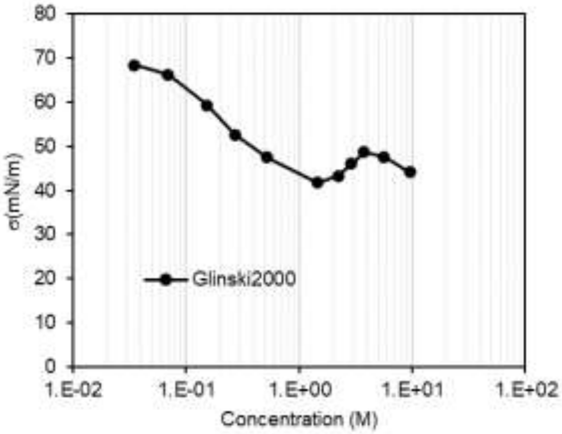

Fig. S61: Experimental surface tension data for 1,5-pentanediol / water mixtures.

Comment: N/A

AK21: p-cresol (4-methylbenzenol)

Dynarowicz 1994	**Graph (experimental points)**		
**C (M)**	**Molar fraction**	**logC (M)**	**σ (mN/m)**
1.56E-03	2.81E-05	**-2.81**	74.1
4.94E-03	8.90E-05	**-2.31**	72.6
9.91E-03	1.79E-04	**-2.00**	67.9
2.02E-02	3.65E-04	**-1.69**	62.2
4.13E-02	7.47E-04	**-1.38**	54.1
6.01E-02	1.09E-03	**-1.22**	48.9
8.22E-02	1.49E-03	**-1.09**	44.8
1.01E-01	1.83E-03	**-1.00**	41.5

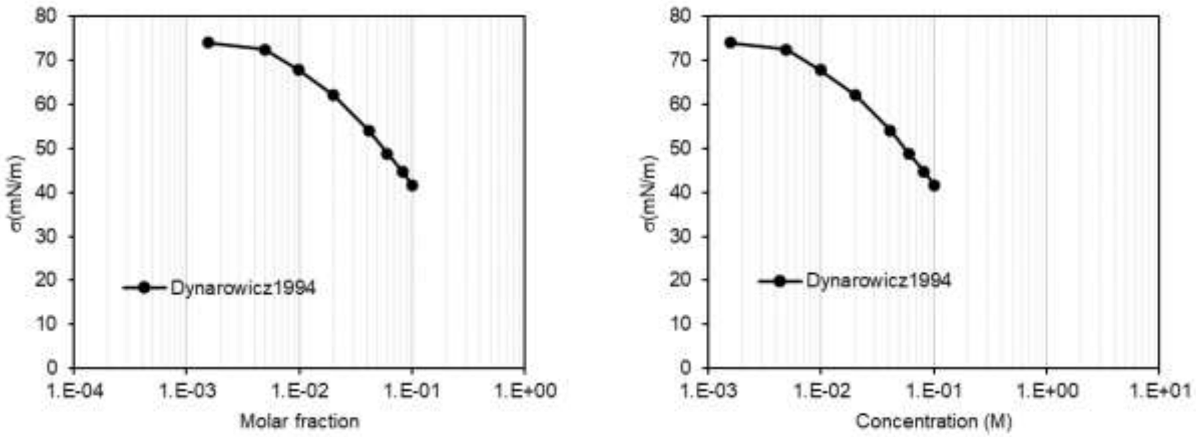

Fig. S62: Experimental surface tension data for p-Cresol / water mixtures.

Comment: N/A

AK22: heptan-1-ol

Glinski et al. 1996 **Graph (experimental points)**				Romero et al. 2009 **Table**			***Recommended***		
**C (M)**	**Molar fraction**	**logC (M)**	**σ (mN/m)**	**C (M)**	**Molar fraction**	**σ (mN/m)**	***C (M)***	***Molar fraction***	***σ (mN/m)***
2.24E-05	4.03E-07	**-4.65**	72.2	0.00E+00	**0.00E+00**	71.81	*2.24E-05*	*4.03E-07*	*71.9*
6.76E-05	1.22E-06	**-4.17**	72.2	5.56E-04	**1.00E-05**	67.47	*3.15E-05*	*5.66E-07*	*71.8*
1.35E-04	2.43E-06	**-3.87**	70.7	1.67E-03	**3.00E-05**	58.79	*4.42E-05*	*7.96E-07*	*71.8*
2.24E-04	4.03E-06	**-3.65**	68.8	2.78E-03	**5.00E-05**	52.52	*6.22E-05*	*1.12E-06*	*71.6*
3.31E-04	5.96E-06	**-3.48**	69.0	8.88E-03	**1.60E-04**	36.36	*8.74E-05*	*1.57E-06*	*71.4*
4.37E-04	7.86E-06	**-3.36**	65.4	9.99E-03	**1.80E-04**	34.68	*1.23E-04*	*2.21E-06*	*71.1*
6.46E-04	1.16E-05	**-3.19**	61.4	1.44E-02	**2.60E-04**	30.31	*1.73E-04*	*3.11E-06*	*70.6*
1.02E-03	1.84E-05	**-2.99**	59.7				*2.43E-04*	*4.37E-06*	*69.9*
1.38E-03	2.49E-05	**-2.86**	56.5				*3.41E-04*	*6.14E-06*	*68.8*
1.86E-03	3.35E-05	**-2.73**	53.3				*4.80E-04*	*8.63E-06*	*67.3*
2.63E-03	4.74E-05	**-2.58**	48.0				*6.74E-04*	*1.21E-05*	*65.0*
4.27E-03	7.68E-05	**-2.37**	42.3				*9.48E-04*	*1.71E-05*	*62.0*
5.01E-03	9.03E-05	**-2.30**	39.7				*1.33E-03*	*2.40E-05*	*58.1*
7.08E-03	1.28E-04	**-2.15**	34.0				*1.87E-03*	*3.37E-05*	*53.5*
8.13E-03	1.46E-04	**-2.09**	33.7				*2.63E-03*	*4.74E-05*	*48.5*
1.00E-02	1.80E-04	**-2.00**	28.6				*3.70E-03*	*6.66E-05*	*43.6*
1.12E-02	2.02E-04	**-1.95**	28.0				*5.20E-03*	*9.36E-05*	*39.2*
							7.30E-03	*1.32E-04*	*35.6*
							1.03E-02	*1.85E-04*	*32.8*
							1.44E-02	*2.60E-04*	*30.8*

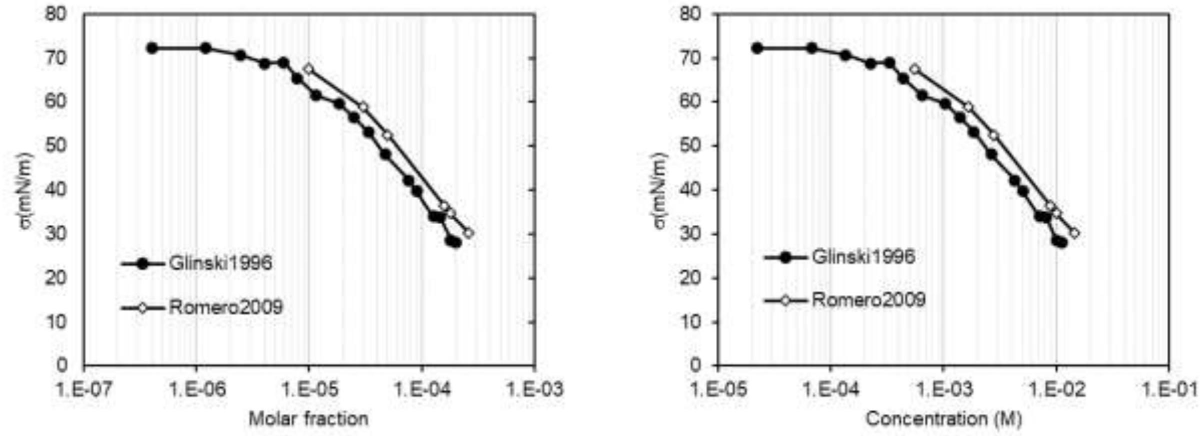

Fig. S63 (a): Comparison of the experimental surface tension data for heptan-1-ol / water mixtures.

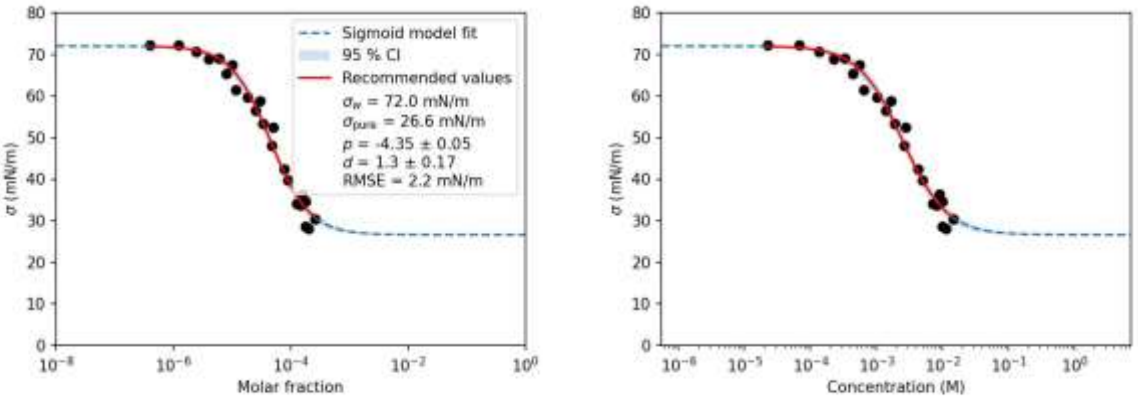

Fig. S63 (b): Surface tension fit with the Sigmoid model (*Kleinheins et al. 2023*) for heptan-1-ol / water mixtures. Solid red line: model fit inside the concentration range covered by experimental data, reported as recommended values. Blue shading: fit parameters with 95 % confidence interval (CI). RMSE: root mean squared error. Markers: data used for fitting.

Comment: N/A

AK23: hexane-1,2-diol

Romero et al. 2007 **Table**		
**C (M)**	**Molar fraction**	**σ (mN/m)**
0.00E+00	**0.00E+00**	71.8
5.52E-02	**1.00E-03**	53.8
8.81E-02	**1.60E-03**	46.6
1.10E-01	**2.00E-03**	43.4
1.85E-01	**3.40E-03**	39.3
2.80E-01	**5.20E-03**	34.7
4.09E-01	**7.70E-03**	30.0
5.64E-01	**1.08E-02**	27.2
7.18E-01	**1.40E-02**	25.7
8.81E-01	**1.75E-02**	25.3
1.21E+00	**2.51E-02**	25.2
1.65E+00	**3.60E-02**	25.0
2.01E+00	**4.60E-02**	24.9
2.19E+00	**5.14E-02**	24.9
2.62E+00	**6.55E-02**	24.8
3.09E+00	**8.31E-02**	24.8
3.42E+00	**9.68E-02**	24.4
3.74E+00	**1.12E-01**	24.1
8.01E+00	**1.00E+00**	23.8

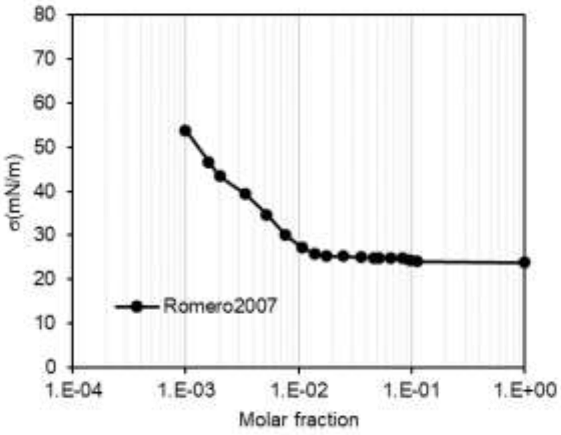

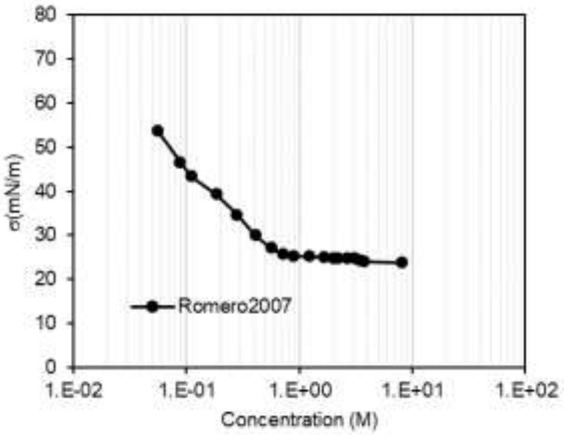

Fig. S64: Experimental surface tension data for hexan-1,2-diol / water mixtures.

Comment: N/A

AK24: hexane-1,6-diol

Romero et al. 2007 **Table**		
**C (M)**	**Molar fraction**	**σ (mN/m)**
0.00E+00	**0.00E+00**	71.8
7.17E-02	**1.30E-03**	62.0
9.35E-02	**1.70E-03**	60.4
1.15E-01	**2.10E-03**	59.4
1.58E-01	**2.90E-03**	57.5
3.06E-01	**5.70E-03**	53.8
3.74E-01	**7.00E-03**	52.1
4.70E-01	**8.90E-03**	50.2
7.19E-01	**1.40E-02**	48.3
8.08E-01	**1.59E-02**	47.5
1.16E+00	**2.37E-02**	46.5
1.70E+00	**3.73E-02**	45.0
2.02E+00	**4.63E-02**	43.6
2.29E+00	**5.45E-02**	42.9
2.87E+00	**7.42E-02**	42.8
3.46E+00	**9.80E-02**	42.7
3.84E+00	**1.17E-01**	42.6

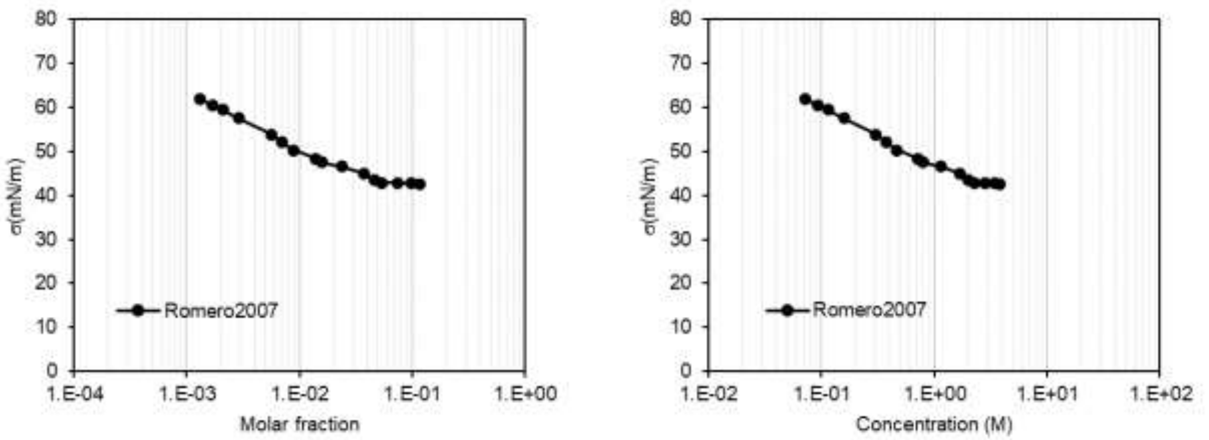

Fig. S65: Experimental surface tension data for hexan-1,6-diol / water mixtures.

Comment: N/A

AK25: hexane-1,5-diol

Romero et al. 2007 **Table**		
**C (M)**	**Molar fraction**	**σ (mN/m)**
0.00E+00	**0.00E+00**	71.8
5.52E-02	**1.00E-03**	62.8
1.15E-01	**2.10E-03**	56.1
2.01E-01	**3.70E-03**	53.6
2.86E-01	**5.30E-03**	51.2
3.79E-01	**7.10E-03**	49.3
4.70E-01	**8.90E-03**	47.9
5.79E-01	**1.11E-02**	46.0
7.43E-01	**1.45E-02**	43.8
9.69E-01	**1.94E-02**	40.3
1.52E+00	**3.24E-02**	38.0
2.18E+00	**5.07E-02**	36.6
2.87E+00	**7.38E-02**	35.8
8.18E+00	**1.00E+00**	33.9

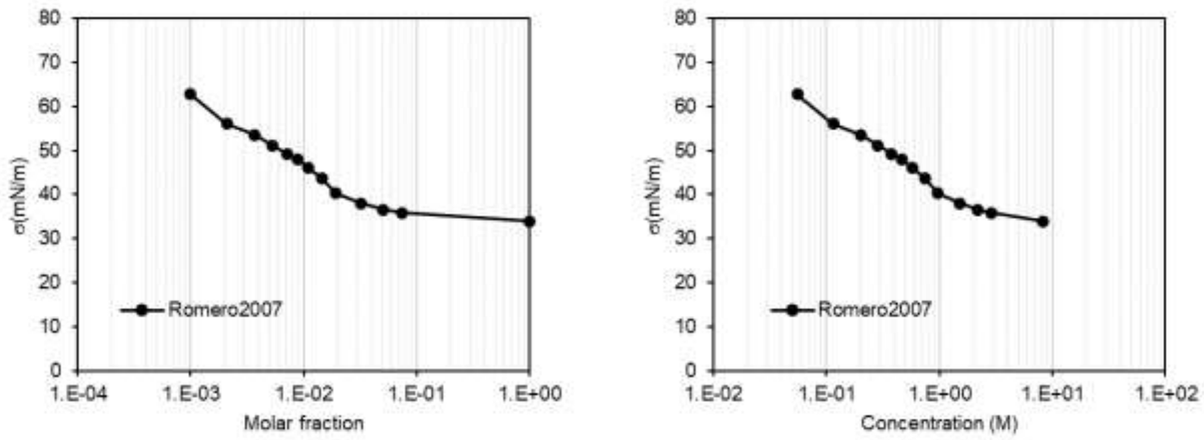

Fig. S66: Experimental surface tension data for hexan-1,5-diol / water mixtures.

Comment: N/A

AK26: hexane-2,5-diol

Romero et al. 2007 **Table**		
**C (M)**	**Molar fraction**	**σ (mN/m)**
0.00E+00	**0.00E+00**	71.8
5.52E-02	**1.00E-03**	67.2
1.48E-01	**2.70E-03**	58.6
2.01E-01	**3.70E-03**	55.2
3.63E-01	**6.80E-03**	51.6
4.65E-01	**8.80E-03**	49.5
5.40E-01	**1.03E-02**	48.7
6.95E-01	**1.35E-02**	46.8
8.50E-01	**1.68E-02**	45.2
1.27E+00	**2.63E-02**	42.1
1.69E+00	**3.70E-02**	40.0
2.02E+00	**4.62E-02**	39.0
2.56E+00	**6.30E-02**	37.4
3.05E+00	**8.08E-02**	37.0
3.41E+00	**9.60E-02**	36.8
3.75E+00	**1.12E-01**	36.2
8.10E+00	**1.00E+00**	31.6

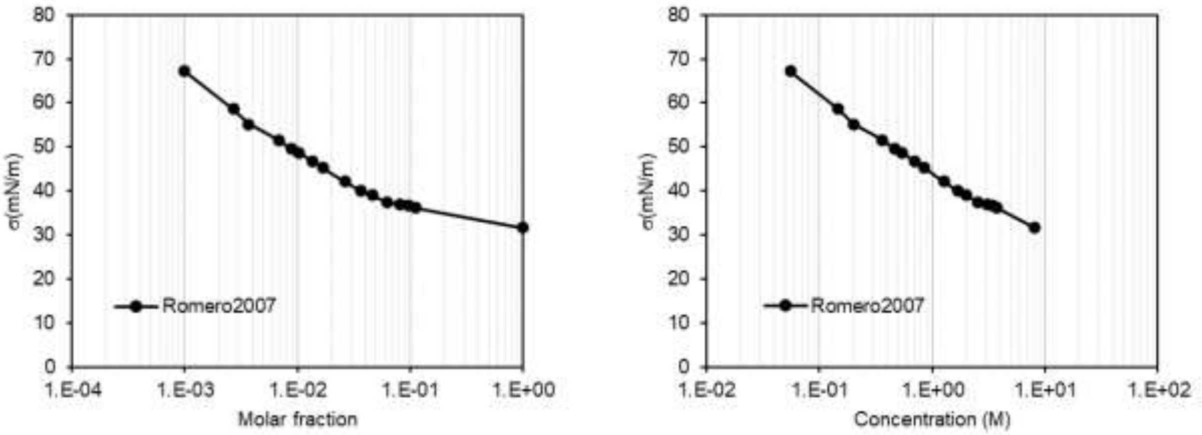

Fig. S67: Experimental surface tension data for hexan-2,5-diol / water mixtures.

Comment: N/A

AK27: 4-ethylphenol

Dynarowicz 1994	**Graph (experimental points)**		
**C (M)**	**Molar fraction**	**logC (M)**	**σ (mN/m)**
1.00E-04	1.80E-06	**-4.00**	74.0
6.46E-04	1.16E-05	**-3.19**	73.7
1.56E-03	2.81E-05	**-2.81**	72.1
2.53E-03	4.55E-05	**-2.60**	69.2
5.03E-03	9.06E-05	**-2.30**	62.8
1.00E-02	1.80E-04	**-2.00**	55.7
2.06E-02	3.72E-04	**-1.69**	46.5
3.25E-02	5.87E-04	**-1.49**	40.4
4.13E-02	7.47E-04	**-1.38**	36.4
4.99E-02	9.02E-04	**-1.30**	33.4

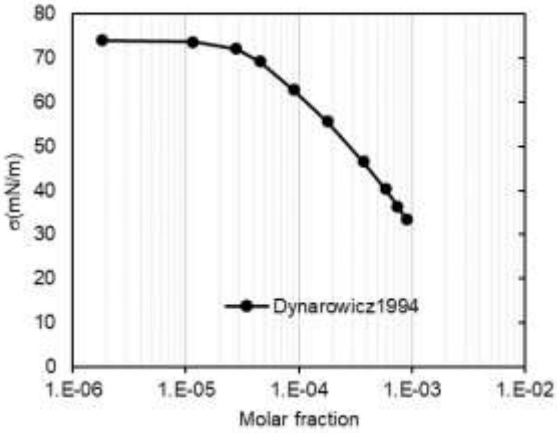

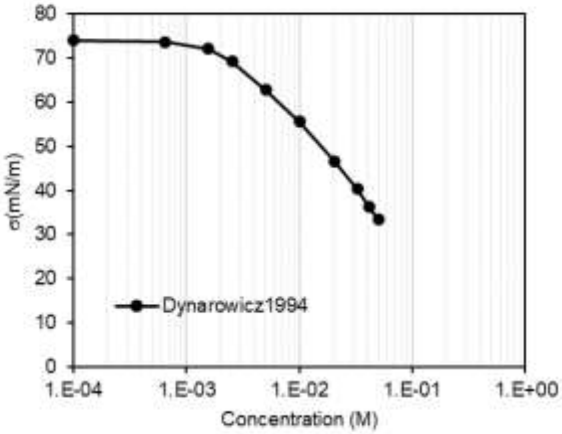

Fig. S68: Experimental surface tension data for 4-ethylphenol / water mixtures.

Comment: N/A

AK28: octan-1-ol

Hommelen 1959 **Graph (experimental points)**				Romero et al. 2009 **Table**			Le et al. 2012 **Graph (experimental points)**		
**C (M)**	**Molar fraction**	**logC(M)**	**σ (mN/m)**	**C (M)**	**Molar fraction**	**σ (mN/m)**	**C (M)**	**Molar fraction**	**σ (mN/m)**
8.13E-05	1.46E-06	**-4.09**	71.8	0.00E+00	**0.00E+00**	71.81	**1.50E-04**	2.70E-06	70.0
2.95E-04	5.31E-06	**-3.53**	62.7	1.67E-03	**3.00E-05**	43.19	**2.00E-04**	3.60E-06	69.7
7.94E-04	1.43E-05	**-3.10**	51.6	2.78E-03	**5.00E-05**	38.30	**2.50E-04**	4.50E-06	69.6
1.17E-03	2.12E-05	**-2.93**	46.2	3.33E-03	**6.00E-05**	35.54	**3.10E-04**	5.58E-06	68.8
1.48E-03	2.66E-05	**-2.83**	43.2	3.89E-03	**7.00E-05**	33.18	**4.00E-04**	7.20E-06	68.2
3.16E-03	5.69E-05	**-2.50**	31.6	5.00E-03	**9.00E-05**	32.14	**4.90E-04**	8.82E-06	66.9
3.63E-03	6.54E-05	**-2.44**	29.5	5.55E-03	**1.00E-04**	32.00	**6.20E-04**	1.12E-05	65.4
							**7.80E-04**	1.40E-05	63.6
							**9.70E-04**	1.75E-05	61.4
							**1.10E-03**	1.98E-05	59.9
							**1.20E-03**	2.16E-05	58.6
							**1.40E-03**	2.52E-05	57.1
							**1.60E-03**	2.88E-05	55.4
							**1.70E-03**	3.06E-05	54.0
							**1.90E-03**	3.42E-05	53.0
							**2.76E-03**	4.97E-05	42.3

Recommended		
C (M)	***Molar fraction***	***σ (mN/m)***
8.11E-05	*1.46E-06*	*71.7*
1.01E-04	*1.82E-06*	*71.6*
1.27E-04	*2.28E-06*	*71.4*
1.58E-04	*2.85E-06*	*71.2*
1.97E-04	*3.55E-06*	*70.8*
2.47E-04	*4.44E-06*	*70.2*
3.08E-04	*5.55E-06*	*69.4*
3.85E-04	*6.93E-06*	*68.3*
4.81E-04	*8.65E-06*	*66.8*
6.01E-04	*1.08E-05*	*64.7*
7.50E-04	*1.35E-05*	*62.1*
9.37E-04	*1.69E-05*	*58.8*
1.17E-03	*2.11E-05*	*55.0*
1.46E-03	*2.63E-05*	*50.8*
1.83E-03	*3.29E-05*	*46.5*
2.28E-03	*4.11E-05*	*42.4*
2.85E-03	*5.13E-05*	*38.8*
3.56E-03	*6.41E-05*	*35.8*
4.44E-03	*8.01E-05*	*33.5*
5.55E-03	*1.00E-04*	*31.7*

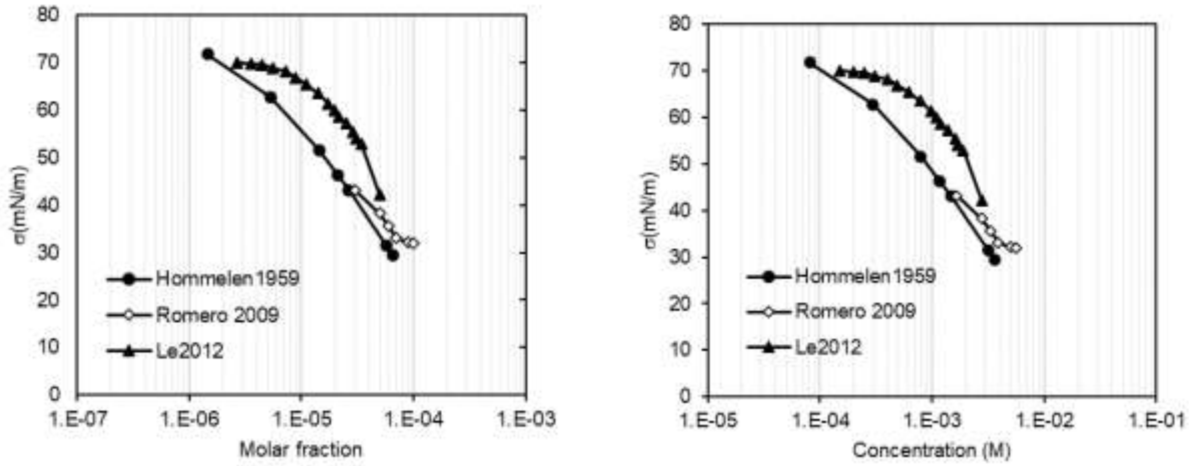

Fig. S69 (a): Comparison of the experimental surface tension data for octan-1-ol / water mixtures.

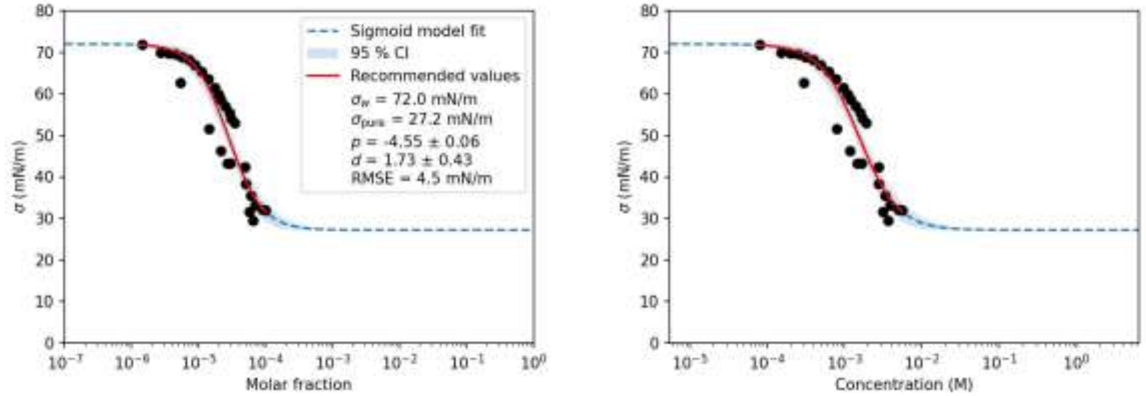

Fig. S69 (b): Surface tension fit with the Sigmoid model (*Kleinheins et al. 2023*) for octan-1-ol / water mixtures. Solid red line: model fit inside the concentration range covered by experimental data, reported as recommended values. Blue shading: fit parameters with 95 % confidence interval (CI). RMSE: root mean squared error. Markers: data used for fitting.

Comment: N/A

AK29: octan-2-ol

Le et al. 2012	**Graph (experimental points)**	
**C (M)**	**Molar fraction**	**σ (mN/m)**
**1.56E-04**	2.81E-06	70.0
**1.98E-04**	3.56E-06	69.7
**2.50E-04**	4.50E-06	69.0
**3.10E-04**	5.58E-06	68.4
**4.00E-04**	7.20E-06	67.3
**4.40E-04**	7.92E-06	66.8
**5.50E-04**	9.90E-06	65.5
**6.90E-04**	1.24E-05	64.4
**8.80E-04**	1.58E-05	62.4
**1.10E-03**	1.98E-05	60.5
**1.38E-03**	2.48E-05	58.3
**1.73E-03**	3.11E-05	55.7
**2.20E-03**	3.96E-05	52.8
**2.80E-03**	5.04E-05	49.0
**3.10E-03**	5.58E-05	47.3
**4.30E-03**	7.74E-05	40.6

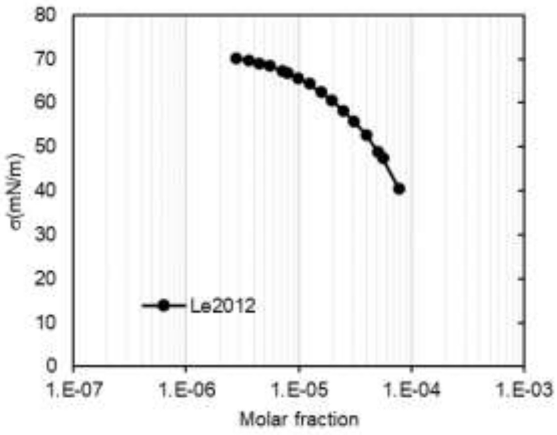

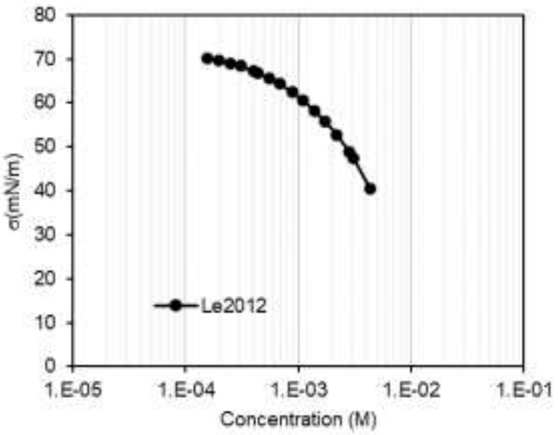

Fig. S70: Experimental surface tension data for octan-2-ol / water mixtures.

Comment: N/A

AK30: 4-propylphenol

Dynarowicz 1994	**Graph (experimental points)**		
**C (M)**	**Molar fraction**	**logC (M)**	**σ (mN/m)**
1.00E-04	1.80E-06	**-4.00**	74.0
3.88E-04	6.99E-06	**-3.41**	72.3
6.12E-04	1.10E-05	**-3.21**	70.1
9.65E-04	1.74E-05	**-3.02**	66.0
2.08E-03	3.74E-05	**-2.68**	57.5
4.10E-03	7.38E-05	**-2.39**	48.1
6.18E-03	1.11E-04	**-2.21**	42.4
1.03E-02	1.85E-04	**-1.99**	34.9

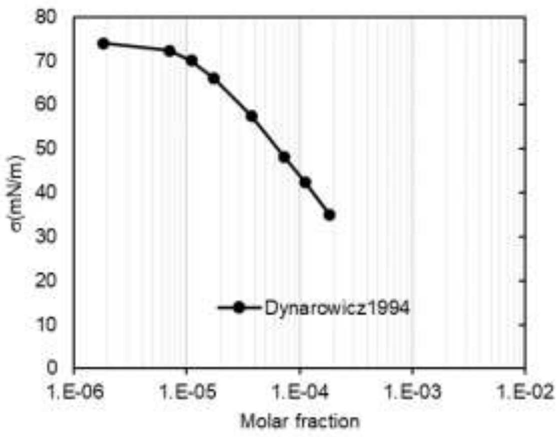

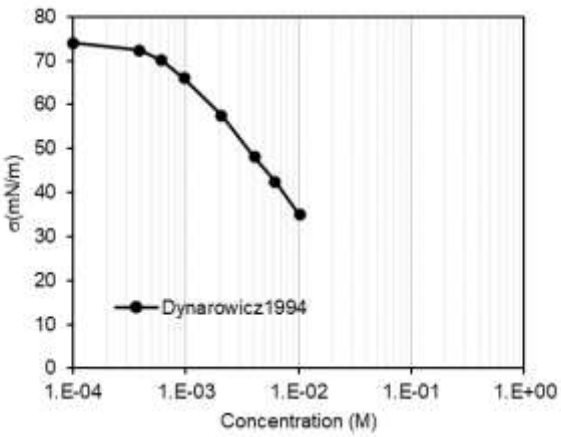

Fig. S71: Experimental surface tension data for 4-propylphenol / water mixtures.

Comment: N/A

AK31: 1-naphthol (naphthalen-1-ol)

Chakraborty et al. 2016 **Graph (experimental points)**		
**C (M)**	**Molar fraction**	**σ (mN/m)**
**2.88E-03**	5.19E-05	64.0
**3.11E-03**	5.60E-05	63.5
**3.27E-03**	5.89E-05	63.3
**3.50E-03**	6.30E-05	61.5
**3.66E-03**	6.59E-05	60.5
**3.90E-03**	7.02E-05	59.5
**4.10E-03**	7.38E-05	59.0
**4.45E-03**	8.01E-05	58.5
**4.65E-03**	8.37E-05	57.0
**4.90E-03**	8.82E-05	57.0
**5.15E-03**	9.27E-05	57.0

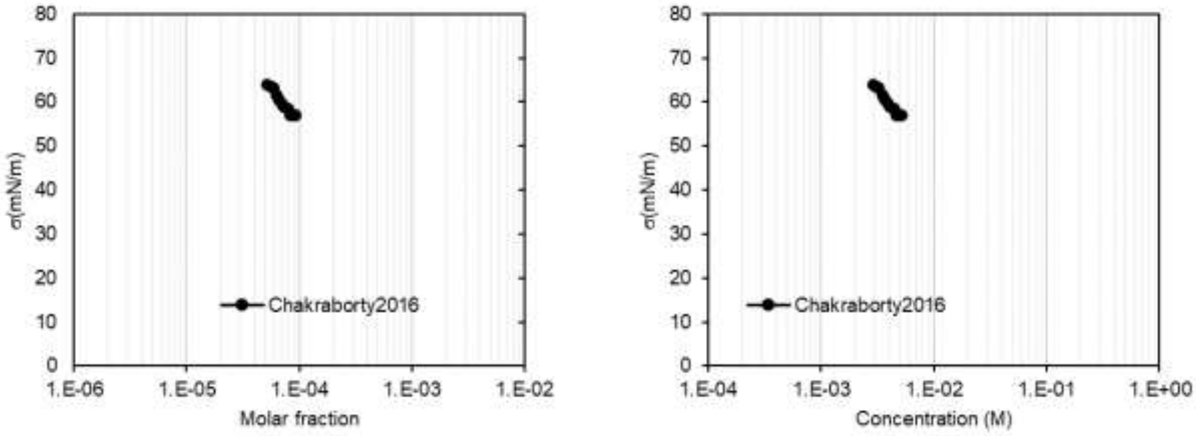

Fig. S72: Experimental surface tension data for 1-naphthol / water mixtures.

Comment:

Chakraborty et al. 2016 : data reported at 29.85°C.

AK32: 2-naphthol (naphthalen-2-ol)

Chakraborty et al. 2016 **Graph (experimental points)**		
**C (M)**	**Molar fraction**	**σ (mN/m)**
**1.88E-03**	3.38E-05	66.0
**2.16E-03**	3.89E-05	63.0
**2.33E-03**	4.19E-05	61.5
**2.66E-03**	4.79E-05	60.0
**2.80E-03**	5.04E-05	58.5
**3.11E-03**	5.60E-05	57.0
**3.44E-03**	6.19E-05	56.3
**3.66E-03**	6.59E-05	55.5
**4.00E-03**	7.20E-05	55.0
**4.44E-03**	8.00E-05	55.5
**4.88E-03**	8.79E-05	55.5
**5.22E-03**	9.40E-05	55.5

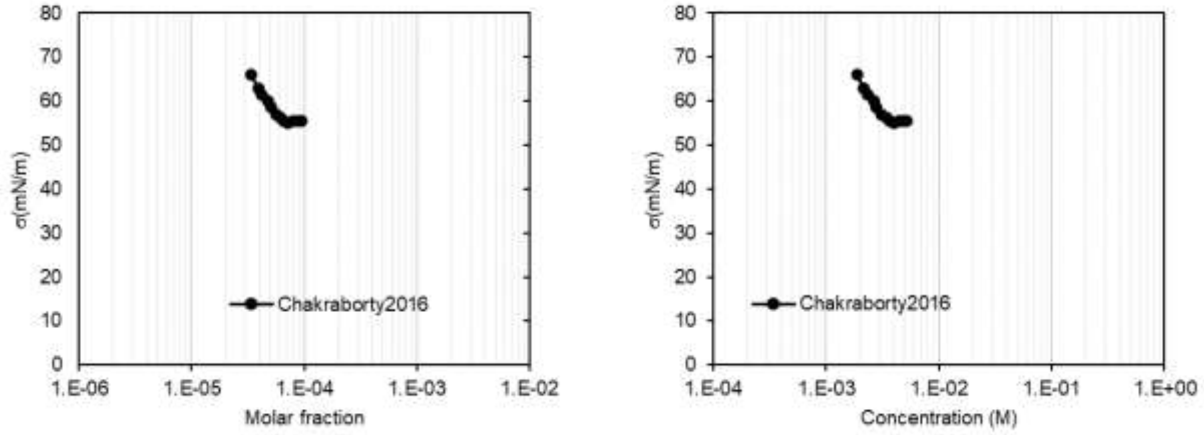

Fig. S73: Experimental surface tension data for 2-naphthol / water mixtures.

Comment:

Chakraborty et al. 2016 : data reported at 29.85°C.

AK33: nonan-1-ol

Le et al. 2012	**Graph (experimental points)**	
**C (M)**	**Molar fraction**	**σ (mN/m)**
**3.30E-05**	5.94E-07	71.0
**4.10E-05**	7.38E-07	70.8
**5.10E-05**	9.18E-07	70.7
**6.60E-05**	1.19E-06	70.5
**8.40E-05**	1.51E-06	70.5
**1.10E-04**	1.98E-06	70.0
**1.30E-04**	2.34E-06	69.7
**1.60E-04**	2.88E-06	69.2
**2.04E-04**	3.67E-06	68.2
**2.60E-04**	4.68E-06	66.3
**3.30E-04**	5.94E-06	64.7
**3.70E-04**	6.66E-06	64.0
**4.10E-04**	7.38E-06	62.9
**4.70E-04**	8.46E-06	60.8
**5.20E-04**	9.36E-06	58.6
**5.90E-04**	1.06E-05	55.9
**6.80E-04**	1.22E-05	52.5
**7.10E-04**	1.28E-05	50.0

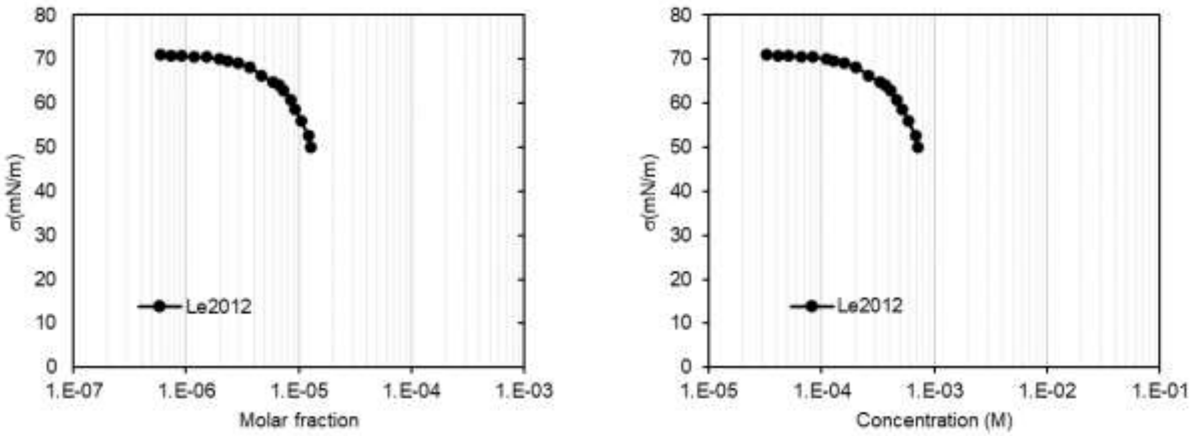

Fig. S74: Experimental surface tension data for nonan-1-ol / water mixtures.

Comment: N/A

AK34: nonan-5-ol

Le et al. 2012	**Graph (experimental points)**	
**C (M)**	**Molar fraction**	**σ (mN/m)**
**1.99E-05**	3.58E-07	71.1
**2.50E-05**	4.50E-07	71.0
**3.10E-05**	5.58E-07	71.0
**3.90E-05**	7.02E-07	70.9
**4.90E-05**	8.82E-07	70.7
**6.00E-05**	1.08E-06	70.7
**7.90E-05**	1.42E-06	70.5
**9.80E-05**	1.76E-06	70.3
**1.30E-04**	2.34E-06	70.0
**1.50E-04**	2.70E-06	69.6
**1.98E-04**	3.56E-06	68.8
**2.50E-04**	4.50E-06	68.2
**3.00E-04**	5.40E-06	67.3
**4.10E-04**	7.38E-06	65.9
**5.00E-04**	9.00E-06	64.3
**6.20E-04**	1.12E-05	62.7
**7.90E-04**	1.42E-05	60.5
**1.00E-03**	1.80E-05	58.4
**1.10E-03**	1.98E-05	57.4
**1.20E-03**	2.16E-05	56.4

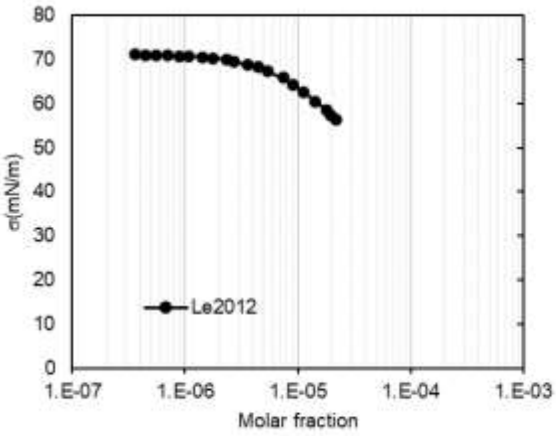

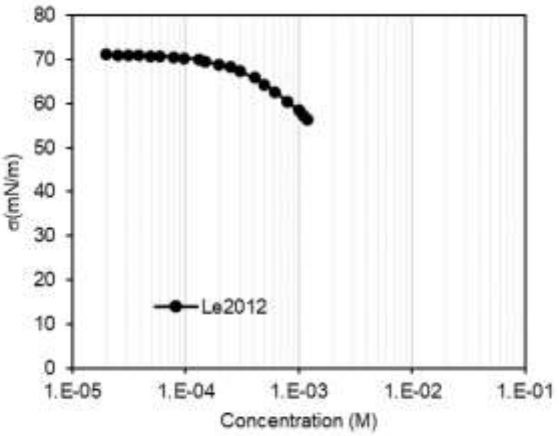

Fig. S75: Experimental surface tension data for nonan-5-ol / water mixtures.

Comment: N/A

AK35: 4-tert-butylphenol

Dynarowicz 1994	**Graph (experimental points)**		
**C (M)**	**Molar fraction**	**logC (M)**	**σ (mN/m)**
1.00E-04	1.80E-06	**-4.00**	73.7
1.99E-04	3.58E-06	**-3.70**	71.7
3.99E-04	7.18E-06	**-3.40**	67.1
6.12E-04	1.10E-05	**-3.21**	63.1
8.14E-04	1.47E-05	**-3.09**	60.0
1.00E-03	1.80E-05	**-3.00**	57.6

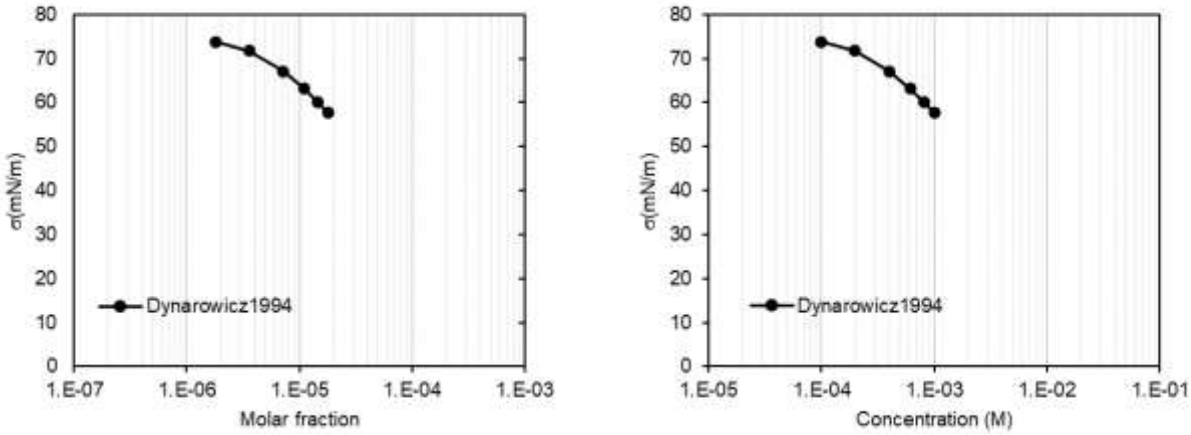

Fig. S76: Experimental surface tension data for 4-tert-butylphenol / water mixtures.

Comment: N/A

AK36: 4-sec-butylphenol

Dynarowicz 1994	**Graph (experimental points)**		
**C (M)**	**Molar fraction**	**logC (M)**	**σ (mN/m)**
1.00E-04	1.80E-06	**-4.00**	73.7
1.99E-04	3.58E-06	**-3.70**	70.8
3.99E-04	7.18E-06	**-3.40**	65.0
6.23E-04	1.12E-05	**-3.21**	60.4
8.22E-04	1.48E-05	**-3.09**	57.3
1.01E-03	1.82E-05	**-3.00**	54.5

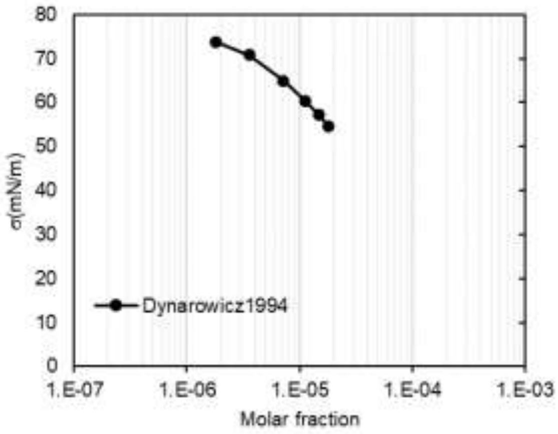

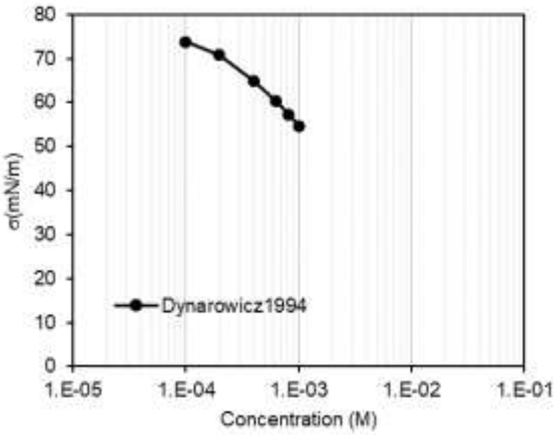

Fig. S77: Experimental surface tension data for 4-sec-butylphenol / water mixtures.

Comment: N/A

AK37: 2,3-dihydroxynaphthalene

Chakrabortyet al. 2016 **Graph (experimental points)**		
**C (M)**	**Molar fraction**	**σ (mN/m)**
**1.01E-03**	1.82E-05	59.6
**1.50E-03**	2.70E-05	58.4
**2.09E-03**	3.76E-05	56.8
**2.53E-03**	4.56E-05	55.8
**2.89E-03**	5.20E-05	54.9
**3.30E-03**	5.94E-05	53.9
**3.46E-03**	6.23E-05	53.8
**3.82E-03**	6.88E-05	53.4
**3.95E-03**	7.11E-05	53.2
**4.19E-03**	7.55E-05	52.7
**4.43E-03**	7.98E-05	52.7
**4.54E-03**	8.18E-05	52.6
**4.74E-03**	8.54E-05	52.6
**4.84E-03**	8.72E-05	52.4

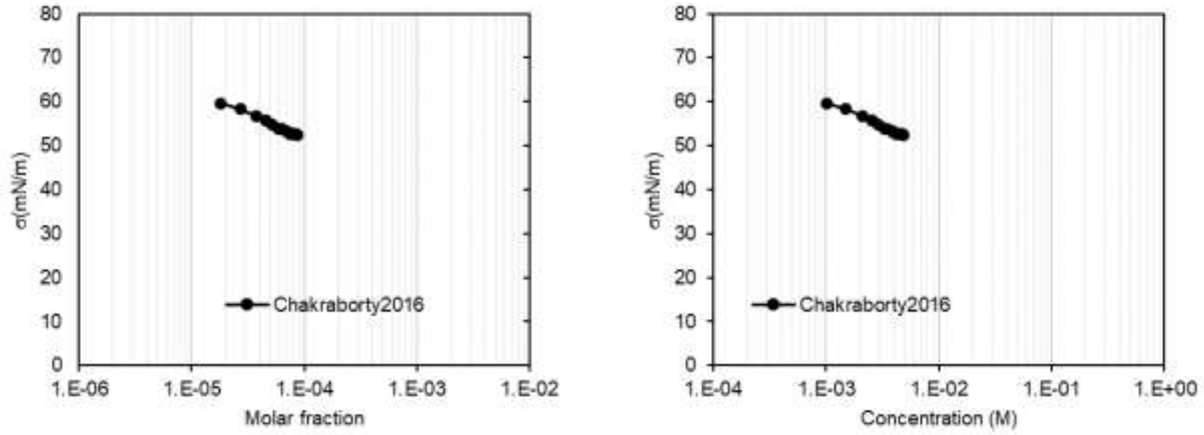

Fig. S78: Experimental surface tension data for 2,3-dihydroxynaphthalene / water mixtures.

Comment:

Chakraborty et al. 2016 : data reported at 29.85°C.

S3.3 Amines. sugars

SA1: colamine (2-aminoethan-1-ol)

Vázquez et al. 1997 **Table**			Idris et al. 2017 **Table**				***Recommended***		
**C (M)**	**Molar fraction**	**σ (mN/m)**	**C (M)**	**Molar fraction**	**Mass fraction**	**σ (mN/m)**	***C (M)***	***Molar fraction***	***σ (mN/m)***
0.00E+00	**0.00E+00**	72.01	4.94E+00	1.12E-01	**3.00E-01**	59.61	*8.05E-01*	*1.50E-02*	*67.4*
8.05E-01	**1.50E-02**	68.45	6.60E+00	1.64E-01	**4.00E-01**	57.94	*9.96E-01*	*1.87E-02*	*66.9*
1.65E+00	**3.20E-02**	65.97	8.26E+00	2.28E-01	**5.00E-01**	56.52	*1.23E+00*	*2.33E-02*	*66.3*
2.44E+00	**4.90E-02**	64.09	9.93E+00	3.07E-01	**6.00E-01**	55.20	*1.51E+00*	*2.91E-02*	*65.7*
3.30E+00	**6.90E-02**	62.63	1.33E+01	5.41E-01	**8.00E-01**	53.40	*1.86E+00*	*3.63E-02*	*65.0*
4.93E+00	**1.12E-01**	60.41	1.50E+01	7.26E-01	**9.00E-01**	50.60	*2.28E+00*	*4.53E-02*	*64.3*
6.59E+00	**1.64E-01**	58.74	1.67E+01	1.00E+00	**1.00E+00**	48.10	*2.77E+00*	*5.65E-02*	*63.5*
8.27E+00	**2.28E-01**	57.31					*3.36E+00*	*7.05E-02*	*62.7*
9.94E+00	**3.07E-01**	55.99					*4.05E+00*	*8.79E-02*	*61.8*
1.16E+01	**4.07E-01**	54.66					*4.85E+00*	*1.10E-01*	*60.8*
1.33E+01	**5.41E-01**	53.18					*5.76E+00*	*1.37E-01*	*59.8*
1.50E+01	**7.26E-01**	51.38					*6.78E+00*	*1.71E-01*	*58.7*
1.67E+01	**1.00E+00**	48.95					*7.90E+00*	*2.13E-01*	*57.6*
							9.11E+00	*2.65E-01*	*56.4*
							1.04E+01	*3.31E-01*	*55.1*
							1.17E+01	*4.13E-01*	*53.8*
							1.30E+01	*5.15E-01*	*52.5*
							1.43E+01	*6.43E-01*	*51.2*
							1.55E+01	*8.02E-01*	*49.8*
							1.67E+01	*1.00E+00*	*48.5*

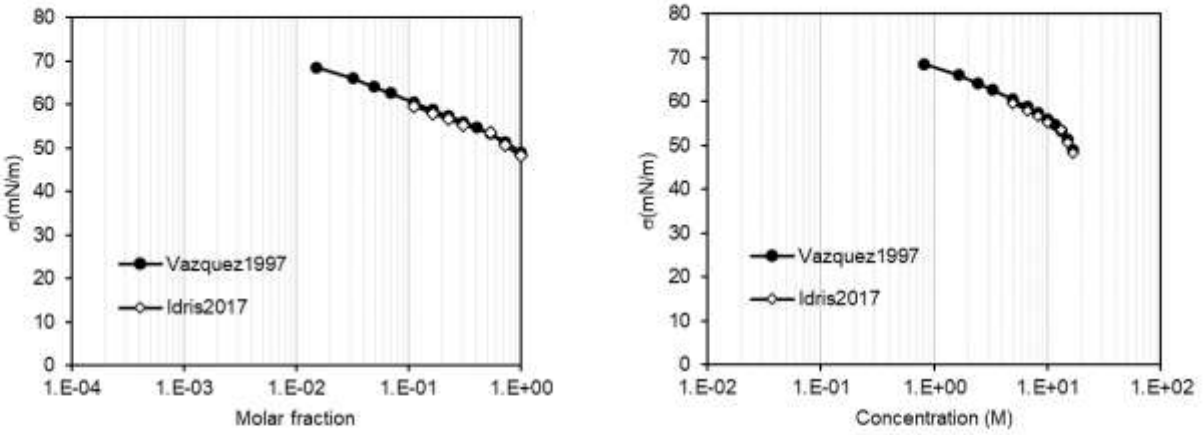

Fig. S79 (a): Comparison of the experimental data for colamine / water mixtures.

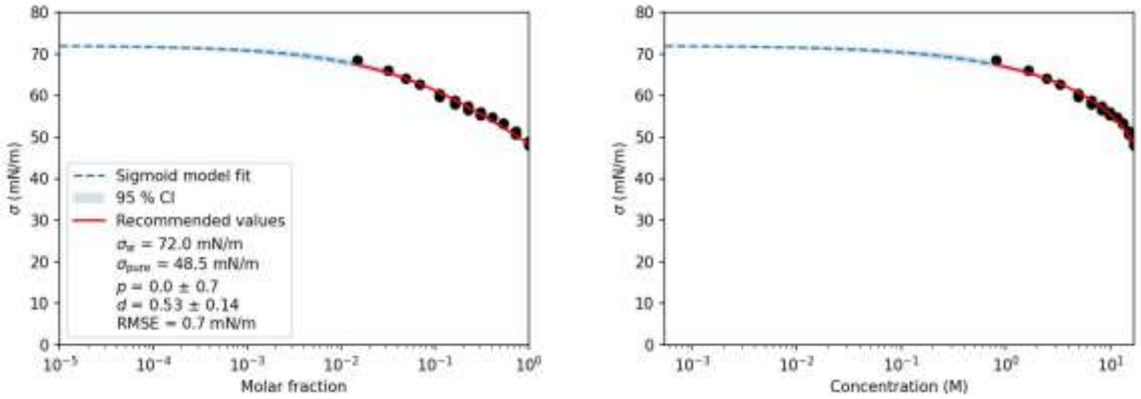

Fig. S79 (b): Surface tension fit with the Sigmoid model (*Kleinheins et al. 2023*) for colamine / water mixtures. Solid red line: model fit inside the concentration range covered by experimental data, reported as recommended values. Blue shading: fit parameters with 95 % confidence interval (CI). RMSE: root mean squared error. Markers: data used for fitting.

Comment:

Idris et al. 2017 : data reported at 30°C.

SA2: pyrrolidine (prolamine)

Gomez-Dıaz et al. 2004	**Table**	
**C (M)**	**Molar fraction**	**σ (mN/m)**
0.00E+00	**0.00E+00**	72.05
8.21E-01	**1.56E-02**	55.32
2.60E+00	**5.64E-02**	45.61
4.33E+00	**1.08E-01**	41.19
6.05E+00	**1.79E-01**	39.74
8.06E+00	**3.04E-01**	37.09
9.30E+00	**4.21E-01**	35.45
1.01E+01	**5.22E-01**	34.40
1.06E+01	**6.09E-01**	33.54
1.10E+01	**6.86E-01**	32.59
1.14E+01	**7.84E-01**	31.45
1.18E+01	**9.16E-01**	30.42
1.21E+01	**1.00E+00**	29.75
0.00E+00	**0.00E+00**	72.05
8.21E-01	**1.56E-02**	55.32
2.60E+00	**5.64E-02**	45.61
4.33E+00	**1.08E-01**	41.19
6.05E+00	**1.79E-01**	39.74
8.06E+00	**3.04E-01**	37.09

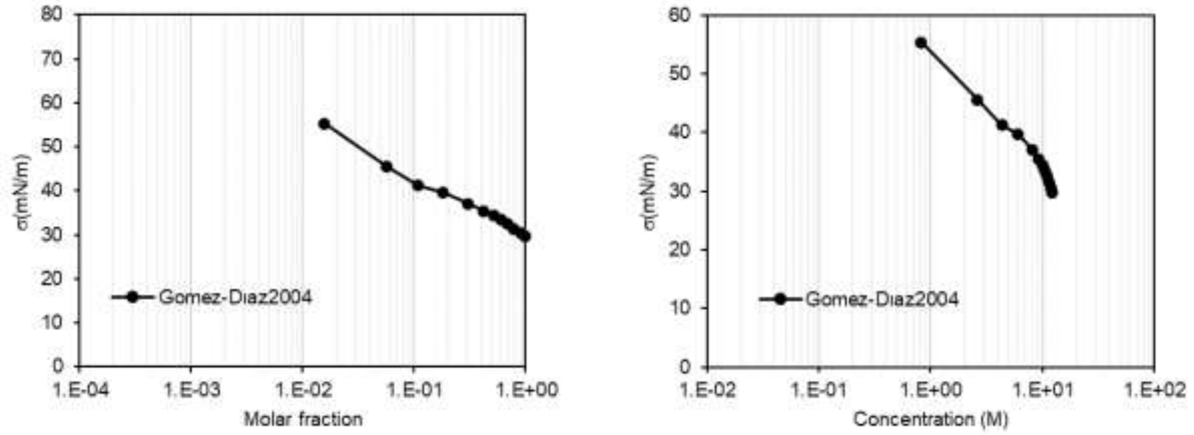

Fig. S80: Experimental data for pyrrolidine / water mixtures.

Comment: N/A

SA3: glycine (aminoacetic acid)

Chandra et al. 2013 **Table**			Rodríguez et al. 2017 **Table**			***Recommended***		
**C (M)**	**Molar fraction**	**σ (mN/m)**	**C (M)**	**Molar fraction**	**σ (mN/m)**	***C (M)***	***Molar fraction***	***σ (mN/m)***
**0.00E+00**	0.00E+00	71.97	0.00E+00	**0.00E+00**	71.73	*9.72E-03*	*1.75E-04*	*72.0*
**5.02E-02**	9.06E-04	72.07	9.72E-03	**1.75E-04**	71.75	*1.12E-02*	*2.02E-04*	*72.0*
**7.55E-02**	1.36E-03	72.14	1.94E-02	**3.49E-04**	71.79	*1.30E-02*	*2.34E-04*	*72.0*
**1.01E-01**	1.82E-03	72.03	3.87E-02	**6.98E-04**	71.86	*1.50E-02*	*2.70E-04*	*72.0*
**1.26E-01**	2.28E-03	71.88	5.78E-02	**1.04E-03**	71.92	*1.73E-02*	*3.12E-04*	*72.0*
**1.51E-01**	2.74E-03	71.79	7.72E-02	**1.39E-03**	71.99	*2.00E-02*	*3.61E-04*	*72.0*
			9.62E-02	**1.74E-03**	72.05	*2.32E-02*	*4.17E-04*	*72.0*
			1.15E-01	**2.09E-03**	72.11	*2.68E-02*	*4.82E-04*	*72.0*
			1.46E-01	**2.64E-03**	72.20	*3.09E-02*	*5.57E-04*	*72.0*
						3.57E-02	*6.44E-04*	*72.0*
						4.13E-02	*7.44E-04*	*72.0*
						4.77E-02	*8.60E-04*	*72.0*
						5.51E-02	*9.94E-04*	*72.0*
						6.37E-02	*1.15E-03*	*72.0*
						7.36E-02	*1.33E-03*	*72.0*
						8.50E-02	*1.54E-03*	*72.0*
						9.81E-02	*1.77E-03*	*72.0*
						1.13E-01	*2.05E-03*	*72.0*
						1.31E-01	*2.37E-03*	*72.0*
						1.51E-01	*2.74E-03*	*72.0*

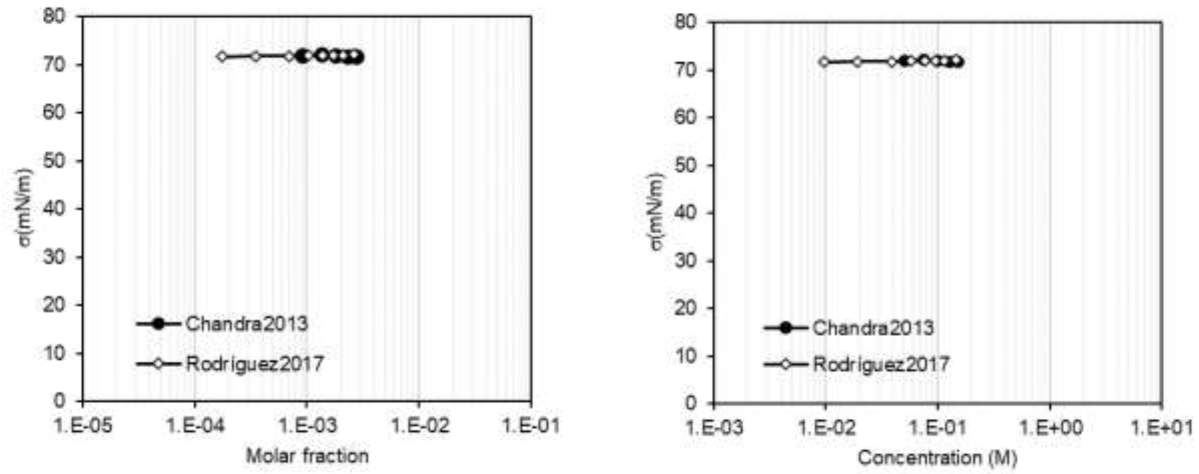

Fig. S81 (a): Comparison of the experimental data for glycine / water mixtures.

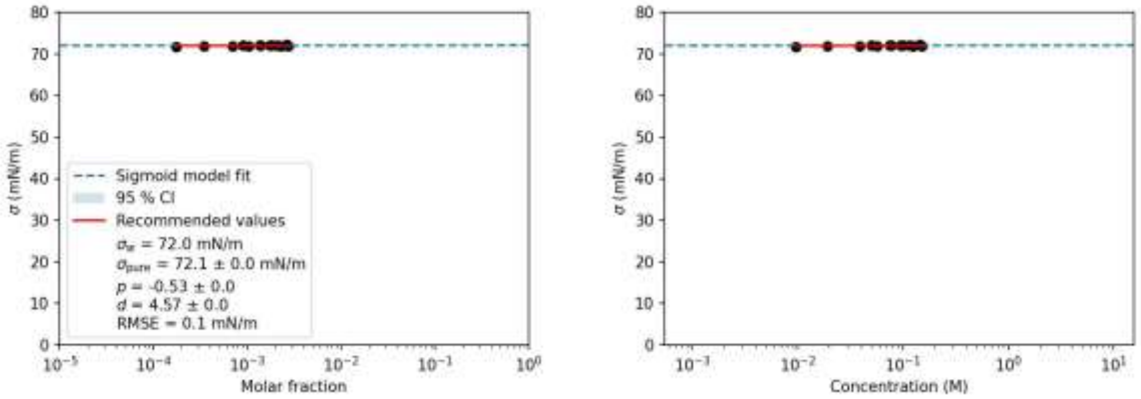

Fig. S81 (b): Surface tension fit with the Sigmoid model (*Kleinheins et al. 2023*) for glycine / water mixtures. Solid red line: model fit inside the concentration range covered by experimental data, reported as recommended values. Blue shading: fit parameters with 95 % confidence interval (CI). RMSE: root mean squared error. Markers: data used for fitting.

Comment: N/A

SA4: threamine (1-aminopropan-2-ol)

Alvarez et al. 2003 **Table**		
**C (M)**	**Molar fraction**	**σ (mN/m)**
0.00E+00	**0.00E+00**	72.01
6.67E-01	**1.25E-02**	66.34
1.33E+00	**2.59E-02**	62.20
2.65E+00	**5.65E-02**	56.95
3.96E+00	**9.31E-02**	53.59
5.27E+00	**1.38E-01**	51.07
6.57E+00	**1.93E-01**	49.00
7.86E+00	**2.64E-01**	47.18
9.14E+00	**3.59E-01**	45.40
1.04E+01	**4.89E-01**	43.43
1.17E+01	**6.83E-01**	40.97
1.30E+01	**1.00E+00**	37.38

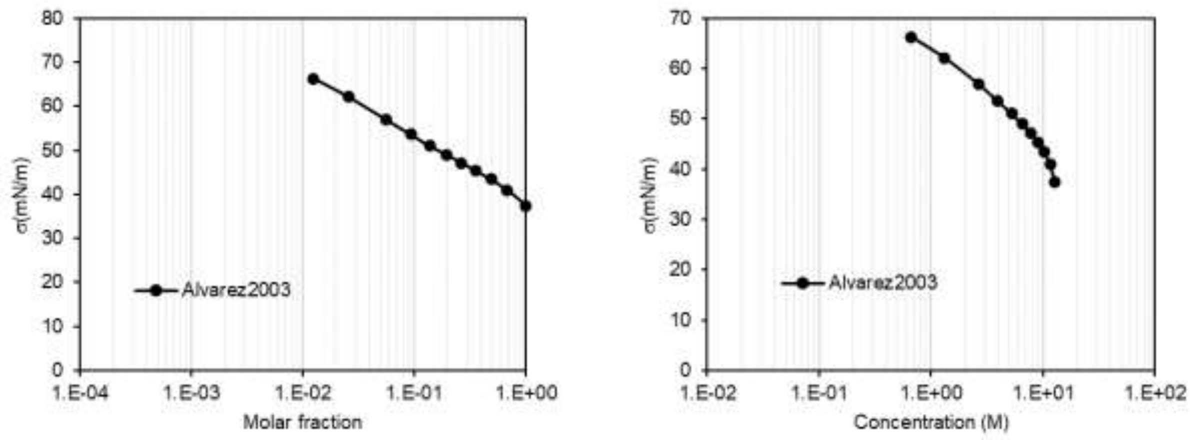

Fig. S82: Experimental data for threamine / water mixtures.

Comment: N/A

SA5: 3-aminopropan-1-ol

Alvarez et al. 2003 **Table**		
**C (M)**	**Molar fraction**	**σ (mN/m)**
0.00E+00	**0.00E+00**	72.01
6.67E-01	**1.25E-02**	69.62
1.33E+00	**2.59E-02**	67.45
2.65E+00	**5.65E-02**	63.69
3.97E+00	**9.31E-02**	60.50
5.29E+00	**1.38E-01**	57.76
6.60E+00	**1.93E-01**	55.29
7.90E+00	**2.64E-01**	53.08
9.20E+00	**3.59E-01**	51.01
1.05E+01	**4.89E-01**	48.89
1.18E+01	**6.83E-01**	46.65
1.31E+01	**1.00E+00**	43.90

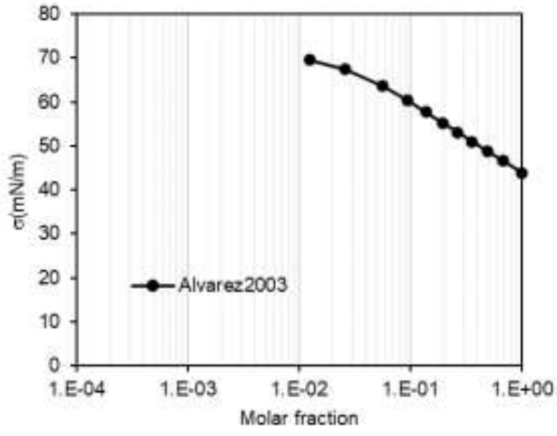

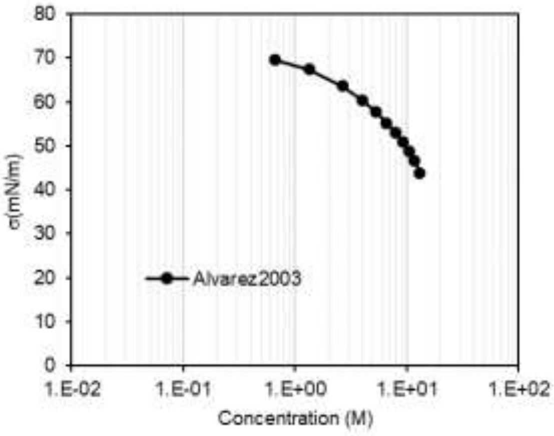

Fig. S83: Experimental data for 3-aminopropan-1-ol / water mixtures.

Comment: N/A

SA6: 2-(methylamino)ethan-1-ol

Álvarez et al. 2008 **Table**			Vankat et al. 2010 **Graph (experimental points)**			***Recommended***		
**C (M)**	**Molar fraction**	**σ (mN/m)**	**C (M)**	**Molar fraction**	**σ (mN/m)**	***C (M)***	***Molar fraction***	***σ (mN/m)***
0.00E+00	**0.00E+00**	72.01	1.37E+00	**2.70E-02**	61.0	*6.66E-01*	*1.25E-02*	*65.7*
6.66E-01	**1.25E-02**	67.18	2.72E+00	**5.90E-02**	56.0	*8.29E-01*	*1.57E-02*	*64.8*
1.33E+00	**2.60E-02**	63.36	4.13E+00	**1.00E-01**	52.5	*1.03E+00*	*1.98E-02*	*63.7*
2.71E+00	**5.87E-02**	57.87	5.24E+00	**1.40E-01**	49.0	*1.28E+00*	*2.50E-02*	*62.6*
3.91E+00	**9.31E-02**	54.16				*1.58E+00*	*3.14E-02*	*61.3*
5.19E+00	**1.38E-01**	51.14				*1.94E+00*	*3.96E-02*	*59.9*
6.44E+00	**1.93E-01**	48.42				*2.36E+00*	*4.99E-02*	*58.4*
7.68E+00	**2.64E-01**	45.57				*2.87E+00*	*6.28E-02*	*56.8*
8.90E+00	**3.59E-01**	43.23				*3.45E+00*	*7.91E-02*	*55.1*
9.98E+00	**4.73E-01**	41.13				*4.12E+00*	*9.96E-02*	*53.3*
1.13E+01	**6.77E-01**	38.46				*4.86E+00*	*1.25E-01*	*51.5*
1.19E+01	**8.17E-01**	37.01				*5.68E+00*	*1.58E-01*	*49.5*
1.22E+01	**9.21E-01**	35.96				*6.55E+00*	*1.99E-01*	*47.6*
1.25E+01	**1.00E+00**	35.28				*7.46E+00*	*2.51E-01*	*45.7*
						8.39E+00	*3.16E-01*	*43.8*
						9.31E+00	*3.98E-01*	*41.9*
						1.02E+01	*5.01E-01*	*40.1*
						1.10E+01	*6.30E-01*	*38.4*
						1.18E+01	*7.94E-01*	*36.8*
						1.25E+01	*1.00E+00*	*35.3*

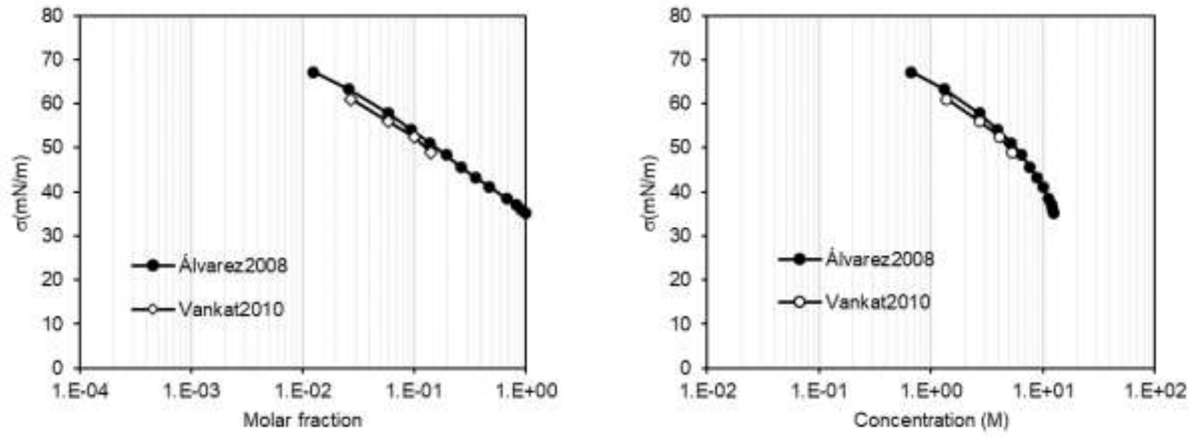

Fig. S84 (a): Comparison of the experimental data for 2-(methylamino)ethan-1-ol / water mixtures.

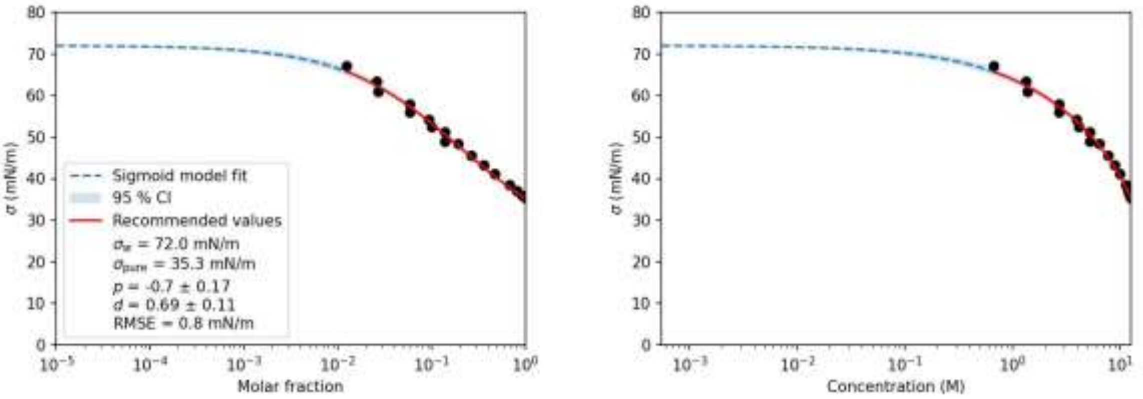

Fig. S84 (b): Surface tension fit with the Sigmoid model (*Kleinheins et al. 2023*) for 2-(methylamino)ethan-1-ol / water mixtures. Solid red line: model fit inside the concentration range covered by experimental data, reported as recommended values. Blue shading: fit parameters with 95 % confidence interval (CI). RMSE: root mean squared error. Markers: data used for fitting.

Comment: N/A

SA7: piperidine (pyridine)

Gomez-Dıaz et al. 2004 **Table**		
**C (M)**	**Molar fraction**	**σ (mN/m)**
0.00E+00	**0.00E+00**	72.05
4.08E-01	**7.60E-03**	56.85
6.82E-01	**1.30E-02**	43.58
1.15E+00	**2.28E-02**	38.27
2.11E+00	**4.58E-02**	37.20
3.62E+00	**9.20E-02**	34.99
5.06E+00	**1.54E-01**	34.02
6.74E+00	**2.67E-01**	33.90
7.78E+00	**3.78E-01**	32.75
8.87E+00	**5.66E-01**	31.15
9.54E+00	**7.52E-01**	30.42
1.01E+01	**1.00E+00**	29.56

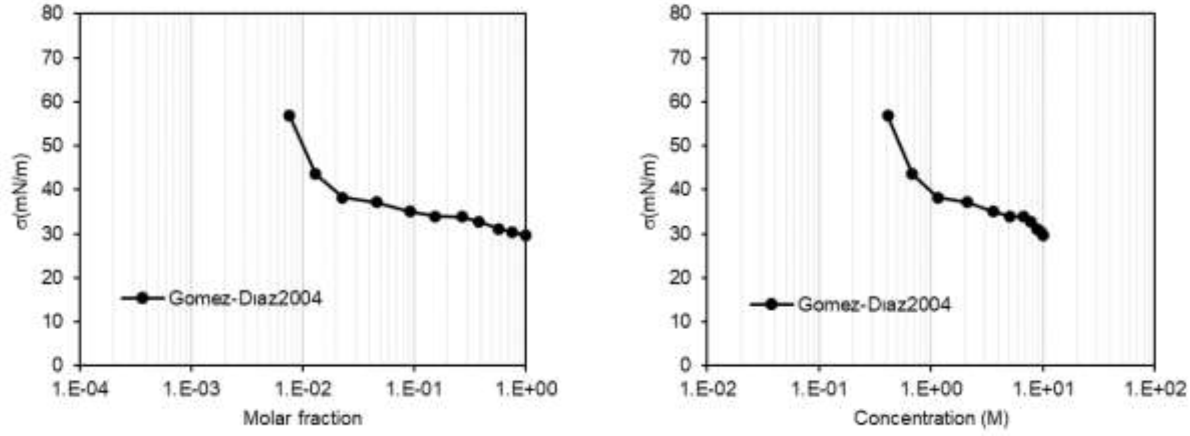

Fig. S85: Experimental data for piperidine / water mixtures.

Comment: N/A

SA8: N.N'-dimethylethane-1,2-diamine

Blanco et al. 2016 **Table**		
**C (M)**	**Molar fraction**	**σ (mN/m)**
0.00E+00	**0.00E+00**	72.6
3.73E+00	**1.00E-01**	42.6
5.60E+00	**2.00E-01**	37.1
6.74E+00	**3.00E-01**	32.9
7.49E+00	**4.00E-01**	30.6
8.05E+00	**5.04E-01**	29.4
8.44E+00	**6.00E-01**	28.6
8.76E+00	**7.01E-01**	27.9
9.01E+00	**8.00E-01**	27.4
9.22E+00	**9.00E-01**	26.9
9.39E+00	**1.00E+00**	26.4

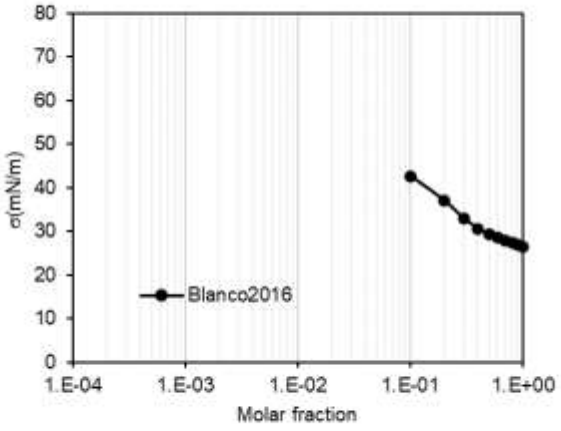

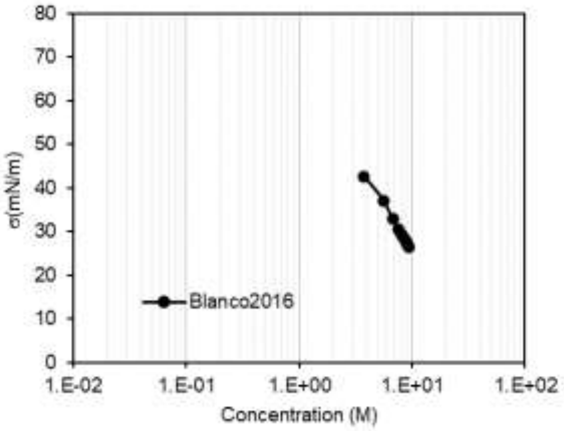

Fig. S86: Experimental data for N.N'-dimethylethane-1,2-diamine / water mixtures.

Comment: N/A

SA9: DL-alanine (2-aminopropanoic acid)

Chandra et al. 2013 **Table**			Rodríguez et al. 2017 **Table**			***Recommended***		
**C (M)**	**Molar fraction**	**σ (mN/m)**	**C (M)**	**Molar fraction**	**σ (mN/m)**	***C (M)***	***Molar fraction***	***σ (mN/m)***
0.00E+00	**0.00E+00**	71.97	0.00E+00	**0.00E+00**	71.73	*9.77E-03*	*1.76E-04*	*72.0*
2.49E+00	**5.03E-02**	72.07	9.77E-03	**1.76E-04**	71.83	*1.39E-02*	*2.51E-04*	*72.0*
3.54E+00	**7.55E-02**	71.12	1.96E-02	**3.53E-04**	71.86	*1.99E-02*	*3.59E-04*	*72.0*
4.49E+00	**1.01E-01**	72.00	3.91E-02	**7.05E-04**	71.90	*2.84E-02*	*5.12E-04*	*72.0*
5.36E+00	**1.26E-01**	71.88	5.85E-02	**1.06E-03**	71.95	*4.05E-02*	*7.31E-04*	*72.0*
6.14E+00	**1.52E-01**	71.76	7.78E-02	**1.41E-03**	72.00	*5.78E-02*	*1.04E-03*	*72.0*
			9.69E-02	**1.75E-03**	72.07	*8.24E-02*	*1.49E-03*	*72.0*
			1.16E-01	**2.11E-03**	72.10	*1.17E-01*	*2.12E-03*	*72.0*
			1.46E-01	**2.64E-03**	72.18	*1.67E-01*	*3.03E-03*	*72.0*
						2.38E-01	*4.33E-03*	*72.0*
						3.38E-01	*6.18E-03*	*72.0*
						4.80E-01	*8.82E-03*	*72.0*
						6.78E-01	*1.26E-02*	*72.0*
						9.56E-01	*1.80E-02*	*72.0*
						1.34E+00	*2.57E-02*	*72.0*
						1.87E+00	*3.66E-02*	*72.0*
						2.57E+00	*5.23E-02*	*71.9*
						3.50E+00	*7.46E-02*	*71.7*
						4.69E+00	*1.06E-01*	*71.7*
						6.14E+00	*1.52E-01*	*71.7*

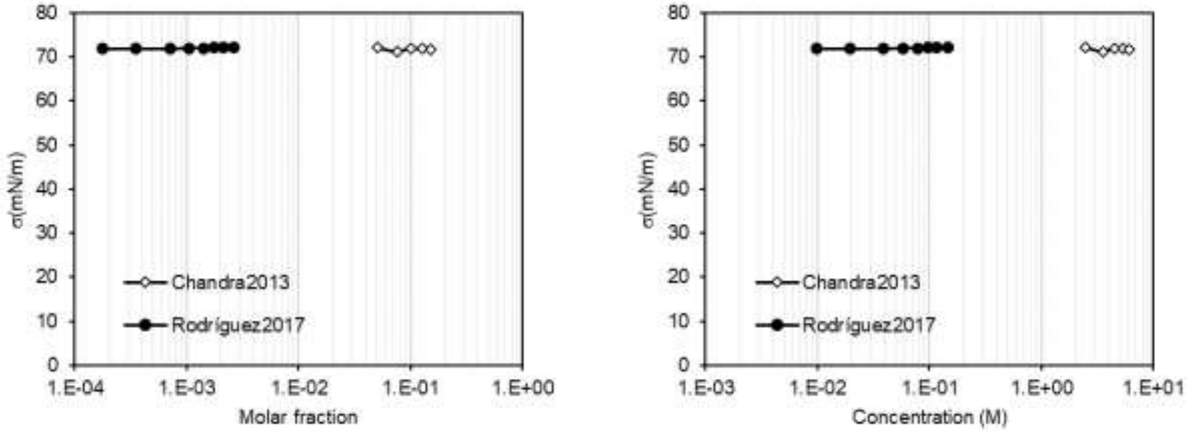

Fig. S87 (a): Experimental data for DL-alanine / water mixtures.

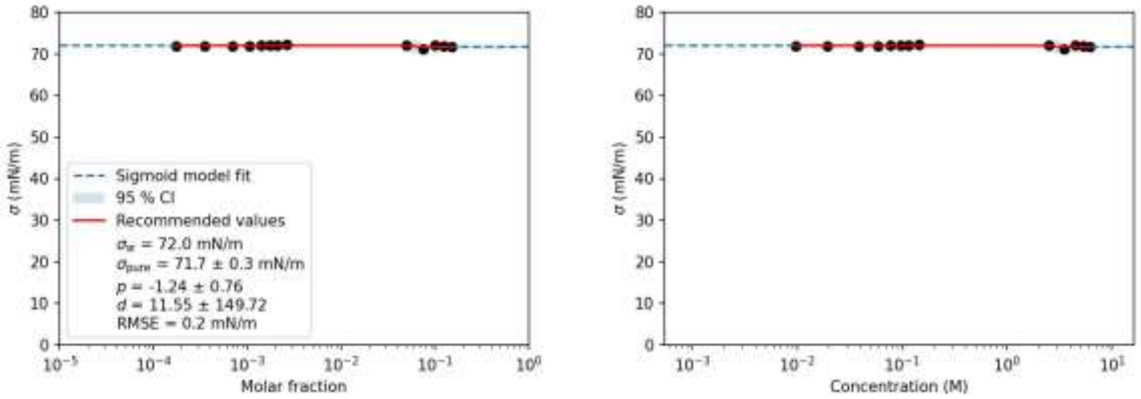

Fig. S87 (b): Surface tension fit with the Sigmoid model (*Kleinheins et al. 2023*) for DL-alanine / water mixtures. Solid red line: model fit inside the concentration range covered by experimental data, reported as recommended values. Blue shading: fit parameters with 95 % confidence interval (CI). RMSE: root mean squared error. Markers: data used for fitting.

Comment

Rodríguez et al. 2017: DL-Alanine
Chandra et al. 2013 : L-Alanine

SA10: β-alanine (3-aminopropanoic acid)

Cadena et al. 2016 **Table**		
**C (M)**	**Molar fraction**	**σ (mN/m)**
0.00E+00	**0.00E+00**	72.03
1.06E-02	**1.90E-04**	72.09
4.66E-02	**8.40E-04**	72.19
6.81E-02	**1.23E-03**	72.30
1.00E-01	**1.81E-03**	72.32
2.08E-01	**3.77E-03**	72.50
3.97E-01	**7.28E-03**	72.70
6.95E-01	**1.29E-02**	73.18
9.30E-01	**1.75E-02**	73.39

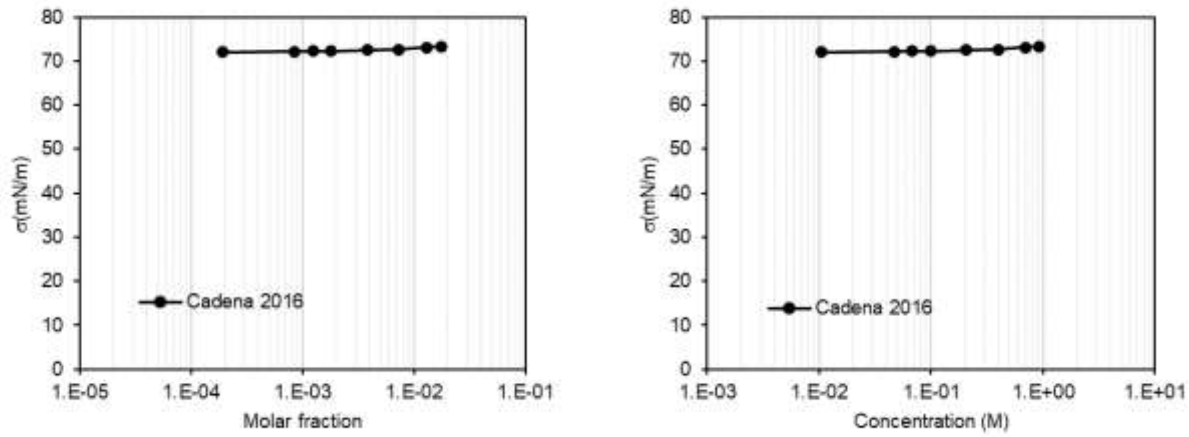

Fig. S88: Experimental data for β-alanine / water mixtures.

Comment: N/A

SA11: 2-(ethylamino)ethan-1-ol

Álvarez et al. 2008 **Table**		
**C (M)**	**Molar fraction**	**σ (mN/m)**
0.00E+00	**0.00E+00**	72.01
5.52E-01	**1.04E-02**	59.57
1.11E+00	**2.20E-02**	53.58
2.21E+00	**4.82E-02**	47.89
3.27E+00	**7.95E-02**	44.95
4.33E+00	**1.19E-01**	42.97
5.36E+00	**1.68E-01**	41.51
6.33E+00	**2.29E-01**	40.34
7.23E+00	**3.06E-01**	39.19
8.21E+00	**4.25E-01**	37.75
9.32E+00	**6.48E-01**	35.58
9.78E+00	**7.93E-01**	34.13
1.00E+01	**8.85E-01**	33.23
1.03E+01	**1.00E+00**	32.21

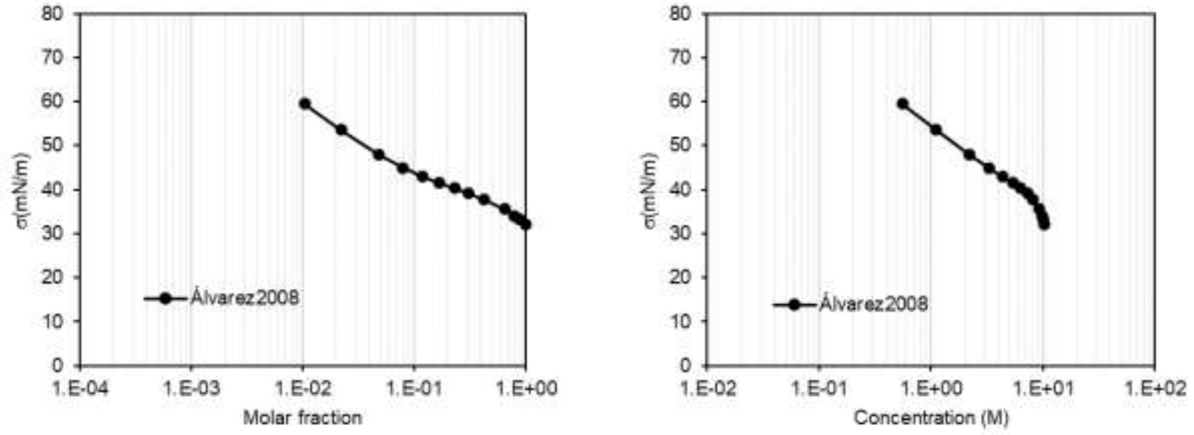

Fig. S89: Experimental data for 2-(ethylamino)ethan-1-ol / water mixtures.

Comment: N/A

SA12: 2-amino-2-methylpropan-1-ol

Vázquez et al. 1997 **Table**		
**C (M)**	**Molar fraction**	**σ (mN/m)**
0.00E+00	**0.00E+00**	72.01
5.83E-01	**1.10E-02**	59.74
1.12E+00	**2.20E-02**	53.44
1.65E+00	**3.40E-02**	49.53
2.21E+00	**4.80E-02**	46.87
2.75E+00	**6.30E-02**	44.95
3.31E+00	**8.00E-02**	43.41
4.37E+00	**1.19E-01**	41.22
5.42E+00	**1.68E-01**	39.58
6.45E+00	**2.32E-01**	38.25
7.48E+00	**3.20E-01**	37.00
8.50E+00	**4.47E-01**	35.63
9.49E+00	**6.45E-01**	33.94
1.05E+01	**1.00E+00**	31.37

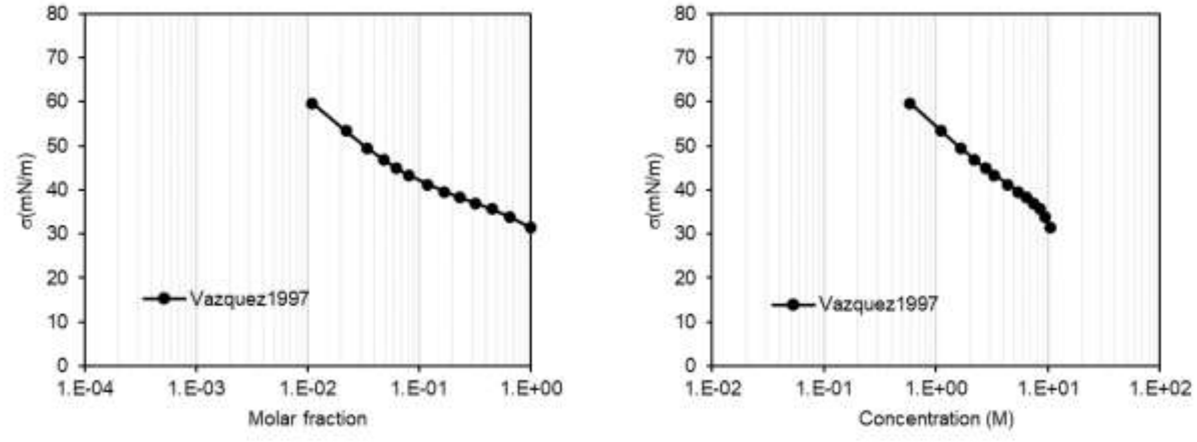

Fig. S90: Experimental data for 2-amino-2-methylpropan-1-ol / water mixtures.

Comment: N/A

SA13: 2-(dimethylamino)ethan-1-ol

Maham et al. 2001	**Table**	
**C (M)**	**Molar fraction**	**σ (mN/m)**
0.00E+00	**0.00E+00**	72.0
1.17E+00	**2.34E-02**	53.2
2.24E+00	**4.94E-02**	47.8
3.11E+00	**7.54E-02**	44.7
3.79E+00	**9.92E-02**	42.4
5.85E+00	**2.03E-01**	37.8
7.01E+00	**2.99E-01**	35.7
7.80E+00	**3.94E-01**	34.5
8.38E+00	**4.89E-01**	33.4
8.87E+00	**5.97E-01**	32.7
9.54E+00	**8.08E-01**	32.0
9.95E+00	**1.00E+00**	31.5

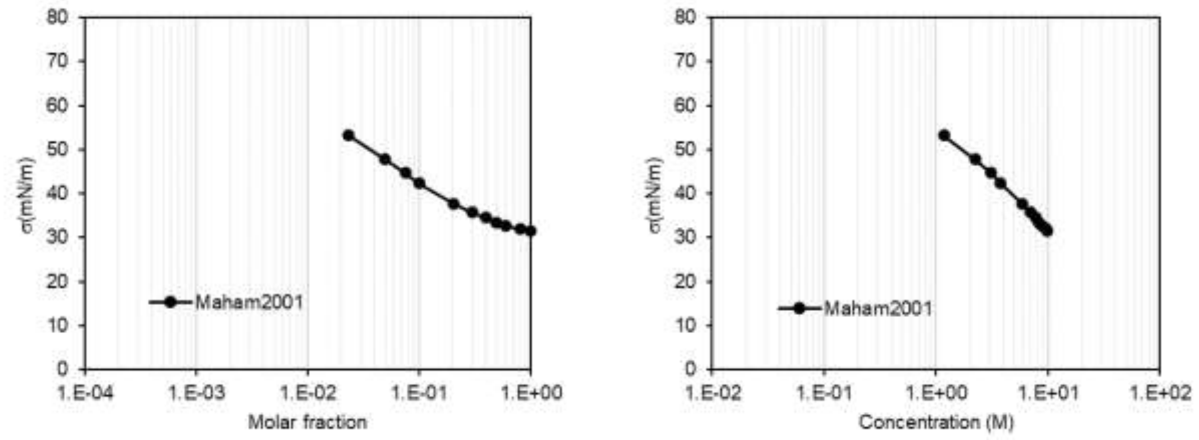

Fig. S91: Experimental data for 2-(dimethylamino)ethan-1-ol / water mixtures.

Comment: N/A

SA14: cyclohexanamine

Yokoyama et al. 1995 **Graph (experimental points)**			
**C (M)**	**Molar fraction**	**logC (M)**	**σ (mN/m)**
1.00E-03	1.80E-05	**-3.0**	70.5
3.98E-03	7.17E-05	**-2.4**	68.1
1.00E-02	1.80E-04	**-2.0**	65.2
2.00E-02	3.60E-04	**-1.7**	61.6
3.98E-02	7.20E-04	**-1.4**	57.4
6.31E-02	1.14E-03	**-1.2**	54.2
1.00E-01	1.82E-03	**-1.0**	48.7
1.58E-01	2.90E-03	**-0.8**	45.2
2.00E-01	3.67E-03	**-0.7**	42.0
3.16E-01	5.88E-03	**-0.5**	37.9
3.98E-01	7.47E-03	**-0.4**	34.8
5.01E-01	9.51E-03	**-0.3**	33.5
6.31E-01	1.21E-02	**-0.2**	32.9
7.94E-01	1.56E-02	**-0.1**	32.8
8.13E-01	1.60E-02	**-0.09**	32.5
1.00E+00	2.01E-02	**0.0**	32.4
2.00E+00	4.52E-02	**0.3**	32.4

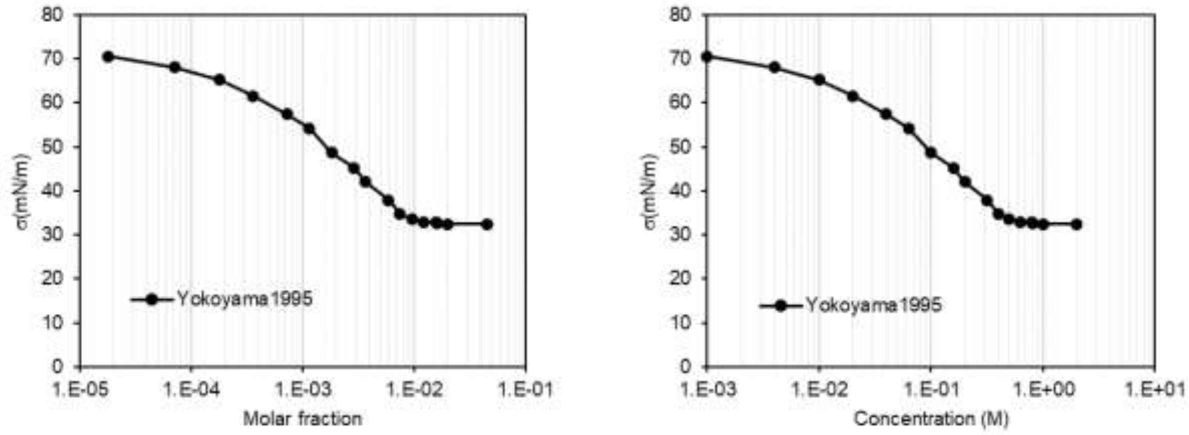

Fig. S92: Experimental data for cyclohexanamine / water mixtures.

Comment: N/A

SA15: piperidic acid (4-aminobutanoic acid)

Cadena et al. 2016 **Table**		
**C (M)**	**Molar fraction**	**σ (mN/m)**
0.00E+00	**0.00E+00**	72.03
1.11E-02	**2.00E-04**	72.09
3.60E-02	**6.50E-04**	72.18
6.25E-02	**1.13E-03**	72.22
9.60E-02	**1.74E-03**	72.27
2.47E-01	**4.53E-03**	72.43
3.70E-01	**6.85E-03**	72.54
6.63E-01	**1.26E-02**	72.91
8.11E-01	**1.56E-02**	73.13

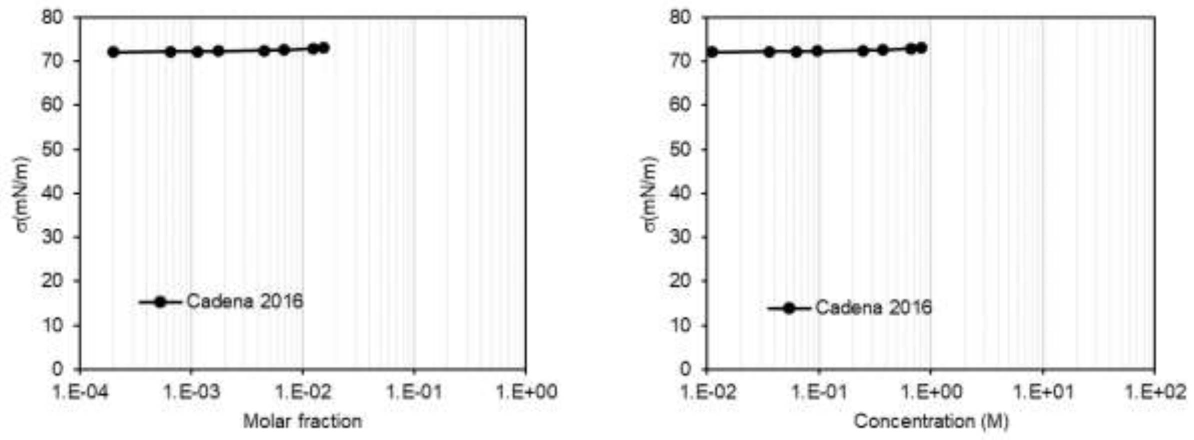

Fig. S93: Experimental data for piperidic acid / water mixtures.

Comment: N/A

SA16: DL-2-aminobutanoic acid

Rodríguez et al. 2017	**Table**	
**C (M)**	**Molar fraction**	**σ (mN/m)**
0.00E+00	**0.00E+00**	71.73
9.83E-03	**1.77E-04**	71.8
1.95E-02	**3.51E-04**	71.79
3.89E-02	**7.02E-04**	71.77
5.83E-02	**1.05E-03**	71.75
7.75E-02	**1.40E-03**	71.74
9.68E-02	**1.75E-03**	71.72
1.16E-01	**2.10E-03**	71.71
1.45E-01	**2.64E-03**	71.69

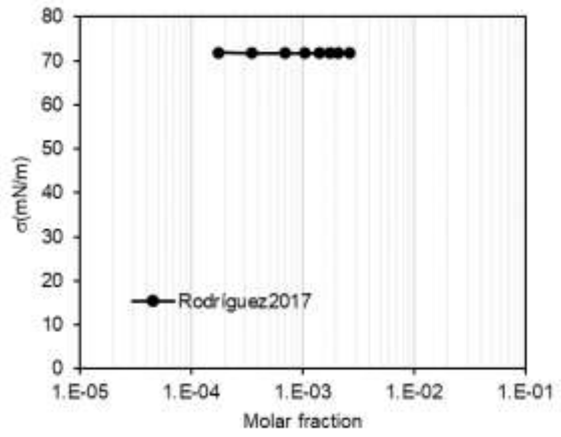

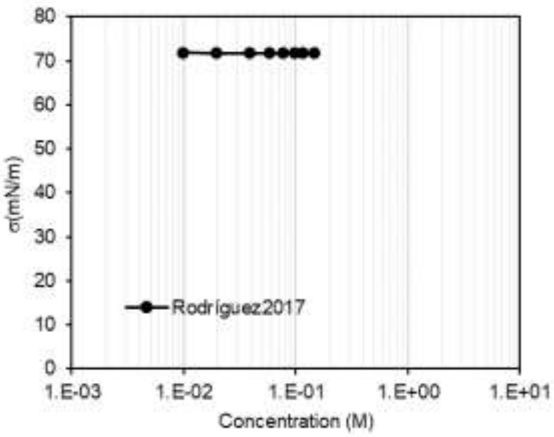

Fig. S94: Experimental data for DL-2-aminobutanoic acid / water mixtures.

Comment: N/A

SA17: 1-dimethylaminopropan-2-ol

Wang et al. 2019 **Table**			
**C (M)**	**Molar fraction**	**Mass fraction**	**σ (mN/m)**
2.75E+00	2.98E-02	**3.00E-01**	36.2
3.60E+00	4.16E-02	**4.00E-01**	33.5
4.42E+00	5.46E-02	**5.00E-01**	31.5
8.11E+00	1.46E-01	**1.00E+00**	24.0

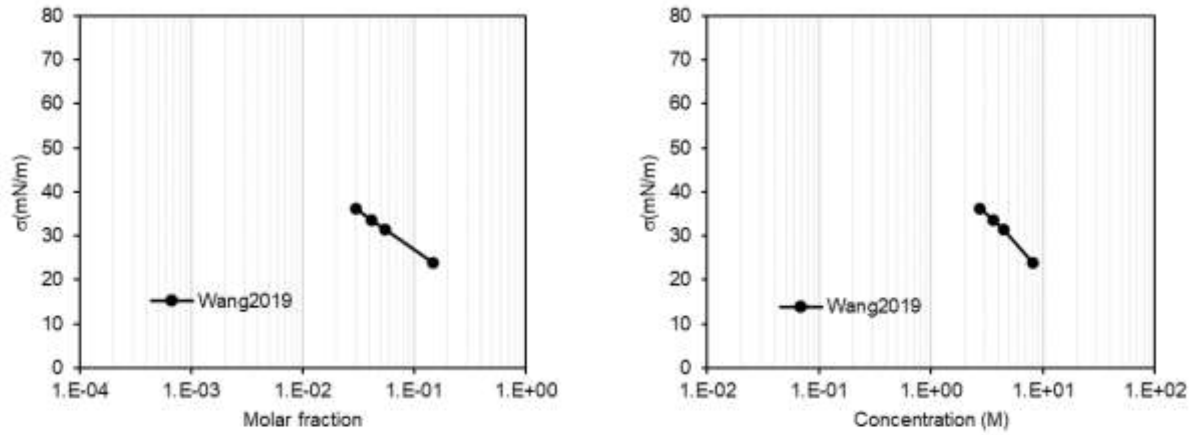

Fig. S95: Experimental data for 1-dimethylaminopropan-2-ol / water mixtures.

Comment: N/A

Wang et al. : data reported at 30°C.

SA18: diolamine (2,2′-azanediyldi(ethan-1-ol))

Rinker et al. 1994 **Table**				Aguila-Hernández et al. 2001 **Table**			
**C (M)**	**Molar fraction**	**Mass fraction**	**σ (mN/m)**	**C (M)**	**Molar fraction**	**Mass fraction**	**σ (mN/m)**
9.60E-01	1.87E-02	**1.00E-01**	63.90	9.60E-01	1.87E-02	**1.00E-01**	64.14
1.94E+00	4.10E-02	**2.00E-01**	65.27	1.94E+00	4.10E-02	**2.00E-01**	66.45
2.93E+00	6.84E-02	**3.00E-01**	61.94	2.93E+00	6.84E-02	**3.00E-01**	61.84

Vázquez et al. 1996 **Table**			***Recommended***		
**C (M)**	**Molar fraction**	**σ (mN/m)**	***C (M)***	***Molar fraction***	***σ (mN/m)***
0.00E+00	**0.00E+00**	72.01	*9.61E-01*	*1.87E-02*	*65.9*
9.75E-01	**1.90E-02**	66.70	*1.16E+00*	*2.31E-02*	*65.3*
1.93E+00	**4.10E-02**	63.25	*1.41E+00*	*2.84E-02*	*64.7*
2.92E+00	**6.80E-02**	60.75	*1.69E+00*	*3.51E-02*	*64.0*
3.93E+00	**1.02E-01**	58.82	*2.02E+00*	*4.32E-02*	*63.3*
4.97E+00	**1.46E-01**	57.20	*2.41E+00*	*5.33E-02*	*62.5*
6.02E+00	**2.04E-01**	55.75	*2.84E+00*	*6.57E-02*	*61.7*
7.09E+00	**2.85E-01**	54.32	*3.33E+00*	*8.10E-02*	*60.8*
8.19E+00	**4.07E-01**	52.72	*3.88E+00*	*9.99E-02*	*59.8*
9.30E+00	**6.06E-01**	50.65	*4.46E+00*	*1.23E-01*	*58.8*
1.04E+01	**1.00E+00**	47.21	*5.09E+00*	*1.52E-01*	*57.8*
			5.75E+00	*1.87E-01*	*56.7*
			6.42E+00	*2.31E-01*	*55.6*
			7.09E+00	*2.85E-01*	*54.5*
			7.74E+00	*3.51E-01*	*53.3*
			8.37E+00	*4.33E-01*	*52.1*
			8.96E+00	*5.33E-01*	*50.9*
			9.51E+00	*6.58E-01*	*49.7*
			1.00E+01	*8.11E-01*	*48.4*
			1.04E+01	*1.00E+00*	*47.2*

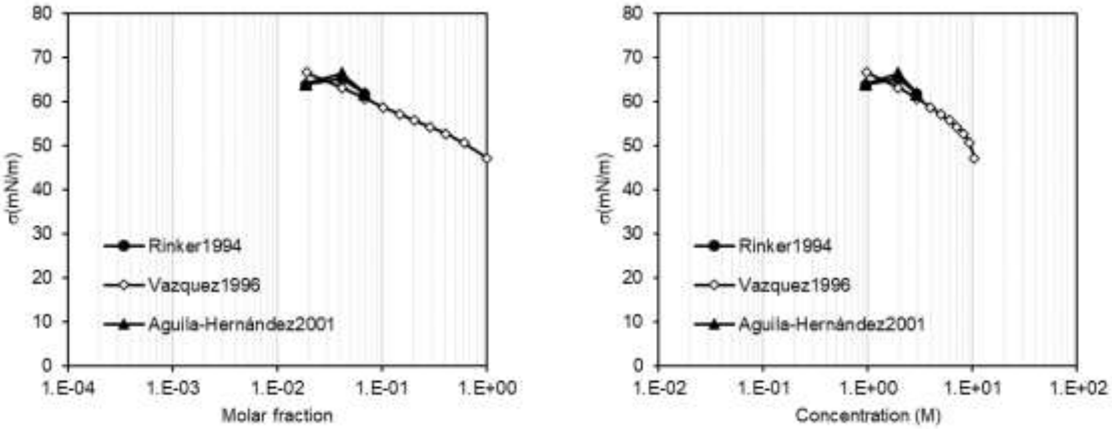

Fig. S96 (a): Comparison of the experimental data for diolamine / water mixtures.

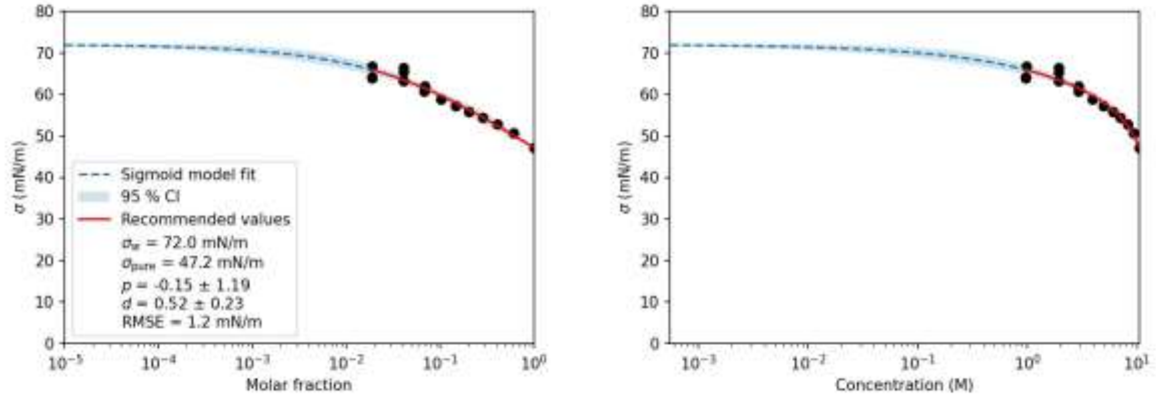

Fig. S96 (b): Surface tension fit with the Sigmoid model (*Kleinheins et al. 2023*) for diolamine / water mixtures. Solid red line: model fit inside the concentration range covered by experimental data, reported as recommended values. Blue shading: fit parameters with 95 % confidence interval (CI). RMSE: root mean squared error. Markers: data used for fitting.

Comment: N/A

SA19: 5-aminopentanoic acid

Cadena et al. 2016 **Table**		
**C (M)**	**Molar fraction**	**σ (mN/m)**
0.00E+00	**0.00E+00**	72.03
1.11E-02	**2.00E-04**	72.11
3.49E-02	**6.30E-04**	72.18
6.13E-02	**1.11E-03**	72.22
9.58E-02	**1.74E-03**	72.23
2.30E-01	**4.22E-03**	72.31
4.34E-01	**8.12E-03**	72.33
6.32E-01	**1.20E-02**	72.42
7.15E-01	**1.37E-02**	72.43

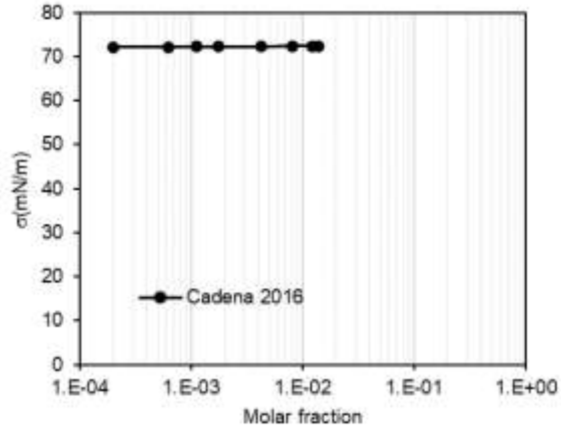

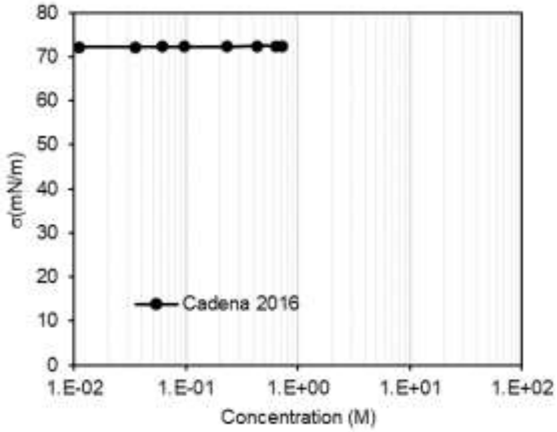

Fig. S97: Experimental data for 5-aminopentanoic acid / water mixtures.

Comment: N/A

SA20: DL-norvaline (2-aminopentanoic acid)

Rodríguez et al. 2017	**Table**	
**C (M)**	**Molar fraction**	**σ (mN/m)**
0.00E+00	**0.00E+00**	71.73
9.60E-03	**1.73E-04**	71.84
1.92E-02	**3.47E-04**	71.80
3.11E-02	**5.61E-04**	71.71
5.75E-02	**1.04E-03**	71.63
7.65E-02	**1.39E-03**	71.53
9.55E-02	**1.73E-03**	71.47
1.14E-01	**2.08E-03**	71.41
1.45E-01	**2.64E-03**	71.25

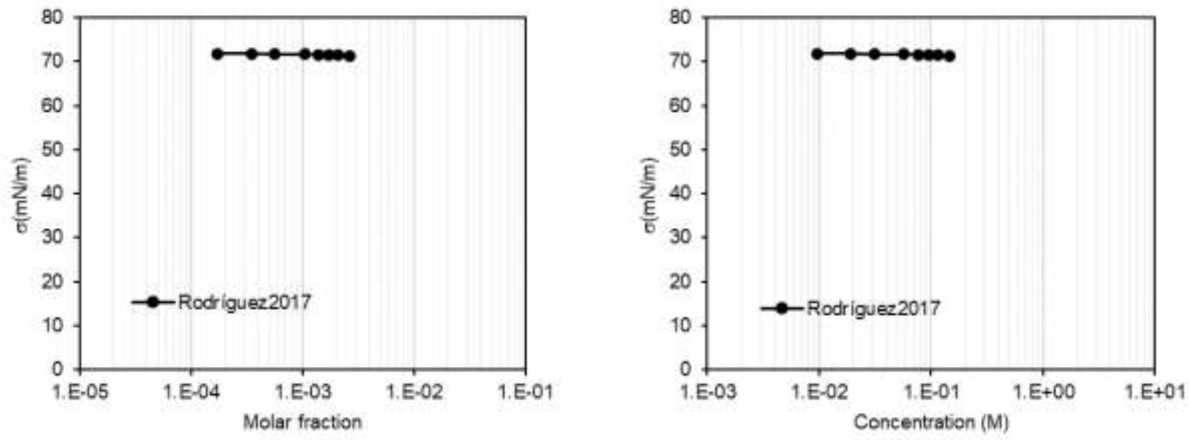

Fig. S98: Experimental data for DL-norvaline / water mixtures.

Comment: N/A

SA21: methyl diethanolamine (2.2′-(methylazanediyl)di(ethan-1-ol))

Rinker et al. 1994 **Table**				Aguila-Hernández et al. 2007 **Table**			
**C (M)**	**Molar fraction**	**Mass fraction**	**σ (mN/m)**	**C (M)**	**Molar fraction**	**Mass fraction**	**σ (mN/m)**
8.43E-01	1.65E-02	**1.00E-01**	62.24	0.00E+00	0.00E+00	**0.00E+00**	69.33
1.69E+00	3.64E-02	**2.00E-01**	58.47	8.42E-01	1.65E-02	**1.00E-01**	58.45
2.55E+00	6.08E-02	**3.00E-01**	55.27	1.69E+00	3.64E-02	**2.00E-01**	54.38
3.41E+00	9.15E-02	**4.00E-01**	52.61	2.55E+00	6.09E-02	**3.00E-01**	51.74
4.28E+00	1.31E-01	**5.00E-01**	50.28	3.42E+00	9.16E-02	**4.00E-01**	49.51
				4.29E+00	1.31E-01	**5.00E-01**	47.83
				8.75E+00	1.00E+00	**1.00E+00**	37.29

Alvarez et al. 1998 **Table**			Maham et al. 2001 **Table**			**Recommended**		
**C (M)**	**Molar fraction**	**σ (mN/m)**	**C (M)**	**Molar fraction**	**σ (mN/m)**	**C (M)**	**Molar fraction**	**σ (mN/m)**
0.00E+00	**0.00E+00**	72.01	0.00E+00	**0.00E+00**	72.0	*4.26E-01*	*8.00E-03*	*64.0*
4.26E-01	**8.00E-03**	63.72	1.21E+00	**2.46E-02**	59.1	*5.43E-01*	*1.03E-02*	*62.9*
8.66E-01	**1.70E-02**	60.60	2.10E+00	**4.73E-02**	55.0	*6.90E-01*	*1.33E-02*	*61.8*
1.27E+00	**2.60E-02**	58.53	2.99E+00	**7.56E-02**	51.3	*8.73E-01*	*1.71E-02*	*60.6*
1.68E+00	**3.60E-02**	57.28	3.65E+00	**1.01E-01**	48.4	*1.10E+00*	*2.21E-02*	*59.3*
2.12E+00	**4.80E-02**	55.61	5.36E+00	**1.99E-01**	44.7	*1.37E+00*	*2.85E-02*	*57.9*
2.56E+00	**6.10E-02**	53.50	6.49E+00	**3.11E-01**	41.8	*1.71E+00*	*3.68E-02*	*56.5*
3.40E+00	**9.10E-02**	51.35	7.20E+00	**4.22E-01**	40.4	*2.10E+00*	*4.74E-02*	*55.0*
4.28E+00	**1.31E-01**	49.59	7.52E+00	**4.90E-01**	39.7	*2.56E+00*	*6.11E-02*	*53.4*
5.17E+00	**1.85E-01**	48.23	7.89E+00	**5.89E-01**	39.3	*3.08E+00*	*7.88E-02*	*51.8*
6.05E+00	**2.61E-01**	46.00	8.46E+00	**8.17E-01**	38.6	*3.66E+00*	*1.02E-01*	*50.3*
6.94E+00	**3.77E-01**	44.20	8.75E+00	**1.00E+00**	38.3	*4.28E+00*	*1.31E-01*	*48.7*
7.84E+00	**5.76E-01**	41.80				*4.93E+00*	*1.69E-01*	*47.1*
8.75E+00	**1.00E+00**	38.90				*5.59E+00*	*2.18E-01*	*45.6*
						6.24E+00	*2.81E-01*	*44.2*
						6.85E+00	*3.62E-01*	*42.8*
						7.42E+00	*4.67E-01*	*41.5*
						7.93E+00	*6.02E-01*	*40.3*
						8.37E+00	*7.76E-01*	*39.2*
						8.75E+00	*1.00E+00*	*38.2*

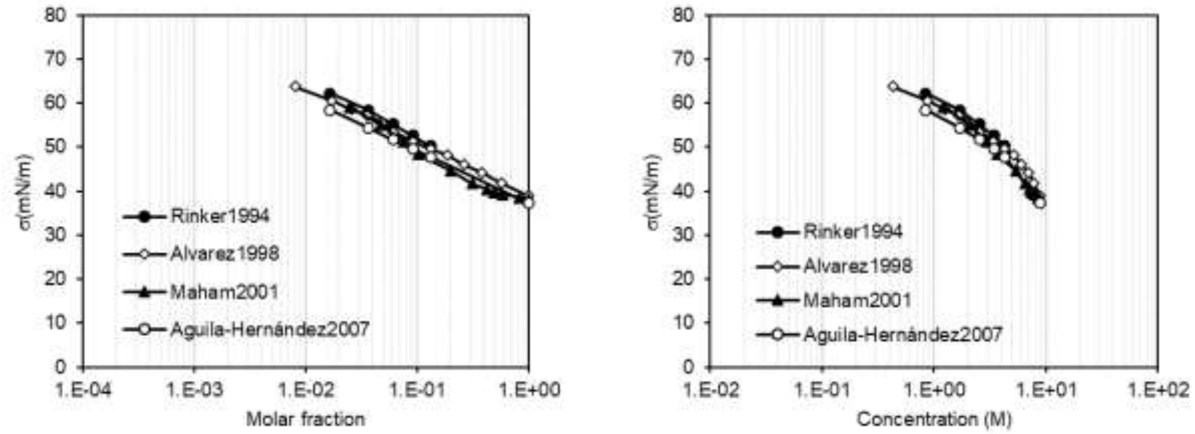

Fig. S99: Comparison of the experimental data for methyl diethanolamine / water mixtures.

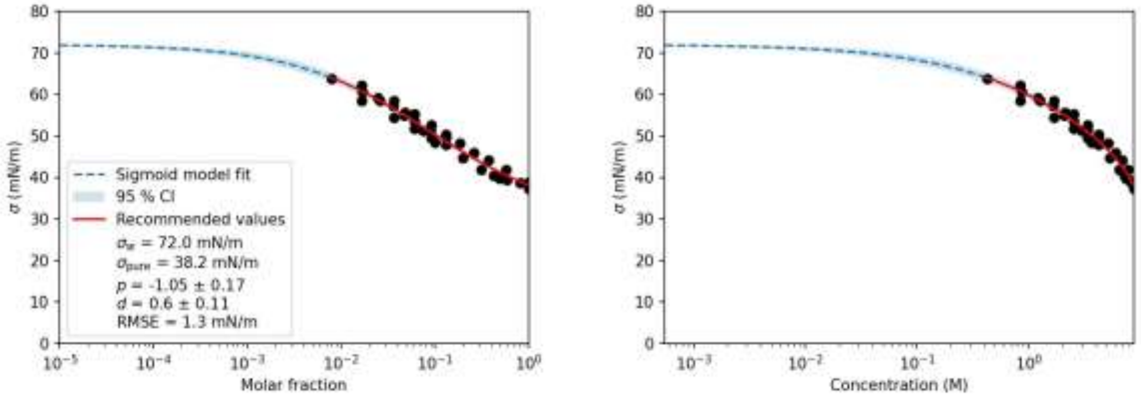

Fig. S99 (b): Surface tension fit with the Sigmoid model (*Kleinheins et al. 2023*) for methyl diethanolamine / water mixtures. Solid red line: model fit inside the concentration range covered by experimental data, reported as recommended values. Blue shading: fit parameters with 95 % confidence interval (CI). RMSE: root mean squared error. Markers: data used for fitting.

Comment :

Aguila-Hernández et al. 2007 : data reported at 40°C.

SA22: 2-amino-2-ethyl-1,3-propanediol

Jun Yoon et al. 2002 **Table**			
**C (M)**	**Molar fraction**	**Mass fraction**	**σ (mN/m)**
1.71E+00	3.64E-02	**2.00E-01**	57.51
3.48E+00	9.15E-02	**4.00E-01**	52.12
5.32E+00	1.85E-01	**6.00E-01**	48.93
6.27E+00	2.61E-01	**7.00E-01**	47.36
7.24E+00	3.77E-01	**8.00E-01**	45.78

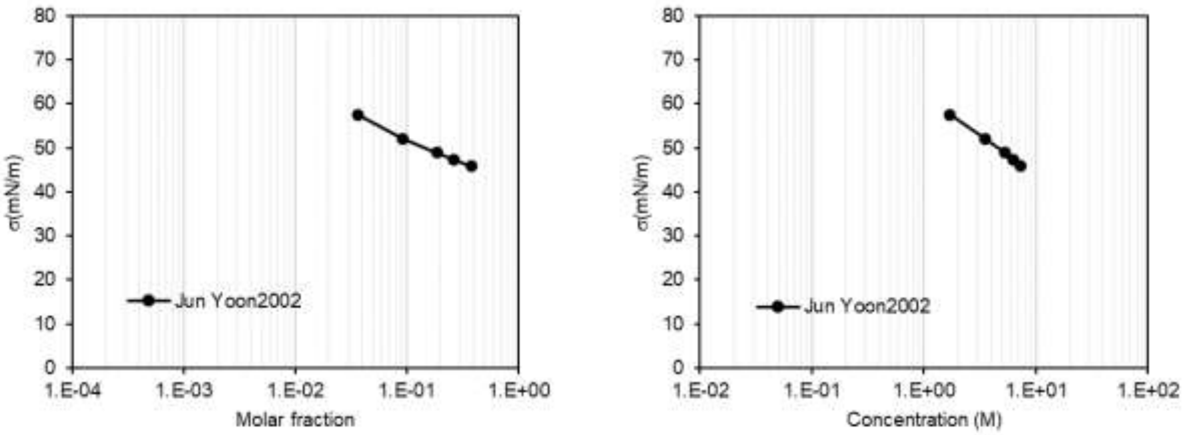

Fig. S100: Experimental data for AEPD / water mixtures.

Comment:

Jun Yoon et al. 2002 : data reported at 30°C.

SA23: 2-amino-2-hydroxymethyl-propane-1,3-diol

Murshid et al. 2011 **Table**			
**C (M)**	**Molar fraction**	**Mass fraction**	**σ (mN/m)**
8.27E-02	1.50E-03	**1.00E-02**	71.95
5.88E-01	1.11E-02	**7.00E-02**	67.81
1.11E+00	2.17E-02	**1.30E-01**	63.37
1.64E+00	3.37E-02	**1.90E-01**	60.05
2.20E+00	4.72E-02	**2.50E-01**	56.77

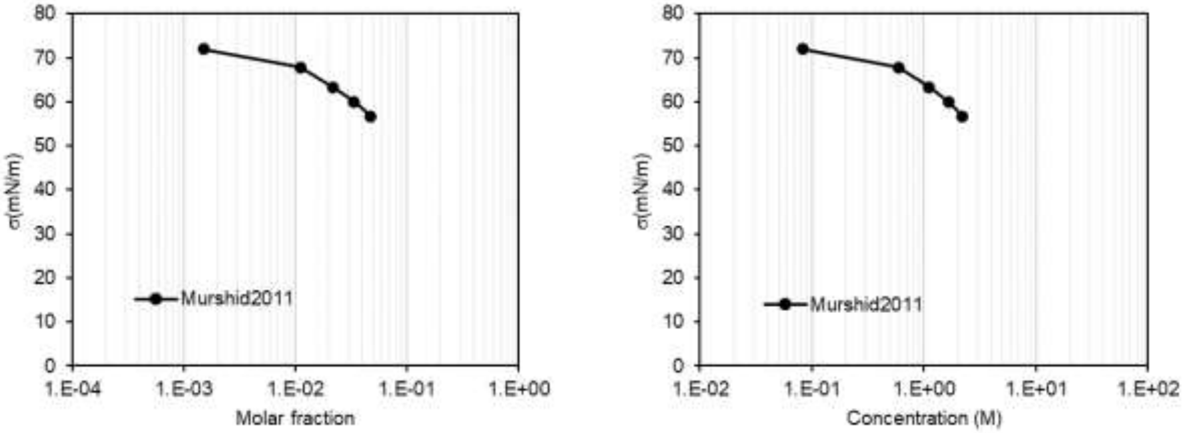

Fig. S101: Experimental data for 2-amino-2-hydroxymethyl-propane-1,3-diol / water mixtures.

Comment: N/A

SA24: erythritol ((2R,3S)-butane-1,2,3,4-tetrol)

Romero et al. 2010 **Table**			
**C (M)**	**Molar fraction**	**Molality (mol/kg)**	**σ (mN/m)**
0.00E+00	0.00E+00	**0.00E+00**	72.0
2.92E-01	5.36E-03	**3.00E-01**	72.3
5.70E-01	1.07E-02	**6.00E-01**	72.4
8.35E-01	1.59E-02	**9.00E-01**	72.5
1.09E+00	2.11E-02	**1.20E+00**	72.8
1.33E+00	2.62E-02	**1.50E+00**	72.9

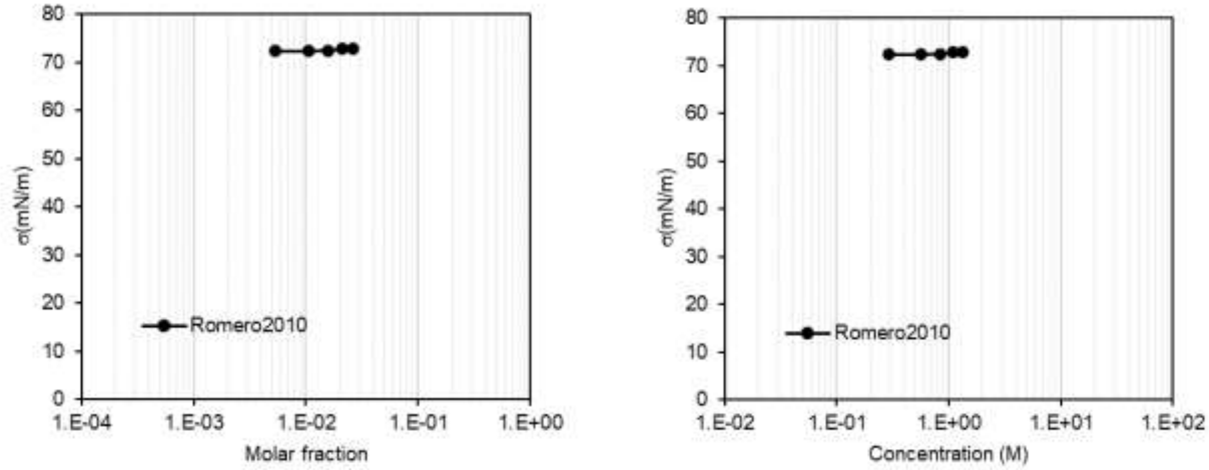

Fig. S102: Experimental data for erythritol / water mixtures.

Comment: N/A

SA25: 6-aminohexanoic acid

Cadena et al. 2016 **Table**		
**C (M)**	**Molar fraction**	**σ (mN/m)**
0.00E+00	**0.00E+00**	72.03
1.05E-02	**1.90E-04**	72.07
4.81E-02	**8.70E-04**	72.13
6.89E-02	**1.25E-03**	72.14
1.03E-01	**1.88E-03**	72.18
2.45E-01	**4.53E-03**	72.24
5.15E-01	**9.84E-03**	72.27
5.70E-01	**1.10E-02**	72.28
7.78E-01	**1.54E-02**	72.28

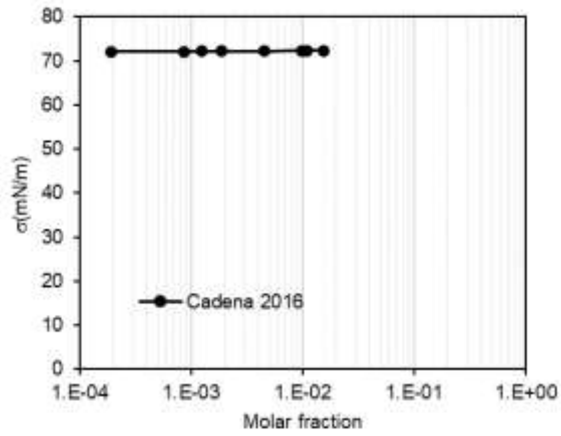

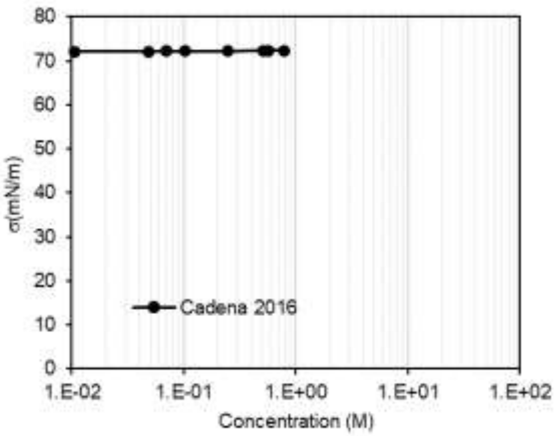

Fig. S103: Experimental data for 6-aminohexanoic acid / water mixtures.

Comment: N/A

SA26: DL-norleucine (2-aminohexanoic acid)

Rodríguez et al. 2017 **Table**		
**C (M)**	**Molar fraction**	**σ (mN/m)**
0.00E+00	**0.00E+00**	71.73
8.66E-03	**1.56E-04**	71.55
1.72E-02	**3.11E-04**	71.28
2.90E-02	**5.24E-04**	71.14
3.87E-02	**6.99E-04**	71.00
4.83E-02	**8.73E-04**	70.82
5.79E-02	**1.05E-03**	70.59
6.75E-02	**1.22E-03**	70.42
7.78E-02	**1.41E-03**	70.18

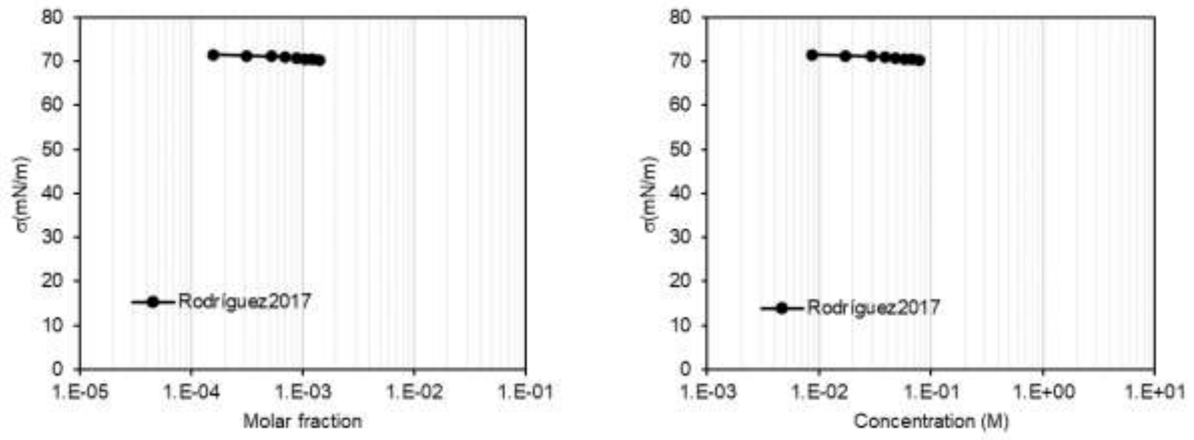

Fig. S104: Experimental data for DL-norleucine / water mixtures.

Comment: N/A

SA27: L-leucine ((S)-2-amino-4-methylpentanoic acid)

Glinski et al. 2000b **Graph (experimental points)**			Matubayasi et al. 2005 **Graph (experimental points)**			***Recommended***		
**C (M)**	**Molar fraction**	**σ (mN/m)**	**C (M)**	**Molar fraction**	**σ (mN/m)**	***C (M)***	***Molar fraction***	***σ (mN/m)***
0.00E+00	**0.00E+00**	72.0	**1.10E-02**	1.98E-04	71.7	*9.99E-03*	*1.80E-04*	*71.8*
1.00E-02	**1.80E-04**	71.5	**1.20E-02**	2.16E-04	71.7	*1.16E-02*	*2.08E-04*	*71.7*
2.00E-02	**3.61E-04**	71.0	**1.70E-02**	3.06E-04	71.6	*1.34E-02*	*2.41E-04*	*71.7*
3.00E-02	**5.41E-04**	71.0	**2.00E-02**	3.61E-04	71.6	*1.55E-02*	*2.79E-04*	*71.6*
4.00E-02	**7.22E-04**	70.5	**2.60E-02**	4.69E-04	71.3	*1.79E-02*	*3.22E-04*	*71.5*
5.00E-02	**9.04E-04**	70.5	**2.70E-02**	4.87E-04	71.4	*2.07E-02*	*3.73E-04*	*71.5*
6.00E-02	**1.09E-03**	69.5	**2.90E-02**	5.23E-04	71.4	*2.39E-02*	*4.31E-04*	*71.4*
7.00E-02	**1.27E-03**	68.5	**3.50E-02**	6.32E-04	71.3	*2.76E-02*	*4.99E-04*	*71.3*
8.00E-02	**1.45E-03**	68.5	**3.70E-02**	6.68E-04	71.2	*3.20E-02*	*5.77E-04*	*71.1*
9.00E-02	**1.63E-03**	68.0	**4.30E-02**	7.77E-04	71.1	*3.69E-02*	*6.67E-04*	*71.0*
1.20E-01	**2.18E-03**	67.5	**4.90E-02**	8.86E-04	70.8	*4.27E-02*	*7.72E-04*	*70.8*
1.30E-01	**2.37E-03**	67.5	**5.00E-02**	9.04E-04	70.8	*4.94E-02*	*8.93E-04*	*70.6*
			**5.30E-02**	9.58E-04	70.8	*5.71E-02*	*1.03E-03*	*70.4*
			**6.00E-02**	1.09E-03	70.6	*6.60E-02*	*1.19E-03*	*70.1*
			**6.30E-02**	1.14E-03	70.5	*7.63E-02*	*1.38E-03*	*69.8*
			**6.50E-02**	1.18E-03	70.4	*8.81E-02*	*1.60E-03*	*69.5*
			**6.60E-02**	1.19E-03	70.5	*1.02E-01*	*1.85E-03*	*69.1*
			**7.00E-02**	1.27E-03	70.3	*1.18E-01*	*2.14E-03*	*68.6*
			**7.50E-02**	1.36E-03	70.3	*1.36E-01*	*2.47E-03*	*68.1*
			**8.30E-02**	1.50E-03	70.0	*1.57E-01*	*2.86E-03*	*67.5*
			**8.30E-02**	1.50E-03	70.0			
			**8.60E-02**	1.56E-03	69.9			
			**8.90E-02**	1.61E-03	69.9			
			**9.90E-02**	1.80E-03	69.5			
			**9.90E-02**	1.80E-03	69.6			
			**1.11E-01**	2.02E-03	69.1			
			**1.18E-01**	2.15E-03	68.9			
			**1.25E-01**	2.27E-03	68.6			
			**1.26E-01**	2.29E-03	68.6			
			**1.27E-01**	2.31E-03	68.6			
			**1.35E-01**	2.46E-03	68.2			
			**1.41E-01**	2.57E-03	68.1			
			**1.57E-01**	2.86E-03	67.5			

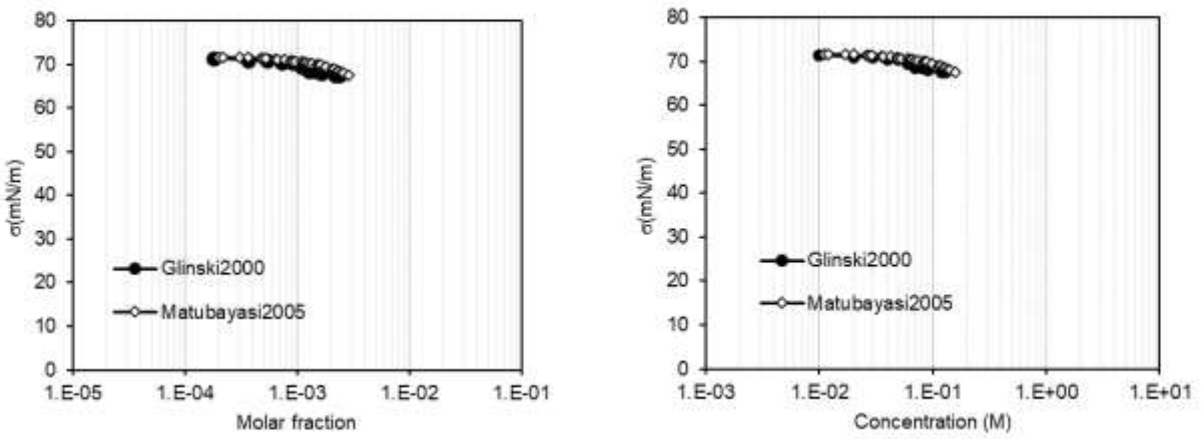

Fig. S105 (a): Comparison of the experimental data for L-leucine / water mixtures.

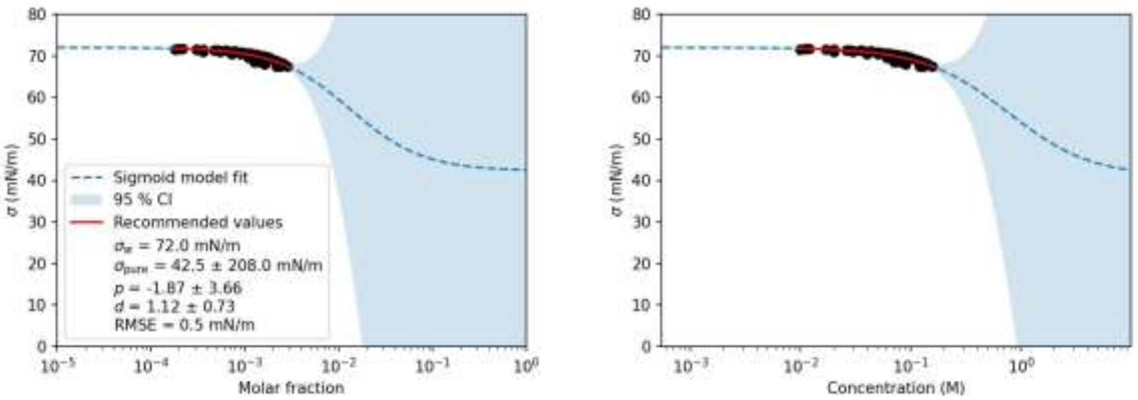

Fig. S105 (b): Surface tension fit with the Sigmoid model (*Kleinheins et al. 2023*) for L-leucine / water mixtures. Solid red line: model fit inside the concentration range covered by experimental data, reported as recommended values. Blue shading: fit parameters with 95 % confidence interval (CI). RMSE: root mean squared error. Markers: data used for fitting. **Note: the modelling is reliable only in the range of the experimental data. Therefore, for this compound, the modelling should be used only in the experimental data range.**

Comment: N/A

SA28: hexamethylenetetramine (1,3,5,7-tetraazaadamantane)

Gómez-Díaz et al. 2018 **Table**		
**C (M)**	**Molar fraction**	**σ (mN/m)**
**0.00E+00**	0.00E+00	72.6
**2.07E-01**	3.79E-03	69.2
**4.25E-01**	7.94E-03	67.4
**6.54E-01**	1.25E-02	66.2
**8.97E-01**	1.75E-02	65.4
**1.16E+00**	2.33E-02	64.8
**1.45E+00**	2.99E-02	64.4
**1.75E+00**	3.71E-02	64.0
**2.07E+00**	4.54E-02	63.7
**2.41E+00**	5.50E-02	63.5
**2.78E+00**	6.60E-02	63.3
**3.18E+00**	7.93E-02	63.2
**3.62E+00**	9.52E-02	63.1
**4.09E+00**	1.14E-01	63.0
**4.61E+00**	1.39E-01	62.9
**5.16E+00**	1.69E-01	62.9

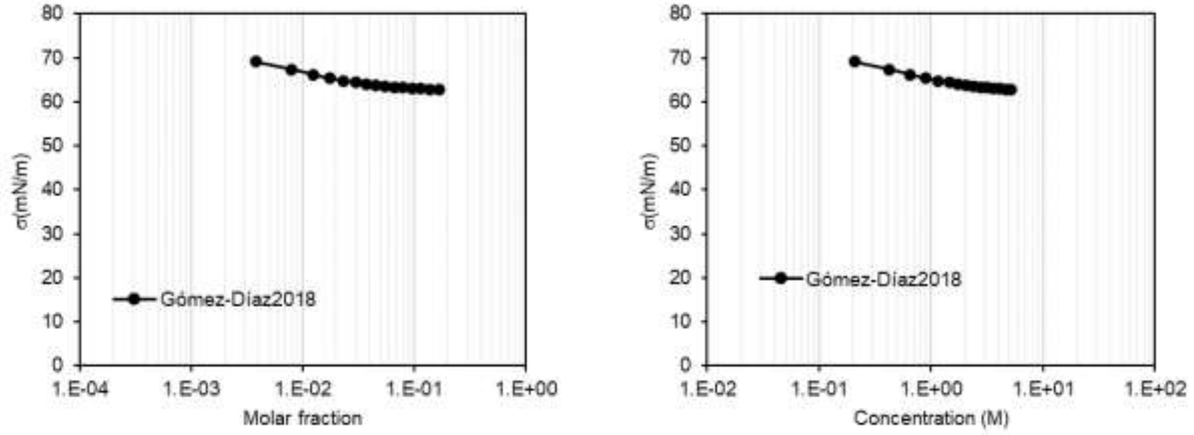

Fig. S106: Experimental data for hexamethylenetetramine / water mixtures.

Comment: N/A

SA29: trolamine (2,2′,2″-nitrilotri(ethan-1-ol))

Vázquez et al. 1996 **Table**		
**C (M)**	**Molar fraction**	**σ (mN/m)**
**0.00E+00**	0.00E+00	72.01
**6.67E-01**	1.30E-02	65.85
**1.36E+00**	2.90E-02	61.32
**2.07E+00**	4.90E-02	57.80
**2.79E+00**	7.40E-02	55.10
**3.55E+00**	1.08E-01	52.88
**4.30E+00**	1.53E-01	51.03
**5.09E+00**	2.20E-01	49.48
**5.89E+00**	3.26E-01	48.16
**6.70E+00**	5.21E-01	47.00
**7.54E+00**	1.00E+00	45.95

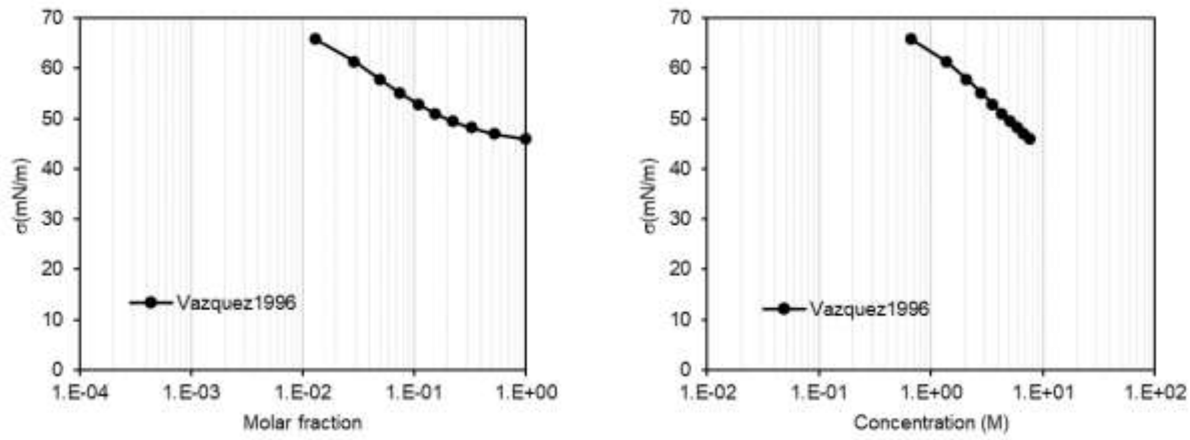

Fig. S107: Experimental data for trolamine / water mixtures.

Comment: N/A

SA30: xylitol (meso-Xylitol)

Romero et al. 2010 **Table**			
**C (M)**	**Molar fraction**	**Molality (mol/kg)**	**σ (mN/m)**
0.00E+00	0.00E+00	**0.00E+00**	72.0
2.91E-01	5.36E-03	**3.00E-01**	72.5
5.65E-01	1.07E-02	**6.00E-01**	72.7
8.24E-01	1.59E-02	**9.00E-01**	73.0
1.07E+00	2.11E-02	**1.20E+00**	73.3
1.30E+00	2.62E-02	**1.50E+00**	73.5

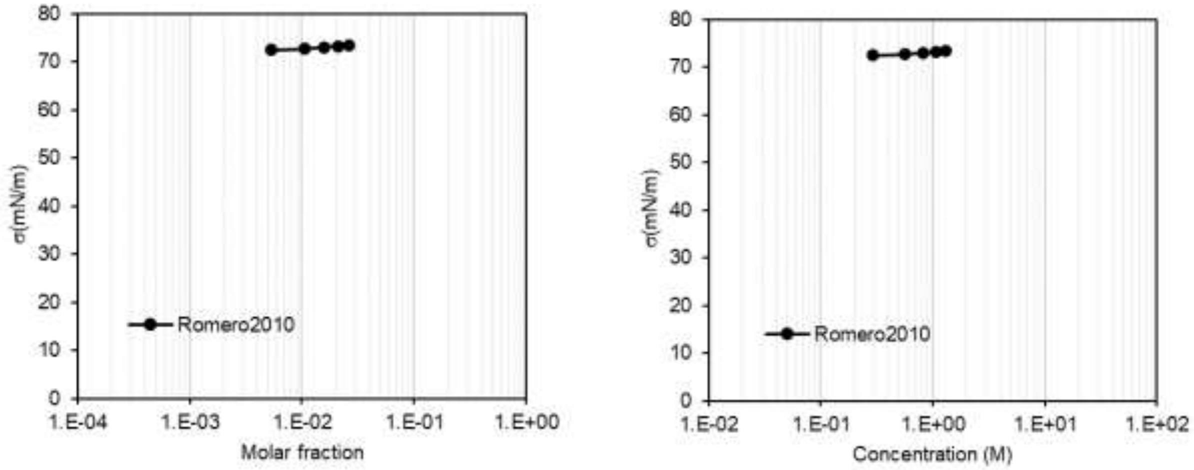

Fig. S108: Experimental data for xylitol / water mixtures.

Comment: N/A

SA31: levoglucosan ((1R,2S,3S,4R,5R)-6,8- dioxabicyclo[3.2.1]octane-2,3,4-triol)

Svenningsson et al. 2006 **Graph (experimental points)**				Topping et al. 2007 **Graph (experimental points)**			
**C (M)**	**Molar fraction**	**Molality (mol/kgw)**	**σ (mN/m)**	**C (M)**	**Molar fraction**	**Mass fraction**	**σ (mN/m)**
3.11E-01	5.75E-03	**3.22E-01**	72.0	0.00E+00	0.00E+00	**0.00E+00**	73.0
1.45E+00	2.96E-02	**1.70E+00**	71.1	3.14E-01	5.81E-03	**5.00E-02**	72.0
5.76E+00	1.99E-01	**1.39E+01**	70.0	1.89E+00	4.00E-02	**2.75E-01**	70.0

Auman et al. 2009 **Graph (experimental points)**			Tuckerman et al. 2004 **Graph (experimental points)**				***Recommended***		
**C (M)**	**Molar fraction**	**σ (mN/m)**	**C (M)**	**Molar fraction**	**C (mg/mL)**	**σ (mN/m)**	***C (M)***	***Molar fraction***	***σ (mN/m)***
**1.32E-02**	2.38E-04	71.8	5.92E-06	1.07E-07	**9.60E-04**	72.7	*5.94E-06*	*1.07E-07*	*72.0*
**2.65E-02**	4.78E-04	71.8	1.20E-05	2.15E-07	**1.94E-03**	73.1	*1.27E-05*	*2.29E-07*	*72.0*
**6.49E-02**	1.17E-03	71.7	2.34E-05	4.22E-07	**3.80E-03**	72.9	*2.72E-05*	*4.89E-07*	*72.0*
**1.29E-01**	2.35E-03	71.7	4.75E-05	8.55E-07	**7.70E-03**	73.3	*5.81E-05*	*1.05E-06*	*72.0*
**4.03E-01**	7.51E-03	71.3	9.99E-05	1.80E-06	**1.62E-02**	72.8	*1.24E-04*	*2.23E-06*	*72.0*
**6.80E-01**	1.30E-02	71.1	1.91E-04	3.44E-06	**3.10E-02**	73.1	*2.65E-04*	*4.78E-06*	*72.0*
**1.32E+00**	2.67E-02	69.5	3.89E-04	6.99E-06	**6.30E-02**	72.9	*5.67E-04*	*1.02E-05*	*72.0*
			7.71E-04	1.39E-05	**1.25E-01**	73.0	*1.21E-03*	*2.18E-05*	*72.0*
			1.58E-03	2.84E-05	**2.56E-01**	73.0	*2.59E-03*	*4.67E-05*	*72.0*
			3.06E-03	5.51E-05	**4.96E-01**	73.1	*5.54E-03*	*9.98E-05*	*72.0*
			6.42E-03	1.16E-04	**1.04E+00**	73.1	*1.18E-02*	*2.13E-04*	*72.0*
			1.22E-02	2.20E-04	**1.98E+00**	72.8	*2.53E-02*	*4.56E-04*	*72.0*
			3.19E-02	5.75E-04	**5.17E+00**	72.9	*5.39E-02*	*9.75E-04*	*72.0*
							1.15E-01	*2.08E-03*	*72.0*
							2.43E-01	*4.46E-03*	*71.9*
							5.07E-01	*9.53E-03*	*71.4*
							1.04E+00	*2.04E-02*	*70.5*
							2.02E+00	*4.35E-02*	*70.1*
							3.63E+00	*9.31E-02*	*70.0*
							5.80E+00	*1.99E-01*	*70.0*

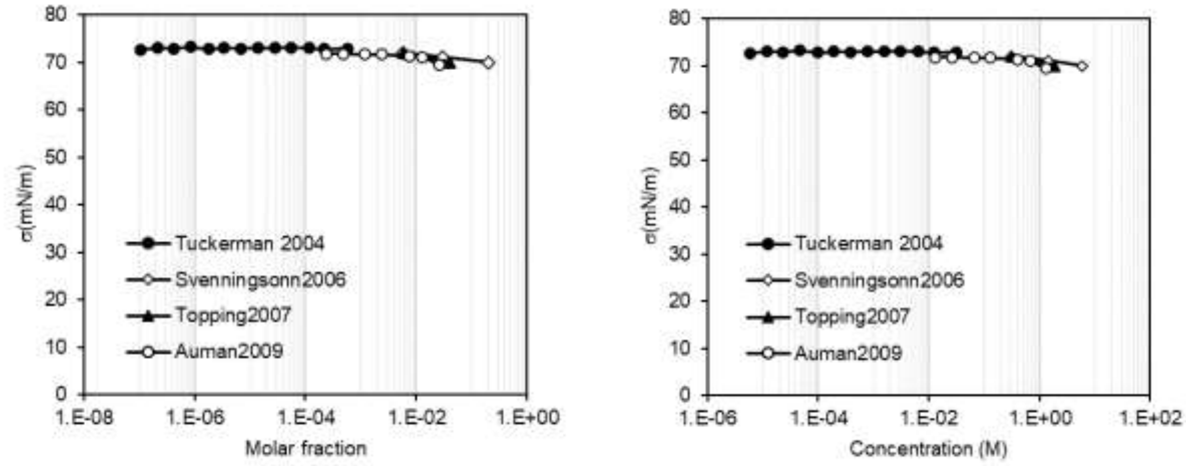

Fig. S109 (a): Comparison of the experimental data for levoglucosan / water mixtures.

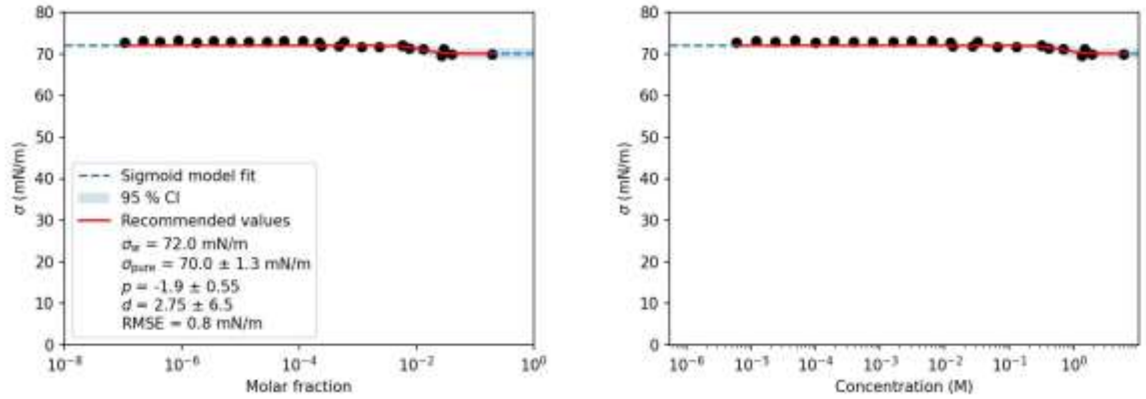

Fig. S109 (b): Surface tension fit with the Sigmoid model (*Kleinheins et al. 2023*) for levoglucosan / water mixtures. Solid red line: model fit inside the concentration range covered by experimental data, reported as recommended values. Blue shading: fit parameters with 95 % confidence interval (CI). RMSE: root mean squared error. Markers: data used for fitting.

Comment: N/A

SA32: L-phenylalanine

Chandra et al. 2013 **Table**		
**C (M)**	**Molar fraction**	**σ (mN/m)**
**0.00E+00**	0.00E+00	71.97
**5.05E-02**	9.15E-04	71.09
**7.59E-02**	1.38E-03	70.66
**1.02E-01**	1.85E-03	69.74
**1.27E-01**	2.33E-03	68.37
**1.53E-01**	2.81E-03	66.91

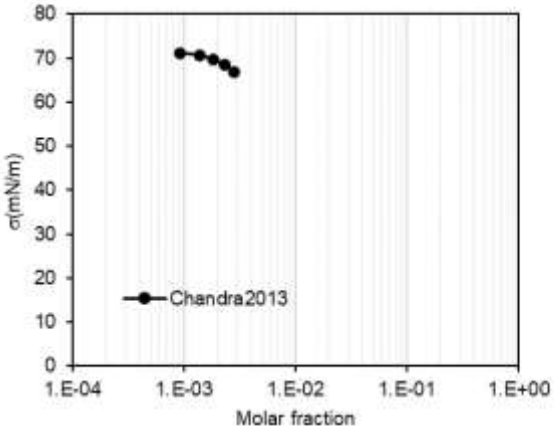

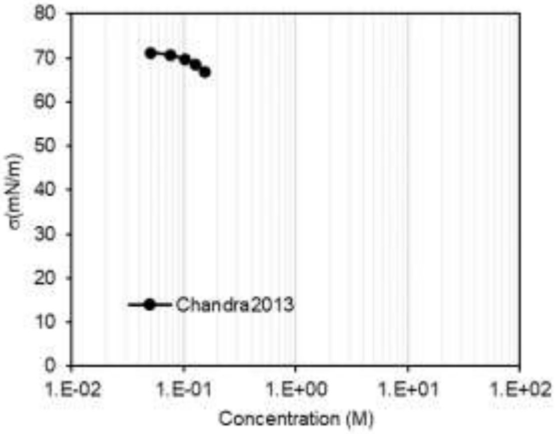

Fig. S110: Experimental data for L-phenylalanine / water mixtures.

Comment: N/A

SA33: D-(+)-glucose ((2R,3S,4R,5R)-2,3,4,5,6-pentahydroxyhexanal)

Romero et al. 2010 **Table**				Lee et al. 2013 **Graph (experimental points)**			
**C (M)**	**Molar fraction**	**Molality (mol/kg)**	**σ (mN/m)**	**C (M)**	**Molar fraction**	**Molality (mol/kgw)**	**σ (mN/m)**
0.00E+00	0.00E+00	**0.00E+00**	72.0	1.04E-02	1.87E-04	**1.04E-02**	72.1
2.89E-01	5.36E-03	**3.00E-01**	71.3	9.84E-02	1.79E-03	**9.97E-02**	72.3
5.60E-01	1.07E-02	**6.00E-01**	71.8	2.79E-01	5.16E-03	**2.89E-01**	72.4
8.13E-01	1.59E-02	**9.00E-01**	71.9	4.71E-01	8.88E-03	**4.99E-01**	72.8
1.05E+00	2.11E-02	**1.20E+00**	72.3	8.78E-01	1.73E-02	**9.81E-01**	72.9
1.27E+00	2.62E-02	**1.50E+00**	72.9	1.11E+00	2.25E-02	**1.28E+00**	72.9
				1.64E+00	3.54E-02	**2.04E+00**	73.9

Auman et al. 2009 **Graph (experimental points)**			***Recommended***		
**C (M)**	**Molar fraction**	**σ (mN/m)**	***C (M)***	***Molar fraction***	***σ (mN/m)***
**1.66E-02**	2.99E-04	72.1	*1.04E-02*	*1.87E-04*	*72.0*
**3.16E-02**	5.71E-04	72.1	*1.43E-02*	*2.59E-04*	*72.0*
**6.57E-02**	1.19E-03	72.1	*1.98E-02*	*3.58E-04*	*72.0*
**1.72E-01**	3.15E-03	72.3	*2.74E-02*	*4.94E-04*	*72.0*
**4.17E-01**	7.83E-03	72.5	*3.78E-02*	*6.84E-04*	*72.0*
**8.30E-01**	1.63E-02	72.9	*5.23E-02*	*9.46E-04*	*72.0*
**1.65E+00**	3.54E-02	74.1	*7.21E-02*	*1.31E-03*	*72.0*
**2.48E+00**	5.91E-02	75.2	*9.95E-02*	*1.81E-03*	*72.0*
**3.31E+00**	8.84E-02	76.4	*1.37E-01*	*2.50E-03*	*72.0*
			1.89E-01	*3.46E-03*	*72.0*
			2.59E-01	*4.78E-03*	*72.0*
			3.54E-01	*6.61E-03*	*72.1*
			4.84E-01	*9.14E-03*	*72.1*
			6.57E-01	*1.26E-02*	*72.3*
			8.86E-01	*1.75E-02*	*72.5*
			1.19E+00	*2.42E-02*	*73.0*
			1.57E+00	*3.34E-02*	*73.7*
			2.05E+00	*4.62E-02*	*74.6*
			2.63E+00	*6.39E-02*	*75.5*
			3.31E+00	*8.84E-02*	*76.4*

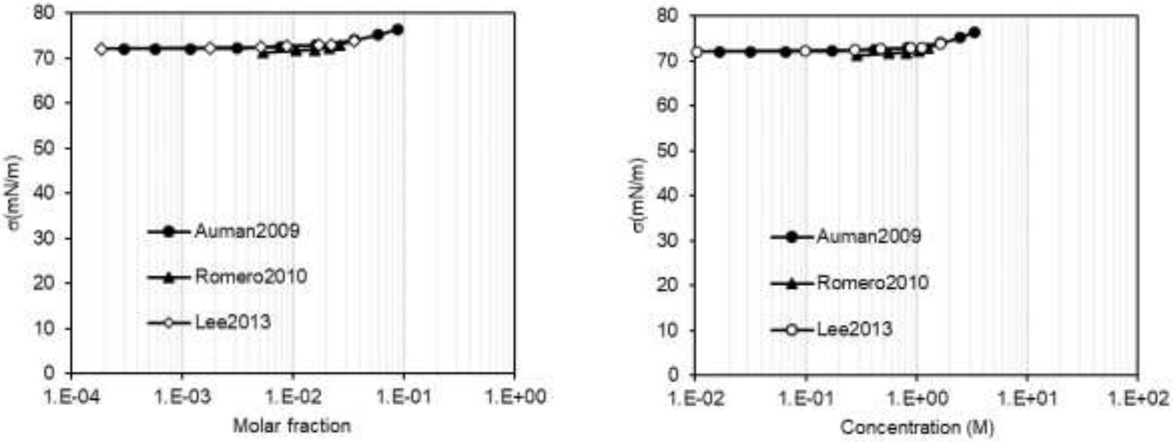

Fig. S111 (a): Comparison of the experimental data for D-(+)-glucose / water mixtures.

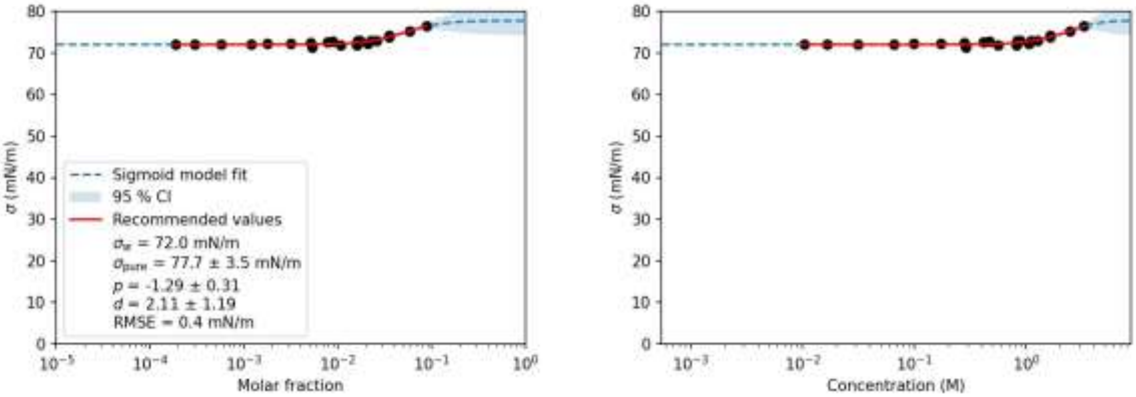

Fig. S111 (b): Surface tension fit with the Sigmoid model (*Kleinheins et al. 2023*) for D-(+)-glucose / water mixtures. Solid red line: model fit inside the concentration range covered by experimental data, reported as recommended values. Blue shading: fit parameters with 95 % confidence interval (CI). RMSE: root mean squared error. Markers: data used for fitting.

Comment: N/A

SA34: D-(+)-galactose ((2R,3S,4S,5R)-2,3,4,5,6-pentahydroxyhexanal)

Auman et al. 2009 **Graph (experimental points)**		
**C (M)**	**Molar fraction**	**σ (mN/m)**
**1.26E-02**	2.27E-04	72.0
**2.45E-02**	4.42E-04	72.1
**4.98E-02**	9.01E-04	72.2
**1.21E-01**	2.21E-03	72.2
**3.09E-01**	5.74E-03	72.3
**6.35E-01**	1.22E-02	72.6
**1.22E+00**	2.51E-02	73.3
**1.82E+00**	4.02E-02	73.8
**2.48E+00**	5.97E-02	74.2

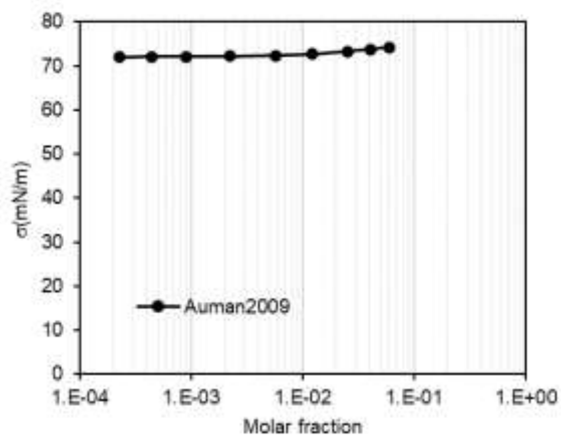

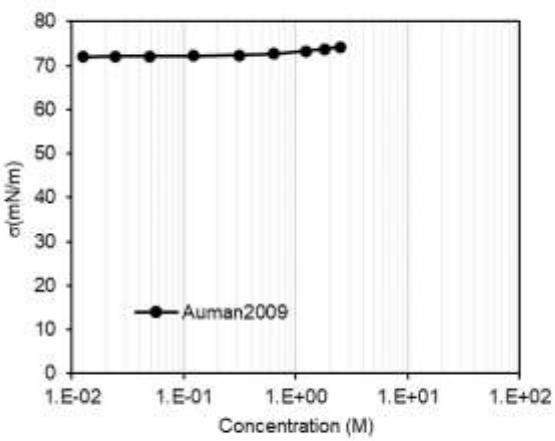

Fig. S112: Experimental data for D-(+)-galactose / water mixtures.

Comment: N/A

SA35: inositol (1,2,3,4,5,6-hexahydroxycyclohexane)

Romero et al. 2010 **Table**			
**C (M)**	**Molar fraction**	**Molality (mol/kg)**	**σ (mN/m)**
0.00E+00	0.00E+00	**0.00E+00**	72.0
1.47E-01	2.69E-03	**1.50E-01**	72.0
2.90E-01	5.36E-03	**3.00E-01**	72.1
5.64E-01	1.07E-02	**6.00E-01**	72.5
6.95E-01	1.33E-02	**7.50E-01**	72.7

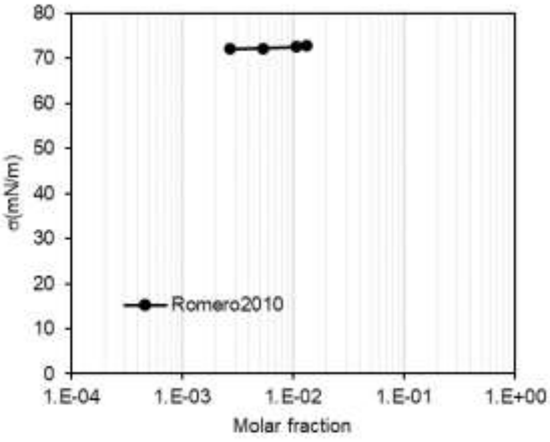

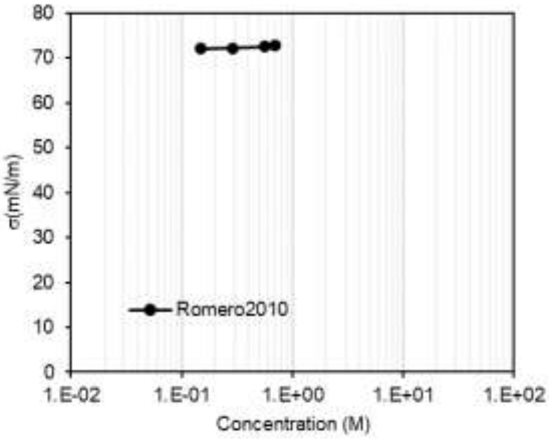

Fig. S113: Experimental data for inositol / water mixtures.

Comment: N/A

SA36: sorbitol ((2S,3R,4R,5R)-hexane-1,2,3,4,5,6-hexol)

Romero et al. 2010 **Table**			
**C (M)**	**Molar fraction**	**Molality (mol/kg)**	**σ (mN/m)**
0.00E+00	0.00E+00	**0.00E+00**	72.0
2.89E-01	5.36E-03	**3.00E-01**	72.4
5.58E-01	1.07E-02	**6.00E-01**	72.8
8.09E-01	1.59E-02	**9.00E-01**	73.0
1.04E+00	2.11E-02	**1.20E+00**	73.4
1.27E+00	2.62E-02	**1.50E+00**	73.8

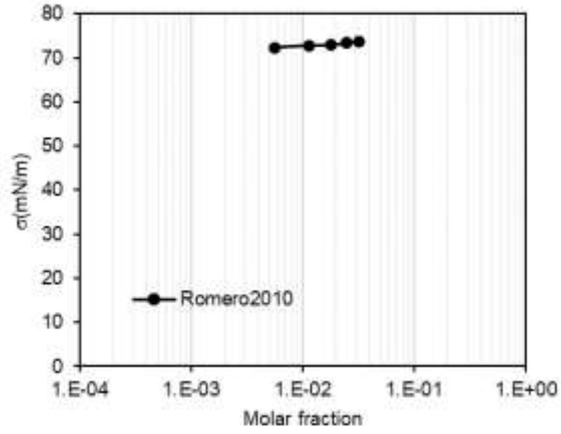

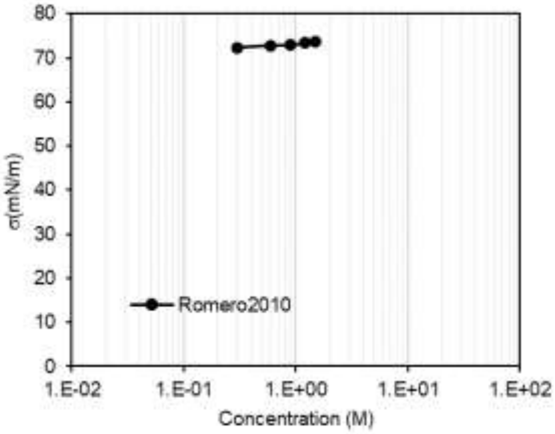

Fig. S114: Experimental data for sorbitol / water mixtures.

Comment: N/A

SA37: D-(+)-maltose ((3R,4R,5S,6R)-6-(hydroxymethyl)-5-[[(2R,3R,4S,5S,6R)-3,4,5-trihydroxy-6-(hydroxymethyl)oxan-2-yl]oxy]oxane-2,3,4-triol)

Auman et al. 2009 **Graph (experimental points)**		
**C (M)**	**Molar fraction**	**σ (mN/m)**
**1.57E-02**	2.83E-04	71.9
**3.20E-02**	5.79E-04	71.9
**8.01E-02**	1.46E-03	71.9
**1.94E-01**	3.61E-03	71.8
**3.99E-01**	7.71E-03	71.5
**7.33E-01**	1.51E-02	71.0
**1.15E+00**	2.58E-02	70.4
**1.54E+00**	3.77E-02	69.9

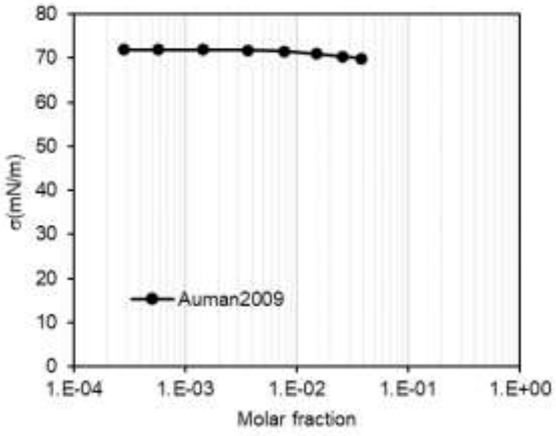

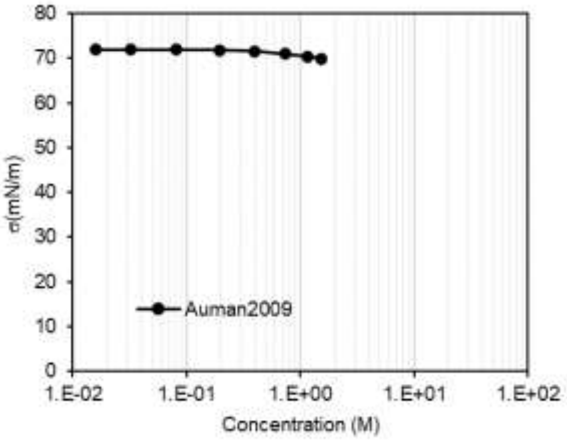

Fig. S115: Experimental data for D-(+)-maltose / water mixtures.

Comment: N/A

SA38: sucrose (β-D-fructofuranosyl α-D-glucopyranoside)

Auman et al. 2009 **Graph (experimental points)**		
**C (M)**	**Molar fraction**	**σ (mN/m)**
**1.00E-02**	1.80E-04	72.1
**2.01E-02**	3.63E-04	72.1
**4.10E-02**	7.44E-04	72.1
**1.05E-01**	1.93E-03	72.2
**2.53E-01**	4.79E-03	72.4
**5.20E-01**	1.04E-02	72.8
**1.05E+00**	2.37E-02	73.7
**1.59E+00**	4.19E-02	74.8
**2.05E+00**	6.24E-02	75.9

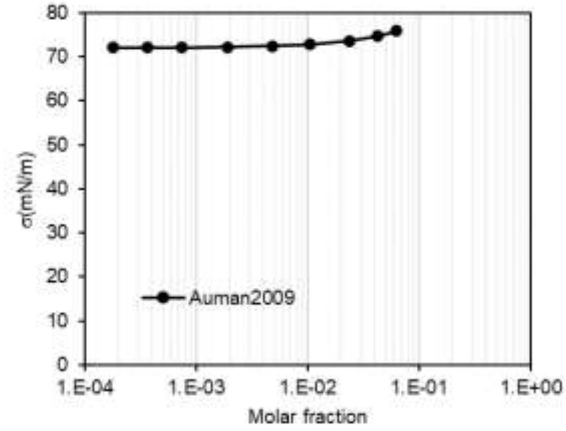

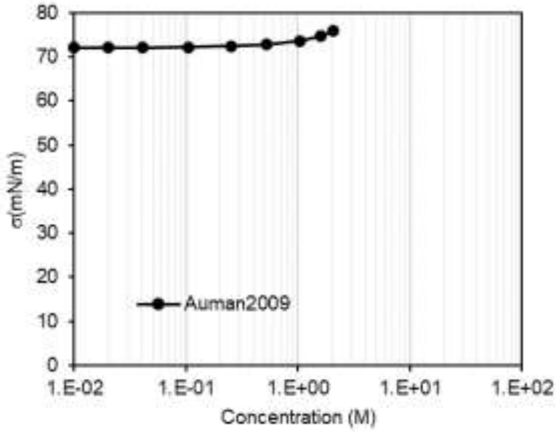

Fig. S116: Experimental data for sucrose / water mixtures.

Comment: N/A

S3.4 Synthetic and biological surfactants

Note that the list of references is not exhaustive for these surfactants.

SB1: SDS (sodium dodecyl sulfate)

Persson et al. 2003 **Graph (experimental points)**		
**C (M)**	**Molar fraction**	**σ (mN/m)**
**9.90E-04**	1.78E-05	67.7
**1.92E-03**	3.45E-05	62.6
**3.52E-03**	6.34E-05	54.9
**4.21E-03**	7.58E-05	52.3
**4.77E-03**	8.60E-05	50.1
**5.47E-03**	9.86E-05	47.9
**6.01E-03**	1.08E-04	46.3
**6.54E-03**	1.18E-04	44.8
**7.04E-03**	1.27E-04	43.6
**7.57E-03**	1.37E-04	42.2
**7.90E-03**	1.42E-04	41.1
**8.24E-03**	1.49E-04	40.4
**8.77E-03**	1.58E-04	39.4
**9.15E-03**	1.65E-04	39.1
**9.54E-03**	1.72E-04	39.1
**1.05E-02**	1.89E-04	39.1

Zdziennicka et al. 2012 **Graph (experimental points)**			
**C (M)**	**Molar fraction**	**logC (M)**	**σ (mN/m)**
1.00E-08	1.80E-10	**-8.00**	72.8
1.00E-07	1.80E-09	**-7.00**	72.9
5.06E-07	9.11E-09	**-6.30**	73.1
9.65E-07	1.74E-08	**-6.02**	72.9
4.03E-06	7.25E-08	**-5.39**	72.9
7.90E-06	1.42E-07	**-5.10**	72.7
1.05E-05	1.88E-07	**-4.98**	72.6
1.88E-05	3.39E-07	**-4.73**	72.8
6.46E-05	1.16E-06	**-4.19**	72.3
9.84E-05	1.77E-06	**-4.01**	72.1
2.10E-04	3.78E-06	**-3.68**	71.4
6.09E-04	1.10E-05	**-3.22**	68.2
8.28E-04	1.49E-05	**-3.08**	66.3
1.00E-03	1.80E-05	**-3.00**	64.3
1.87E-03	3.36E-05	**-2.73**	57.5
3.87E-03	6.98E-05	**-2.41**	49.7
5.89E-03	1.06E-04	**-2.23**	43.4
8.02E-03	1.45E-04	**-2.10**	38.8
8.25E-03	1.49E-04	**-2.08**	38.2
1.00E-02	1.80E-04	**-2.00**	38.3

Chauhan et al. 2013 **Graph (experimental points)**			
**C (M)**	**Molar fraction**	**logC (M)**	**σ (mN/m)**
1.01E-03	1.81E-05	**-3.00**	69.8
2.01E-03	3.62E-05	**-2.70**	63.1
3.01E-03	5.43E-05	**-2.52**	58.7
4.01E-03	7.22E-05	**-2.40**	54.2
4.98E-03	8.97E-05	**-2.30**	50.2
6.01E-03	1.08E-04	**-2.22**	46.1
7.05E-03	1.27E-04	**-2.15**	43.4
8.05E-03	1.45E-04	**-2.09**	41.3
8.94E-03	1.61E-04	**-2.05**	39.9
1.01E-02	1.83E-04	**-1.99**	39.9
1.10E-02	1.99E-04	**-1.96**	39.9
1.20E-02	2.17E-04	**-1.92**	39.9
1.30E-02	2.35E-04	**-1.88**	39.6
1.41E-02	2.54E-04	**-1.85**	39.6

Petkova et al. 2020 **Graph (experimental points)**		
**C (M)**	**Molar fraction**	**σ (mN/m)**
**4.28E-05**	7.70E-07	72.5
**5.98E-05**	1.08E-06	72.5
**1.00E-04**	1.80E-06	72.2
**1.21E-04**	2.18E-06	72.1
**2.11E-04**	3.80E-06	71.1
**3.27E-04**	5.88E-06	71.0
**4.22E-04**	7.60E-06	70.6
**6.21E-04**	1.12E-05	67.8
**1.04E-03**	1.87E-05	63.7
**1.24E-03**	2.24E-05	63.3
**1.95E-03**	3.52E-05	57.2
**3.39E-03**	6.12E-05	48.3
**3.96E-03**	7.14E-05	43.3
**4.22E-03**	7.61E-05	41.6
**9.87E-03**	1.78E-04	38.3
**1.67E-02**	3.02E-04	38.3

El Haber et al. 2023 **Table**		
**C (M)**	**Molar fraction**	**σ (mN/m)**
**0.00E+00**	0.00E+00	73.38
**4.10E-05**	7.38E-07	72.91
**8.30E-05**	1.49E-06	72.38
**2.70E-04**	4.86E-06	71.85
**5.10E-04**	9.18E-06	71.02
**7.70E-04**	1.39E-05	69.21
**9.90E-04**	1.78E-05	68.22
**2.80E-03**	5.04E-05	55.69
**7.00E-03**	1.26E-04	37.58
**8.90E-03**	1.61E-04	33.44
**1.10E-02**	1.99E-04	34.29
**2.10E-02**	3.80E-04	35.18
**4.90E-02**	8.94E-04	34.76
**7.80E-02**	1.43E-03	34.21
**9.60E-02**	1.77E-03	34.06

Recommended		
C (M)	***Molar fraction***	***σ (mN/m)***
1.00E-08	*1.80E-10*	*72.0*
2.33E-08	*4.20E-10*	*72.0*
5.45E-08	*9.80E-10*	*72.0*
1.27E-07	*2.29E-09*	*72.0*
2.97E-07	*5.34E-09*	*72.0*
6.92E-07	*1.25E-08*	*72.0*
1.62E-06	*2.91E-08*	*72.0*
3.77E-06	*6.78E-08*	*72.0*
8.80E-06	*1.58E-07*	*72.0*
2.05E-05	*3.69E-07*	*72.0*
4.79E-05	*8.62E-07*	*72.0*
1.12E-04	*2.01E-06*	*71.8*
2.61E-04	*4.70E-06*	*71.4*
6.09E-04	*1.10E-05*	*69.6*
1.42E-03	*2.56E-05*	*63.9*
3.31E-03	*5.97E-05*	*52.3*

**2.06E-02**	3.72E-04	38.1		*7.72E-03*	*1.39E-04*	*41.5*
**2.52E-02**	4.58E-04	38.1		*1.80E-02*	*3.25E-04*	*36.8*
**3.48E-02**	6.33E-04	37.6		*4.17E-02*	*7.58E-04*	*35.3*
**5.32E-02**	9.72E-04	37.6		*9.58E-02*	*1.77E-03*	*35.0*

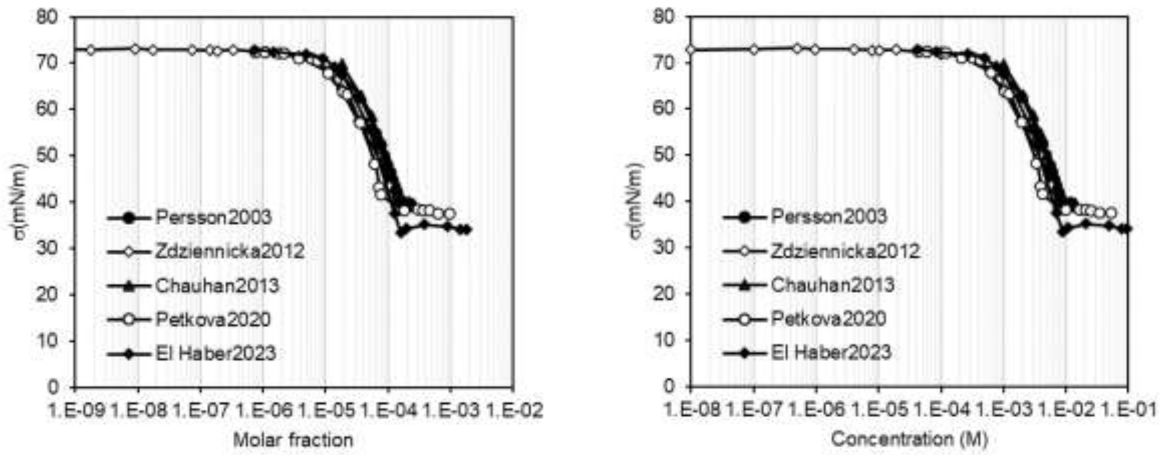

Fig. S117 (a): Experimental data for SDS / water mixtures.

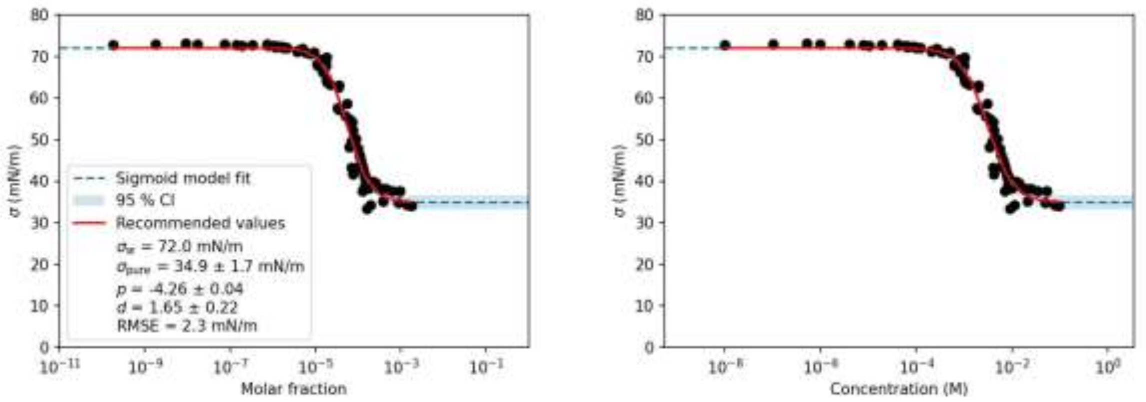

Fig. S117 (b): Surface tension fit with the Sigmoid model (*Kleinheins et al. 2023*) for SDS / water mixtures. Solid red line: model fit inside the concentration range covered by experimental data, reported as recommended values. Blue shading: fit parameters with 95 % confidence interval (CI). RMSE: root mean squared error. Markers: data used for fitting.

Comment:

Zdziennicka et al. 2012 : data reported at 19.85°C.

SB2: DTAB (dodecyltrimethylammonium bromide)

Asnacios et al. 1998 **Graph (experimental points)**		
**C (M)**	**Molar fraction**	**σ (mN/m)**
**1.50E-04**	2.70E-06	73.0
**3.09E-04**	5.57E-06	72.9
**5.78E-04**	1.04E-05	72.3
**1.17E-03**	2.11E-05	70.3
**2.46E-03**	4.43E-05	65.2
**4.69E-03**	8.45E-05	57.1
**9.48E-03**	1.71E-04	45.5
**9.66E-03**	1.74E-04	44.0
**1.15E-02**	2.08E-04	41.8
**1.30E-02**	2.34E-04	40.3
**1.37E-02**	2.48E-04	39.4
**1.84E-02**	3.33E-04	39.2
**2.52E-02**	4.57E-04	38.9
**3.95E-02**	7.19E-04	38.7

Ritacco et al. 2003 **Graph (experimental points)**		
**C (M)**	**Molar fraction**	**σ (mN/m)**
**4.94E-04**	8.89E-06	71.1
**9.70E-04**	1.75E-05	70.1
**1.94E-03**	3.50E-05	64.4
**2.93E-03**	5.28E-05	61.1
**3.98E-03**	7.18E-05	57.2
**4.98E-03**	8.97E-05	55.4
**6.07E-03**	1.09E-04	53.0
**6.93E-03**	1.25E-04	49.8
**7.98E-03**	1.44E-04	46.0
**8.89E-03**	1.60E-04	43.0
**1.12E-02**	2.02E-04	40.5
**1.47E-02**	2.66E-04	39.7
**1.96E-02**	3.55E-04	39.8
**3.01E-02**	5.47E-04	40.0
**4.05E-02**	7.38E-04	40.0

Shah et al. 2015 **Graph (experimental points)**			
**C (M)**	**Molar fraction**	**logC (M)**	**σ (mN/m)**
9.87E-03	1.78E-04	**-2.01**	43.4
1.07E-02	1.93E-04	**-1.97**	42.8
1.17E-02	2.12E-04	**-1.93**	42.0
1.27E-02	2.29E-04	**-1.90**	40.8
1.37E-02	2.48E-04	**-1.86**	39.6
1.49E-02	2.69E-04	**-1.83**	38.5
1.78E-02	3.22E-04	**-1.75**	37.7
2.10E-02	3.81E-04	**-1.68**	37.6
2.52E-02	4.57E-04	**-1.60**	37.5
3.00E-02	5.46E-04	**-1.52**	37.3
4.25E-02	7.74E-04	**-1.37**	37.0

Petkova et al. 2020 **Graph (experimental points)**			***Recommended***		
**C (M)**	**Molar fraction**	**σ (mN/m)**	***C (M)***	***Molar fraction***	***σ (mN/m)***
**5.00E-04**	9.00E-06	71.6	*1.50E-04*	*2.70E-06*	*72.0*
**1.00E-03**	1.80E-05	70.6	*2.04E-04*	*3.67E-06*	*72.0*
**2.50E-03**	4.50E-05	65.9	*2.77E-04*	*4.98E-06*	*71.9*
**5.00E-03**	9.01E-05	57.4	*3.76E-04*	*6.77E-06*	*71.8*
**1.00E-02**	1.81E-04	46.2	*5.11E-04*	*9.20E-06*	*71.7*
**2.50E-02**	4.53E-04	39.2	*6.94E-04*	*1.25E-05*	*71.4*
**5.00E-02**	9.13E-04	37.7	*9.43E-04*	*1.70E-05*	*70.9*
			1.28E-03	*2.31E-05*	*70.0*
			1.74E-03	*3.14E-05*	*68.5*
			2.36E-03	*4.26E-05*	*65.9*
			3.21E-03	*5.79E-05*	*62.0*
			4.36E-03	*7.86E-05*	*56.9*
			5.92E-03	*1.07E-04*	*51.4*
			8.04E-03	*1.45E-04*	*46.5*
			1.09E-02	*1.97E-04*	*42.7*
			1.48E-02	*2.68E-04*	*40.3*
			2.01E-02	*3.64E-04*	*38.8*
			2.73E-02	*4.95E-04*	*38.0*
			3.69E-02	*6.72E-04*	*37.5*
			5.00E-02	*9.13E-04*	*37.3*

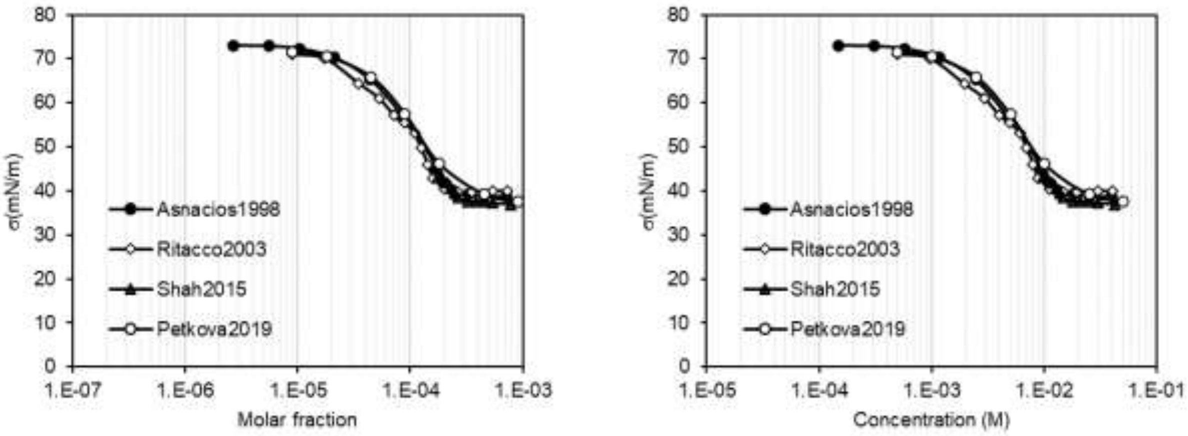

Fig. S118 (a): Experimental data for DTAB / water mixtures.

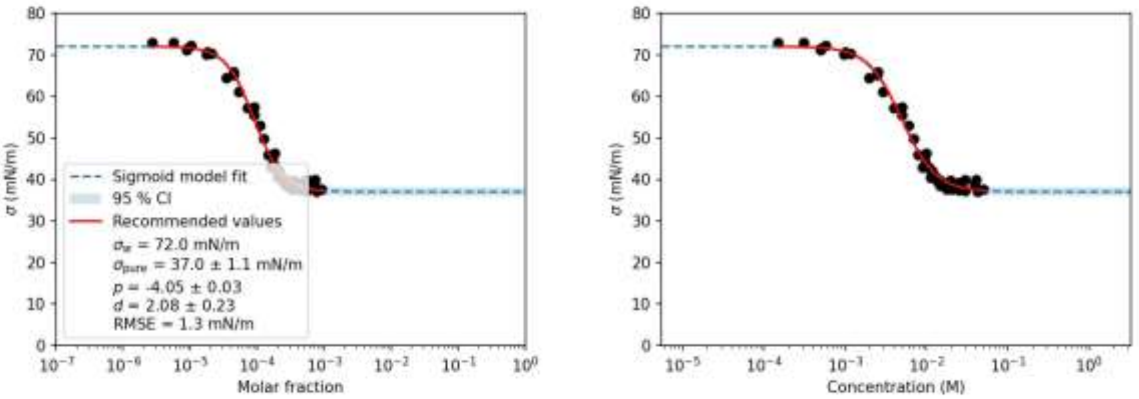

Fig. S118 (b): Surface tension fit with the Sigmoid model (*Kleinheins et al. 2023*) for DTAB / water mixtures. Solid red line: model fit inside the concentration range covered by experimental data, reported as recommended values. Blue shading: fit parameters with 95 % confidence interval (CI). RMSE: root mean squared error. Markers: data used for fitting.

Comment: N/A

SB3: CTAB (cetyltrimethylammonium bromide)

Adamczy et al. 1999 **Graph (experimental points)**

C (M)	Molar fraction	σ (mN/m)
**2.97E-06**	5.35E-08	72.0
**5.02E-06**	9.03E-08	71.3
**1.00E-05**	1.80E-07	70.6
**1.47E-05**	2.64E-07	70.0
**1.97E-05**	3.54E-07	69.3
**2.96E-05**	5.33E-07	67.3
**4.82E-05**	8.67E-07	66.2
**8.04E-05**	1.45E-06	64.0
**9.99E-05**	1.80E-06	63.2
**2.00E-04**	3.59E-06	56.8
**2.97E-04**	5.34E-06	53.5
**5.09E-04**	9.17E-06	46.2
**5.64E-04**	1.02E-05	44.6
**5.94E-04**	1.07E-05	44.1
**6.33E-04**	1.14E-05	43.1
**7.11E-04**	1.28E-05	40.8
**7.88E-04**	1.42E-05	39.2
**9.08E-04**	1.64E-05	36.6
**1.03E-03**	1.86E-05	36.5
**2.97E-06**	5.35E-08	72.0

Lundqvist et al. 2002 **Graph (experimental points)**

C (M)	Molar fraction	logC (M)	σ (mN/m)
9.75E-05	1.75E-06	**-4.01**	66.8
1.44E-04	2.59E-06	**-3.84**	63.4
2.08E-04	3.75E-06	**-3.68**	59.7
2.97E-04	5.34E-06	**-3.53**	55.4
4.23E-04	7.62E-06	**-3.37**	50.2
6.16E-04	1.11E-05	**-3.21**	44.3
8.84E-04	1.59E-05	**-3.05**	37.7
1.28E-03	2.30E-05	**-2.89**	37.2
2.76E-03	4.97E-05	**-2.56**	37.0
7.24E-03	1.31E-04	**-2.14**	36.3

Zdziennicka et al. 2012 **Graph (experimental points)**

C (M)	Molar fraction	logC (M)	σ (mN/m)
9.97E-09	1.79E-10	**-8.00**	72.8
9.65E-08	1.74E-09	**-7.02**	72.6
4.64E-07	8.35E-09	**-6.33**	72.0
9.62E-07	1.73E-08	**-6.02**	72.2
4.62E-06	8.32E-08	**-5.34**	71.6
9.06E-06	1.63E-07	**-5.04**	71.4
1.99E-05	3.58E-07	**-4.70**	70.8
3.11E-05	5.60E-07	**-4.51**	70.0
4.01E-05	7.21E-07	**-4.40**	69.6
5.77E-05	1.04E-06	**-4.24**	68.9
6.83E-05	1.23E-06	**-4.17**	68.7
8.55E-05	1.54E-06	**-4.07**	67.9
9.31E-05	1.68E-06	**-4.03**	67.3
9.58E-05	1.72E-06	**-4.02**	65.8
1.88E-04	3.39E-06	**-3.73**	60.8
3.04E-04	5.48E-06	**-3.52**	54.7
4.16E-04	7.48E-06	**-3.38**	50.2
4.93E-04	8.87E-06	**-3.31**	46.9
6.01E-04	1.08E-05	**-3.22**	43.8
7.11E-04	1.28E-05	**-3.15**	41.8
8.20E-04	1.48E-05	**-3.09**	37.9
1.06E-03	1.90E-05	**-2.98**	37.5
2.01E-03	3.63E-05	**-2.70**	37.5

Petkova et al. 2020 **Graph (experimental points)**

C (M)	Molar fraction	σ (mN/m)
**1.00E-04**	1.80E-06	70.5
**2.50E-04**	4.50E-06	68.2
**4.42E-04**	7.95E-06	64.0
**5.51E-04**	9.92E-06	59.7
**8.16E-04**	1.47E-05	54.0
**1.00E-03**	1.80E-05	52.5
**2.82E-03**	5.09E-05	43.2
**1.00E-02**	1.81E-04	41.6

Qazi et al. 2020 **Graph (experimental points)**

C (M)	Molar fraction	σ (mN/m)
**2.92E-05**	5.26E-07	67.3
**3.44E-05**	6.19E-07	67.2
**4.05E-05**	7.30E-07	67.0
**4.59E-05**	8.26E-07	66.8
**5.63E-05**	1.01E-06	66.6
**6.77E-05**	1.22E-06	66.1
**7.83E-05**	1.41E-06	65.4
**9.23E-05**	1.66E-06	64.4
**1.09E-04**	1.96E-06	63.1
**1.31E-04**	2.36E-06	61.5
**1.55E-04**	2.79E-06	59.9
**1.91E-04**	3.43E-06	57.8
**2.21E-04**	3.98E-06	55.9
**2.66E-04**	4.79E-06	53.9
**3.15E-04**	5.66E-06	51.8
**3.64E-04**	6.56E-06	49.4
**4.39E-04**	7.91E-06	47.1
**5.09E-04**	9.16E-06	44.7
**6.27E-04**	1.13E-05	42.2

Recommended

C (M)	*Molar fraction*	*σ (mN/m)*
9.94E-09	1.79E-10	72.0
2.02E-08	3.64E-10	72.0
4.12E-08	7.42E-10	72.0
8.39E-08	1.51E-09	72.0
1.71E-07	3.07E-09	72.0
3.47E-07	6.25E-09	72.0
7.07E-07	1.27E-08	72.0
1.44E-06	2.59E-08	72.0
2.93E-06	5.27E-08	71.9
5.96E-06	1.07E-07	71.8
1.21E-05	2.18E-07	71.5
2.47E-05	4.45E-07	70.8
5.03E-05	9.05E-07	68.9
1.02E-04	1.84E-06	64.6
2.08E-04	3.75E-06	57.0
4.24E-04	7.63E-06	47.7
8.63E-04	1.55E-05	40.5
1.76E-03	3.16E-05	36.6
3.57E-03	6.44E-05	34.9

	**7.26E-04**	1.31E-05	39.8	7.26E-03	1.31E-04	34.3
	**1.03E-03**	1.86E-05	37.3			
	**1.22E-03**	2.19E-05	37.3			
	**1.49E-03**	2.69E-05	37.0			
	**1.79E-03**	3.23E-05	37.0			
	**2.11E-03**	3.81E-05	36.9			
	**2.44E-03**	4.40E-05	36.9			
	**2.82E-03**	5.07E-05	36.8			
	**3.32E-03**	5.98E-05	36.8			
	**3.91E-03**	7.05E-05	36.6			

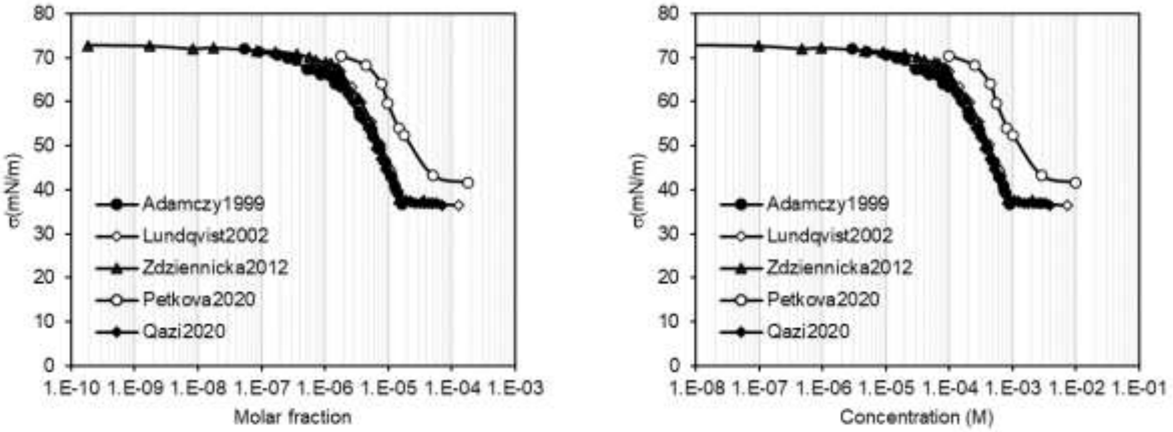

Fig. S119 (a): Experimental data for CTAB / water mixtures.

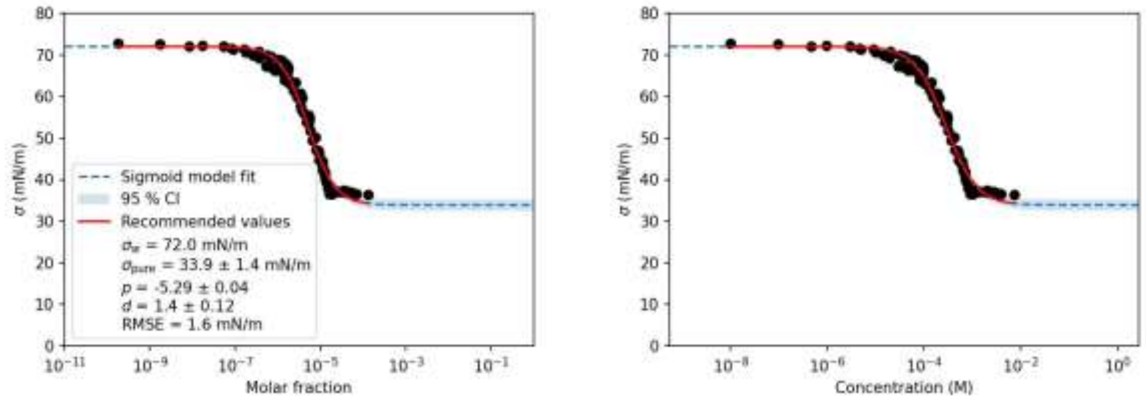

Fig. S119 (b): Surface tension fit with the Sigmoid model (*Kleinheins et al. 2023*) for CTAB / water mixtures. Solid red line: model fit inside the concentration range covered by experimental data, reported as recommended values. Blue shading: fit parameters with 95 % confidence interval (CI). RMSE: root mean squared error. Markers: data used for fitting.

Comment:

Lundqvist et al. 2002 : data reported at 27°C.

Zdziennicka et al. 2012: data reported at 19.85°C.

The data from *Petkova et al. 2020* were not included for the fitting, because they differ from the other surface tension isotherms.

Differences between the different references may come from the purity of the chemicals.

SB4: AOT (sodium bis(2-ethylhexyl)sulfosuccinate)

Pedrosa et al. 2002 **Graph (experimental points)**			Yuan et al. 2004 **Graph (experimental points)**			Xie et al. 2009 **Graph (experimental points)**		
**C (M)**	**Molar fraction**	**σ (mN/m)**	**C (M)**	**Molar fraction**	**σ (mN/m)**	**C (M)**	**Molar fraction**	**σ (mN/m)**
**1.54E-07**	2.77E-09	72.0	**1.86E-04**	3.34E-06	44.8	**1.77E-04**	3.19E-06	63.3
**5.47E-07**	9.85E-09	71.7	**4.26E-04**	7.67E-06	40.7	**2.26E-04**	4.06E-06	60.2
**1.07E-06**	1.93E-08	71.2	**7.00E-04**	1.26E-05	38.1	**3.16E-04**	5.70E-06	58.7
**1.68E-06**	3.02E-08	71.3	**1.06E-03**	1.91E-05	36.0	**4.09E-04**	7.36E-06	56.1
**3.24E-06**	5.83E-08	70.6	**1.50E-03**	2.71E-05	33.9	**4.55E-04**	8.19E-06	54.6
**1.47E-05**	2.65E-07	69.9	**2.05E-03**	3.69E-05	31.5	**5.91E-04**	1.06E-05	52.5
**3.46E-05**	6.24E-07	69.5	**2.68E-03**	4.83E-05	29.7	**6.83E-04**	1.23E-05	50.4
**6.18E-05**	1.11E-06	67.5	**3.42E-03**	6.16E-05	29.5	**8.63E-04**	1.55E-05	48.5
**8.61E-05**	1.55E-06	66.2	**4.19E-03**	7.56E-05	29.3	**1.04E-03**	1.88E-05	46.2
**1.26E-04**	2.27E-06	64.2	**5.40E-03**	9.74E-05	28.8	**1.40E-03**	2.52E-05	44.5
**1.94E-04**	3.50E-06	60.5	**6.56E-03**	1.18E-04	28.5	**1.67E-03**	3.01E-05	43.2
**2.50E-04**	4.50E-06	57.9	**7.98E-03**	1.44E-04	28.3	**2.12E-03**	3.82E-05	40.2
**3.06E-04**	5.51E-06	55.0	**9.62E-03**	1.74E-04	27.8	**2.66E-03**	4.79E-05	38.0
**3.28E-04**	5.90E-06	53.5	**1.14E-02**	2.06E-04	27.7	**3.32E-03**	5.99E-05	36.9
**4.35E-04**	7.83E-06	51.4	**1.25E-02**	2.26E-04	27.5	**4.17E-03**	7.52E-05	36.5
**5.59E-04**	1.01E-05	48.4	**1.54E-02**	2.80E-04	27.5	**5.24E-03**	9.45E-05	36.0
**7.80E-04**	1.41E-05	45.7	**1.74E-02**	3.16E-04	27.4	**6.48E-03**	1.17E-04	35.8
**9.38E-04**	1.69E-05	43.6	**1.99E-02**	3.60E-04	27.2	**8.22E-03**	1.48E-04	35.3
**1.25E-03**	2.25E-05	40.7	**2.42E-02**	4.39E-04	27.2	**1.04E-02**	1.87E-04	34.5
**1.25E-03**	2.25E-05	39.9	**2.67E-02**	4.85E-04	27.2	**1.28E-02**	2.32E-04	34.0
**1.31E-03**	2.37E-05	38.7	**2.99E-02**	5.44E-04	27.2	**1.60E-02**	2.90E-04	33.4
**1.66E-03**	2.99E-05	37.1	**3.49E-02**	6.36E-04	27.3	**2.00E-02**	3.63E-04	32.8
**1.84E-03**	3.31E-05	35.8				**2.50E-02**	4.54E-04	32.5
**2.48E-03**	4.47E-05	32.8						
**3.45E-03**	6.23E-05	31.2						
**5.14E-03**	9.26E-05	30.1						
**7.14E-03**	1.29E-04	29.7						
**8.28E-03**	1.50E-04	29.6						
**1.17E-02**	2.12E-04	29.5						

Krawzyk et al. 2015 **Graph (experimental points)**				Okamura et al. 2024 **Graph (experimental line)**			***Recommended***		
**C (M)**	**Molar fraction**	**logC (M)**	**σ (mN/m)**	**C (M)**	**Molar fraction**	**σ (mN/m)**	***C (M)***	***Molar fraction***	***σ (mN/m)***
1.00E-07	1.80E-09	**-7.00**	72.7	**5.00E-05**	9.00E-07	63.7	*1.00E-07*	*1.80E-09*	*72.0*
4.99E-07	8.99E-09	**-6.30**	72.2	**1.00E-04**	1.80E-06	54.8	*1.96E-07*	*3.53E-09*	*71.9*
1.00E-06	1.80E-08	**-6.00**	70.9	**3.00E-04**	5.40E-06	48.1	*3.84E-07*	*6.91E-09*	*71.9*
4.98E-06	8.96E-08	**-5.30**	67.5	**5.00E-04**	9.00E-06	45.2	*7.52E-07*	*1.35E-08*	*71.8*
1.00E-05	1.80E-07	**-5.00**	65.5	**1.00E-03**	1.80E-05	38.0	*1.47E-06*	*2.65E-08*	*71.6*
5.04E-05	9.07E-07	**-4.30**	57.0	**2.00E-03**	3.60E-05	31.8	*2.88E-06*	*5.19E-08*	*71.3*
1.00E-04	1.80E-06	**-4.00**	54.5	**2.30E-03**	4.14E-05	30.9	*5.65E-06*	*1.02E-07*	*70.8*
1.00E-03	1.80E-05	**-3.00**	39.9	**3.00E-03**	5.41E-05	30.1	*1.11E-05*	*1.99E-07*	*69.9*
5.01E-03	9.04E-05	**-2.30**	30.4	**4.00E-03**	7.21E-05	29.9	*2.17E-05*	*3.90E-07*	*68.5*
1.00E-02	1.81E-04	**-2.00**	29.2	**5.00E-03**	9.02E-05	29.2	*4.25E-05*	*7.64E-07*	*66.3*
				**9.00E-03**	1.63E-04	27.9	*8.32E-05*	*1.50E-06*	*62.9*
				**1.00E-02**	1.81E-04	26.8	*1.63E-04*	*2.93E-06*	*58.3*
				**1.20E-02**	2.17E-04	26.8	*3.19E-04*	*5.75E-06*	*52.4*

	**1.50E-02**	2.72E-04	26.4	*6.25E-04*	*1.13E-05*	*46.1*
	**1.80E-02**	3.26E-04	26.7	*1.22E-03*	*2.20E-05*	*40.2*
	**2.00E-02**	3.63E-04	26.1	*2.40E-03*	*4.32E-05*	*35.4*
	**2.50E-02**	4.54E-04	26.7	*4.69E-03*	*8.46E-05*	*31.8*
	**2.60E-02**	4.73E-04	26.1	*9.18E-03*	*1.66E-04*	*29.5*
				1.79E-02	*3.25E-04*	*28.0*
				3.49E-02	*6.36E-04*	*27.1*

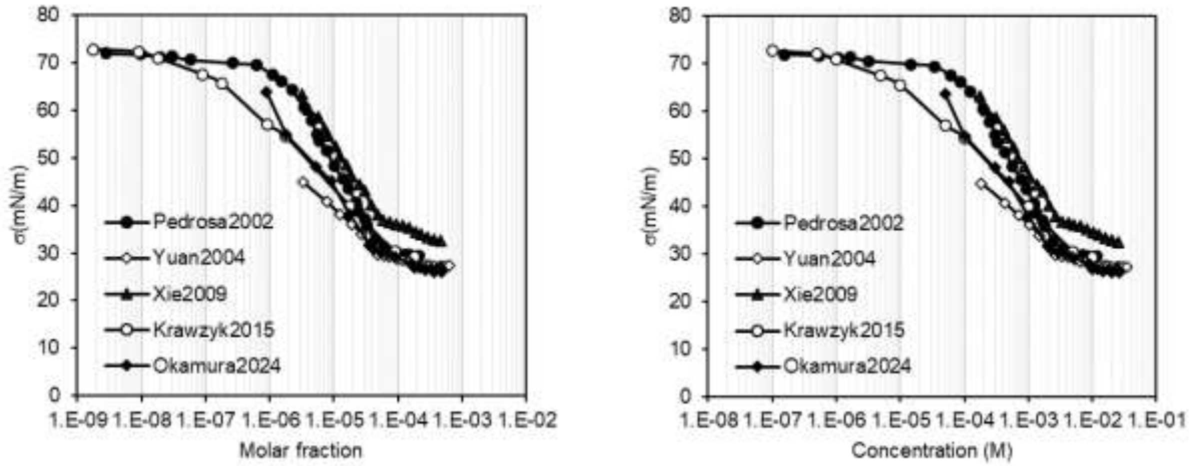

Fig. S120 (a): Experimental data for AOT / water mixtures.

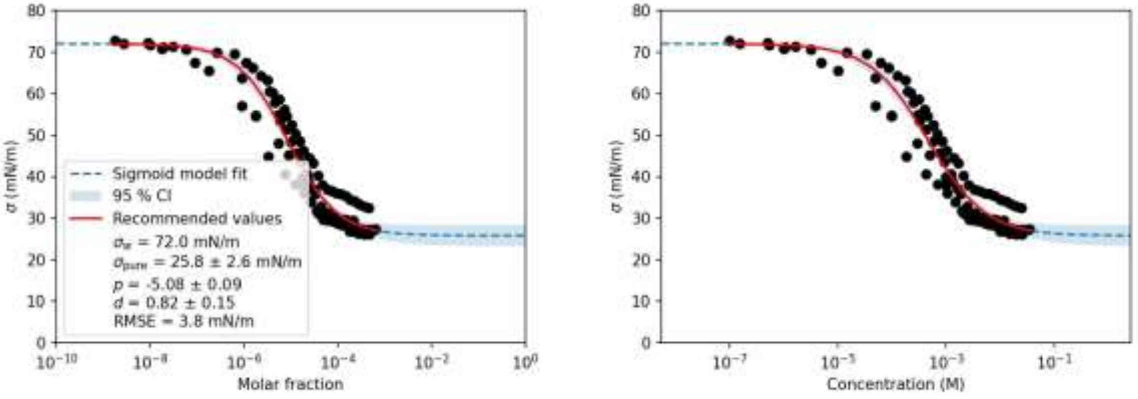

Fig. S120 (b): Surface tension fit with the Sigmoid model (*Kleinheins et al. 2023*) for AOT / water mixtures. Solid red line: model fit inside the concentration range covered by experimental data, reported as recommended values. Blue shading: fit parameters with 95 % confidence interval (CI). RMSE: root mean squared error. Markers: data used for fitting.

Comment:

Pedrosa et al. 2002, Yuan et al. 2004 and Xie et al. 2009 : data reported at 30°C.

SB5: Triton X114 ((1,1,3,3-tetramethylbutyl)phenyl-polyethylene glycol)

Singh et al. 1984 **Table**				Schulze et al. 1985 **Graph (experimental points)**			Sidim et al. 2012 **Graph (experimental points)**		
**C (M)**	**C (% v/v)**	**Molar fraction**	**σ (mN/m)**	**C (M)**	**Molar fraction**	**σ (mN/m)**	**C (M)**	**Molar fraction**	**σ (mN/m)**
0.00E+00	**0.00E+00**	0.00E+00	72.8	**1.00E-05**	1.80E-07	54.1	**5.00E-05**	9.00E-07	47.3
3.94E-08	**1.00E-05**	7.09E-10	67.4	**2.00E-05**	3.60E-07	49.4	**7.00E-05**	1.26E-06	37.6
3.94E-07	**1.00E-04**	7.09E-09	62.9	**3.00E-05**	5.40E-07	46.6	**2.00E-04**	3.60E-06	28.4
3.94E-06	**1.00E-03**	7.09E-08	48.2	**5.00E-05**	9.00E-07	43.3	**5.00E-04**	9.00E-06	28.1
3.94E-05	**1.00E-02**	7.09E-07	32.4	**1.00E-04**	1.80E-06	38.3	**1.00E-03**	1.80E-05	27.8
3.94E-04	**1.00E-01**	7.09E-06	30.3	**2.00E-04**	3.60E-06	33.8	**2.00E-03**	3.60E-05	28.0
4.93E-04	**1.25E-01**	8.87E-06	30.1	**3.00E-04**	5.40E-06	31.7	**4.00E-03**	7.21E-05	28.3
9.85E-04	**2.50E-01**	1.77E-05	29.5	**5.70E-04**	1.03E-05	31.3	**5.00E-03**	9.02E-05	28.4
1.97E-03	**5.00E-01**	3.55E-05	29.7	**8.10E-04**	1.46E-05	31.3			
3.94E-03	**1.00E+00**	7.11E-05	29.7	**1.20E-03**	2.16E-05	31.3			
7.88E-03	**2.00E+00**	1.42E-04	29.1	**1.70E-03**	3.06E-05	31.2			
				**5.80E-03**	1.05E-04	31.1			
				**1.20E-02**	2.17E-04	31.1			

Zdziennicka et al. 2012 **Graph (experimental points)**				Guo et al. 2022 **Graph (experimental points)**				***Recommended***		
**C (M)**	**logC (M)**	**Molar fraction**	**σ (mN/m)**	**C (M)**	**C(ppm)**	**Molar fraction**	**σ (mN/m)**	***C (M)***	***Molar fraction***	***σ (mN/m)***
1.00E-08	**-8.00**	1.81E-10	72.5	0.00E+00	**0.00E+00**	0.00E+00	72.7	*1.01E-08*	*1.81E-10*	*71.4*
1.91E-08	**-7.72**	3.44E-10	71.8	4.58E-05	**2.49E+01**	8.24E-07	42.4	*2.10E-08*	*3.78E-10*	*71.0*
2.08E-07	**-6.68**	3.74E-09	71.0	9.04E-05	**4.99E+01**	1.63E-06	34.6	*4.39E-08*	*7.90E-10*	*70.5*
5.39E-07	**-6.27**	9.70E-09	67.3	1.34E-04	**7.50E+01**	2.42E-06	32.4	*9.17E-08*	*1.65E-09*	*69.7*
1.08E-06	**-5.96**	1.95E-08	62.2	1.77E-04	**9.99E+01**	3.18E-06	30.2	*1.91E-07*	*3.45E-09*	*68.4*
2.12E-06	**-5.67**	3.82E-08	59.2	2.18E-04	**1.25E+02**	3.93E-06	29.3	*4.00E-07*	*7.20E-09*	*66.6*
5.05E-06	**-5.30**	9.09E-08	52.8	2.59E-04	**1.50E+02**	4.66E-06	28.9	*8.36E-07*	*1.50E-08*	*64.0*
1.08E-05	**-4.97**	1.94E-07	48.6	3.37E-04	**2.00E+02**	6.07E-06	29.0	*1.75E-06*	*3.14E-08*	*60.5*
2.05E-05	**-4.69**	3.68E-07	45.2					*3.65E-06*	*6.56E-08*	*56.2*
5.15E-05	**-4.29**	9.28E-07	40.2					*7.62E-06*	*1.37E-07*	*51.3*
1.01E-04	**-4.00**	1.82E-06	35.9					*1.59E-05*	*2.86E-07*	*46.2*
2.15E-04	**-3.67**	3.86E-06	31.6					*3.32E-05*	*5.98E-07*	*41.5*
4.98E-04	**-3.30**	8.97E-06	30.6					*6.94E-05*	*1.25E-06*	*37.5*
1.01E-03	**-3.00**	1.81E-05	30.7					*1.45E-04*	*2.61E-06*	*34.4*
1.92E-03	**-2.72**	3.45E-05	30.6					*3.03E-04*	*5.45E-06*	*32.2*
5.27E-03	**-2.28**	9.51E-05	30.8					*6.33E-04*	*1.14E-05*	*30.6*
								1.32E-03	*2.38E-05*	*29.6*
								2.76E-03	*4.97E-05*	*28.9*
								5.75E-03	*1.04E-04*	*28.4*
								1.20E-02	*2.17E-04*	*28.1*

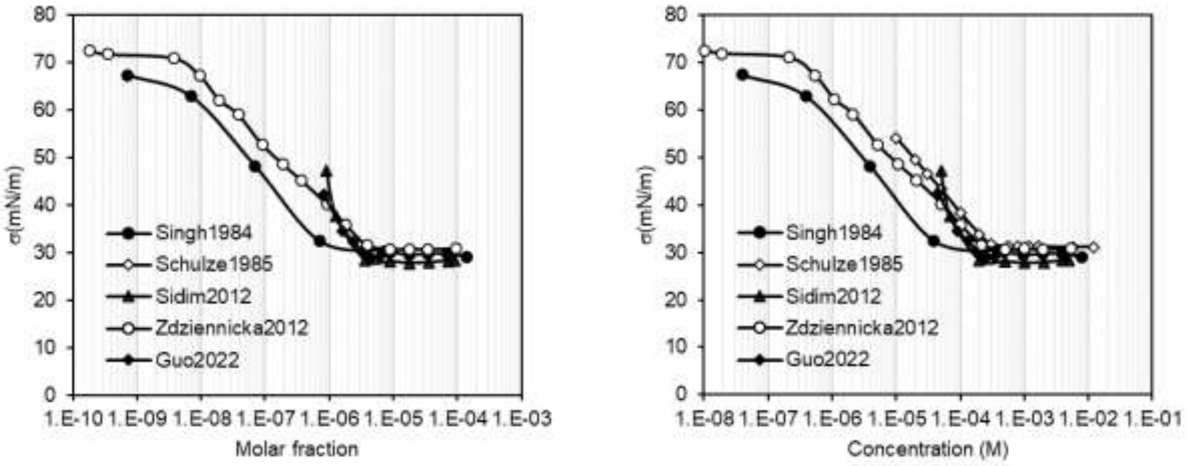

Fig. S121 (a): Experimental data for Triton X114 / water mixtures.

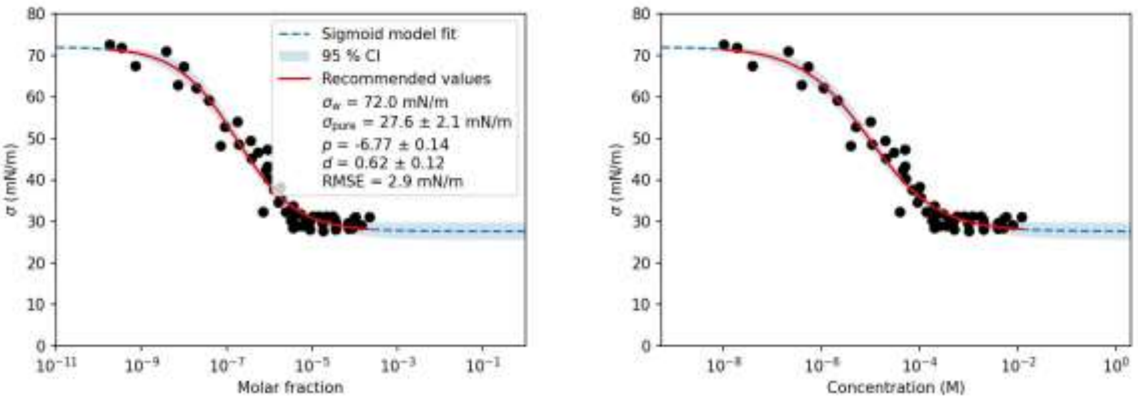

Fig. S121 (b): Surface tension fit with the Sigmoid model (*Kleinheins et al. 2023*) for Triton X114 / water mixtures. Solid red line: model fit inside the concentration range covered by experimental data, reported as recommended values. Blue shading: fit parameters with 95 % confidence interval (CI). RMSE: root mean squared error. Markers: data used for fitting.

Comment:

Zdziennicka et al. 2012 : data reported at 19.85°C.
Schulze et al. 1985 : data reported at 15°C.
Singh et al. 1984: Concentration (% v/v) with solution at 2% of surfactant
Differences between the different references may come from the purity of the chemicals.

SB6: Brij35 (polyoxyethylene lauryl ether)

Tripathi et al. 2007 **Graph (experimental averaged points)**				Pisaev et al. 2008 **Graph (experimental points)**			Krawzyk et al. 2015 **Graph (experimental points)**			
**C (M)**	**Molar fraction**	**C (mg/L)**	**σ (mN/m)**	**C (M)**	**Molar fraction**	**σ (mN/m)**	**C (M)**	**Molar fraction**	**logC (M)**	**σ (mN/m)**
9.81E-06	1.77E-07	**1.18E+01**	61.2	**1.42E-06**	2.55E-08	61.8	1.00E-07	1.80E-09	**-7.00**	71.2
1.24E-05	2.24E-07	**1.49E+01**	57.9	**3.86E-06**	6.95E-08	59.1	4.92E-07	8.85E-09	**-6.31**	67.2
2.38E-05	4.28E-07	**2.85E+01**	54.9	**7.87E-06**	1.42E-07	56.6	5.03E-06	9.05E-08	**-5.30**	57.6
2.89E-05	5.21E-07	**3.47E+01**	51.7	**1.45E-05**	2.62E-07	53.1	1.00E-05	1.80E-07	**-5.00**	54.2
3.62E-05	6.51E-07	**4.33E+01**	49.7	**3.15E-05**	5.68E-07	49.0	1.00E-04	1.80E-06	**-4.00**	43.4
3.77E-05	6.79E-07	**4.52E+01**	48.7	**4.64E-05**	8.36E-07	46.6	4.63E-04	8.34E-06	**-3.33**	43.4
5.11E-05	9.20E-07	**6.13E+01**	44.6	**6.85E-05**	1.23E-06	45.0	1.00E-03	1.80E-05	**-3.00**	43.7
6.04E-05	1.09E-06	**7.24E+01**	44.6	**1.01E-04**	1.82E-06	43.6	4.94E-03	8.95E-05	**-2.31**	43.6
6.70E-05	1.21E-06	**8.04E+01**	44.8	**1.35E-04**	2.43E-06	43.6	1.00E-02	1.82E-04	**-2.00**	43.5
7.53E-05	1.35E-06	**9.02E+01**	44.8	**2.00E-04**	3.60E-06	43.6				
8.30E-05	1.49E-06	**9.95E+01**	44.8							
1.12E-04	2.02E-06	**1.35E+02**	44.7							
1.68E-04	3.02E-06	**2.01E+02**	44.6							
1.90E-04	3.42E-06	**2.28E+02**	44.0							

Ozan et al. 2019 **Graph (experimental points)**				El Haber at al. 2023 **Table**			***Recommended***		
**C (M)**	**Molar fraction**	**logC (M)**	**σ (mN/m)**	**C (M)**	**Molar fraction**	**σ (mN/m)**	***C (M)***	***Molar fraction***	***σ (mN/m)***
6.62E-06	1.19E-07	**-5.18**	61.0	**0.00E+00**	0.00E+00	73.30	*1.00E-07*	*1.80E-09*	*71.5*
2.66E-05	4.80E-07	**-4.57**	54.5	**5.23E-06**	9.41E-08	71.88	*1.91E-07*	*3.43E-09*	*71.1*
3.29E-05	5.92E-07	**-4.48**	53.6	**2.44E-05**	4.39E-07	56.79	*3.63E-07*	*6.54E-09*	*70.5*
5.37E-05	9.66E-07	**-4.27**	51.8	**3.43E-05**	6.17E-07	53.73	*6.93E-07*	*1.25E-08*	*69.4*
6.52E-05	1.17E-06	**-4.19**	49.2	**4.59E-05**	8.26E-07	53.32	*1.32E-06*	*2.38E-08*	*67.8*
1.00E-04	1.80E-06	**-4.00**	46.5	**6.41E-05**	1.15E-06	50.40	*2.52E-06*	*4.53E-08*	*65.3*
1.33E-04	2.40E-06	**-3.88**	44.4	**8.62E-05**	1.55E-06	49.35	*4.80E-06*	*8.64E-08*	*62.0*
2.03E-04	3.65E-06	**-3.69**	43.7	**1.08E-04**	1.94E-06	47.80	*9.15E-06*	*1.65E-07*	*58.1*
2.60E-04	4.69E-06	**-3.58**	42.9	**2.00E-04**	3.60E-06	45.66	*1.75E-05*	*3.14E-07*	*54.1*
3.31E-04	5.97E-06	**-3.48**	42.7	**3.85E-04**	6.93E-06	45.66	*3.33E-05*	*5.99E-07*	*50.6*
				**5.59E-04**	1.01E-05	45.42	*6.34E-05*	*1.14E-06*	*47.9*
				**7.58E-04**	1.37E-05	44.87	*1.21E-04*	*2.18E-06*	*46.1*
				**1.03E-03**	1.86E-05	45.34	*2.31E-04*	*4.15E-06*	*44.9*
				**4.95E-03**	8.96E-05	45.13	*4.39E-04*	*7.91E-06*	*44.2*
				**7.76E-03**	1.41E-04	45.22	*8.37E-04*	*1.51E-05*	*43.7*
				**1.04E-02**	1.89E-04	45.30	*1.60E-03*	*2.88E-05*	*43.5*
				**2.06E-02**	3.80E-04	44.91	*3.04E-03*	*5.48E-05*	*43.3*
							5.77E-03	*1.05E-04*	*43.3*
							1.09E-02	*1.99E-04*	*43.2*
							2.06E-02	*3.80E-04*	*43.2*

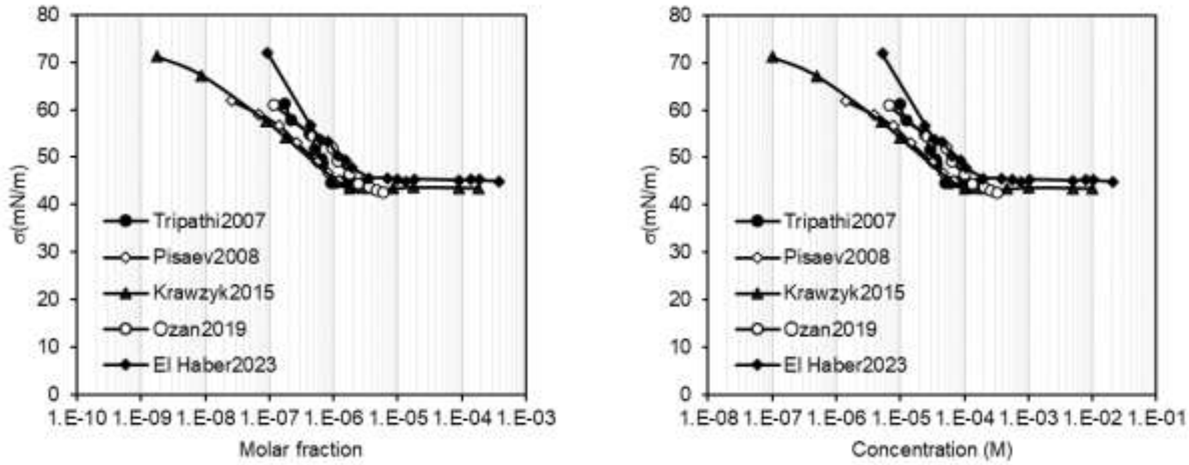

Fig. S122 (a): Experimental data for Brij35 / water mixtures.

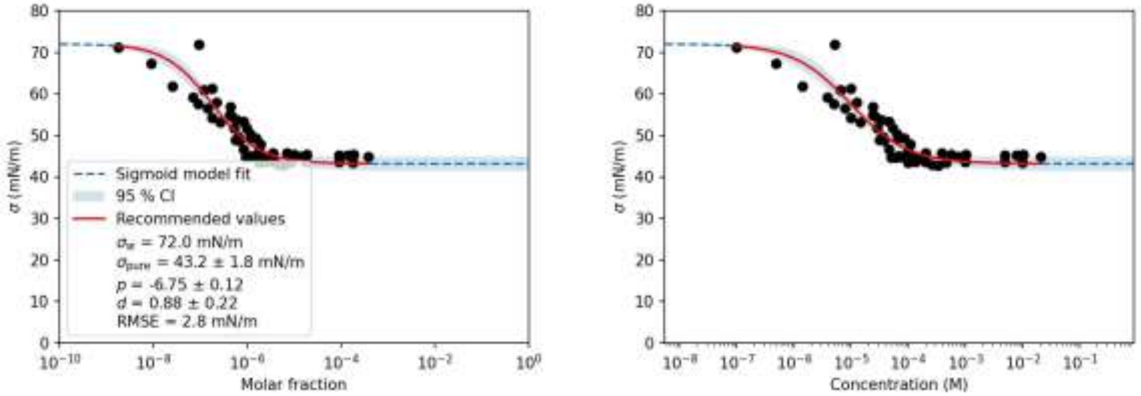

Fig. S122 (b): Surface tension fit with the Sigmoid model (*Kleinheins et al. 2023*) for Brij35 / water mixtures. Solid red line: model fit inside the concentration range covered by experimental data, reported as recommended values. Blue shading: fit parameters with 95 % confidence interval (CI). RMSE: root mean squared error. Markers: data used for fitting.

Comment: N/A

SB7: mono-rhamnolipid

Para et al. 1989 **Graph (experimental averaged points)**				Helvacı et al. 2004 **Graph (experimental points)**			Manko et al. 2014 **Graph (experimental points)**			
**C (M)**	**Molar fraction**	**Mass fraction (ppm)**	**σ (mN/m)**	**C (M)**	**Molar fraction**	**σ (mN/m)**	**C (M)**	**Molar fraction**	**logC (M)**	**σ (mN/m)**
1.87E-05	3.36E-07	**9.43E+00**	51.6	**1.00E-07**	1.80E-09	66.0	4.00E-10	7.20E-12	**-9.40**	71.7
2.24E-05	4.03E-07	**1.13E+01**	48.2	**2.01E-07**	3.61E-09	64.0	9.83E-10	1.77E-11	**-9.01**	71.7
3.82E-05	6.87E-07	**1.93E+01**	38.7	**3.01E-07**	5.41E-09	62.3	2.48E-09	4.46E-11	**-8.61**	71.6
4.80E-05	8.64E-07	**2.42E+01**	35.6	**1.01E-06**	1.81E-08	57.6	5.93E-09	1.07E-10	**-8.23**	71.7
5.88E-05	1.06E-06	**2.97E+01**	32.5	**3.01E-06**	5.41E-08	52.2	1.23E-08	2.22E-10	**-7.91**	71.6
7.39E-05	1.33E-06	**3.73E+01**	28.5	**7.02E-06**	1.26E-07	47.6	2.01E-08	3.62E-10	**-7.70**	71.5
9.20E-05	1.66E-06	**4.64E+01**	28.1	**1.01E-05**	1.81E-07	45.4	4.02E-08	7.23E-10	**-7.40**	71.3
1.14E-04	2.05E-06	**5.74E+01**	28.0	**2.01E-05**	3.61E-07	40.2	1.00E-07	1.80E-09	**-7.00**	70.8
2.23E-04	4.01E-06	**1.12E+02**	26.6	**3.01E-05**	5.41E-07	37.6	1.97E-07	3.55E-09	**-6.70**	70.0
2.76E-04	4.96E-06	**1.39E+02**	26.2	**4.00E-05**	7.21E-07	36.0	9.96E-07	1.79E-08	**-6.00**	64.5
3.49E-04	6.28E-06	**1.76E+02**	25.7	**5.01E-05**	9.02E-07	34.6	1.99E-06	3.59E-08	**-5.70**	59.5
4.36E-04	7.85E-06	**2.20E+02**	25.3	**7.06E-05**	1.27E-06	32.2	1.00E-05	1.81E-07	**-5.00**	44.5
				**1.00E-04**	1.80E-06	30.0	2.01E-05	3.62E-07	**-4.70**	37.4
							3.97E-05	7.14E-07	**-4.40**	30.9
							6.38E-05	1.15E-06	**-4.20**	28.4
							7.94E-05	1.43E-06	**-4.10**	27.9

Recommended		
C (M)	***Molar fraction***	***σ (mN/m)***
4.00E-10	*7.20E-12*	*71.8*
8.31E-10	*1.50E-11*	*71.7*
1.73E-09	*3.11E-11*	*71.6*
3.59E-09	*6.47E-11*	*71.4*
7.47E-09	*1.34E-10*	*71.1*
1.55E-08	*2.79E-10*	*70.7*
3.23E-08	*5.81E-10*	*70.1*
6.71E-08	*1.21E-09*	*69.2*
1.39E-07	*2.51E-09*	*67.9*
2.90E-07	*5.21E-09*	*66.0*
6.02E-07	*1.08E-08*	*63.5*
1.25E-06	*2.25E-08*	*60.1*
2.60E-06	*4.68E-08*	*55.8*
5.41E-06	*9.73E-08*	*50.7*
1.12E-05	*2.02E-07*	*45.2*
2.34E-05	*4.21E-07*	*39.6*
4.86E-05	*8.74E-07*	*34.4*
1.01E-04	*1.82E-06*	*29.8*
2.10E-04	*3.78E-06*	*26.2*
4.36E-04	*7.85E-06*	*23.4*

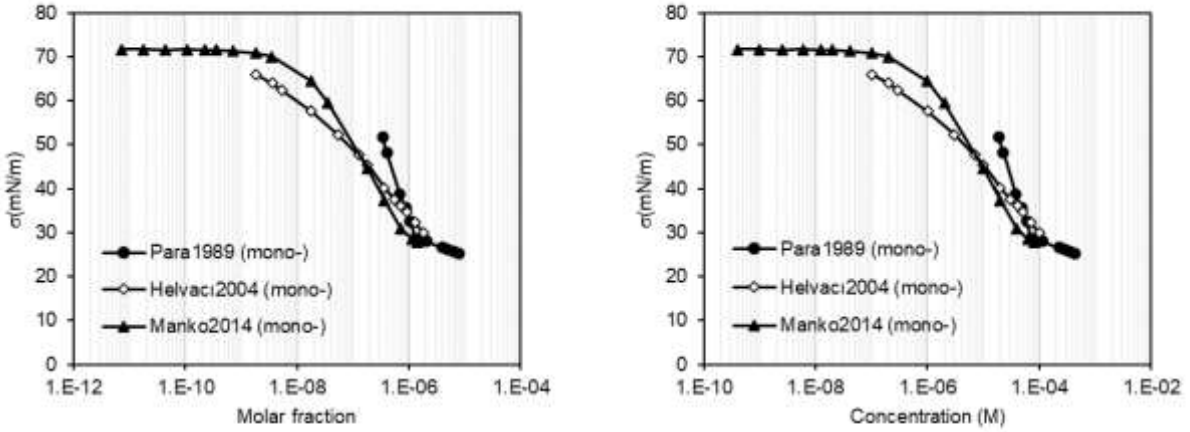

Fig. S123 (a): Experimental data for mono-rhamnolipid / water mixtures.

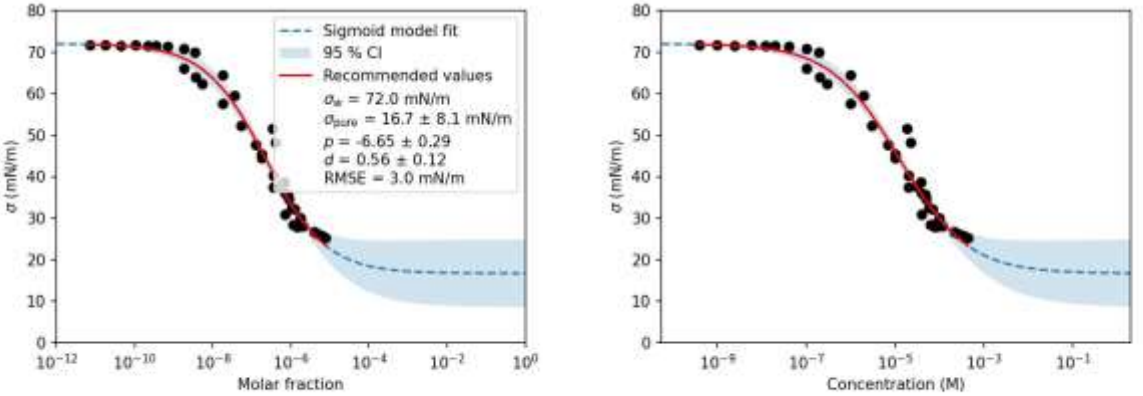

Fig. S123 (b): Surface tension fit with the Sigmoid model (*Kleinheins et al. 2023*) for mono-rhamnolipid / water mixtures. Solid red line: model fit inside the concentration range covered by experimental data, reported as recommended values. Blue shading: fit parameters with 95 % confidence interval (CI). RMSE: root mean squared error. Markers: data used for fitting.

Comment: N/A

SB8: di-rhamnolipid

Para et al. 1989 **Graph (experimental averaged points)**				Helvacı et al. 2004 **Graph (experimental points)**			Manko et al. 2014 **Graph (experimental points)**			
**C (M)**	**Molar fraction**	**Mass fraction (ppm)**	**σ (mN/m)**	**C (M)**	**Molar fraction**	**σ (mN/m)**	**C (M)**	**Molar fraction**	**logC (M)**	**σ (mN/m)**
6.18E-06	1.11E-07	**4.02E+00**	53.5	**1.00E-07**	1.80E-09	70.1	3.10E-10	5.57E-12	**-9.51**	71.7
9.79E-06	1.76E-07	**6.36E+00**	50.6	**2.00E-07**	3.60E-09	68.3	7.70E-10	1.39E-11	**-9.11**	71.7
1.23E-05	2.21E-07	**7.98E+00**	49.5	**3.00E-07**	5.39E-09	67.0	1.94E-09	3.49E-11	**-8.71**	71.7
1.60E-05	2.89E-07	**1.04E+01**	47.5	**9.97E-07**	1.79E-08	64.6	4.65E-09	8.37E-11	**-8.33**	71.6
1.94E-05	3.50E-07	**1.26E+01**	46.0	**3.00E-06**	5.41E-08	59.3	9.66E-09	1.74E-10	**-8.01**	71.6
2.45E-05	4.41E-07	**1.59E+01**	44.8	**6.99E-06**	1.26E-07	53.3	1.53E-08	2.76E-10	**-7.81**	71.5
3.09E-05	5.57E-07	**2.01E+01**	43.4	**1.00E-05**	1.80E-07	50.7	3.11E-08	5.60E-10	**-7.51**	71.3
3.80E-05	6.84E-07	**2.47E+01**	42.0	**2.00E-05**	3.60E-07	45.0	7.74E-08	1.39E-09	**-7.11**	70.9
4.77E-05	8.58E-07	**3.10E+01**	39.9	**3.00E-05**	5.39E-07	42.0	1.57E-07	2.82E-09	**-6.80**	70.1
5.98E-05	1.08E-06	**3.88E+01**	38.9	**3.99E-05**	7.19E-07	40.0	7.70E-07	1.39E-08	**-6.11**	64.5
7.54E-05	1.36E-06	**4.90E+01**	36.9	**4.99E-05**	8.98E-07	38.1	1.54E-06	2.77E-08	**-5.81**	59.5
9.59E-05	1.73E-06	**6.23E+01**	35.4	**7.01E-05**	1.26E-06	34.5	7.67E-06	1.38E-07	**-5.12**	44.5
1.20E-04	2.16E-06	**7.81E+01**	34.1	**1.00E-04**	1.81E-06	32.0	1.55E-05	2.80E-07	**-4.81**	37.4
1.49E-04	2.68E-06	**9.66E+01**	32.8				3.07E-05	5.52E-07	**-4.51**	30.9
1.81E-04	3.26E-06	**1.18E+02**	32.1				4.94E-05	8.88E-07	**-4.31**	28.5
2.23E-04	4.01E-06	**1.45E+02**	30.7				6.22E-05	1.12E-06	**-4.21**	27.9
2.89E-04	5.20E-06	**1.88E+02**	29.7							
3.57E-04	6.43E-06	**2.32E+02**	29.2							
4.57E-04	8.24E-06	**2.97E+02**	29.0							
5.81E-04	1.05E-05	**3.78E+02**	28.8							

Recommended		
C (M)	***Molar fraction***	***σ (mN/m)***
3.09E-10	*5.57E-12*	*72.0*
6.62E-10	*1.19E-11*	*71.9*
1.42E-09	*2.55E-11*	*71.9*
3.03E-09	*5.45E-11*	*71.8*
6.48E-09	*1.17E-10*	*71.7*
1.39E-08	*2.50E-10*	*71.5*
2.97E-08	*5.34E-10*	*71.1*
6.35E-08	*1.14E-09*	*70.6*
1.36E-07	*2.44E-09*	*69.6*
2.90E-07	*5.23E-09*	*68.1*
6.21E-07	*1.12E-08*	*65.9*
1.33E-06	*2.39E-08*	*62.5*
2.84E-06	*5.12E-08*	*58.0*
6.08E-06	*1.10E-07*	*52.5*
1.30E-05	*2.34E-07*	*46.5*
2.78E-05	*5.01E-07*	*40.8*
5.96E-05	*1.07E-06*	*36.1*
1.27E-04	*2.29E-06*	*32.5*
2.73E-04	*4.91E-06*	*30.1*
5.83E-04	*1.05E-05*	*28.4*

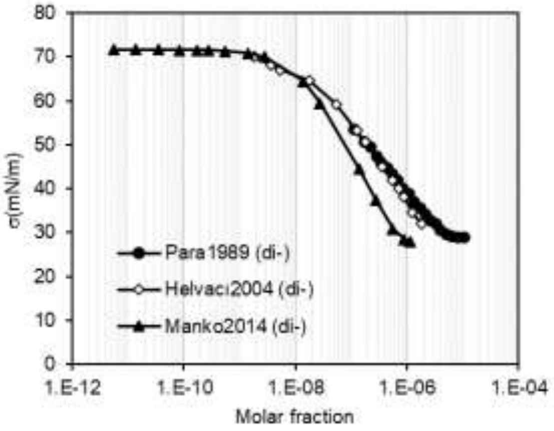

Fig. S124 (a): Experimental data for di-rhamnolipid / water mixtures.

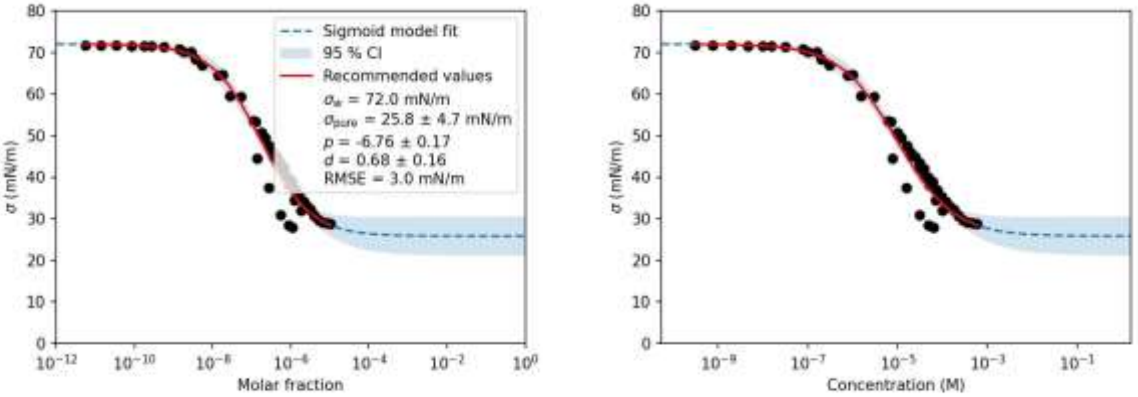

Fig. S124 (b): Surface tension fit with the Sigmoid model (*Kleinheins et al. 2023*) for di-rhamnolipid / water mixtures. Solid red line: model fit inside the concentration range covered by experimental data, reported as recommended values. Blue shading: fit parameters with 95 % confidence interval (CI). RMSE: root mean squared error. Markers: data used for fitting.

Comment: N/A

SB9: surfactin *from Bacillus subtilis*

Abdel-Mawgoud et al. 2004 **Graph (experimental points)**				Razafindralambo et al. 2004 **Graph (experimental points)**			Ekström et al. 2011 **Graph (experimental points)**		
**C (M)**	**Molar fraction**	**C (mg/L)**	**σ (mN/m)**	**C (M)**	**Molar fraction**	**σ (mN/m)**	**C (M)**	**Molar fraction**	**σ (mN/m)**
7.97E-07	1.44E-08	**8.26E-01**	68.3	**4.86E-06**	8.76E-08	43.5	**5.19E-07**	9.33E-09	70.3
1.99E-06	3.59E-08	**2.07E+00**	68.1	**7.76E-06**	1.40E-07	39.2	**1.04E-06**	1.88E-08	70.4
2.69E-06	4.84E-08	**2.79E+00**	67.5	**9.73E-06**	1.75E-07	37.6	**3.17E-06**	5.71E-08	68.5
3.89E-06	7.00E-08	**4.03E+00**	67.7	**1.45E-05**	2.61E-07	34.0	**9.50E-06**	1.71E-07	40.2
5.78E-06	1.04E-07	**5.99E+00**	62.2	**1.91E-05**	3.44E-07	31.9	**2.85E-05**	5.12E-07	29.0
7.67E-06	1.38E-07	**7.95E+00**	56.6	**2.91E-05**	5.24E-07	32.1			
9.57E-06	1.72E-07	**9.91E+00**	49.9	**3.84E-05**	6.92E-07	31.1			
1.16E-05	2.08E-07	**1.20E+01**	44.7						
1.35E-05	2.42E-07	**1.39E+01**	39.3						
1.53E-05	2.76E-07	**1.59E+01**	38.5						
1.73E-05	3.12E-07	**1.80E+01**	37.4						
1.92E-05	3.46E-07	**1.99E+01**	37.3						
2.12E-05	3.82E-07	**2.20E+01**	37.3						
2.31E-05	4.16E-07	**2.40E+01**	37.1						
2.51E-05	4.52E-07	**2.60E+01**	36.8						
2.89E-05	5.20E-07	**2.99E+01**	36.2						
4.82E-05	8.68E-07	**5.00E+01**	34.1						
7.23E-05	1.30E-06	**7.50E+01**	33.3						
9.66E-05	1.74E-06	**1.00E+02**	32.5						
1.21E-04	2.17E-06	**1.25E+02**	32.3						
1.45E-04	2.60E-06	**1.50E+02**	32.0						
1.69E-04	3.04E-06	**1.75E+02**	31.9						

Long et al. 2017 **Graph (experimental points)**				***Recommended***		
**C (M)**	**Molar fraction**	**C (mg/L)**	**σ (mN/m)**	***C (M)***	***Molar fraction***	***σ (mN/m)***
0.00E+00	0.00E+00	**0.00E+00**	72.6	*4.82E-07*	*8.68E-09*	*68.9*
4.82E-07	8.68E-09	**5.00E-01**	68.6	*6.56E-07*	*1.18E-08*	*67.6*
7.72E-07	1.39E-08	**8.00E-01**	64.5	*8.93E-07*	*1.61E-08*	*65.8*
9.65E-07	1.74E-08	**1.00E+00**	61.9	*1.22E-06*	*2.19E-08*	*63.4*
1.45E-06	2.61E-08	**1.50E+00**	53.2	*1.66E-06*	*2.98E-08*	*60.5*
1.93E-06	3.47E-08	**2.00E+00**	45.1	*2.25E-06*	*4.06E-08*	*56.9*
2.89E-06	5.21E-08	**3.00E+00**	36.5	*3.07E-06*	*5.52E-08*	*53.0*
3.86E-06	6.95E-08	**4.00E+00**	32.8	*4.17E-06*	*7.51E-08*	*48.9*
5.79E-06	1.04E-07	**6.00E+00**	29.5	*5.68E-06*	*1.02E-07*	*45.0*
7.72E-06	1.39E-07	**8.00E+00**	27.9	*7.74E-06*	*1.39E-07*	*41.5*
9.65E-06	1.74E-07	**1.00E+01**	27.2	*1.05E-05*	*1.90E-07*	*38.6*
1.45E-05	2.61E-07	**1.50E+01**	27.1	*1.43E-05*	*2.58E-07*	*36.3*
1.93E-05	3.47E-07	**2.00E+01**	26.9	*1.95E-05*	*3.51E-07*	*34.6*
2.89E-05	5.21E-07	**3.00E+01**	26.6	*2.66E-05*	*4.78E-07*	*33.3*
4.63E-05	8.34E-07	**4.80E+01**	26.6	*3.61E-05*	*6.51E-07*	*32.3*
				4.92E-05	*8.86E-07*	*31.7*
				6.70E-05	*1.21E-06*	*31.2*
				9.11E-05	*1.64E-06*	*30.9*
				1.24E-04	*2.23E-06*	*30.7*
				1.69E-04	*3.04E-06*	*30.6*

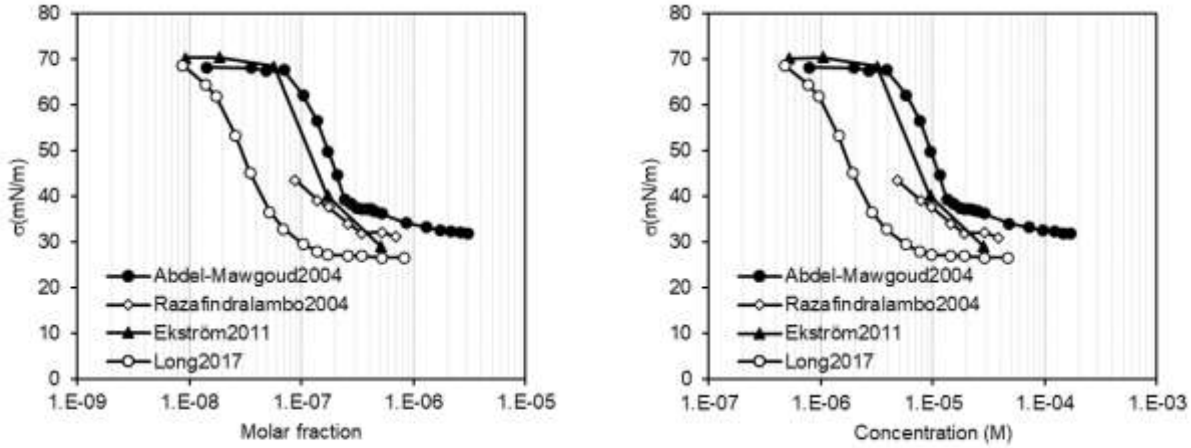

Fig. S125 (a): Experimental data for surfactin / water mixtures.

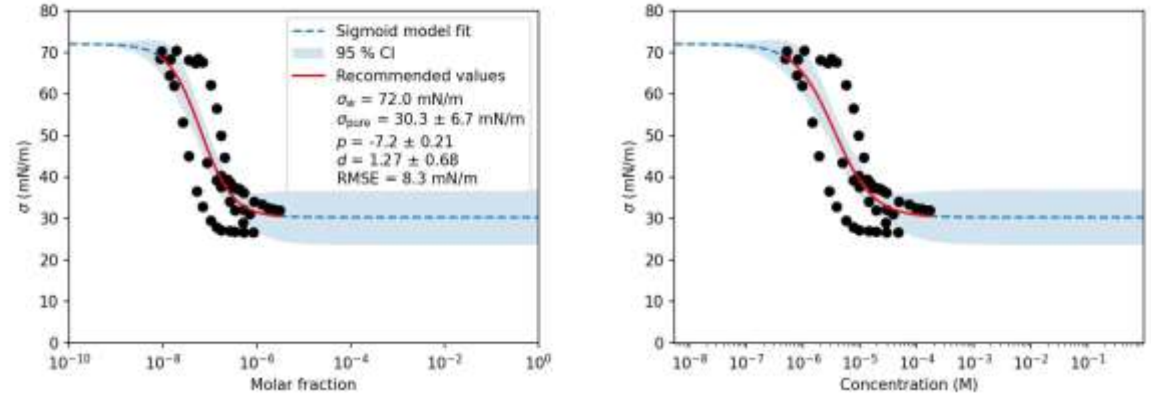

Fig. S125 (b): Surface tension fit with the Sigmoid model (*Kleinheins et al. 2023*) for surfactin / water mixtures. Solid red line: model fit inside the concentration range covered by experimental data, reported as recommended values. Blue shading: fit parameters with 95 % confidence interval (CI). RMSE: root mean squared error. Markers: data used for fitting.

Comment:

Differences between the different references may come from the purity of the compounds.

SB10: syringafactin B/C *from Xanthomonas and Pseudomonas*

Renard et al. 2019	**Graph (experimental points)**		
**C (M)**	**Molar fraction**	**C (mg/L)**	**σ (mN/m)**
1.38E-06	2.48E-08	**1.51E+00**	70.7
4.71E-06	8.48E-08	**5.16E+00**	70.5
1.19E-05	2.14E-07	**1.30E+01**	70.1
2.96E-05	5.32E-07	**3.24E+01**	66.5
7.59E-05	1.37E-06	**8.31E+01**	49.0
2.57E-04	4.62E-06	**2.81E+02**	34.9
6.43E-04	1.16E-05	**7.04E+02**	25.0
4.06E-03	7.35E-05	**4.45E+03**	25.0
1.38E-06	2.48E-08	**1.51E+00**	70.7
4.71E-06	8.48E-08	**5.16E+00**	70.5
1.19E-05	2.14E-07	**1.30E+01**	70.1
2.96E-05	5.32E-07	**3.24E+01**	66.5

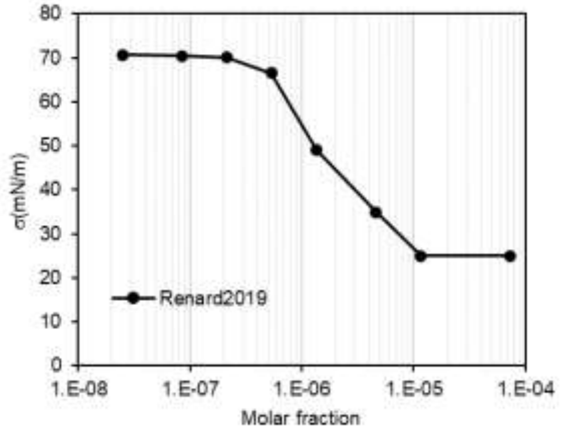

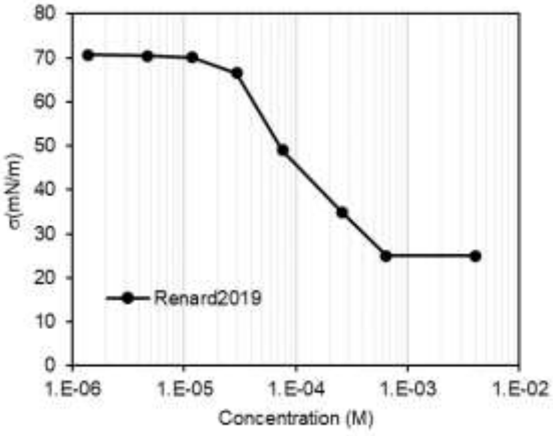

Fig. S126: Experimental data for syringafactin B/C / water mixtures.

Comment:

These concentrations are calculated assuming a density of 1.

SB11: viscosin *from Pseudomonas*

De Bruijn et al. 2007 **Graph (experimental points)**				Saini et al 2008 **Graph (experimental points)**			
**C (M)**	**Molar fraction**	**C (mg/L)**	**σ (mN/m)**	**C (M)**	**Molar fraction**	**C (mg/L)**	**σ (mN/m)**
8.88E-07	1.60E-08	**1.00E+00**	73.0	1.78E-06	3.20E-08	**2.00E+00**	44.6
1.79E-06	3.21E-08	**2.01E+00**	72.3	2.61E-06	4.70E-08	**2.94E+00**	42.4
4.46E-06	8.03E-08	**5.02E+00**	67.2	4.36E-06	7.85E-08	**4.91E+00**	36.9
8.90E-06	1.60E-07	**1.00E+01**	39.6	6.54E-06	1.18E-07	**7.36E+00**	36.4
1.11E-05	1.99E-07	**1.25E+01**	38.3	8.71E-06	1.57E-07	**9.80E+00**	32.8
2.22E-05	4.00E-07	**2.50E+01**	34.5	1.75E-05	3.15E-07	**1.97E+01**	32.2
4.44E-05	7.99E-07	**5.00E+01**	32.4	2.63E-05	4.74E-07	**2.96E+01**	29.7
8.87E-05	1.60E-06	**9.98E+01**	31.5	3.52E-05	6.34E-07	**3.97E+01**	29.9
8.88E-04	1.60E-05	**1.00E+03**	30.6	4.40E-05	7.92E-07	**4.95E+01**	28.6
				6.62E-05	1.19E-06	**7.45E+01**	28.1
				8.81E-05	1.59E-06	**9.92E+01**	27.9
				1.33E-04	2.40E-06	**1.50E+02**	27.8
				1.78E-04	3.20E-06	**2.00E+02**	27.6

Renard et al 2019 **Graph (experimental points)**				***Recommended***		
**C (M)**	**Molar fraction**	**C (mg/L)**	**σ (mN/m)**	***C (M)***	***Molar fraction***	***σ (mN/m)***
1.94E-06	3.49E-08	**2.18E+00**	71.5	*8.89E-07*	*1.60E-08*	*69.6*
2.67E-06	4.81E-08	**3.01E+00**	70.8	*1.32E-06*	*2.37E-08*	*67.8*
3.10E-06	5.58E-08	**3.49E+00**	70.3	*1.95E-06*	*3.51E-08*	*65.0*
3.89E-06	7.00E-08	**4.38E+00**	69.9	*2.88E-06*	*5.19E-08*	*60.7*
7.79E-06	1.40E-07	**8.77E+00**	57.9	*4.27E-06*	*7.68E-08*	*55.1*
1.02E-05	1.83E-07	**1.14E+01**	51.0	*6.32E-06*	*1.14E-07*	*48.5*
1.25E-05	2.24E-07	**1.40E+01**	39.9	*9.35E-06*	*1.68E-07*	*42.1*
1.55E-05	2.79E-07	**1.75E+01**	32.9	*1.38E-05*	*2.49E-07*	*36.8*
2.22E-05	4.00E-07	**2.50E+01**	30.9	*2.05E-05*	*3.69E-07*	*33.0*
3.87E-05	6.96E-07	**4.35E+01**	27.4	*3.03E-05*	*5.46E-07*	*30.5*
7.75E-05	1.40E-06	**8.72E+01**	26.9	*4.49E-05*	*8.09E-07*	*29.0*
1.54E-04	2.77E-06	**1.73E+02**	26.4	*6.65E-05*	*1.20E-06*	*28.1*
7.70E-04	1.39E-05	**8.67E+02**	25.3	*9.84E-05*	*1.77E-06*	*27.6*
1.53E-03	2.76E-05	**1.72E+03**	24.0	*1.46E-04*	*2.62E-06*	*27.3*
				2.16E-04	*3.88E-06*	*27.2*
				3.19E-04	*5.75E-06*	*27.1*
				4.72E-04	*8.51E-06*	*27.1*
				6.99E-04	*1.26E-05*	*27.0*
				1.03E-03	*1.86E-05*	*27.0*
				1.53E-03	*2.76E-05*	*27.0*

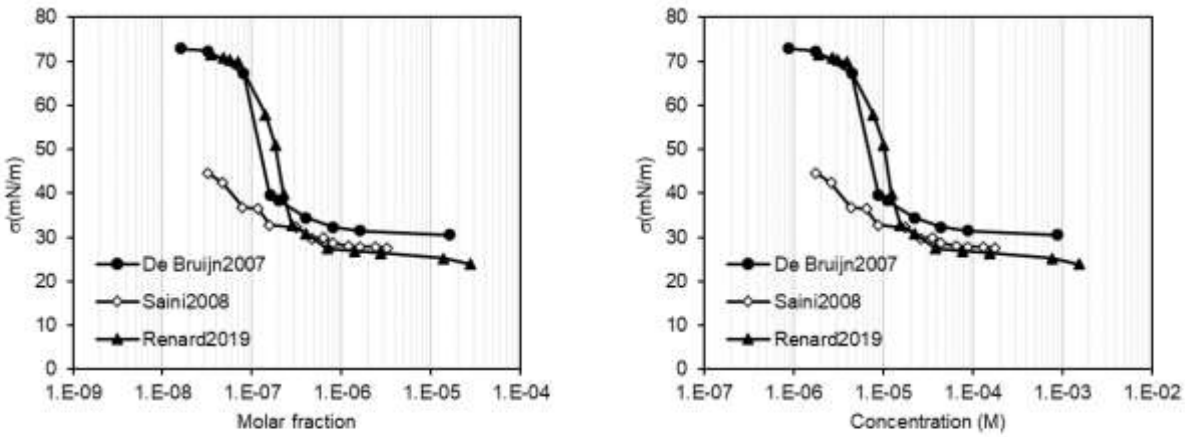

Fig. S127 (a): Experimental data for viscosin / water mixtures.

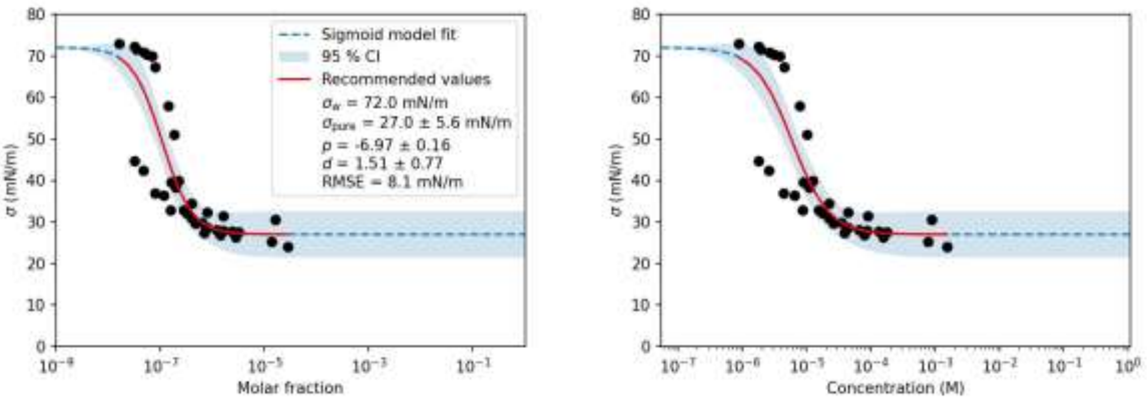

Fig. S127 (b): Surface tension fit with the Sigmoid model (*Kleinheins et al. 2023*) for viscosin / water mixtures. Solid red line: model fit inside the concentration range covered by experimental data, reported as recommended values. Blue shading: fit parameters with 95 % confidence interval (CI). RMSE: root mean squared error. Markers: data used for fitting.

Comment:

Differences between the different references may come from the purity of the compounds.

S3.5 Macromolecules

MA1: SRFA (Suwannee river fulvic acid)

Kiss et al. 2005 **Graph (experimental points)**			
**C (M)**	**Molar fraction**	**C (g/L)**	**σ (mN/m)**
0.00E+00	0.00E+00	**0.00E+00**	71.7
1.31E-04	2.37E-06	**7.49E-02**	70.6
2.61E-04	4.69E-06	**1.49E-01**	69.5
5.26E-04	9.47E-06	**3.00E-01**	68.7
1.05E-03	1.89E-05	**5.98E-01**	66.6
1.69E-03	3.04E-05	**9.63E-01**	65.6
2.10E-03	3.79E-05	**1.20E+00**	64.6

Svenningsson et al. 2006 **Graph (experimental points)**			
**C (M)**	**Molar fraction**	**Molality (mol/kg)**	**σ (mN/m)**
7.96E-03	1.44E-04	**8.00E-03**	70.8
4.42E-02	8.08E-04	**4.50E-02**	67.1
1.01E-01	1.88E-03	**1.05E-01**	60.6
3.59E-01	7.42E-03	**4.16E-01**	52.0

Taraniuk et al. 2007 **Graph (experimental points)**			
**C (M)**	**Molar fraction**	**ln (C mol/m^3)**	**σ (mN/m)**
2.73E-04	4.91E-06	**-1.30E+00**	66.1
9.94E-04	1.79E-05	**-5.71E-03**	63.9
1.75E-03	3.15E-05	**5.58E-01**	54.8
1.97E-03	3.55E-05	**6.79E-01**	59.4
5.90E-03	1.06E-04	**1.78E+00**	57.0
8.77E-03	1.58E-04	**2.17E+00**	49.3
1.18E-02	2.13E-04	**2.47E+00**	47.9
1.75E-02	3.18E-04	**2.86E+00**	47.8
2.19E-02	3.97E-04	**3.08E+00**	45.0
3.50E-02	6.39E-04	**3.56E+00**	40.4
4.37E-02	7.99E-04	**3.78E+00**	43.9
8.80E-02	1.64E-03	**4.48E+00**	39.6
1.75E-01	3.36E-03	**5.16E+00**	38.2

Topping et al. 2007 **Graph (experimental points)**			
**C (M)**	**Molar fraction**	**Mass fraction**	**σ (mN/m)**
3.31E-05	5.96E-07	**1.89E-05**	71.0
3.28E-04	5.90E-06	**1.87E-04**	71.0
1.65E-03	2.98E-05	**9.41E-04**	67.0
4.00E-03	7.21E-05	**2.28E-03**	60.7
1.61E-02	2.92E-04	**9.15E-03**	51.9

Aumann et al. 2010 **Graph (experimental points)**			
**C (M)**	**Molar fraction**	**C (g/L)**	**σ (mN/m)**
8.77E-06	1.58E-07	**5.00E-03**	71.7
4.39E-05	7.89E-07	**2.50E-02**	71.6
8.77E-05	1.58E-06	**5.00E-02**	71.1
1.75E-04	3.16E-06	**1.00E-01**	70.4
3.51E-04	6.32E-06	**2.00E-01**	68.5
8.77E-04	1.58E-05	**5.00E-01**	63.4
1.75E-03	3.16E-05	**1.00E+00**	59.6
5.26E-03	9.49E-05	**3.00E+00**	52.2
8.77E-03	1.58E-04	**5.00E+00**	49.5
1.75E-02	3.18E-04	**1.00E+01**	46.6
3.51E-02	6.40E-04	**2.00E+01**	44.7

Recommended		
C (M)	***Molar fraction***	***σ (mN/m)***
8.78E-06	*1.58E-07*	*71.8*
1.48E-05	*2.67E-07*	*71.7*
2.51E-05	*4.51E-07*	*71.5*
4.23E-05	*7.62E-07*	*71.3*
7.15E-05	*1.29E-06*	*70.9*
1.21E-04	*2.18E-06*	*70.4*
2.04E-04	*3.68E-06*	*69.7*
3.45E-04	*6.21E-06*	*68.6*
5.83E-04	*1.05E-05*	*67.1*
9.84E-04	*1.77E-05*	*65.1*
1.66E-03	*2.99E-05*	*62.5*
2.81E-03	*5.06E-05*	*59.4*
4.74E-03	*8.55E-05*	*55.8*
8.00E-03	*1.44E-04*	*52.2*
1.35E-02	*2.44E-04*	*48.6*
2.27E-02	*4.12E-04*	*45.5*
3.82E-02	*6.97E-04*	*42.9*
6.39E-02	*1.18E-03*	*40.9*
1.06E-01	*1.99E-03*	*39.4*
1.75E-01	*3.36E-03*	*38.4*

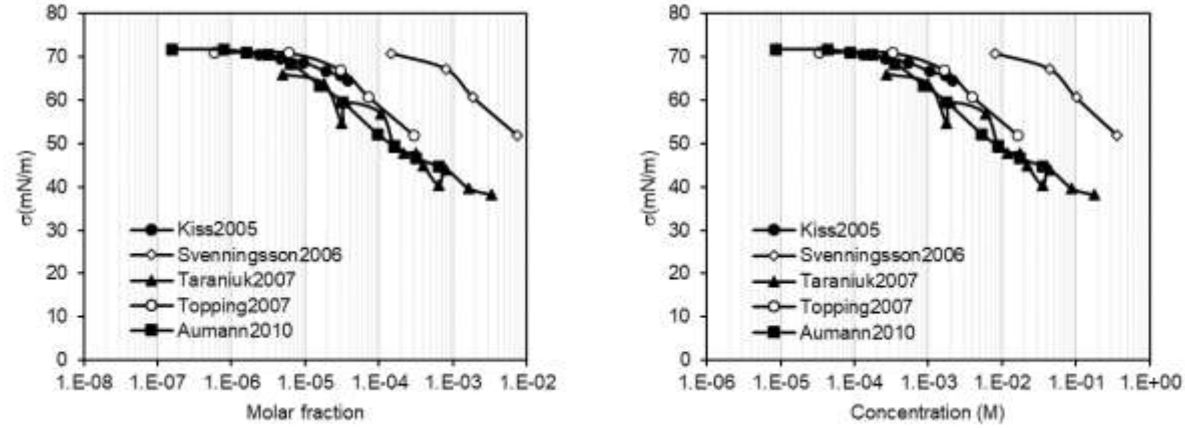

Fig. S128 (a): Experimental data for SRFA / water mixtures.

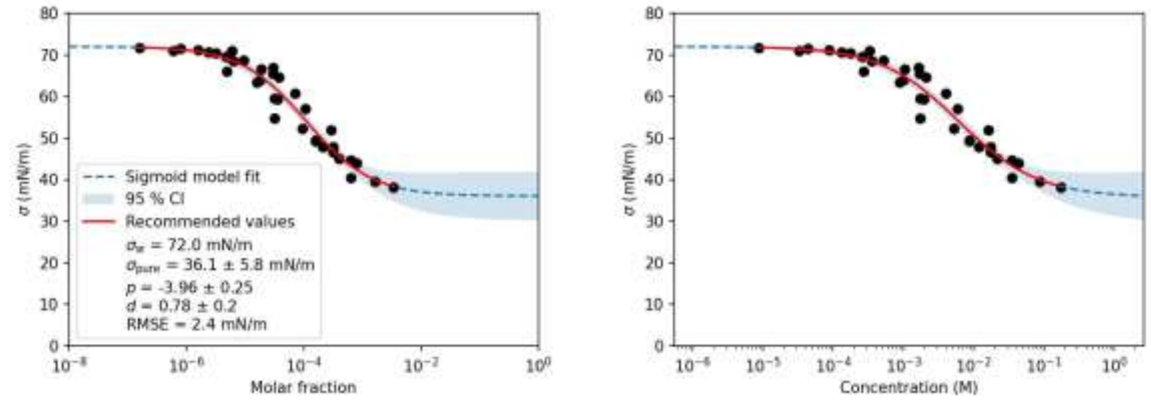

Fig. S128 (b): Surface tension fit with the Sigmoid model (*Kleinheins et al. 2023*) for SRFA / water mixtures. Solid red line: model fit inside the concentration range covered by experimental data, reported as recommended values. Blue shading: fit parameters with 95 % confidence interval (CI). RMSE: root mean squared error. Markers: data used for fitting.

Comment:

The concentration C(M) and the molar fraction are calculated assuming a molecular weight of 570 g/mol and a density of 1.5 g/cm^3 (*Dinar et al. 2006*).

Dinar et al., *The density of humic acids and humic like substances (HULIS) from fresh and aged wood burning and pollution aerosol particles*, Atmos. Chem. Phys., **2006**, *6*, 5213–5224.

The data from *Svenningsson et al. 2006* were not included for the fitting, because they differ from the other surface tension isotherms.

MA2: NAFA (Nordic aquatic fulvic acid)

Kiss et al. 2005 **Graph (experimental points)**				Kristensen et al. 2014 **Graph (experimental points)**			
**C (M)**	**Molar fraction**	**C (g/L)**	**σ (mN/m)**	**C (M)**	**Molar fraction**	**C (g/L)**	**σ (mN/m)**
0.00E-05	0.00E-07	**0.00E-02**	71.7	2.21E-05	3.98E-07	**9.42E-02**	71.6
1.77E-05	3.19E-07	**7.55E-02**	71.7	5.51E-05	9.92E-07	**2.35E-01**	70.9
3.52E-05	6.33E-07	**1.50E-01**	71.4	1.06E-04	1.91E-06	**4.53E-01**	69.6
7.03E-05	1.27E-06	**3.00E-01**	70.1	2.89E-04	5.20E-06	**1.23E+00**	65.6
1.41E-04	2.53E-06	**5.99E-01**	67.8	7.32E-04	1.32E-05	**3.12E+00**	58.6
2.27E-04	4.09E-06	**9.69E-01**	64.6	1.46E-03	2.63E-05	**6.21E+00**	55.2
2.83E-04	5.09E-06	**1.21E+00**	63.4				

Lin et al. 2020 **Graph (experimental points)**				***Recommended***		
**C (M)**	**Molar fraction**	**C (g/L)**	**σ (mN/m)**	***C (M)***	***Molar fraction***	***σ (mN/m)***
2.19E-05	3.94E-07	**9.35E-02**	71.0	*1.77E-05*	*3.19E-07*	*71.6*
5.50E-05	9.89E-07	**2.34E-01**	70.1	*2.24E-05*	*4.02E-07*	*71.5*
1.05E-04	1.90E-06	**4.49E-01**	66.9	*2.82E-05*	*5.08E-07*	*71.3*
2.88E-04	5.19E-06	**1.23E+00**	62.3	*3.56E-05*	*6.40E-07*	*71.1*
7.36E-04	1.33E-05	**3.14E+00**	55.5	*4.49E-05*	*8.08E-07*	*70.7*
1.45E-03	2.62E-05	**6.19E+00**	52.5	*5.66E-05*	*1.02E-06*	*70.4*
				7.14E-05	*1.28E-06*	*69.8*
				9.00E-05	*1.62E-06*	*69.2*
				1.14E-04	*2.04E-06*	*68.4*
				1.43E-04	*2.58E-06*	*67.4*
				1.81E-04	*3.25E-06*	*66.3*
				2.28E-04	*4.10E-06*	*65.0*
				2.87E-04	*5.18E-06*	*63.5*
				3.62E-04	*6.53E-06*	*61.9*
				4.57E-04	*8.24E-06*	*60.3*
				5.76E-04	*1.04E-05*	*58.8*
				7.27E-04	*1.31E-05*	*57.3*
				9.16E-04	*1.65E-05*	*55.9*
				1.15E-03	*2.08E-05*	*54.8*
				1.46E-03	*2.63E-05*	*53.8*

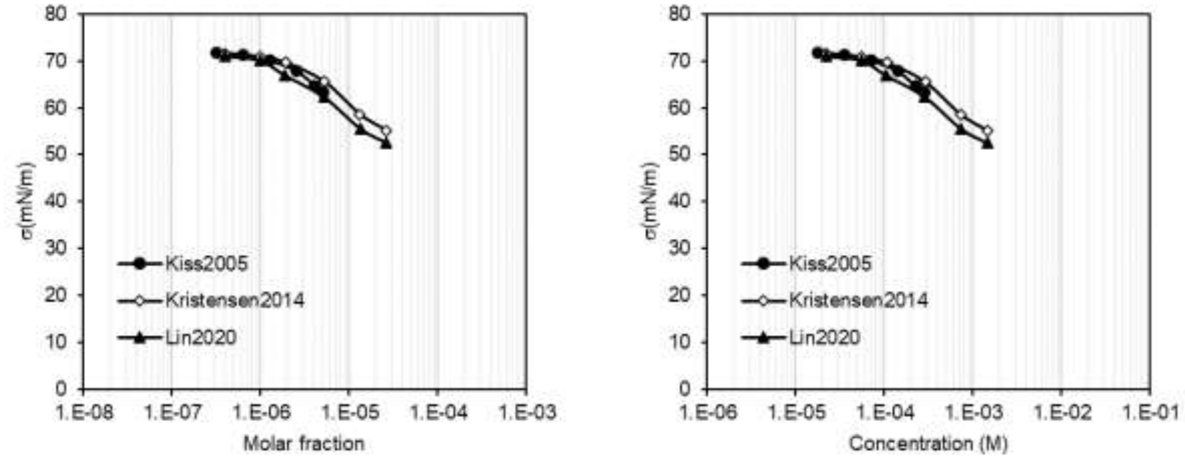

Fig. S129 (a): Experimental data for NAFA / water mixtures.

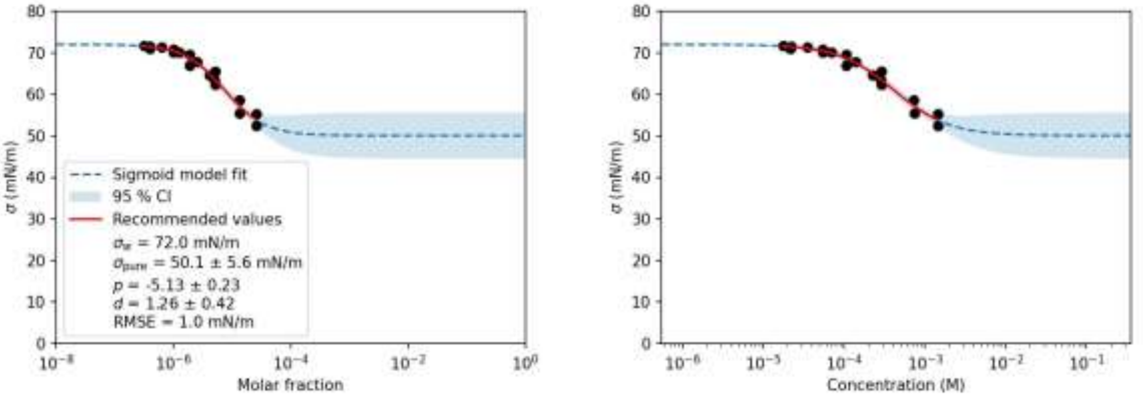

Fig. S129 (b): Surface tension fit with the Sigmoid model (*Kleinheins et al. 2023*) for NAFA / water mixtures. Solid red line: model fit inside the concentration range covered by experimental data, reported as recommended values. Blue shading: fit parameters with 95 % confidence interval (CI). RMSE: root mean squared error. Markers: data used for fitting.

Comment:

The concentration C(M) and the molar fraction are calculated assuming a molecular weight of 4266 g/mol (*Mäkela et al. 2008*) and a density of 1.5 g/cm^3 (*Dinar et al. 2006*, for SRFA).

Mäkela et al., *Molecular Size Distribution and Structure Investigations of Humic Substances in Groundwater*, Working Report 2008-36, Posiva Oy, **2008**, 1-21.

Dinar et al., *The density of humic acids and humic like substances (HULIS) from fresh and aged wood burning and pollution aerosol particles*, Atmos. Chem. Phys., **2006**, *6*, 5213–5224.

MA3: Humic acid

Guetzloff et al. 1994		**Graph (experimental points)**	
**C (M)**	**Molar fraction**	**C (g/L)**	**σ (mN/m)**
2.33E-04	4.19E-06	**5.3E-02**	63.9
2.23E-03	4.01E-05	**5.0E-01**	55.2
1.10E-02	1.98E-04	**2.5E+00**	50.3
2.21E-02	3.99E-04	**5.0E+00**	49.5
2.53E-02	4.58E-04	**5.7E+00**	49.8
3.04E-02	5.49E-04	**6.9E+00**	50.0
3.24E-02	5.86E-04	**7.3E+00**	48.2
3.92E-02	7.09E-04	**8.9E+00**	48.4
4.69E-02	8.50E-04	**1.1E+01**	48.1
5.27E-02	9.54E-04	**1.2E+01**	48.1

Tuckermann et al. 2004		**Graph (experimental points)**	
**C (M)**	**Molar fraction**	**C (g/L)**	**σ (mN/m)**
4.42E-06	7.96E-08	**1.00E-03**	73.4
8.84E-06	1.59E-07	**2.00E-03**	73.4
1.81E-05	3.26E-07	**4.10E-03**	73.4
3.63E-05	6.53E-07	**8.20E-03**	73.5
7.30E-05	1.31E-06	**1.65E-02**	73.2
1.46E-04	2.63E-06	**3.30E-02**	73.0
3.02E-04	5.44E-06	**6.84E-02**	73.3
5.88E-04	1.06E-05	**1.33E-01**	72.8
1.16E-03	2.09E-05	**2.62E-01**	72.4
2.36E-03	4.25E-05	**5.34E-01**	71.4
4.42E-03	7.96E-05	**1.00E+00**	67.7
8.84E-03	1.59E-04	**2.00E+00**	64.3
2.21E-02	3.99E-04	**5.00E+00**	58.7

Aumann et al. 2010 HA1		**Graph (experimental points)**	
**C (M)**	**Molar fraction**	**C (g/L)**	**σ (mN/m)**
4.42E-06	7.96E-08	**1.00E-03**	72.0
2.21E-05	3.98E-07	**5.00E-03**	71.9
4.42E-05	7.96E-07	**1.00E-02**	71.8
2.21E-04	3.98E-06	**5.00E-02**	71.5
4.42E-04	7.96E-06	**1.00E-01**	71.5
8.84E-04	1.59E-05	**2.00E-01**	70.7
1.77E-03	3.18E-05	**4.00E-01**	70.1
4.42E-03	7.96E-05	**1.00E+00**	66.1
1.11E-02	1.99E-04	**2.50E+00**	60.1
2.21E-02	3.99E-04	**5.00E+00**	56.5
4.42E-02	8.01E-04	**1.00E+01**	54.0
8.84E-02	1.61E-03	**2.00E+01**	52.5

Aumann et al. 2010 HA2		**Graph (experimental points)**	
**C (M)**	**Molar fraction**	**C (g/L)**	**σ (mN/m)**
4.42E-06	7.96E-08	**1.00E-03**	72.0
2.21E-05	3.98E-07	**5.00E-03**	71.9
4.42E-05	7.96E-07	**1.00E-02**	71.9
2.21E-04	3.98E-06	**5.00E-02**	71.7
4.42E-04	7.96E-06	**1.00E-01**	71.5
8.84E-04	1.59E-05	**2.00E-01**	71.5
1.77E-03	3.18E-05	**4.00E-01**	71.2
4.42E-03	7.96E-05	**1.00E+00**	70.7
1.11E-02	1.99E-04	**2.50E+00**	70.2
2.21E-02	3.99E-04	**5.00E+00**	69.4
4.42E-02	8.01E-04	**1.00E+01**	67.6
8.84E-02	1.61E-03	**2.00E+01**	65.8

Klavins et al.et al. 2010		**Graph (experimental points)**	
**C (M)**	**Molar fraction**	**C (mg/L)**	**σ (mN/m)**
2.21E-04	3.98E-06	**5.00E+01**	65.2
4.42E-04	7.96E-06	**1.00E+02**	63.4
6.63E-04	1.19E-05	**1.50E+02**	62.6
1.11E-03	1.99E-05	**2.50E+02**	61.9
1.33E-03	2.39E-05	**3.00E+02**	61.5
1.55E-03	2.79E-05	**3.50E+02**	61.1
1.77E-03	3.18E-05	**4.00E+02**	60.4
2.21E-03	3.98E-05	**5.00E+02**	59.8
2.87E-03	5.18E-05	**6.50E+02**	58.9
3.32E-03	5.97E-05	**7.50E+02**	58.5
4.42E-03	7.96E-05	**1.00E+03**	57.1

Recommended		
C (M)	***Molar fraction***	***σ (mN/m)***
4.42E-06	*7.96E-08*	*71.9*
7.45E-06	*1.34E-07*	*71.8*
1.26E-05	*2.26E-07*	*71.8*
2.12E-05	*3.81E-07*	*71.6*
3.57E-05	*6.42E-07*	*71.5*
6.01E-05	*1.08E-06*	*71.3*
1.01E-04	*1.82E-06*	*70.9*
1.71E-04	*3.07E-06*	*70.4*
2.88E-04	*5.18E-06*	*69.8*
4.84E-04	*8.72E-06*	*68.9*
8.16E-04	*1.47E-05*	*67.7*
1.38E-03	*2.48E-05*	*66.3*
2.32E-03	*4.17E-05*	*64.5*
3.90E-03	*7.03E-05*	*62.6*

	6.58E-03	*1.18E-04*	*60.6*
	1.11E-02	*2.00E-04*	*58.7*
	1.86E-02	*3.36E-04*	*57.0*
	3.14E-02	*5.67E-04*	*55.6*
	5.27E-02	*9.55E-04*	*54.5*
	8.84E-02	*1.61E-03*	*53.6*

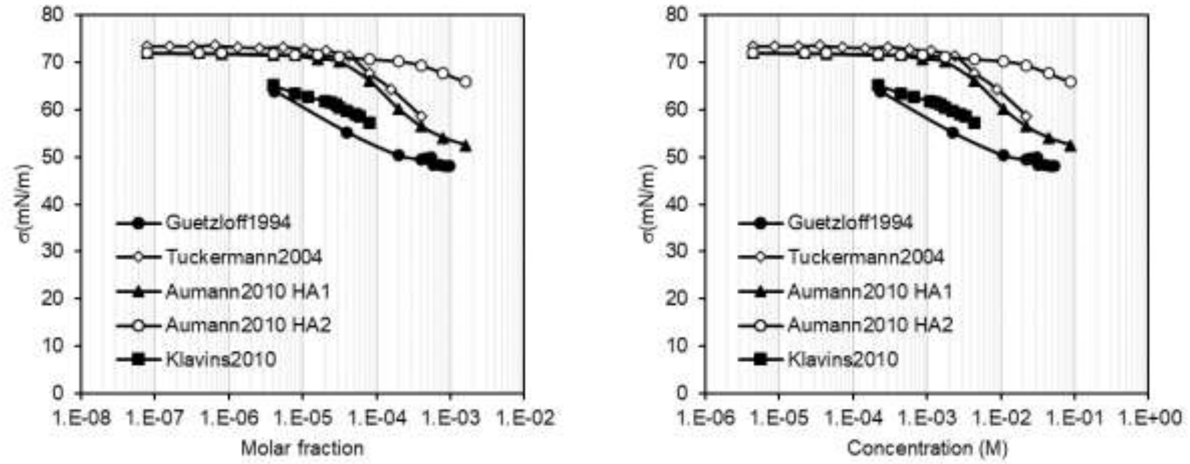

Fig. S130 (a): Experimental data for humic acid / water mixtures.

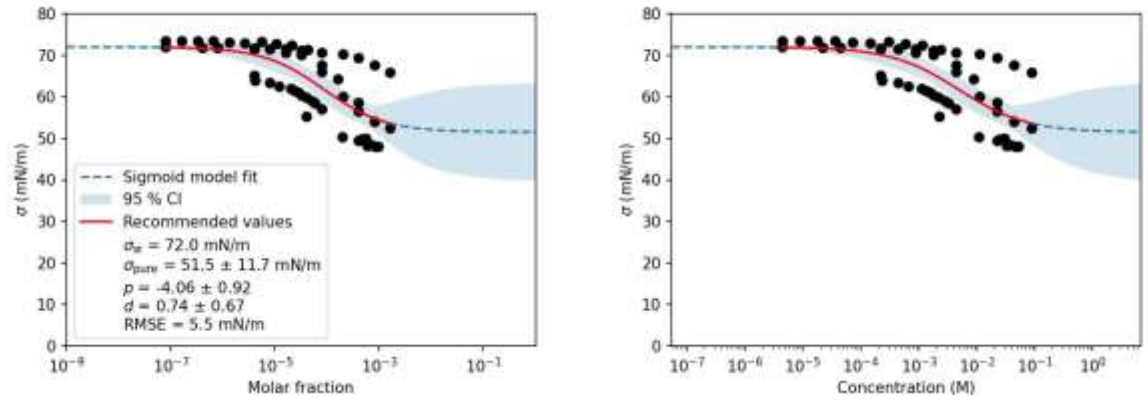

Fig. S130 (b): Surface tension fit with the Sigmoid model (*Kleinheins et al. 2023*) for humic acid / water mixtures. Solid red line: model fit inside the concentration range covered by experimental data, reported as recommended values. Blue shading: fit parameters with 95 % confidence interval (CI). RMSE: root mean squared error. Markers: data used for fitting.

Comment:

The concentration C(M) and the molar fraction are calculated assuming a molecular weight of 226.14 g/mol (FischerScientific) and a density of 1.5 g/cm^3 (*Dinar et al. 2006*, for SRFA).

Differences between the different references may come from the purity of the compounds.

MA4: HULIS (Humic acid like substances) extracted from atmospheric aerosols

Kiss et al. 2005 **(average of 6 sets (different dates), experimental dots)**				Salma et al. 2006 **Graph (experimental points)**			
**C (M)**	**Molar fraction**	**C (g/L)**	**σ (mN/m)**	**C (M)**	**Molar fraction**	**C (mg/L)**	**σ (mN/m)**
0.00E+00	0.00E+00	**0.00E+00**	71.7	0.00E+00	0.00E+00	**0.00E+00**	72.7
7.89E-05	1.42E-06	**4.00E-02**	64.0	8.61E-05	1.55E-06	**4.37E+01**	59.6
1.21E-04	2.17E-06	**6.13E-02**	62.3	1.72E-04	3.09E-06	**8.70E+01**	54.1
1.71E-04	3.08E-06	**8.67E-02**	61.0	3.48E-04	6.27E-06	**1.77E+02**	53.1
2.34E-04	4.22E-06	**1.19E-01**	59.4	7.05E-04	1.27E-05	**3.57E+02**	52.1
3.31E-04	5.97E-06	**1.68E-01**	57.5	1.07E-03	1.92E-05	**5.42E+02**	51.1
4.59E-04	8.26E-06	**2.33E-01**	56.1	1.61E-03	2.90E-05	**8.17E+02**	50.2
6.50E-04	1.17E-05	**3.29E-01**	54.1	2.02E-03	3.63E-05	**1.02E+03**	49.6
8.90E-04	1.60E-05	**4.51E-01**	52.1				
1.19E-03	2.15E-05	**6.05E-01**	50.3				
1.63E-03	2.94E-05	**8.29E-01**	48.3				
2.14E-03	3.86E-05	**1.09E+00**	46.7				

Taraniuk et al. 2007 HULIS 1 **Graph (experimental points)**				Taraniuk et al. 2007 HULIS 2 **Graph (experimental points)**			
**C (M)**	**Molar fraction**	**ln (C mol/m^3)**	**σ (mN/m)**	**C (M)**	**Molar fraction**	**ln (C mol/m^3)**	**σ (mN/m)**
1.40E-03	2.52E-05	**3.35E-01**	69.7	4.82E-04	8.68E-06	**-7.30E-01**	70.1
2.00E-03	3.60E-05	**6.92E-01**	54.3	9.70E-04	1.75E-05	**-3.04E-02**	58.9
3.96E-03	7.14E-05	**1.38E+00**	48.8	2.42E-03	4.35E-05	**8.82E-01**	41.5
6.02E-03	1.09E-04	**1.79E+00**	41.4				

Taraniuk et al. 2007 HULIS 3 **Graph (experimental points)**				***Recommended***		
**C (M)**	**Molar fraction**	**ln (C mol/m^3)**	**σ (mN/m)**	***C (M)***	***Molar fraction***	***σ (mN/m)***
1.99E-04	3.59E-06	**-1.61E+00**	69.6	*7.89E-05*	*1.42E-06*	*63.1*
5.01E-04	9.01E-06	**-6.92E-01**	51.9	*9.91E-05*	*1.78E-06*	*62.4*
9.92E-04	1.79E-05	**-7.60E-03**	46.8	*1.25E-04*	*2.24E-06*	*61.7*
2.00E-03	3.60E-05	**6.92E-01**	43.3	*1.57E-04*	*2.82E-06*	*61.0*
2.01E-03	3.63E-05	**7.00E-01**	42.9	*1.97E-04*	*3.54E-06*	*60.2*
				2.47E-04	*4.45E-06*	*59.4*
				3.11E-04	*5.59E-06*	*58.5*
				3.90E-04	*7.03E-06*	*57.6*
				4.91E-04	*8.83E-06*	*56.6*
				6.16E-04	*1.11E-05*	*55.5*
				7.75E-04	*1.39E-05*	*54.4*
				9.73E-04	*1.75E-05*	*53.2*
				1.22E-03	*2.20E-05*	*52.0*
				1.54E-03	*2.77E-05*	*50.7*
				1.93E-03	*3.48E-05*	*49.3*
				2.43E-03	*4.37E-05*	*47.9*
				3.05E-03	*5.49E-05*	*46.3*
				3.83E-03	*6.90E-05*	*44.7*
				4.81E-03	*8.67E-05*	*43.1*
				6.04E-03	*1.09E-04*	*41.3*

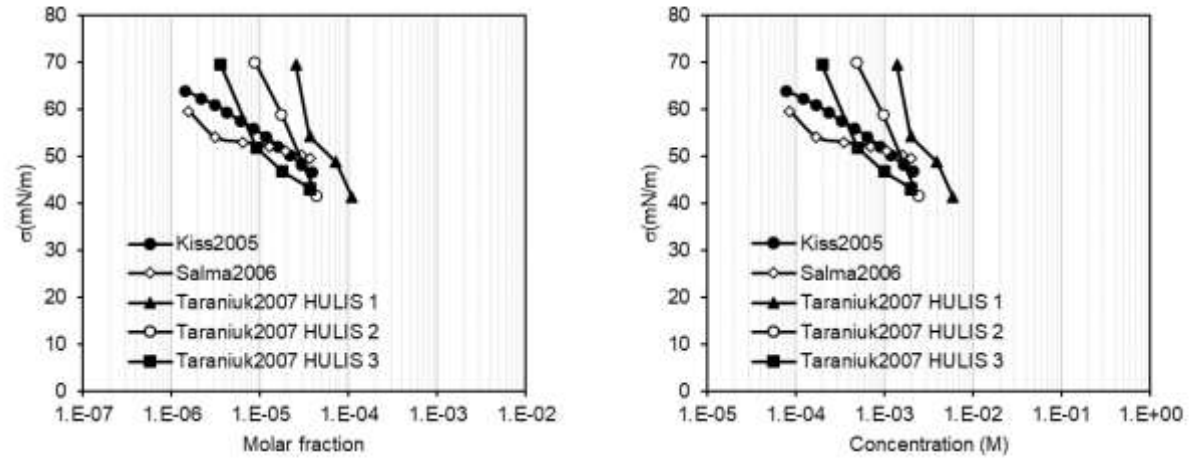

Fig. S131 (a): Experimental data for extracted HULIS / water mixtures.

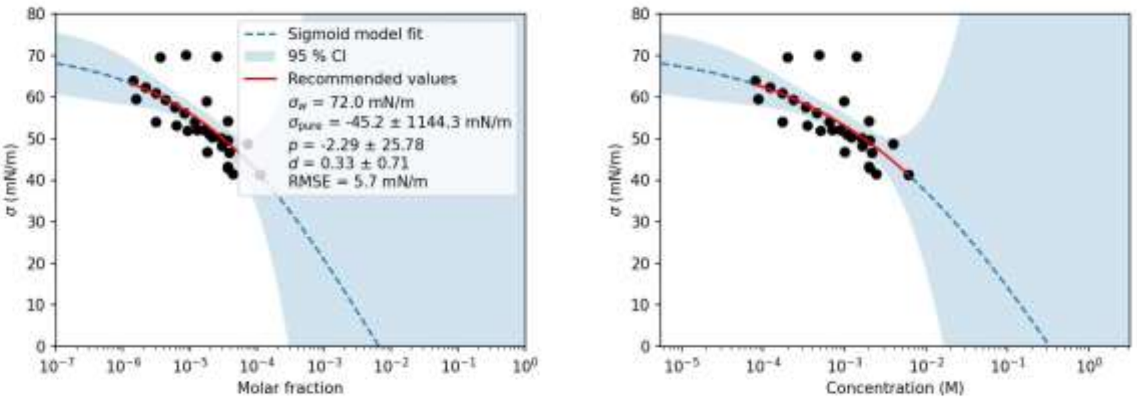

Fig. S131 (b): Surface tension fit with the Sigmoid model (*Kleinheins et al. 2023*) for extracted HULIS / water mixtures. Solid red line: model fit inside the concentration range covered by experimental data, reported as recommended values. Blue shading: fit parameters with 95 % confidence interval (CI). RMSE: root mean squared error. Markers: data used for fitting. **Note: the modelling is reliable only in the range of the experimental data. Therefore, for this compound, the modelling should be used only in the experimental data range.**

Comment:

Salma et al. 2006: PM2.5 urban-type atmospheric aerosol
Kiss et al. 2005: PM1.5 tropospheric fine aerosol HULIS
Taraniuk et al. 2007 HULIS 1 : PM10 pollution HULIS, M = 500 g/mol
Taraniuk et al. 2007 HULIS 2: PM10 aged biomass burning HULIS, M = 410 g/mol
Taraniuk et al. 2007 HULIS 3: PM10 fresh biomass burning HULIS, M = 610 g/mol

The concentration C(M) and the molar fraction are calculated assuming a density of 1.6 g/cm^3 (*Dinar et al. 2006*, for extracted atmospheric HULIS).

For *Salma et al. 2006* and *Kiss et al. 2005*, the concentration C(M) and the molar fraction are calculated assuming a molecular weight of 507 g/mol (*Taraniuk et al. 2007*).

The variety of atmospheric aerosols can explain the differences between the surface tension isotherms.

MA5: EPS (Extracellular polymeric substances)

Radchenkova et al. 2015 **Table**				Schwehr et al. 2018 **Graph (experimental points)**			
**C (M)**	**Molar fraction**	**C (m/vw %)**	**σ (mN/m)**	**C (M)**	**Molar fraction**	**ln (C BSA mg/L)**	**σ (mN/m)**
0.00E+00	0.00E+00	**0.00**	72	2.13E-09	3.84E-11	**-1.96E+00**	73.3
1.58E-06	2.85E-08	**0.25**	60	2.99E-09	5.38E-11	**-1.62E+00**	73.8
3.16E-06	5.70E-08	**0.50**	57	5.95E-09	1.07E-10	**-9.35E-01**	73.8
6.33E-06	1.14E-07	**1.00**	56	8.97E-09	1.62E-10	**-5.24E-01**	73.9
9.49E-06	1.71E-07	**1.50**	54	1.19E-08	2.14E-10	**-2.42E-01**	73.9
1.27E-05	2.28E-07	**2.00**	52	1.48E-08	2.66E-10	**-2.60E-02**	74.0
				1.48E-08	2.66E-10	**-2.60E-02**	73.7
				1.79E-08	3.21E-10	**1.65E-01**	74.0
				2.09E-08	3.76E-10	**3.20E-01**	74.1
				2.07E-08	3.72E-10	**3.12E-01**	73.2
				2.94E-08	5.29E-10	**6.62E-01**	73.6
				4.45E-08	8.01E-10	**1.08E+00**	73.1
				6.00E-08	1.08E-09	**1.38E+00**	70.2
				7.49E-08	1.35E-09	**1.60E+00**	69.1
				9.06E-08	1.63E-09	**1.79E+00**	67.5
				1.05E-07	1.89E-09	**1.94E+00**	67.7
				1.19E-07	2.14E-09	**2.06E+00**	67.1
				1.35E-07	2.43E-09	**2.19E+00**	67.1
				1.50E-07	2.69E-09	**2.29E+00**	66.7
				1.50E-07	2.70E-09	**2.29E+00**	68.0
				2.99E-07	5.38E-09	**2.98E+00**	67.4
				4.51E-07	8.12E-09	**3.39E+00**	67.2
				6.06E-07	1.09E-08	**3.69E+00**	66.1
				7.59E-07	1.37E-08	**3.91E+00**	66.6

Maulana et al. 2022 EPS1 **Graph (experimental points)**				Maulana et al. 2022 EPS2 **Graph (experimental points)**			
**C (M)**	**Molar fraction**	**C (m/vw %)**	**σ (mN/m)**	**C (M)**	**Molar fraction**	**C (m/vw %)**	**σ (mN/m)**
0.00E+00	0.00E+00	**0.0**	73.0	0.00E+00	0.00E+00	**0.0**	73.0
6.33E-07	1.14E-08	**0.1**	68.8	6.33E-07	1.14E-08	**0.1**	66.7
1.27E-06	2.28E-08	**0.2**	65.7	1.27E-06	2.28E-08	**0.2**	65.1
3.16E-06	5.70E-08	**0.5**	62.1	3.16E-06	5.70E-08	**0.5**	60.2
6.33E-06	1.14E-07	**1.0**	59.8	6.33E-06	1.14E-07	**1.0**	60.1
1.27E-05	2.28E-07	**2.0**	59.6	1.27E-05	2.28E-07	**2.0**	61.5

Recommended		
C (M)	***Molar fraction***	***σ (mN/m)***
2.13E-09	*3.84E-11*	*71.9*
3.37E-09	*6.07E-11*	*71.9*
5.32E-09	*9.58E-11*	*71.8*
8.41E-09	*1.51E-10*	*71.7*
1.33E-08	*2.39E-10*	*71.6*
2.10E-08	*3.78E-10*	*71.4*

3.32E-08	*5.97E-10*	*71.1*
5.24E-08	*9.43E-10*	*70.8*
8.28E-08	*1.49E-09*	*70.3*
1.31E-07	*2.35E-09*	*69.7*
2.07E-07	*3.72E-09*	*68.8*
3.26E-07	*5.88E-09*	*67.8*
5.16E-07	*9.28E-09*	*66.5*
8.15E-07	*1.47E-08*	*65.0*
1.29E-06	*2.32E-08*	*63.4*
2.03E-06	*3.66E-08*	*61.8*
3.21E-06	*5.78E-08*	*60.3*
5.07E-06	*9.14E-08*	*58.9*
8.01E-06	*1.44E-07*	*57.8*
1.26E-05	*2.28E-07*	*56.8*

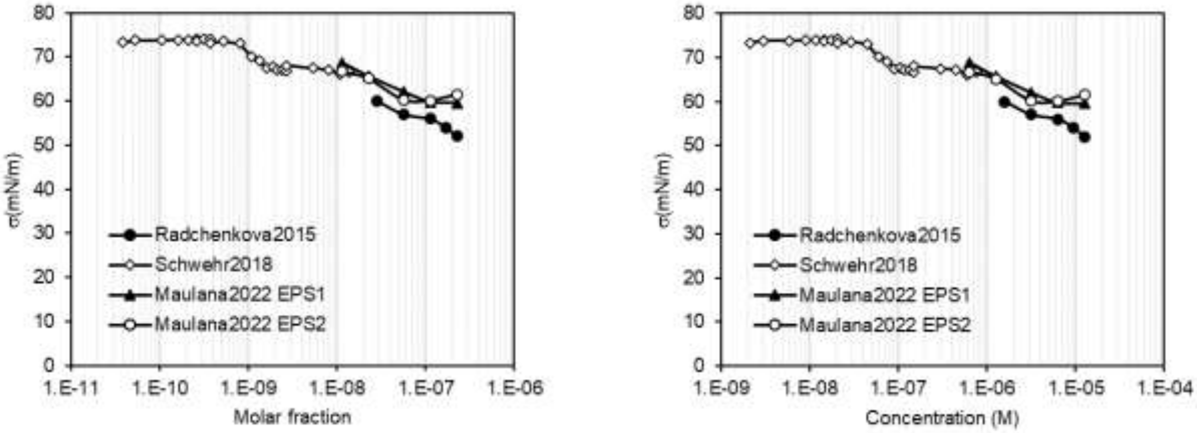

Fig. S132 (a): Experimental data for extracellular polymeric substances / water mixtures.

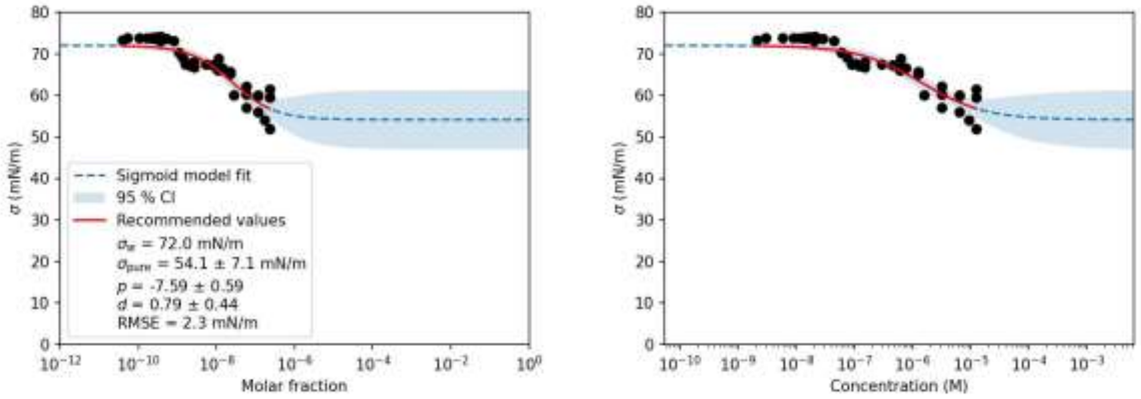

Fig. S132 (b): Surface tension fit with the Sigmoid model (*Kleinheins et al. 2023*) for extracellular polymeric substances / water mixtures. Solid red line: model fit inside the concentration range covered by experimental data, reported as recommended values. Blue shading: fit parameters with 95 % confidence interval (CI). RMSE: root mean squared error. Markers: data used for fitting.

Comment:

Radchenkova et al. 2015, *Maulana et al. 2022* EPS1 and 2 : The concentration C(M) and the molar fraction are calculated assuming a molecular weight of 158kDa (158 kg/mol) (=average value calculated from the data reported by *Rehman et al. 2021* for EPS) and a density of 1.

Rehman et al., *Physicochemical Properties of Extracellular Polymeric Substances Produced by Three Bacterial Isolates From Biofouled Reverse Osmosis Membranes*, Front. Microbiol., **2021**, *12*, 668761 https://doi.org/10.3389/fmicb.2021.668761

Radchenkova et al. 2015: exopolysaccharide produced from *Aeribacillus pallidus.*

Schwehr et al. 2018 : solution modeling extracellular polymeric substances (EPS) concentrated at the air-water interface of seawater. The solution includes Bovine Serum Albumin (BSA) (molecular weight of 66kDa).

Maulana et al. 2022: extracted extracellular polymeric substances from textile wastewater activated sludges.
